# Hydrogen‐Bond‐Enabled Dynamic Kinetic Resolution of Axially Chiral Amides Mediated by a Chiral Counterion

**DOI:** 10.1002/anie.201814362

**Published:** 2019-02-06

**Authors:** Alison J. Fugard, Antti S. K. Lahdenperä, Jaqueline S. J. Tan, Aroonroj Mekareeya, Robert S. Paton, Martin D. Smith

**Affiliations:** ^1^ Chemistry Research Laboratory University of Oxford 12 Mansfield Road Oxford OX1 3TA UK; ^2^ Department of Chemistry Colorado State University Fort Collins CO 80523 USA

**Keywords:** axial chirality, counterion, energy transfer, phase transfer, visible light

## Abstract

Non‐biaryl atropisomers are valuable in medicine, materials, and catalysis, but their enantioselective synthesis remains a challenge. Herein, a counterion‐mediated O‐alkylation method for the generation of atropisomeric amides with an er up to 99:1 is outlined. This dynamic kinetic resolution is enabled by the observation that the rate of racemization of atropisomeric naphthamides is significantly increased by the presence of an intramolecular O−H⋅⋅⋅NCO hydrogen bond. Upon O‐alkylation of the H‐bond donor, the barrier to rotation is significantly increased. Quantum calculations demonstrate that the intramolecular H‐bond reduces the rotational barrier about the aryl–amide bond, stabilizing the planar transition state for racemization by approximately 40 kJ mol^−1^, thereby facilitating the observed dynamic kinetic resolution.

Axially chiral molecules are of fundamental importance across a range of different fields including catalysis, medicine, and materials. Biphenyl derivatives with restricted rotation about the biaryl axis have been intensively investigated since the seminal report of Kenner and Christie in 1922^1^ and are exemplars of the field, both in their study and their applications. More recently, non‐biaryl atropisomers including anilides, amides, and imides have also been investigated,^2^ amid a growing realization of the importance of these molecules in medicine^3^ and other fields, such as catalysis.^4^ Appropriately substituted tertiary amides can possess significant barriers to rotation,^5^ and this has been exploited for stereoselective *ortho*‐functionalization reactions^6^ and for long range stereocontrol.^7^ Several approaches to the catalytic enantioselective synthesis of atropisomeric amides have been disclosed including metal‐catalysed^8^ and organocatalytic methods.^9^ Walsh has described an enantioselective proline‐catalysed aldol reaction on a naphthamide‐derived aldehyde, and Miller has demonstrated an elegant peptide catalysed atropselective bromination.^10^ More recently, the Sparr group described a proline‐catalysed aldol‐elimination procedure for the enantioselective synthesis of axially chiral aromatic amides.^11^ As part of a programme focused on the catalytic enantioselective synthesis of axially chiral molecules, we observed that the barrier to rotation of certain napthamides was dependent on the presence of a hydrogen‐bond donor proximal to the amide group (Figure 1). Napthamide **1**, which bears a 2‐hydroxy group, has a barrier to rotation about the C_aryl_–C_amide_ bond of 97.5 kJ mol^−1^ at 298 K in CH_2_Cl_2_ solution, which is close to the boundary for atropisomerism as defined by Oki.^12^ In contrast, amide **2**, in which the 2‐naphthol is derivatized as a benzyl ether, has a significantly higher barrier to rotation of 129.3 kJ mol^−1^ (394 K in *m*‐xylene), which is sufficient to essentially preclude rotation at ambient temperature. As the steric difference between an OH group and OBn group is too small to explain this observation, we postulated that the difference in rotational barrier was likely due to the presence of an intramolecular hydrogen bond between the naphthol OH and the amide nitrogen. In principle, this could stabilize the planar transition state for the interconversion of the two enantiomeric forms.^13^ To probe this, the barrier to rotation of **1** about the C_aryl_–C_amide_ bond in isopropanol, as a hydrogen‐bonding solvent, was also determined. The measured barrier was 113.6 kJ mol^−1^ (298 K), a significant increase versus the barrier in CH_2_Cl_2_. This is consistent with solvation of the phenolic group leading to the disruption of the intramolecular hydrogen bond and an overall increase in size.^14^ We reasoned that the presence of this hydrogen bond offered an opportunity to carry out an enantioselective synthesis of axially chiral amides via atropselective *O*‐functionalization.^15^, ^16^ In this scenario, the increased barrier to rotation of *O*‐functionalized materials precludes room temperature racemization and enables a dynamic kinetic resolution. To probe the feasibility of this procedure, we needed access to a model atropisomeric amide, and this was synthesized through a three‐step procedure from cheap and readily available 1‐naphthylacetic acid using a key photo‐mediated Dieckmann condensation (Scheme 1).

**Figure 1:**
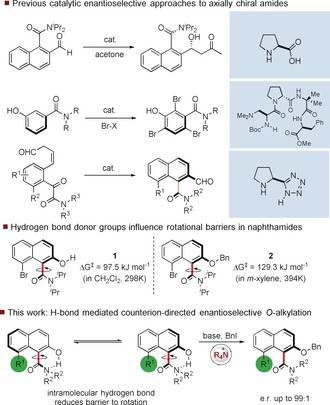
Previous work and approach to dynamic kinetic resolution of atropisomeric naphthamides.

**Scheme 1:**
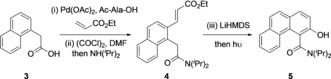
Synthesis of atropisomeric amides. Reaction conditions: i) Pd(OAc)_2_ (0.1 equiv), Ac‐Ala‐OH (0.2 equiv), ethyl acrylate (2.0 equiv), KHCO_3_ (2.0 equiv), 2‐methyl butan‐2‐ol, 90 °C. ii) (COCl)_2_ (2.0 equiv), DMF (0.1 equiv), CH_2_Cl_2_; then NH(^*i*^Pr)_2_ (2 equiv). iii) LiHMDS, (2.1 equiv), THF, 0 °C, 5 mins; then 12 W blue LED, RT, 30 mins. Yields are for isolated and purified materials.

A palladium(II)‐mediated oxidative Heck reaction using the method disclosed by Yu^17^ enabled the selective C‐2 functionalization of **3** in 92 % yield, and this was transformed into amide **4** via the intermediacy of an acid chloride in 63 % yield. The alkene in **4** is incorrectly configured for the Dieckmann reaction, but we reasoned that a cascade energy transfer^18^ isomerization‐cyclization process could be effective.^19^ Treatment of **4** with 2.1 equivalents LiHMDS in THF followed by irradiation with blue LED light afforded 2‐naphthol **5** in 77 % yield. This is a scalable and operationally simple procedure and demonstrates the compatibility of visible‐light isomerization with strong anionic bases.

With this material in hand, the catalytic enantioselective *O*‐alkylation of **5** was examined with a range of different conditions and ammonium salts (Table 1).^20^ Upon exposure of **5** to tetrabutylammonium bromide (TBAB) and 50 % aqueous cesium carbonate in toluene at room temperature, *O*‐alkylation occurred to afford **6**. The use of *N*‐benzyl cinchoninium chloride **7** and aqueous cesium carbonate gave good conversion to the desired product with an e.r. of 77:23 (Table 1, entry 1), and we subsequently explored a range of different *N*‐aryl groups on this catalyst scaffold. *N*‐Anthracenylmethyl catalyst **8** was less selective (72:28 e.r.), but bistrifluoromethylaryl catalyst **9** afforded an augmented e.r. (82:18). Based on this, the effect of substitution in the 3‐ and 5‐positions on the aryl ring was explored. 3,5‐Bis‐*tert*‐butyl catalyst **10** was incrementally more selective, which may reflect increased solubility in the organic phase. To explore this further, catalyst **11**, which bears three aryl groups and four *tert*‐butyl groups was evaluated; this afforded *O*‐benzylated product **6** with 83:17 e.r. (Table 1, entry 5).

**Table 1:** Optimization: atropselective *O*‐alkylation of naphthamides.^[a]^ 
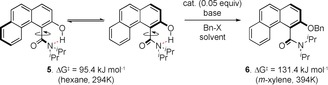

Entry	Cat.	Base^[b]^	Solvent	Bn‐X	e.r.^[c]^
1	**7**	Cs_2_CO_3_ (50 % aq.)	toluene	BnBr	77:23
2	**8**	Cs_2_CO_3_ (50 % aq.)	toluene	BnBr	72:28
3	**9**	Cs_2_CO_3_ (50 % aq.)	toluene	BnBr	82:18
4	**10**	Cs_2_CO_3_ (50 % aq.)	toluene	BnBr	87:13
5	**11**	Cs_2_CO_3_ (50 % aq.)	toluene	BnBr	83:17
6	**11**	KF (25 % aq.)	toluene	BnBr	93:7
7	**11**	KF (25 % aq.)	benzene	BnBr	94:6
8	**11**	KF (25 % aq.)	benzene	BnI	95:5
9	**11**	Cs_2_CO_3_ (50 % aq.)	benzene	BnI	96:4^[d]^
10	**11**	Cs_2_CO_3_ (50 % aq.)	benzene	BnI	97:3^[e]^

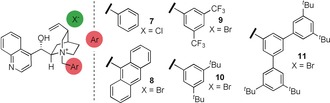

[a] Conditions: substrate **5** (0.02 mmol), catalyst (0.1 equiv), base (1.0 equiv), solvent ([substrate]=0.1 mol dm^−3^), RT, 48 h. [b] base: 50 % aq., w/w (10.0 equiv) [c] e.r. determined by chiral stationary phase HPLC. [d] [substrate]=0.025 mol dm^−3^. [e] [substrate]=0.01 mol dm^−3^.

A change of base to KF afforded a higher e.r. with catalyst **11** (93:7, entry 6, Table 1); screening solvents demonstrated that KF in benzene could lead to significantly higher enantioselectivity (e.r. 94:6, entry 7, Table 1), and a switch to benzyl iodide as electrophile resulted in a further increase in e.r. (95:5). We reasoned that slowing the rate of alkylation vs. the rate of racemization of the substrate through dilution could also be effective; ultimately this led to an increase in selectivity to 97:3 e.r. (Table 1, entry 10). With optimized conditions for the *O*‐alkylation established, the scope of the reaction was examined (Table 2). Clayden has demonstrated that 1‐ and 8‐substituted naphthamides can be axially chiral, and we focused on variations in these positions.^21^ Substrate **5** can be *O*‐alkylated to afford **6** in 93 % yield and 97:3 e.r. Changing the *N*‐alkyl group from *iso*‐propyl to cyclohexyl, as in **12**, is well tolerated (87 % yield, 98:2 e.r.).^22^ Substituting the 4‐position of the phenanthrenyl system with a methyl group, as in **13**, maintained selectivity (at 97:3 e.r. and 83 % yield). 8‐Substituted naphthyl systems are also well tolerated: substrates bearing electron donating substituents, such as 8‐methoxy (**14**, 96:4 e.r., 80 % yield), 8‐methyl (**15**, 98:2 e.r., 72 % yield), and 5,8‐dimethyl (**16**, 99:1 e.r. and 85 % yield), are all alkylated with good enantioselectivities and yields throughout. Substrates bearing electron withdrawing groups such as 8‐trifluoromethyl (**17**, 96:4 e.r., 99 % yield) are also *O*‐alkylated in high enantioselectivity. 8‐Aryl systems, such as **18** (98:2 e.r., 77 % yield), are similarly tolerated with high yield and high enantioselectivity. Substitution on this arene is also possible and substrates bearing *para*‐electron‐withdrawing groups, such as fluorine (**19**, 69 % yield, 98:2 e.r.), and electron‐donating groups, such as methoxy (**20**, 53 % yield, 98:2 e.r.), are also well accommodated.

**Table 2:** Enantioselective *O*‐alkylation of axially chiral amides.^[a]^

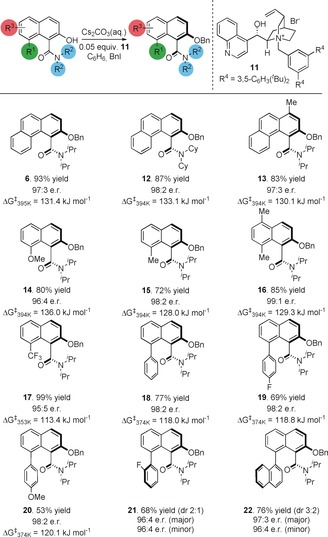

[a] Conditions: substrate (0.1–0.2 mmol), catalyst (0.05 equiv) Cs_2_CO_3_ (50 % aq., 5.0 equiv), solvent ([substrate]=0.01 mol dm^−3^), RT, 48 h. e.r. determined by chiral stationary phase HPLC. Yields are for isolated and purified materials. Rotational barriers measured in *m*‐xylene, at the temperature stated; see Supporting Information for full details.

The ability to incorporate substituents in the *ortho*‐position on this arene also enables the generation of compounds that possess multiple rotational axes.^23^ The *ortho*‐fluoro substituent in **21** restricts rotation about the biaryl axis to close to Oki's definition,^12^ so this material equilibrates relatively rapidly leading to a 2:1 diastereoisomeric mixture (96:4 e.r. for both diastereoisomers). The *O*‐alkylation of a substrate bearing a second rotational axis with a significantly higher barrier was also examined. The 8‐(1‐naphthyl) starting naphthol exists as a 2:1 mixture of racemic diastereoisomers. Treatment under our standard alkylation conditions led to **22**, a 3:2 mixture of diastereoisomers, in 97:3 e.r. for the major diastereoisomer, and 96:4 e.r. for the minor diastereoisomer. These two diastereoisomers do not interconvert at ambient temperature. The enantioselective synthesis of **21** and **22** demonstrates that the restricted rotation about the biaryl axis has little impact on the ability of the ammonium salt to effect the enantioselective *O*‐alkylation.

The value of axially chiral amides is most likely to be realized in their utility as building blocks for other purposes. To this end, we have demonstrated that the benzyl ether in **16** can be directly transformed to aryl silane **23** without loss of enantiopurity (Scheme 2).^24^

**Scheme 2:**
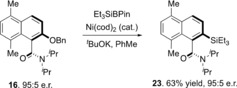
Direct functionalization of benzyl ether to aryl silane^[a]^ [a] Conditions: substrate (0.1 mmol), Et_3_SiBPin (1.3 equiv), Ni(cod)_2_ (0.1 equiv), ^*t*^BuOK (2.2 equiv), toluene ([substrate]=0.1 mol dm^−3^), RT, 3 h. e.r. determined by chiral stationary phase HPLC. Yields are for isolated and purified materials.

Treatment of benzyl ether **16** with triethylsilyl pinacol borane in the presence of a nickel(0) catalyst and *tert*‐butoxide afforded aryl silane **23** in 63 % yield and without any decrease in e.r.

To probe the influence of hydrogen bonding on the rate of racemization, we turned to quantum calculations. Compounds **24** and **25** (which bear a dimethyl‐ rather than a diisopropylamide) were modelled and the effect of *O*‐alkylation upon the rotational barrier was quantified (Figure 2).

**Figure 2:**
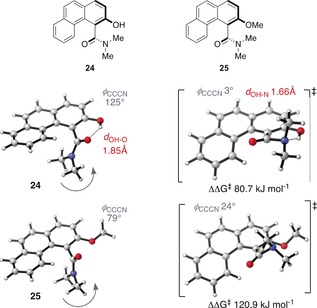
Optimized ground and transition state structures for model napthamides **24** and **25**.

Calculations were performed at the DLPNO‐CCSD(T)/def2‐TZPD//M062X/6‐31G(d) level, with SMD solvation for toluene and CH_2_Cl_2_. The increase in activation barrier, ΔΔ*G* ^≠^, from *O*‐alkylation of **24** is 39.7 kJ mol^−1^, which compares favorably with the experiment (36.0 kJ mol^−1^).^25^ An intramolecular OH–O H‐bond (1.85 Å) is present in the ground‐state structure of napthamide **24**. Rotation about the exocyclic C−C bond results in a transition structure (TS) with a non‐planar amide. In this structure, the pyramidalized nitrogen atom is in very close contact with the hydroxyl proton (1.66 Å). In the absence of a free naphthol, rotation proceeds via a TS in which the non‐planar amide cannot be similarly stabilized. Amide pyramidalization (a consequence of steric demands) enhances the hydrogen‐bond basicity of the nitrogen atom, leading to a stronger hydrogen bond in the transition structure than in the ground state, and contributes to a pronounced reduction in barrier height.

In conclusion, we have developed a highly enantioselective route to axially chiral napthamides. This approach relies on a transition‐state hydrogen bond to mediate substrate racemization that enables a dynamic kinetic resolution via *O*‐alkylation. These molecules may find application in supramolecular chemistry, catalysis, and medicinal chemistry programmes.

## Conflict of interest

The authors declare no conflict of interest.

## Supporting information

As a service to our authors and readers, this journal provides supporting information supplied by the authors. Such materials are peer reviewed and may be re‐organized for online delivery, but are not copy‐edited or typeset. Technical support issues arising from supporting information (other than missing files) should be addressed to the authors.

Supplementary
