# Supplementary material for: Hydrogen‐Bond‐Enabled Dynamic Kinetic Resolution of Axially Chiral Amides Mediated by a Chiral Counterion
Source: Angew Chem Int Ed Engl. 2019 Feb 6;58(9):2795–8. doi: 10.1002/anie.201814362 (PMC6492105; doi:10.1002/anie.201814362)
Supplement: Supplementary file 1 — Supplementary [file ANIE-58-2795-s001.pdf]

Angewandte Chemie
Eine Zeitschrift der Gesellschaft Deutscher Chemiker

Supporting Information

Hydrogen Bond-Enabled Dynamic Kinetic Resolution of Axially Chiral Amides Mediated by a Chiral Counterion

Alison J. Fugard, Antti S. K. Lahdenperä, Jaqueline S. J. Tan, Aroonroj Mekareeya, Robert S. Paton, and Martin D. Smith**

anie_201814362_sm_miscellaneous_information.pdf

Supporting Information

Contents

1.1 General Information S2
1.2 General Experimental Procedures S3
1.3 Catalyst Synthesis S5
1.4 Rotational Barrier Determination S6
1.5 Supplemental Optimization Table S30
1.6 Experimental Procedures
1.6.1 Starting Material Synthesis S35
1.6.2 Enantioselective *O*-alkylation S74
1.6.3 Derivatization of the *O*-alkylation product S87
1.7 References S88
1.8 NMR Spectra and HPLC Traces S89
1.9 Computational Methods S161

1.1 General Information

Reactions requiring moisture-sensitive reagents were carried out in flame-dried glassware, under an atmosphere of argon (balloon pressure). Dry dichloromethane and tetrahydrofuran were purified by filtration through activated alumina columns employing the method of Grubbs *et al*.[1] Tetrahyrofuran was further dried using activated 3 Å molecular sieves in accordance with procedures suggested by Williams *et al*.[2] Water was purified by an Elix® UV-10 system. Reagents were used directly as supplied by major chemical suppliers. Petrol refers to the fraction of petroleum ether which boils in the range 40–60 °C. Brine refers to a saturated aqueous solution of sodium chloride.

Silica gel chromatography was carried out using Merck Geduran® Silicagel (40–63 µm particle size). Thin layer chromatography (TLC) was carried out using pre-coated, aluminium backed plates (Merck Kieselgel 60 F254). Visualisation was achieved with ultraviolet irradiation (254 nm) and staining with permanganate.

NMR spectroscopy was carried out using Bruker Avance spectrometers in the deuterated solvent stated, using the residual non-deuterated solvent signal as an internal reference (^{1}H NMR: $CDCl_3$ (7.26), $(CD_3)_2SO$ (2.50); ^{13}C NMR: $CDCl_3$ (77.16), $(CD_3)_2SO$ (39.52); ^{19}F NMR: $CFCl_3$ (0.00)). Chemical shifts are quoted in ppm, based on appearance rather than interpretation. Signal patterns are indicated as: s, singlet; d, doublet; t, triplet; q, quartet, hept, heptet; m, multiplet. Coupling constants, *J*, are quoted to the nearest 0.1 Hz and are presented as observed. All ^{19}F NMR spectra are reported as proton/fluorine decoupled unless otherwise stated.

Infrared spectra were prepared as a neat film and were recorded using a Bruker Tensor 27 FTIR spectrometer using an ATR module.

HRMS was carried out using Bruker MicroTDF and Micromass GCT spectrometers under electrospray ionization (ESI) or ammonia chemical ionization (CI)/electron ionization (EI) conditions respectively.

Analytical chiral HPLC was carried out on a Dionex UltiMate 3000 HPLC system comprising a Dionex LPG-3400A pump, WPS-3000SL autosampler and TCC-3000SD column compartment, and a Daicel Chiralpak column (0.46 cm × 25 cm), equipped with an appropriate guard column (0.4 cm × 1 cm).

Melting points were determined using a Reichert melting point apparatus and are uncorrected.

Optical rotations were recorded on a Schmidt-Haensch Unipol L2000 polarimeter and values are quoted [° mL g^{-1} dm^{-1}]. Concentrations are quoted in g/100 mL.

1.2 General Experimental Procedures

General Procedure A: C-H Olefination of 2-Phenyl Acetic Acids

Following a modified literature procedure[3] *the appropriate 2-phenyl acetic acid* (1.0 equiv.), potassium bicarbonate (2.0 equiv.), (*S*)-*N*-acetylalanine (0.2 equiv.) and palladium acetate (0.1 equiv.) were added to a 500 mL round bottom flask in 50 mL *tert*-amyl alcohol. The flask was evacuated and back filled with oxygen three times, then ethyl acrylate (2.0 equiv.) was added. The solution was heated to 90 °C for 2 h after which it was cooled to room temperature and filtered through celite. 3 M HCl was added until the solution was at pH 1. The organic layer was extracted with ethyl acetate three times, dried with anhydrous sodium sulfate, filtered, and concentrated *in vacuo*. The crude mixture was purified by column chromatography (*see* experimental methods for specific details) to yield the corresponding acid.

General Procedure B: Amidation of 2-Phenyl Acetic Acids: Method 1 (with Oxalyl Chloride)

The appropriate acid (1.0 equiv.) was suspended in anhydrous dichloromethane. Oxalyl chloride (2.0 equiv.) and a catalytic amount of DMF were added and the reaction was stirred at room temperature for around 1 h until no more gas was produced. The solution was concentrated *in vacuo* and redissolved in anhydrous dichloromethane with the appropriate amine (2.0 equiv.) and stirred at room temperature for 2 h. The solution was diluted with dichloromethane and washed with water and brine. The organic phase was dried with anhydrous sodium sulfate, filtered, and concentrated *in vacuo*. The crude mixture was purified (*see* experimental methods for specific details) to yield the corresponding amide.

General Procedure C: Amidation of 2-Phenyl Acetic Acids: Method 2 (with Mukaiyama's Reagent)

The appropriate acid (1.0 equiv.) was dissolved in anhydrous dichloromethane and Mukaiyama's reagent (2-chloro-1-methylpyridinium iodide) (1.5 equiv.) was added. The reaction mixture was stirred under a nitrogen atmosphere for 30 min. Then, the appropriate amine (3.0 equiv.) was added to the mixture dropwise *via* syringe and stirred for 1 h at room temperature. The reaction was quenched with 1 M HCl (5 mL) and extracted with ethyl acetate three times. The combined organic layers were washed with brine, dried with anhydrous magnesium sulfate, and concentrated *in vacuo*. The crude mixture was purified by column chromatography (*see* experimental methods for specific details) to yield the corresponding amide.

General Procedure D: Hydrogenation of 3-Phenyl Acrylates

The appropriate acrylate (1.0 equiv.) was dissolved in methanol, with Pd/C (10% w/w). The flask was sealed and evacuated and back filled with hydrogen three times. The reaction was stirred under a hydrogen atmosphere overnight. The solution was filtered through celite and concentrated *in vacuo*. The crude was purified as required by column chromatography (*see* experimental methods for specific details) to yield the corresponding ester.

General Procedure E: Cyclisation of Esters and to Silyl Enol Ethers

The appropriate ester (1.0 equiv.) was dissolved in anhydrous THF and cooled to 0 °C under an argon atmosphere. KHMDS (0.5 M, 2.0 equiv.) was added dropwise *via* syringe pump. The solution was warmed to room temperature and stirred for 4 h. TBDMSCl (2.0 equiv.) was added and the solution stirred for 1 h. Saturated aqueous ammonium chloride was added and the organic layer extracted with diethyl ether. The organic layer was dried with anhydrous sodium sulfate, filtered, and

concentrated *in vacuo*. The crude mixture was purified by column chromatography (*see* experimental methods for specific details) to yield the appropriate silyl enol ether.

General Procedure F: Oxidation of Silyl Enol Ethers: Method 1 (with DDQ)

The appropriate silyl enol ether (1.0 equiv.) was dissolved in toluene with DDQ (1.3 equiv.) and heated at reflux for 10–40 min. The solution was cooled to room temperature, filtered through celite (washing with diethyl ether) and concentrated *in vacuo*. The crude mixture was purified by column chromatography (*see* experimental methods for specific details) to yield the appropriate silylated naphthol.

General Procedure G: Oxidation of Silyl Enol Ethers: Method 2 (with NBS)

The appropriate silyl enol ether (1.0 equiv.) was dissolved in benzene. NBS (1.2 equiv.) was added and the solution was heated at reflux overnight. The reaction was cooled to room temperature and the solution concentrated *in vacuo*. The crude was purified by column chromatography (*see* experimental methods for specific details). If necessary, the resultant solid was triturated with hexane and a small amount of dichloromethane and filtered to yield the corresponding naphthol.

General Procedure H: Deprotection of Silylated Naphthols

The appropriate silylated naphthol (1.0 equiv.) was dissolved in methanol. Potassium fluoride (2.0 equiv.) was added and the reaction was stirred at room temperature for 30 min. Saturated aqueous ammonium chloride was added to the solution and the organic layer was extracted with ethyl acetate, dried with anhydrous sodium sulfate, filtered, and concentrated *in vacuo*. The crude was purified, as required, by column chromatography or trituration with ethyl acetate (see experimental method for details) to yield the corresponding naphthol.

General Procedure I: Suzuki Reaction

The appropriate aryl bromide (1.0 equiv.), was suspended in water in a glass vial with the appropriate boronic acid (1.1 equiv.), palladium acetate (0.05 equiv.), TBAB (1.0 equiv.), and sodium carbonate (3.0 equiv.) under nitrogen. The reaction mixture was stirred and heated to reflux until the aryl bromide was fully consumed (15–25 min). The reaction was cooled to room temperature and the organic layer was extracted with ethyl acetate three times. The combined organic layers were then dried with anhydrous magnesium sulfate, filtered and concentrated *in vacuo*. The resulting crude material was purified by column chromatography (*see* experimental methods for specific details) to yield the corresponding biaryl.

General Procedure J: Photocyclisation of Acrylates

The appropriate amide was dissolved in anhydrous THF in a flame-dried vial under a nitrogen atmosphere (with 0.01 mol% $Ir(fppy)_3$, if required). The solution was cooled to 0 °C and LiHMDS in THF was added. The reaction mixture was allowed to stir while irradiating with a 12 W blue lamp (λ = 450 nm). After 1 h, the reaction was quenched with saturated aqueous ammonium chloride, then extracted with ethyl acetate three times. The combined organic phases were dried over anhydrous magnesium sulfate and concentrated *in vacuo*. The crude mixture was purified by column chromatography (*see* experimental methods for specific details) to yield the corresponding naphthol.

General Procedure K: Racemic *O*-Alkylation of Naphthols

The appropriate naphthol was suspended in toluene and benzyl bromide (3.0 equiv.) and TBAB (0.1 equiv.) were added. The mixture was vigorously stirred before 50% aqueous potassium hydroxide (5.0 equiv.) or 50% caesium carbonate (5.0 equiv.) was added. After 16 h, the reaction was quenched with saturated aqueous ammonium chloride. The organic phase was extracted with ethyl acetate, dried with anhydrous sodium sulfate, filtered, and concentrated *in vacuo*. The crude mixture was purified by column chromatography or small scale TLC depending on scale (*see* experimental methods for details) to yield the corresponding benzylated naphthol.

General Procedure L: Asymmetric *O*-Alkylation of Naphthols

The appropriate naphthol was suspended in benzene (0.01 M) and benzyl iodide (3.0 equiv.), catalyst **11** (0.05 equiv.) were added. The mixture was vigorously stirred before 50% aqueous caesium carbonate (5.0 equiv.) was added. After 48 h of vigorous stirring, the reaction was quenched with saturated aqueous ammonium chloride and extracted with ethyl acetate. The organic phase was dried with anhydrous sodium sulfate, filtered and concentrated *in vacuo* at room temperature. The crude mixture was purified by column chromatography (*see* experimental methods for details) to yield the corresponding benzylated naphthol.

1.3 Catalyst Synthesis

Catalysts that were not commercially available were prepared following well established literature procedures of *N*-alkylation of cinchona alkaloids.[4]

1.4 Rotational Barrier Determination

Barriers to rotation of benzylated naphthols were measured in *m*-xylene (10 mg/mL) and heated to the temperature stated by an oil bath. Samples were removed periodically and diluted with 50% isopropanol in hexane at room temperature. Barriers of naphthols were measured in solvent stated (concentration unknown) at room temperature (298 K) and samples were directly injected into HPLC.

Table S1 Barrier to Rotation of **1**

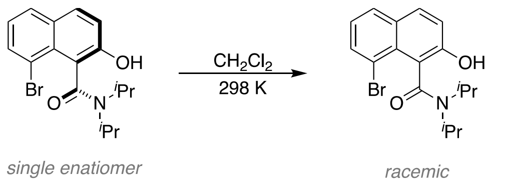

time (h)	time (s)	major ent. (%)	minor ent. (%)	e.e. (%)	ln(100/e.e.)
0.00	0	99.46	0.54	98.92	0.01
0.28	1020	94.96	5.04	89.92	0.11
0.55	1980	90.82	9.18	81.64	0.20
0.82	2940	87.15	12.85	74.30	0.30
1.08	3900	83.54	16.46	67.08	0.40
1.37	4920	80.56	19.44	61.12	0.49
1.63	5880	77.62	22.38	55.24	0.59
1.90	6840	75.07	24.93	50.14	0.69
2.18	7860	72.73	27.27	45.46	0.79
2.45	8820	70.69	29.31	41.38	0.88
2.72	9780	68.74	31.26	37.48	0.98
3.00	10800	67.00	33.00	34.00	1.08
3.27	11760	65.40	34.60	30.80	1.18
3.53	12720	64.05	35.95	28.10	1.27
3.82	13740	62.68	37.32	25.36	1.37
4.08	14700	61.57	38.43	23.14	1.46
4.35	15660	60.55	39.45	21.10	1.56
4.62	16620	59.44	40.56	18.88	1.67
4.90	17640	58.71	41.29	17.42	1.75

5.17	18600	57.76	42.24	15.52	1.86
5.43	19560	57.02	42.98	14.04	1.96
5.72	20580	56.38	43.62	12.76	2.06
5.98	21540	55.78	44.22	11.56	2.16
6.25	22500	55.19	44.81	10.38	2.27
6.53	23520	54.68	45.32	9.36	2.37
6.80	24480	54.42	45.58	8.84	2.43
7.07	25440	53.81	46.19	7.62	2.57
7.33	26400	53.44	46.56	6.88	2.68
7.62	27420	53.13	46.87	6.26	2.77
7.88	28380	52.76	47.24	5.52	2.90

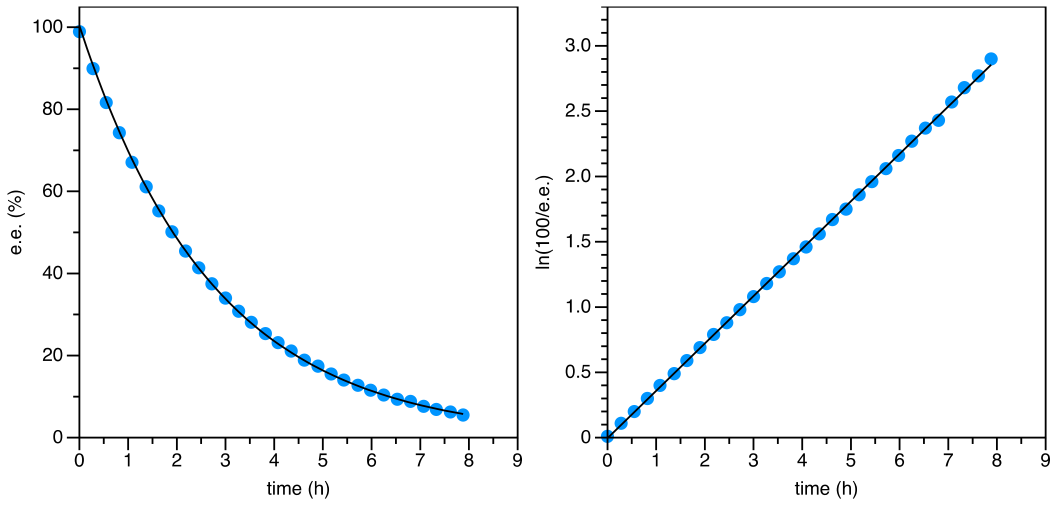

$$\ln\left(\frac{100}{\text{e.e.}}\right) = k_{rac} \times t + \ln\left(\frac{100}{e.e._{t=0}}\right)$$

$$k_{ent} = \frac{1}{2}k_{rac}$$

$$k_{ent} = \frac{1}{2}slope = 5.04 \times 10^{-5}$$

$$\Delta G_T^{\ddagger} = \ln\left(\frac{k_B T}{h \times k_{ent}}\right)RT = 97.5 \text{ kJ mol}^{-1}$$

$$t_{\frac{1}{2}}(rac) = \frac{\ln 2}{k_{rac}} = 1.9 \text{ h}$$

Table S2 Barrier to Rotation of **2**

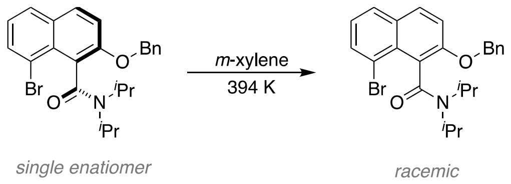

time (h)	time (s)	major ent. (%)	minor ent. (%)	e.e. (%)	ln(100/e.e.)
0.00	0.00	100.00	0.00	100.00	0.00
0.25	900.00	94.98	5.02	89.96	0.11
0.50	1800.00	90.53	9.47	81.06	0.21
1.00	3600.00	82.71	17.29	65.42	0.42
1.53	5520.00	76.30	23.70	52.60	0.64
2.03	7320.00	71.39	28.61	42.78	0.85
2.50	9000.00	67.35	32.65	34.70	1.06
3.00	10800.00	64.56	35.44	29.12	1.23
3.50	12600.00	61.81	38.19	23.62	1.44
4.00	14400.00	59.65	40.35	19.30	1.65
4.52	16260.00	57.84	42.16	15.68	1.85
5.00	18000.00	56.44	43.56	12.88	2.05
5.50	19800.00	55.26	44.74	10.52	2.25

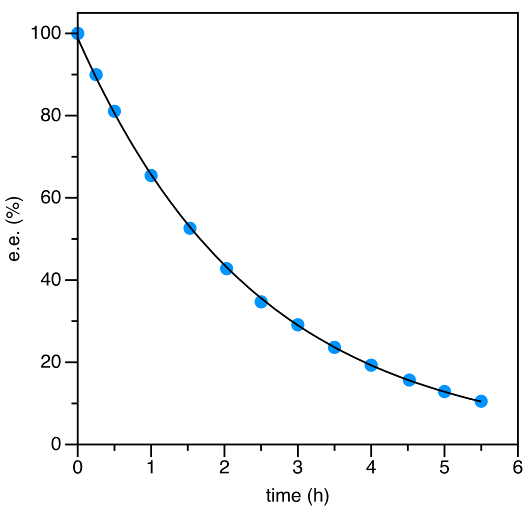

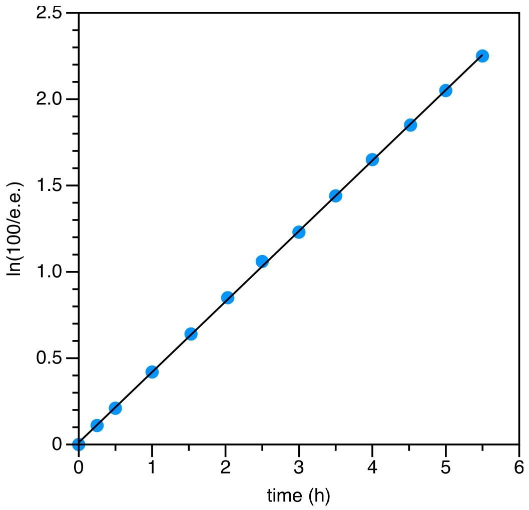

$$\ln\left(\frac{100}{\text{e.e.}}\right) = k_{rac} \times t + \ln\left(\frac{100}{e.e._{t=0}}\right)$$

$$k_{ent} = \frac{1}{2} k_{rac}$$

$$k_{ent} = \frac{1}{2} slope = 5.68 \times 10^{-5}$$

$$\Delta G_T^{\ddagger} = \ln\left(\frac{k_B T}{h \times k_{ent}}\right) RT = 129.3\ \text{kJ mol}^{-1}$$

$$t_{\frac{1}{2}}(rac) = \frac{\ln 2}{k_{rac}} = 102\ \text{min}$$

Table S3 Barrier to Rotation of **6**

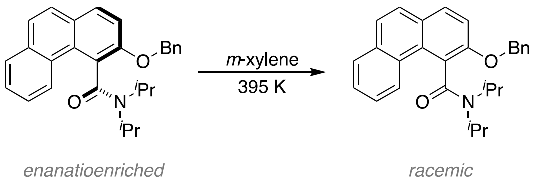

time (h)	time (s)	major ent. (%)	minor ent. (%)	e.e. (%)	ln(100/e.e.)
0.00	0	100.00	0.00	100.00	0
0.18	660	98.25	1.75	96.50	0.04
0.33	1200	96.51	3.49	93.02	0.07
0.50	1800	94.69	5.31	89.38	0.11
0.75	2700	92.51	7.49	85.02	0.16
1.00	3600	89.73	10.27	79.46	0.23
1.25	4500	87.30	12.70	74.60	0.29
1.50	5400	85.02	14.98	70.04	0.36
2.02	7260	81.07	18.93	62.14	0.48
2.62	9420	76.66	23.34	53.32	0.63
3.25	11700	72.70	27.30	45.40	0.79
3.83	13800	69.66	30.34	39.32	0.93
4.58	16500	66.25	33.75	32.50	1.12
5.38	19380	63.47	36.53	26.94	1.31
6.30	22680	60.96	39.04	21.92	1.52
6.82	24540	59.77	40.23	19.54	1.63

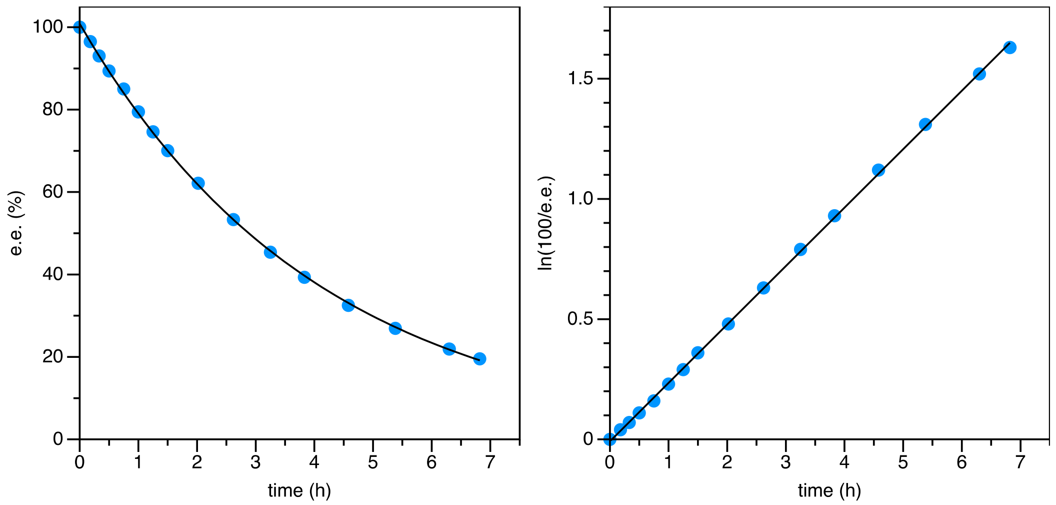

$$\ln\left(\frac{100}{\text{e.e.}}\right) = k_{rac} \times t + \ln\left(\frac{100}{e.e._{t=0}}\right)$$

$$k_{ent} = \frac{1}{2}k_{rac}$$

$$k_{ent} = \frac{1}{2}slope = 3.38 \times 10^{-5}$$

$$\Delta G_T^{\ddagger} = \ln\left(\frac{k_B T}{h \times k_{ent}}\right)RT = 131.4 \text{ kJ mol}^{-1}$$

$$t_{\frac{1}{2}}(rac) = \frac{\ln 2}{k_{rac}} = 2.8 \text{ h}$$

Table S4 Barrier to Rotation of **12**

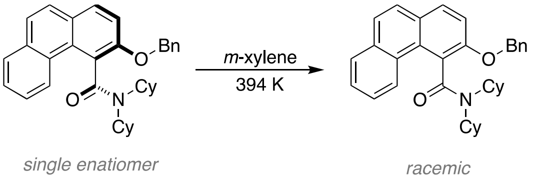

time (h)	time (s)	major ent. (%)	minor ent. (%)	e.e. (%)	ln(100/e.e.)
0.00	0	97.55	2.45	95.10	0.05
0.17	600	96.80	3.20	93.60	0.07
0.25	900	96.29	3.71	92.58	0.08
0.35	1260	95.80	4.20	91.60	0.09
0.42	1500	95.27	4.73	90.54	0.10
0.50	1800	94.96	5.04	89.92	0.11
0.67	2400	93.98	6.02	87.96	0.13
0.83	3000	93.08	6.92	86.16	0.15
1.00	3600	92.09	7.91	84.18	0.17
1.25	4500	90.83	9.17	81.66	0.20
1.50	5400	89.47	10.53	78.94	0.24
1.78	6420	88.05	11.95	76.10	0.27
2.00	7200	86.80	13.20	73.60	0.31
2.33	8400	85.15	14.85	70.30	0.35
2.70	9720	83.69	16.31	67.38	0.39
3.00	10800	82.41	17.59	64.82	0.43
3.50	12600	80.06	19.94	60.12	0.51
4.00	14400	77.90	22.10	55.80	0.58
4.50	16200	76.08	23.92	52.16	0.65
5.00	18000	74.87	25.13	49.74	0.70

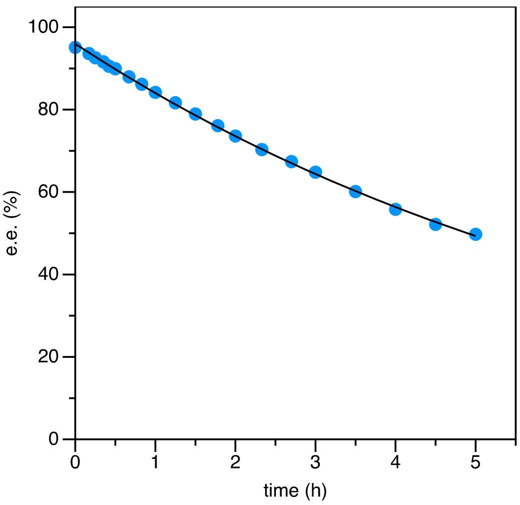

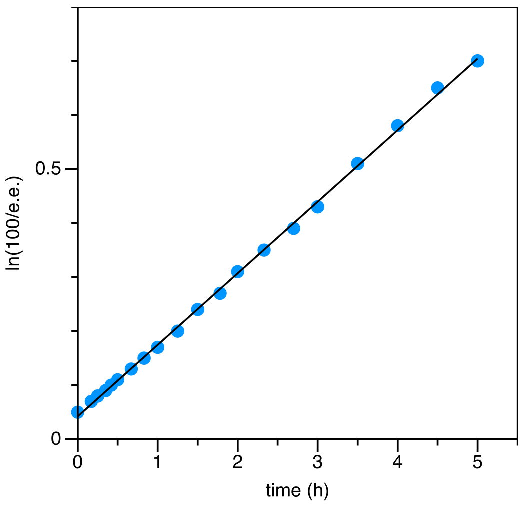

$$\ln\left(\frac{100}{\text{e.e.}}\right) = k_{rac} \times t + \ln\left(\frac{100}{e.e._{t=0}}\right)$$

$$k_{ent} = \frac{1}{2} k_{rac}$$

$$k_{ent} = \frac{1}{2} slope = 3.85 \times 10^{-5}$$

$$\Delta G_T^{\ddagger} = \ln\left(\frac{k_B T}{h \times k_{ent}}\right) RT = 133.1 \text{ kJ mol}^{-1}$$

$$t_{\frac{1}{2}}(rac) = \frac{\ln 2}{k_{rac}} = 5.2 \text{ h}$$

Table S5 Barrier to Rotation of **13**

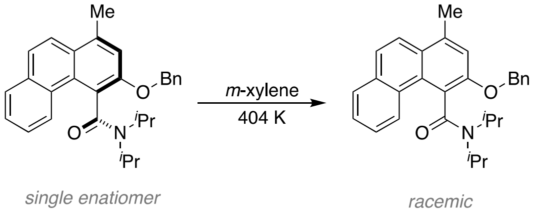

time (h)	time (s)	major ent. (%)	minor ent. (%)	e.e. (%)	ln(100/e.e.)
0.00	0	97.97	2.03	95.94	0.04
0.08	300	94.59	5.41	89.18	0.11
0.17	600	91.70	8.30	83.40	0.18
0.25	900	87.97	12.03	75.94	0.28
0.33	1200	84.93	15.07	69.86	0.36
0.42	1500	82.14	17.86	64.28	0.44
0.50	1800	79.67	20.33	59.34	0.52
0.67	2400	75.32	24.68	50.64	0.68
0.83	3000	71.48	28.52	42.96	0.84
1.00	3600	68.32	31.68	36.64	1.00
1.25	4500	64.49	35.51	28.98	1.24
1.52	5460	61.36	38.64	22.72	1.48
1.75	6300	59.21	40.79	18.42	1.69
2.00	7200	57.44	42.56	14.88	1.91
2.50	9000	54.89	45.11	9.78	2.32

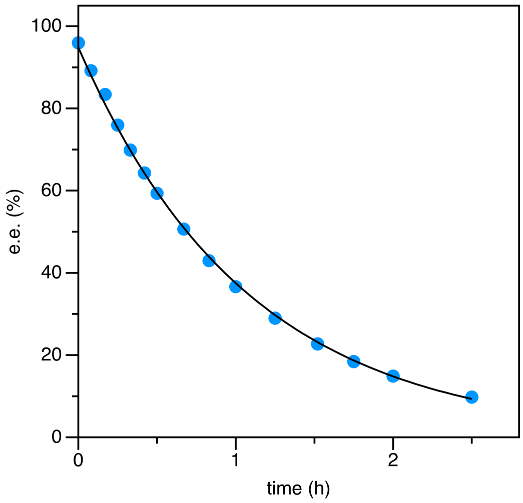

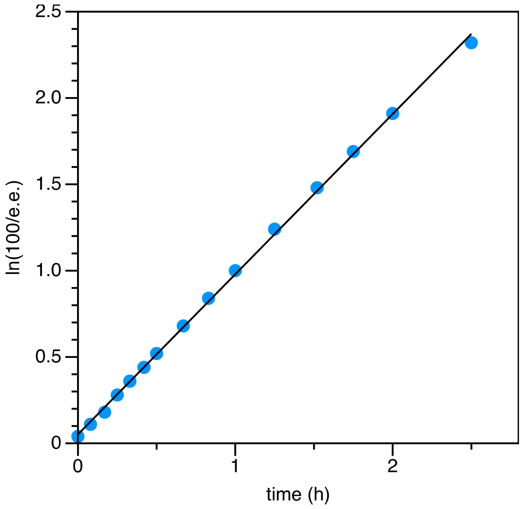

$$\ln\left(\frac{100}{\text{e.e.}}\right) = k_{rac} \times t + \ln\left(\frac{100}{e.e._{t=0}}\right)$$

$$k_{ent} = \frac{1}{2} k_{rac}$$

$$k_{ent} = \frac{1}{2} slope = 1.29 \times 10^{-4}$$

$$\Delta G_T^{\ddagger} = \ln\left(\frac{k_B T}{h \times k_{ent}}\right) RT = 130.1 \text{ kJ mol}^{-1}$$

$$t_{\frac{1}{2}}(rac) = \frac{\ln 2}{k_{rac}} = 45 \text{ min}$$

Table S6 Barrier to Rotation of **14**

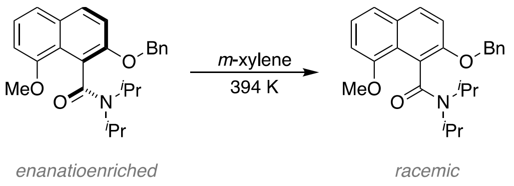

time (h)	time (s)	major ent. (%)	minor ent. (%)	e.e. (%)	ln(100/e.e.)
0.00	0	96.06	3.94	92.12	0.08
0.17	600	95.77	4.23	91.54	0.09
0.33	1200	95.40	4.60	90.80	0.10
0.50	1800	94.91	5.09	89.82	0.11
0.75	2700	94.22	5.78	88.44	0.12
1.00	3600	93.62	6.38	87.24	0.14
1.33	4800	92.68	7.32	85.36	0.16
1.67	6000	91.88	8.12	83.76	0.18
2.33	8400	90.24	9.76	80.48	0.22
3.00	10800	88.78	11.22	77.56	0.25
4.00	14400	86.66	13.34	73.32	0.31
5.00	18000	84.60	15.40	69.20	0.37
6.00	21600	82.40	17.60	64.80	0.43
7.00	25200	80.91	19.09	61.82	0.48
8.00	28800	79.23	20.77	58.46	0.54
9.00	32400	77.69	22.31	55.38	0.59
10.00	36000	75.96	24.04	51.92	0.66
11.00	39600	74.81	25.19	49.62	0.70
12.00	43200	73.46	26.54	46.92	0.76

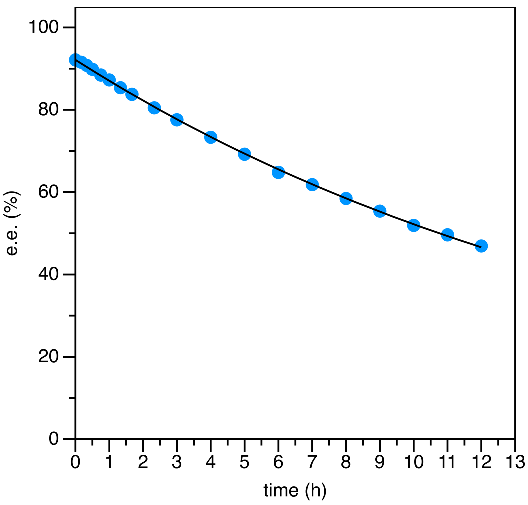

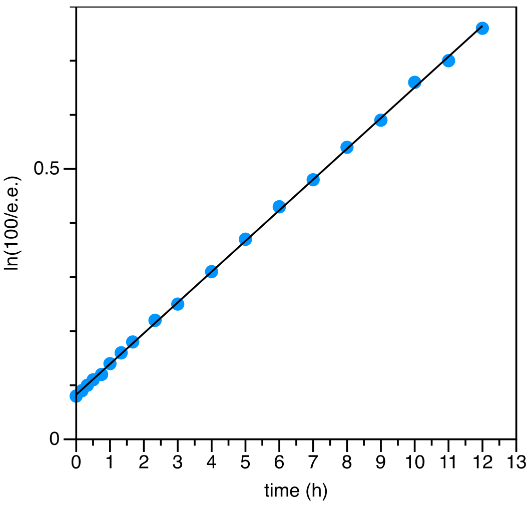

$$\ln\left(\frac{100}{\text{e.e.}}\right) = k_{rac} \times t + \ln\left(\frac{100}{e.e._{t=0}}\right)$$

$$k_{ent} = \frac{1}{2}k_{rac}$$

$$k_{ent} = \frac{1}{2}slope = 7.89 \times 10^{-6}$$

$$\Delta G_T^{\ddagger} = \ln\left(\frac{k_B T}{h \times k_{ent}}\right) RT = 136.0 \text{ kJ mol}^{-1}$$

$$t_{\frac{1}{2}}(rac) = \frac{\ln 2}{k_{rac}} = 12.2 \text{ h}$$

Table S7 Barrier to Rotation of **15**

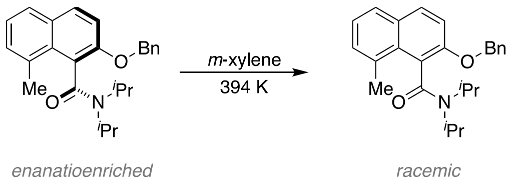

time (h)	time (s)	major ent. (%)	minor ent. (%)	e.e. (%)	ln(100/e.e.)
0.00	0	97.97	2.03	95.94	0.04
0.08	300	95.58	4.42	91.16	0.09
0.17	600	93.31	6.69	86.62	0.14
0.25	900	91.05	8.95	82.10	0.20
0.33	1200	88.99	11.01	77.98	0.25
0.42	1500	87.00	13.00	74.00	0.30
0.52	1860	84.54	15.46	69.08	0.37
0.67	2400	81.28	18.72	62.56	0.47
0.83	3000	78.15	21.85	56.30	0.57
1.25	4500	71.55	28.45	43.10	0.84
1.50	5400	68.23	31.77	36.46	1.01
1.75	6300	65.67	34.33	31.34	1.16
2.00	7200	63.37	36.63	26.74	1.32
2.25	8100	61.42	38.58	22.84	1.48
2.50	9000	59.76	40.24	19.52	1.63
2.75	9900	58.42	41.58	16.84	1.78
3.00	10800	57.19	42.81	14.38	1.94
3.25	11700	56.27	43.73	12.54	2.08

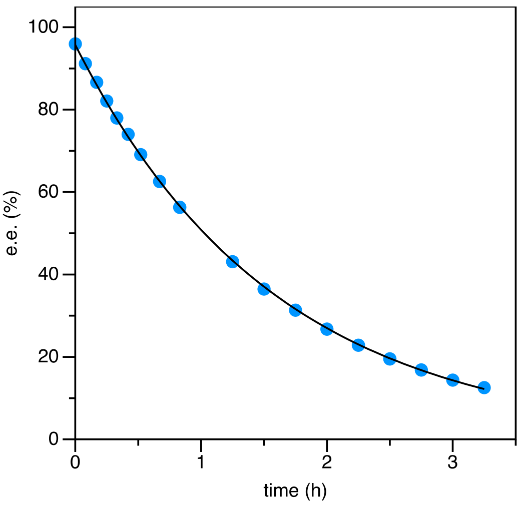

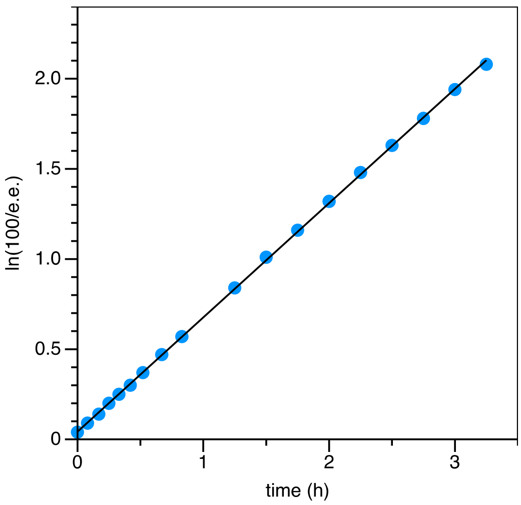

$$\ln\left(\frac{100}{\text{e.e.}}\right) = k_{rac} \times t + \ln\left(\frac{100}{e.e._{t=0}}\right)$$

$$k_{ent} = \frac{1}{2}k_{rac}$$

$$k_{ent} = \frac{1}{2}slope = 1.76 \times 10^{-4}$$

$$\Delta G_T^{\ddagger} = \ln\left(\frac{k_B T}{h \times k_{ent}}\right) RT = 128.0\ \text{kJ mol}^{-1}$$

$$t_{\frac{1}{2}}(rac) = \frac{\ln 2}{k_{rac}} = 66\ \text{min}$$

Table S8 Barrier to Rotation of **16**

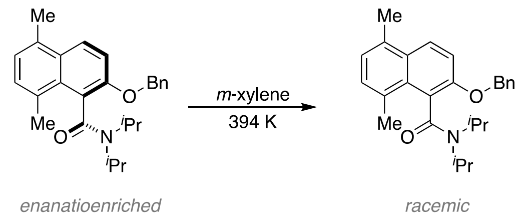

time (h)	time (s)	major ent. (%)	minor ent. (%)	e.e. (%)	ln(100/e.e.)
0.00	0	97.97	2.03	95.94	0.04
0.08	300	97.14	2.86	94.28	0.06
0.17	600	95.48	4.52	90.96	0.09
0.25	900	93.92	6.08	87.84	0.13
0.33	1200	92.18	7.82	84.36	0.17
0.42	1500	90.56	9.44	81.12	0.21
0.50	1800	89.15	10.85	78.30	0.24
0.67	2400	86.31	13.69	72.62	0.32
0.85	3060	83.42	16.58	66.84	0.40
1.00	3600	81.45	18.55	62.90	0.46
1.25	4500	77.97	22.03	55.94	0.58
1.53	5520	74.54	25.46	49.08	0.71
1.75	6300	72.41	27.59	44.82	0.80
2.08	7500	69.33	30.67	38.66	0.95
2.35	8460	67.26	32.74	34.52	1.06
2.67	9600	65.14	34.86	30.28	1.19
3.00	10800	62.91	37.09	25.82	1.35
3.33	12000	61.01	38.99	22.02	1.51
3.67	13200	59.55	40.45	19.10	1.66
4.00	14400	58.28	41.72	16.56	1.80
4.50	16200	56.68	43.32	13.36	2.01

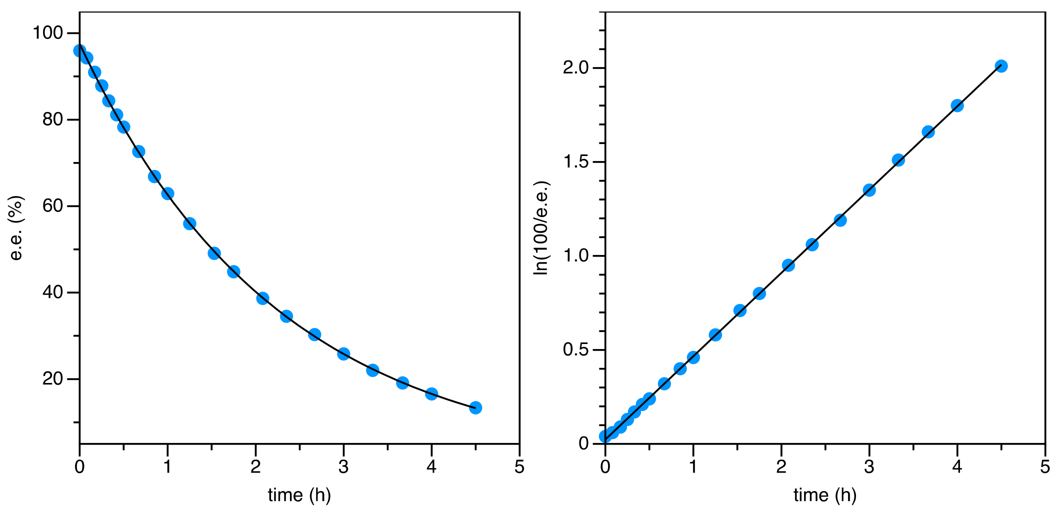

$$\ln\left(\frac{100}{\text{e.e.}}\right) = k_{rac} \times t + \ln\left(\frac{100}{e.e._{t=0}}\right)$$

$$k_{ent} = \frac{1}{2} k_{rac}$$

$$k_{ent} = \frac{1}{2} slope = 6.16 \times 10^{-5}$$

$$\Delta G_T^{\ddagger} = \ln\left(\frac{k_B T}{h \times k_{ent}}\right) RT = 129.3 \text{ kJ mol}^{-1}$$

$$t_{\frac{1}{2}}(rac) = \frac{\ln 2}{k_{rac}} = 94 \text{ min}$$

Table S9 Barrier to Rotation of **17**

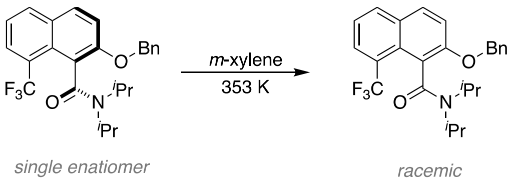

time (h)	time (s)	major ent. (%)	minor ent. (%)	e.e. (%)	ln(100/e.e.)
0.00	0	99.80	0.00	99.80	0.00
0.08	300	96.67	3.33	93.34	0.07
0.17	600	93.17	6.83	86.34	0.15
0.25	900	89.93	10.07	79.86	0.22
0.33	1200	86.85	13.15	73.70	0.31
0.50	1800	81.55	18.45	63.10	0.46
0.67	2400	76.95	23.05	53.90	0.62
0.83	3000	72.03	27.97	44.06	0.82
1.00	3600	69.67	30.33	39.34	0.93
1.28	4620	65.10	34.90	30.20	1.20
1.50	5400	62.25	37.75	24.50	1.41
1.75	6300	59.70	40.30	19.40	1.64
2.00	7200	57.68	42.32	15.36	1.87
2.40	8640	55.27	44.73	10.54	2.25
2.67	9600	54.11	45.89	8.22	2.50
3.07	11040	52.83	47.17	5.66	2.87
3.65	13140	51.62	48.38	3.24	3.43
4.27	15360	50.90	49.10	1.80	4.02
4.75	17100	50.58	49.42	1.16	4.46

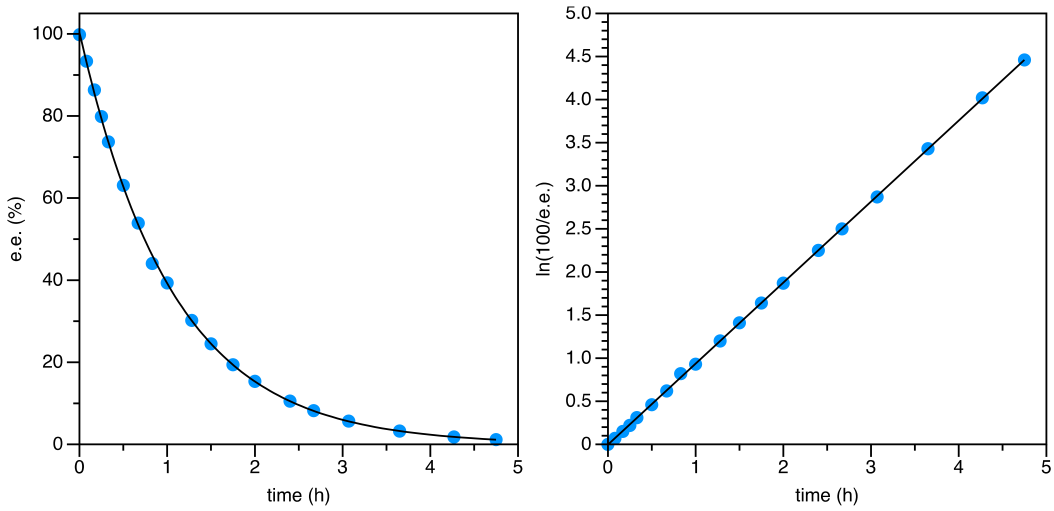

$$\ln\left(\frac{100}{\text{e.e.}}\right) = k_{rac} \times t + \ln\left(\frac{100}{e.e._{t=0}}\right)$$

$$k_{ent} = \frac{1}{2} k_{rac}$$

$$k_{ent} = \frac{1}{2} slope = 1.31 \times 10^{-4}$$

$$\Delta G_T^{\ddagger} = \ln\left(\frac{k_B T}{h \times k_{ent}}\right) RT = 113.4 \text{ kJ mol}^{-1}$$

$$t_{\frac{1}{2}}(rac) = \frac{\ln 2}{k_{rac}} = 44 \text{ min}$$

Table S10 Barrier to Rotation of **18**

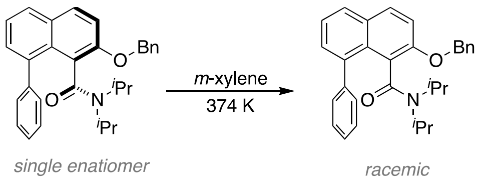

time (h)	time (s)	major ent. (%)	minor ent. (%)	e.e. (%)	ln(100/e.e.)
0.00	0	95.14	4.86	90.28	0.10
0.08	300	89.26	10.74	78.52	0.24
0.17	600	83.37	16.63	66.74	0.40
0.25	900	78.99	21.01	57.98	0.55
0.33	1200	74.54	25.46	49.08	0.71
0.42	1500	71.09	28.91	42.18	0.86
0.50	1800	68.22	31.78	36.44	1.01
0.58	2100	65.79	34.21	31.58	1.15
0.67	2400	63.62	36.38	27.24	1.30
0.75	2700	61.99	38.01	23.98	1.43
0.83	3000	60.25	39.75	20.50	1.58
0.92	3300	58.45	41.55	16.90	1.78
1.00	3600	57.67	42.33	15.34	1.87
1.08	3900	56.21	43.79	12.42	2.09
1.17	4200	55.26	44.74	10.52	2.25
1.33	4800	53.82	46.18	7.64	2.57
1.50	5400	52.75	47.25	5.50	2.90

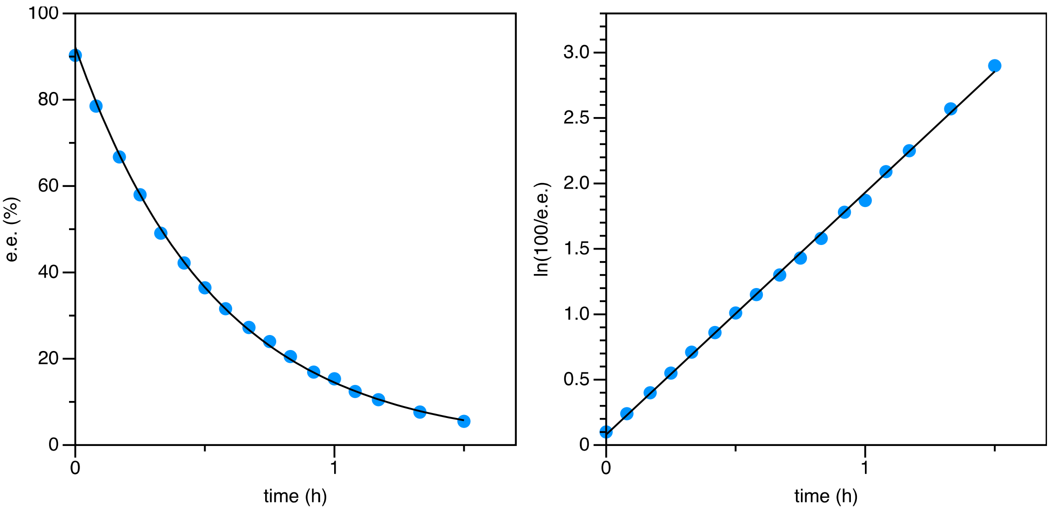

$$\ln\left(\frac{100}{\text{e.e.}}\right) = k_{rac} \times t + \ln\left(\frac{100}{e.e._{t=0}}\right)$$

$$k_{ent} = \frac{1}{2}k_{rac}$$

$$k_{ent} = \frac{1}{2}slope = 2.57 \times 10^{-4}$$

$$\Delta G_T^{\ddagger} = \ln\left(\frac{k_B T}{h \times k_{ent}}\right) RT = 118.0 \text{ kJ mol}^{-1}$$

$$t_{\frac{1}{2}}(rac) = \frac{\ln 2}{k_{rac}} = 22 \text{ min}$$

Table S11 Barrier to Rotation of **19**

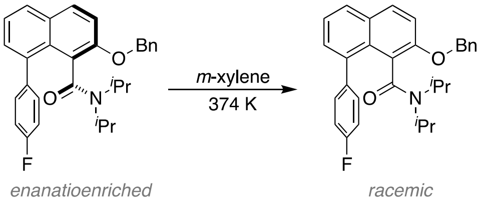

time (h)	time (s)	major ent. (%)	minor ent. (%)	e.e. (%)	ln(100/e.e.)
0.00	0	97.88	2.12	95.76	0.04
0.08	300	93.52	6.48	87.04	0.14
0.17	600	88.96	11.04	77.92	0.25
0.25	900	84.12	15.88	68.24	0.38
0.33	1200	80.30	19.70	60.60	0.50
0.43	1560	76.07	23.93	52.14	0.65
0.50	1800	73.85	26.15	47.70	0.74
0.58	2100	71.18	28.82	42.36	0.86
0.67	2400	68.98	31.02	37.96	0.97
0.75	2700	66.90	33.11	33.79	1.09
0.83	3000	65.08	34.92	30.16	1.20
0.92	3300	63.60	36.40	27.20	1.30
1.00	3600	62.09	37.91	24.18	1.42
1.08	3900	60.86	39.14	21.72	1.53
1.17	4200	59.68	40.32	19.36	1.64
1.33	4800	57.76	42.24	15.52	1.86
1.50	5400	56.26	43.74	12.52	2.08

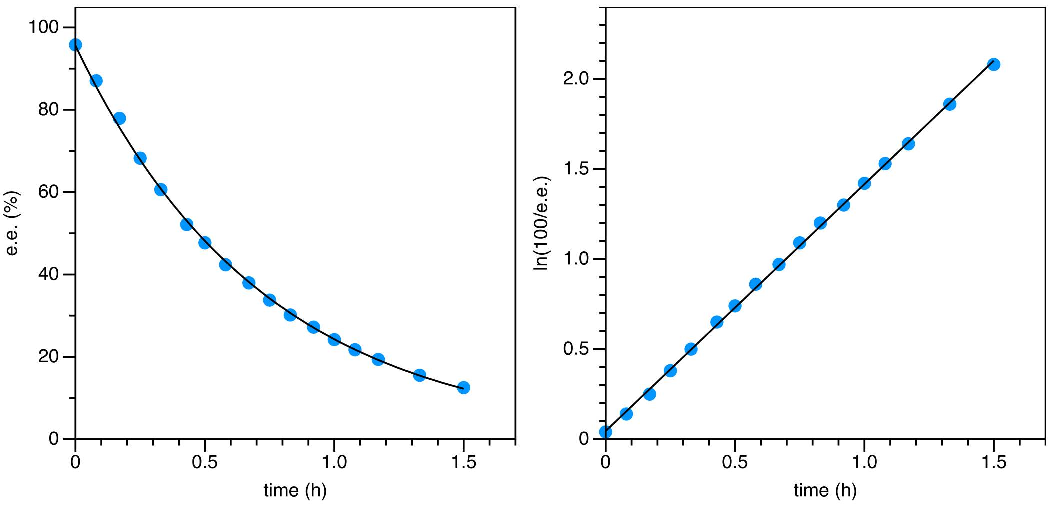

$$\ln\left(\frac{100}{\text{e.e.}}\right) = k_{rac} \times t + \ln\left(\frac{100}{e.e._{t=0}}\right)$$

$$k_{ent} = \frac{1}{2}k_{rac}$$

$$k_{ent} = \frac{1}{2}slope = 1.90 \times 10^{-4}$$

$$\Delta G_T^{\ddagger} = \ln\left(\frac{k_B T}{h \times k_{ent}}\right)RT = 118.8 \text{ kJ mol}^{-1}$$

$$t_{\frac{1}{2}}(rac) = \frac{\ln 2}{k_{rac}} = 30 \text{ min}$$

Table S12 Barrier to Rotation of **20**

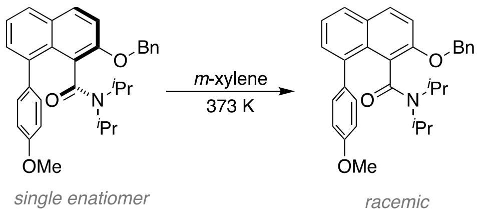

time (h)	time (s)	major ent. (%)	minor ent. (%)	e.e. (%)	ln(100/e.e.)
0.00	0	96.97	3.03	93.94	0.06
0.08	300	93.66	6.34	87.32	0.14
0.17	600	90.36	9.64	80.72	0.21
0.25	900	87.60	12.40	75.20	0.29
0.35	1260	84.58	15.42	69.16	0.37
0.42	1500	82.57	17.43	65.14	0.43
0.50	1800	80.46	19.54	60.92	0.50
0.58	2100	78.30	21.70	56.60	0.57
0.67	2400	76.34	23.66	52.68	0.64
0.83	3000	72.69	27.31	45.38	0.79
1.00	3600	69.46	30.54	38.92	0.94
1.25	4500	65.39	34.61	30.78	1.18
1.52	5460	61.97	38.03	23.94	1.43
1.75	6300	59.65	40.35	19.30	1.65
2.00	7200	57.65	42.35	15.30	1.88
2.25	8100	56.11	43.89	12.22	2.10
2.77	9960	53.92	46.08	7.84	2.55

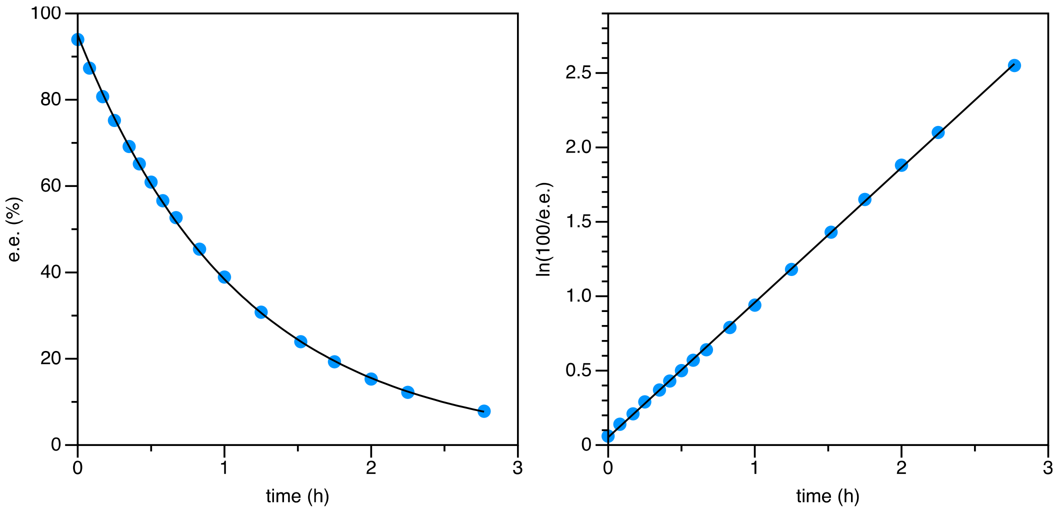

$$\ln\left(\frac{100}{\text{e.e.}}\right) = k_{rac} \times t + \ln\left(\frac{100}{e.e._{t=0}}\right)$$

$$k_{ent} = \frac{1}{2} k_{rac}$$

$$k_{ent} = \frac{1}{2} slope = 1.26 \times 10^{-4}$$

$$\Delta G_T^{\ddagger} = \ln\left(\frac{k_B T}{h \times k_{ent}}\right) RT = 120.1 \text{ kJ mol}^{-1}$$

$$t_{\frac{1}{2}}(rac) = \frac{\ln 2}{k_{rac}} = 46 \text{ min}$$

1.5 Supplemental Optimisation Table

Chiral phase transfer catalysts

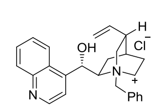

CN 1

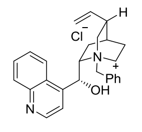

CD 1

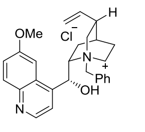

QN 1

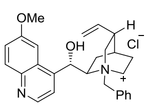

7

QD 1

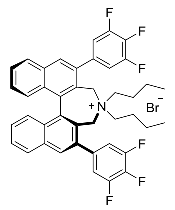

M4

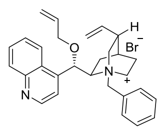

CN12

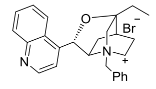

CN4

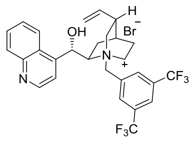

CN14

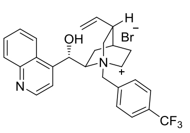

CN2

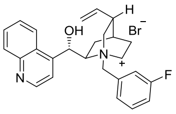

CN3

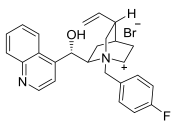

CN7

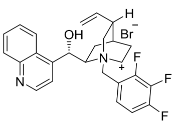

CN10

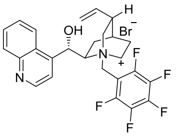

CN9

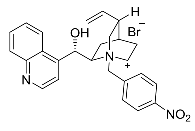

CN8

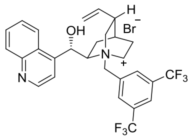

CN6

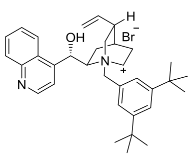

CN15

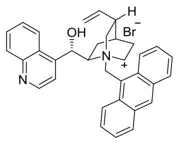

8

CN11

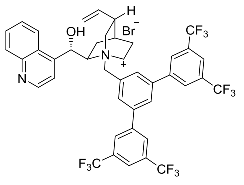

CN13

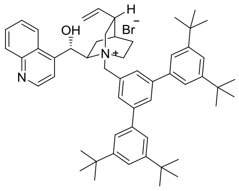

11

CN16

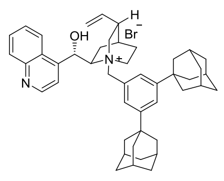

CN25

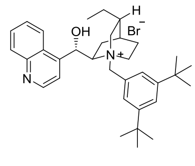

CN24

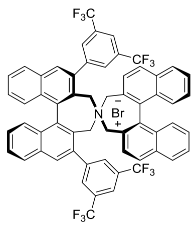

M12

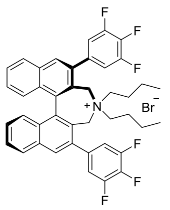

M13

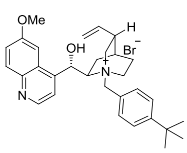

QD 3

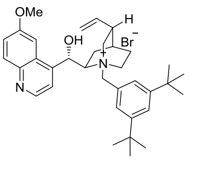

10

QD4

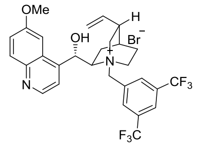

9

QD5

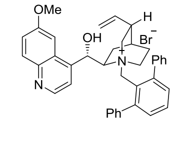

QD6

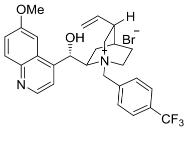

QD8

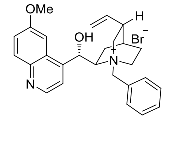

QD11

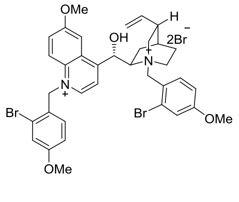

QD12

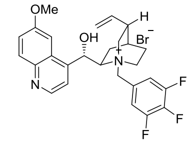

QD14

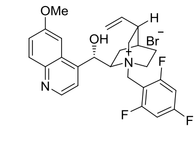

QD15

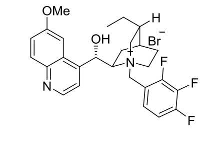

QD17

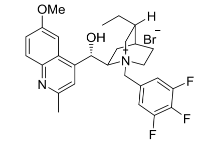

QD18

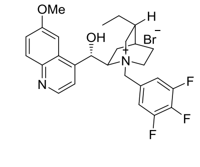

QD19

Table S13 Optimisation of asymmetric *O*-alkylation of naphthol **5**.

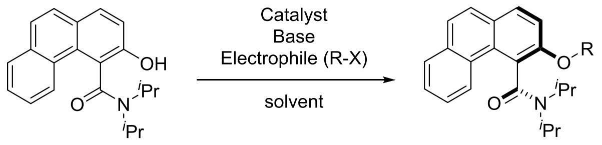

5 **6**

Catalyst	Catalyst Eq	Base	Base (equiv.)	Electrophile	Electrophile (equiv.)	Solvent	Concentration (M)[a]	Temperature	e.r.[b]	Conversion (%)[c]
CN1	0.05	50% KOH (aq)	2.5	BnBr	3	Toluene	0.1	rt	50:50	N/A
CD1	0.05	50% KOH (aq)	2.5	BnBr	3	Toluene	0.1	rt	50:50	N/A
QN1	0.05	50% KOH (aq)	2.5	BnBr	3	Toluene	0.1	rt	50:50	N/A
QD1	0.05	50% KOH (aq)	2.5	BnBr	3	Toluene	0.1	rt	50:50	N/A
CN1	0.05	50% K_2CO_3 (aq)	2.5	BnBr	3	Toluene	0.1	rt	75:25	N/A
CD1	0.05	50% K_2CO_3 (aq)	2.5	BnBr	3	Toluene	0.1	rt	25:75	N/A
QN1	0.05	50% K_2CO_3 (aq)	2.5	BnBr	3	Toluene	0.1	rt	36:64	N/A
QD1	0.05	50% K_2CO_3 (aq)	2.5	BnBr	3	Toluene	0.1	rt	64:36	N/A
CN6	0.05	50% K_2CO_3 (aq)	2.5	BnBr	3	Toluene	0.1	rt	78:22	N/A
CN6	0.1	50% K_2CO_3 (aq)	2.5	BnBr	3	Toluene	0.1	rt	76:24	N/A
CN6	0.2	50% K_2CO_3 (aq)	2.5	BnBr	3	Toluene	0.1	rt	75:25	N/A
CN6	0.025	50% K_2CO_3 (aq)	2.5	BnBr	3	Toluene	0.1	rt	85:15	N/A
CN1	0.05	25% NaOH (aq)	2.5	BnBr	3	Toluene	0.1	rt	50:50	N/A
CN1	0.05	50% K_3PO_4 (aq)	2.5	BnBr	3	Toluene	0.1	rt	70:30	N/A
CN1	0.05	50% Cs_2CO_3 (aq)	2.5	BnBr	3	Toluene	0.1	rt	77:23	N/A
CN1	0.05	25% $CsOH.H_2O$ (aq)	2.5	BnBr	3	Toluene	0.1	rt	52:48	N/A
CN1	0.05	25% $RbOH.H_2O$ (aq)	2.5	BnBr	3	Toluene	0.1	rt	54:46	N/A
CN1	0.05	50% K_2CO_3 (aq)	2.5	BnBr	3	Toluene	0.1	rt	74:26	N/A
CN1	0.05	50% Cs_2CO_3 (aq)	2.5	BnBr	3	Toluene	0.1	rt	77:23	N/A
CN2	0.05	50% Cs_2CO_3 (aq)	2.5	BnBr	3	Toluene	0.1	rt	72:28	N/A
CN3	0.05	50% Cs_2CO_3 (aq)	2.5	BnBr	3	Toluene	0.1	rt	73:27	N/A
CN4	0.05	50% Cs_2CO_3 (aq)	2.5	BnBr	3	Toluene	0.1	rt	51:49	N/A
CN6	0.05	50% Cs_2CO_3 (aq)	2.5	BnBr	3	Toluene	0.1	rt	82:18	N/A
CN7	0.05	50% Cs_2CO_3 (aq)	2.5	BnBr	3	Toluene	0.1	rt	68:32	N/A
CN8	0.05	50% Cs_2CO_3 (aq)	2.5	BnBr	3	Toluene	0.1	rt	70:30	N/A
CN9	0.05	50% Cs_2CO_3 (aq)	2.5	BnBr	3	Toluene	0.1	rt	60:40	N/A
CN10	0.05	50% Cs_2CO_3 (aq)	2.5	BnBr	3	Toluene	0.1	rt	75:25	N/A
CN11	0.05	50% Cs_2CO_3 (aq)	2.5	BnBr	3	Toluene	0.1	rt	72:28	N/A
CN12	0.05	50% Cs_2CO_3 (aq)	2.5	BnBr	3	Toluene	0.1	rt	50:50	N/A
M4	0.05	50% Cs_2CO_3 (aq)	2.5	BnBr	3	Toluene	0.1	rt	57:43	N/A
M12	0.05	50% Cs_2CO_3 (aq)	2.5	BnBr	3	Toluene	0.1	rt	49:51	N/A
M13	0.05	50% Cs_2CO_3 (aq)	2.5	BnBr	3	Toluene	0.1	rt	67:33	N/A
CN6	0.05	50% Cs_2CO_3 (aq)	2.5	BnBr	3	Toluene	0.1	rt	84:16	N/A
CN6	0.05	25% Cs_2CO_3 (aq)	2.5	BnBr	3	Toluene	0.1	rt	82:18	N/A
CN6	0.05	50% K_3PO_4 (aq)	2.5	BnBr	3	Toluene	0.1	rt	77:23	N/A
CN6	0.05	K_3PO_4 (s)	2.5	BnBr	3	Toluene	0.1	rt	64:36	N/A
CN6	0.05	Cs_2CO_3 (s)	2.5	BnBr	3	Toluene	0.1	rt	53:47	N/A
CN6	0.05	Cs_2CO_3 (s)	1.5	BnBr	3	Toluene	0.1	rt	53:47	N/A
CN6	0.05	15% Na_3PO_4 (aq)	2.5	BnBr	3	Toluene	0.1	rt	86:14	N/A
CN6	0.05	Li_2CO_3 (s)	2.5	BnBr	3	Toluene	0.1	rt	N/A	N/A
CN6	0.05	Ag_2CO_3 (s)	2.5	BnBr	3	Toluene	0.1	rt	80:20	N/A
CN6	0.05	Na_2CO_3 (s)	2.5	BnBr	3	Toluene	0.1	rt	92:8 (v. Slow)	N/A
CN6	0.05	2M Na_2CO_3 (aq)	2.5	BnBr	3	Toluene	0.1	rt	87:13	N/A
CN6	0.05	Na_3PO_4	2.5	BnBr	3	Toluene	0.1	rt	88:12	N/A
CN6	0.05	K_2CO_3 (s)	2.5	BnBr	3	Toluene	0.1	rt	75:25	N/A
CN6	0.05	KF (s)	2.5	BnBr	3	Toluene	0.1	rt	91:9	N/A
CN6	0.05	25% KF (aq)	2.5	BnBr	3	Toluene	0.1	rt	75:25	N/A
CN6	0.05	KF (s)	2.5	BnBr	3	Toluene	0.1	rt	90:10	N/A
CN6	0.05	KF (s)	2.5	BnBr	3	Benzene	0.1	rt	90:10	N/A
CN6	0.05	KF (s)	2.5	BnBr	3	p-xylene	0.1	rt	91:9	N/A
CN6	0.05	KF (s)	2.5	BnBr	3	m-xylene	0.1	rt	91:9	N/A
CN6	0.05	KF (s)	2.5	BnBr	3	CCl_4	0.1	rt	87:13	N/A
CN6	0.05	KF (s)	2.5	BnBr	3	$CHCl_3$	0.1	rt	77:23	N/A
CN6	0.05	KF (s)	2.5	BnBr	3	CH_2Cl_2	0.1	rt	83:17	N/A
CN6	0.05	KF (s)	2.5	BnBr	3	1,2-dichlororethane	0.1	rt	83:17	N/A
CN6	0.05	KF (s)	2.5	BnBr	3	Trichloroethane	0.1	rt	86:14	N/A
CN6	0.05	KF (s)	2.5	BnBr	3	Tetrachloroethane	0.1	rt	NR	N/A
CN6	0.05	KF (s)	2.5	BnBr	3	Et_2O	0.1	rt	87:13	N/A
CN6	0.05	KF (s)	2.5	BnBr	3	*i* Pr_2O	0.1	rt	88:12	N/A
CN6	0.05	KF (s)	2.5	BnBr	3	TBME	0.1	rt	84:16	N/A
QD1	0.05	50% Cs_2CO_3 (aq)	2.5	BnBr	3	Toluene	0.1	rt	66:34	N/A
QD3	0.05	50% Cs_2CO_3 (aq)	2.5	BnBr	3	Toluene	0.1	rt	66:34	N/A
QD4	0.05	50% Cs_2CO_3 (aq)	2.5	BnBr	3	Toluene	0.1	rt	81:19	N/A
QD5	0.05	50% Cs_2CO_3 (aq)	2.5	BnBr	3	Toluene	0.1	rt	79:21	N/A
QD6	0.05	50% Cs_2CO_3 (aq)	2.5	BnBr	3	Toluene	0.1	rt	70:30	N/A

a) Substrate concentration in the reaction solvent; b) Chiralpak ADH, 30% isopropanol, 70% hexane, 1.0 mL min^{-1}, λ= 260 nm; c) Determined by ^1H NMR spectroscopy; rt = room temperature; N/A = not applicable; NR = no reaction.

Catalyst	Catalyst Eq	Base	Base (equiv.)	Electrophile	Electrophile (equiv.)	Solvent	Concentration (M)[a]	Temperature	e.r.[b]	Conversion (%)[c]
QD11	0.05	50% Cs_2CO_3 (aq)	2.5	BnBr	3	Toluene	0.1	rt	78:22	N/A
QD12	0.05	50% Cs_2CO_3 (aq)	2.5	BnBr	3	Toluene	0.1	rt	68:32	N/A
QD14	0.05	50% Cs_2CO_3 (aq)	2.5	BnBr	3	Toluene	0.1	rt	64:36	N/A
QD15	0.05	50% Cs_2CO_3 (aq)	2.5	BnBr	3	Toluene	0.1	rt	*61:39*	N/A
QD17	0.05	50% Cs_2CO_3 (aq)	2.5	BnBr	3	Toluene	0.1	rt	66:34	N/A
QD18	0.05	50% Cs_2CO_3 (aq)	2.5	BnBr	3	Toluene	0.1	rt	69:31	N/A
QD19	0.05	50% Cs_2CO_3 (aq)	2.5	BnBr	3	Toluene	0.1	rt	68:32	N/A
CN13	0.05	50% Cs_2CO_3 (aq)	2.5	BnBr	3	Toluene	0.1	rt	90:10 (slower)	N/A
CN14	0.05	50% Cs_2CO_3 (aq)	2.5	BnBr	3	Toluene	0.1	rt	82:18	N/A
CN15	0.05	50% Cs_2CO_3 (aq)	2.5	BnBr	3	Toluene	0.1	rt	87:13	N/A
CN16	0.05	50% Cs_2CO_3 (aq)	2.5	BnBr	3	Toluene	0.1	rt	83:17	N/A
CN15	0.05	50% Cs_2CO_3 (aq)	2.5	BnBr	3	Toluene	0.1	rt	80:20	N/A
CN15	0.05	25% Cs_2CO_3 (aq)	2.5	BnBr	3	Toluene	0.1	rt	81:19	N/A
CN15	0.05	50% K_3PO_4 (aq)	2.5	BnBr	3	Toluene	0.1	rt	77:23	N/A
CN15	0.05	K_3PO_4 (s)	2.5	BnBr	3	Toluene	0.1	rt	73:27	N/A
CN15	0.05	Cs_2CO_3 (s)	2.5	BnBr	3	Toluene	0.1	rt	54:46	N/A
CN15	0.05	15% Na_3PO_4 (aq)	2.5	BnBr	3	Toluene	0.1	rt	89:11	N/A
CN15	0.05	Li_2CO_3 (s)	2.5	BnBr	3	Toluene	0.1	rt	NR	N/A
CN15	0.05	Ag_2CO_3 (s)	2.5	BnBr	3	Toluene	0.1	rt	83:17	N/A
CN15	0.05	Na_2CO_3 (s)	2.5	BnBr	3	Toluene	0.1	rt	94:6 (v. slow)	N/A
CN15	0.05	2M Na_2CO_3 (aq)	2.5	BnBr	3	Toluene	0.1	rt	84:16	N/A
CN15	0.05	Na_3PO_4	2.5	BnBr	3	Toluene	0.1	rt	93:7	N/A
CN15	0.05	K_2CO_3 (s)	2.5	BnBr	3	Toluene	0.1	rt	82:18	N/A
CN15	0.05	KF (s)	2.5	BnBr	3	Toluene	0.1	rt	91:9	N/A
CN15	0.05	25% KF (aq)	2.5	BnBr	3	Toluene	0.1	rt	92:8	N/A
CN15	0.05	50% K_2CO_3 (aq)	2.5	BnBr	3	Toluene	0.1	rt	81:19	N/A
CN15	0.05	25% KF (aq)	2.5	BnBr	3	Toluene	0.1	rt	93:7	N/A
CN15	0.05	25% KF (aq)	2.5	BnBr	3	Benzene	0.1	rt	94:6	N/A
CN15	0.05	25% KF (aq)	2.5	BnBr	3	p-xylene	0.1	rt	88:12	N/A
CN15	0.05	25% KF (aq)	2.5	BnBr	3	m-xylene	0.1	rt	90:10	N/A
CN15	0.05	25% KF (aq)	2.5	BnBr	3	CCl_4	0.1	rt	90:10	N/A
CN15	0.05	25% KF (aq)	2.5	BnBr	3	$CHCl_3$	0.1	rt	87:13	N/A
CN15	0.05	25% KF (aq)	2.5	BnBr	3	CH_2Cl_2	0.1	rt	86:14	N/A
CN15	0.05	25% KF (aq)	2.5	BnBr	3	1,2-dichlororethane	0.1	rt	80:20	N/A
CN15	0.05	25% KF (aq)	2.5	BnBr	3	Trichloroethane	0.1	rt	92:8	N/A
CN15	0.05	25% KF (aq)	2.5	BnBr	3	o-xylene	0.1	rt	91:9	N/A
CN15	0.05	25% KF (aq)	2.5	BnBr	3	Et_2O	0.1	rt	79:21	N/A
CN15	0.05	25% KF (aq)	2.5	BnBr	3	*i* Pr_2O	0.1	rt	60:40	N/A
CN15	0.05	25% KF (aq)	2.5	BnBr	3	MTBE	0.1	rt	80:20	N/A
CN15	0.05	25% KF (aq)	2.5	BnCl	3	Benzene	0.1	rt	94:6	N/A
CN15	0.05	25% KF (aq)	2.5	BnBr	3	Benzene	0.1	rt	93:7	N/A
CN15	0.05	25% KF (aq)	2.5	BnI	3	Benzene	0.1	rt	95:5	N/A
CN15	0.05	25% KF (aq)	2.5	BnOTs	3	Benzene	0.1	rt	85:15	N/A
CN15	0.05	25% KF (aq)	2.5	allyl bromide	3	Benzene	0.1	rt	75:25	N/A
CN15	0.05	25% KF (aq)	2.5	allyl iodide	3	Benzene	0.1	rt	73:27	N/A
CN15	0.05	25% KF (aq)	2.5	MeI	3	Benzene	0.1	rt	73:27	N/A
CN15	0.05	25% KF (aq)	2.5	propargyl bromide	3	Benzene	0.1	rt	64:36	N/A
CN15	0.05	25% KF (aq)	2.5	prenyl bromide	3	Benzene	0.1	rt	90:10	N/A
CN15	0.05	25% KF (aq)	2.5	1-bromonaphthalene	3	Benzene	0.1	rt	90:10	N/A
CN15	0.05	25% KF (aq)	2.5	2-bromonaphthalene	3	Benzene	0.1	rt	92:8	N/A
CN15	0.05	25% KF (aq)	2.5	Ac_2O	3	Benzene	0.1	rt	44:56	N/A
CN15	0.05	25% KF (aq)	2.5	BnI	3	Benzene	0.01	rt	97:3	N/A
CN15	0.05	25% KF (aq)	2.5	BnI	3	Benzene	0.05	rt	96:4	N/A
CN15	0.05	25% KF (aq)	2.5	BnI	3	Benzene	0.1	rt	95:5	N/A
CN15	0.05	25% KF (aq)	2.5	BnI	3	Benzene	0.2	rt	94:6	N/A
CN15	0.05	25% KF (aq)	2.5	BnI	3	Benzene	0.5	rt	NR	N/A
CN15	0.05	25% KF (aq)	2.5	BnI	3	Benzene	1	rt	85:15	N/A
CN15	0.05	50% Cs_2CO_3 (aq)	2.5	BnI	3	Benzene	0.1	rt	89:11	N/A
CN15	0.05	50% KF (aq)	2.5	BnBr	3	Benzene	0.1	rt	93:7	N/A
CN16	0.05	25% KF (aq)	2.5	BnI	3	Benzene	0.1	rt	96:4	N/A
CN15	0.1	25% KF (aq)	2.5	BnI	3	Benzene	0.01	rt	97:3	36
CN15	0.1	25% KF (aq)	2.5	BnI	3	Benzene	0.05	rt	95:5	36
CN15	0.1	25% KF (aq)	2.5	BnI	3	Benzene	0.1	rt	94:6	50
CN15	0.1	25% KF (aq)	2.5	BnI	3	Benzene	0.2	rt	93:7	33
CN15	0.2	25% KF (aq)	2.5	BnI	3	Benzene	0.01	rt	96:4	37
CN15	0.2	25% KF (aq)	2.5	BnI	3	Benzene	0.05	rt	94:6	42
CN15	0.2	25% KF (aq)	2.5	BnI	3	Benzene	0.1	rt	92:8	34
CN15	0.2	25% KF (aq)	2.5	BnI	3	Benzene	0.2	rt	87:13	22
CN15	0.1	25% KF (aq)	5	BnI	3	Benzene	0.1	rt	93:7	63
CN15	0.1	50% KF (aq)	2.5	BnI	3	Benzene	0.1	rt	94:6	60
CN16	0.1	25% KF (aq)	2.5	BnI	3	Benzene	0.1	rt	96:4	38
CN15	0.1	25% KF (aq)	2.5	BnI	3	10% DCM/Benzene	0.1	rt	94:6	54
CN15	0.1	25% KF (aq)	2.5	BnI	3	10% $CHCl_3$/Benzene	0.1	rt	94:6	45
CN16	0.1	25% CsF (aq)	2.5	BnI	3	Benzene	0.1	rt	94:6	14
CN15	0.05	50% Cs_2CO_3 (aq)	5	BnI	3	Benzene	0.05	rt	94:6	82
CN15	0.1	50% Cs_2CO_3 (aq)	5	BnI	3	Benzene	0.05	rt	94:6	90
CN16	0.05	50% Cs_2CO_3 (aq)	5	BnI	3	Benzene	0.05	rt	95:5	95
CN15	0.05	50% Cs_2CO_3 (aq)	5	BnI	3	Benzene	0.05	rt	94:6	86
CN15	0.1	50% Cs_2CO_3 (aq)	5	BnI	3	Benzene	0.05	rt	-	-
CN16	0.05	50% Cs_2CO_3 (aq)	5	BnI	3	Benzene	0.05	rt	95:5	92
CN15	0.1	50% Cs_2CO_3 (aq)	5	BnI	3	Benzene	0.05	rt	93:7	68
CN24	0.1	50% Cs_2CO_3 (aq)	5	BnI	3	Benzene	0.05	rt	93:7	68
CN16	0.05	50% Cs_2CO_3 (aq)	5	BnI	3	Benzene	0.05	rt	95:5	73
CN25	0.05	50% Cs_2CO_3 (aq)	5	BnI	3	Benzene	0.05	rt	96:4	65

a) Substrate concentration in the reaction solvent; b) Chiralpak ADH, 30% isopropanol, 70% hexane, 1.0 mL min^{-1}, λ= 260 nm; c) Determined by ^{1}H NMR spectroscopy; rt = room temperature; N/A = not applicable; NR = no reaction.

Catalyst	Catalyst Eq	Base	Base (equiv.)	Electrophile	Electrophile (equiv.)	Solvent	Concentration (M)[a]	Temperature	e.r.[b]	Conversion (%)[c]
CN16	0.05	50% Cs_2CO_3 (aq)	5	BnI	3	Benzene	0.05	10	93:7	69
CN25	0.05	50% Cs_2CO_3 (aq)	5	BnI	3	Benzene	0.05	10	95:5	56
CN15	0.1	50% Cs_2CO_3 (aq)	5	BnI	3	Benzene	0.05	30	94:6	77
CN16	0.05	50% Cs_2CO_3 (aq)	5	BnI	3	Benzene	0.05	30	94:6	79
CN25	0.05	50% Cs_2CO_3 (aq)	5	BnI	3	Benzene	0.05	30	96:6	-
CN15	0.1	50% Cs_2CO_3 (aq)	5	BnI	3	Benzene	0.05	40	90:10	100
CN16	0.05	50% Cs_2CO_3 (aq)	5	BnI	3	Benzene	0.05	40	92:8	96
CN25	0.05	50% Cs_2CO_3 (aq)	5	BnI	3	Benzene	0.05	40	92:8	85
CN15	0.05	50% Cs_2CO_3 (aq)	5	BnI	3	Benzene	0.05	rt	94:6	65
CN15	0.1	50% Cs_2CO_3 (aq)	5	BnI	3	Benzene	0.05	rt	93:7	69
CN25	0.05	50% Cs_2CO_3 (aq)	5	BnI	3	Benzene	0.05	rt	95:5	65
CN25	0.1	50% Cs_2CO_3 (aq)	5	BnI	3	Benzene	0.05	rt	95:5	66
CN15	0.1	50% Cs_2CO_3 (aq)	5	BnI	3	Benzene	0.025	rt	96:4	56
CN16	0.05	50% Cs_2CO_3 (aq)	5	BnI	3	Benzene	0.025	rt	96:4	70
CN25	0.05	50% Cs_2CO_3 (aq)	5	BnI	3	Benzene	0.025	rt	97:3	40
CN15	0.1	50% Cs_2CO_3 (aq)	5	BnI	3	Benzene	0.01	rt	97:3	42
CN16	0.05	50% Cs_2CO_3 (aq)	5	BnI	3	Benzene	0.01	rt	97:3	57
CN25	0.05	50% Cs_2CO_3 (aq)	5	BnI	3	Benzene	0.01	rt	97:3	25
CN16	0.05	50% Cs_2CO_3 (aq)	5	BnI	3	Benzene	0.025	rt	96:4	97
CN16	0.05	50% Cs_2CO_3 (aq)	5	BnI	3	Benzene	0.01	rt	96:4	96
CN16	0.025	50% Cs_2CO_3 (aq)	5	BnI	3	Benzene	0.05	rt	95:5	93
CN16	0.025	50% Cs_2CO_3 (aq)	5	BnI	3	Benzene	0.025	rt	94:6	97
CN25	0.05	50% Cs_2CO_3 (aq)	5	BnI	3	Benzene	0.025	rt	95:5	94
CN25	0.05	50% Cs_2CO_3 (aq)	5	BnI	3	Benzene	0.01	rt	97:3	83

a) Substrate concentration in the reaction solvent; b) Chiralpak ADH, 30% isopropanol, 70% hexane, 1.0 mL min^{-1}, λ= 260 nm; c) Determined by ^{1}H NMR spectroscopy; rt = room temperature; N/A = not applicable; NR = no reaction.

1.6 Experimental Procedures

1.6.1 Starting material synthesis

2-(2-Bromo-6-iodophenyl)acetic acid, S1

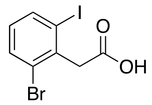

According to modified literature procedure,[5] 2-bromoacetic acid (2.15 g, 10.0 mmol) was dissolved in anhydrous DMF (40 mL) with palladium acetate (225 mg, 1.00 mmol), PIDA (2.41 g, 7.5 mmol) and iodine (1.9 g, 7.50 mmol). The flask was covered in aluminium foil and heated to 60 °C. After 24 h, the mixture was cooled to room temperature and additional PIDA (2.41 g, 7.50 mmol) and iodine (1.9 g, 7.5 mmol) were added. The reaction was covered in foil and heated to 60 °C. After a further 24 h, additional palladium acetate (225 mg, 1.00 mmol), PIDA (2.41 g, 7.50 mmol) and iodine (1.9 g, 7.50 mmol) were added. The reaction was covered in foil and heated to 60 °C. After a further 24 h, additional PIDA (2.41 g, 7.50 mmol) and iodine (1.90 g, 7.50 mmol) were added. The reaction was covered in foil and heated to 60 °C for 24 h. The mixture was cooled to room temperature and diluted with ethyl acetate and washed with saturated aqueous sodium sulfite, dried with sodium sulfate, filtered, and concentrated *in vacuo* to give a crude mixture which was purified by column chromatography, eluting with 10–50% ethyl acetate in petrol, to yield title compound **S1** as a white solid (2.83 g, 85%).

m.p. 145–148 °C

^{1}H NMR (500 MHz, $CDCl_3$) δ_H: 7.82 (dd, J = 7.9, 1.2 Hz, 1H), 7.58 (dd, J = 8.0, 1.2 Hz, 1H), 6.82 (t, J = 8.0 Hz, 1H), 4.22 (s, 2H).

^{13}C{^{1}H} NMR (126 MHz, $CDCl_3$) δ_C: 175.1, 139.0, 136.7, 133.2, 130.6, 124.9, 101.9, 47.3.

FTIR (neat) ν/cm^{-1} = 2981, 1698, 1550, 1428, 1410, 1244, 926, 775, 699.

HRMS (m/z, CI^+): calculated for $C_8H_{10}BrINO_2$ ($[M+NH_4]^+$) = 357.8934, found = 357.8939.

2-(2-Bromo-6-iodophenyl)-*N*,*N*-diisopropylacetamide, S2

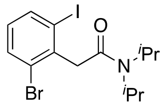

Acid **S1** (2.00 g, 5.87 mmol) was dissolved in anhydrous dichloromethane. Oxalyl chloride (960 µL, 11.7 mmol) and DMF (0.1 mL) were added and the reaction was stirred at room temperature for 1 h until no more bubbles were produced. The solution was concentrated *in vacuo* and redissolved in anhydrous dichloromethane with diisopropylamine (2.47 mL, 17.6 mmol) and stirred at room temperature for 2 h. The solution was diluted with dichloromethane and washed with water and brine. The organic phase was dried with anhydrous sodium sulfate, filtered and concentrated *in vacuo* to give the crude mixture, which was purified by column chromatography, eluting with 5–10% ethyl acetate in petrol, to yield title compound **S2** as a white solid (2.07 g, 83%).

m.p. 114–116 °C

^{1}H NMR (500 MHz, $CDCl_3$) δ_H: 7.78 (dd, J = 7.9, 1.2 Hz, 1H), 7.54 (dd, J = 8.0, 1.2 Hz, 1H), 6.76 (t, J = 7.9 Hz, 1H), 4.12 (s, 2H), 4.12 (hept, J = 6.5 Hz, 1H), 3.48 (hept, J = 6.8 Hz, 1H), 1.41 (d, J = 6.8 Hz, 6H), 1.32 (d, J = 6.6 Hz, 6H).

^{13}C{^{1}H} NMR (126 MHz, $CDCl_3$) δ_C: 166.3, 139.4, 138.7, 132.9, 129.7, 125.0, 102.6, 48.9, 48.4, 46.3, 21.2, 20.9.

FTIR (neat) ν/cm^{-1} = 2962, 1639, 1572, 1549, 1432, 1374, 1336, 1283, 1134, 1045, 785, 700.

HRMS (m/z, ESI$^+$) calculated for $C_{14}H_{20}BrINO$ ([M+H]$^+$) = 423.9768, found = 423.9761.

Methyl (*E*)-3-(3-bromo-2-(2-(diisopropylamino)-2-oxoethyl)phenyl)acrylate, S3

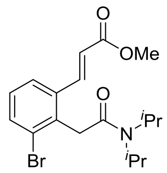

S2 (817 mg, 1.93 mmol) was dissolved in anhydrous DMF (2.0 mL) in a sealed vial with $Pd_2(dba)_3$ (88 mg, 0.093 mmol), methyl acrylate (0.217 mL, 2.41 mmol) and potassium carbonate (532 mg, 3.85 mmol). The solution was heated to 110 °C for 16 h, then diluted with ethyl acetate and washed with water. The organic phase was dried with anhydrous sodium sulfate, filtered, and concentrated *in vacuo* to give the crude mixture which was purified by column chromatography, eluting with 5–10% ethyl acetate in petrol, to yield **S3** as a white solid (650 mg, 88%).

m.p.: 126–128°C

^{1}H NMR (500 MHz, $CDCl_3$) δ_H: 7.83 (d, *J* = 15.8 Hz, 1H), 7.59 (dd, *J* = 8.0, 1.3 Hz, 1H), 7.48 (dd, *J* = 7.7, 1.1 Hz, 1H), 7.13 (t, *J* = 7.9 Hz, 1H), 6.31 (d, *J* = 15.7 Hz, 1H), 4.15 (hept, *J* = 6.7 Hz, 1H), 3.96 (s, H7, 2H), 3.79 (s, H10, 3H), 3.55–3.39 (m, 1H), 1.39 (d, *J* = 6.8 Hz, 6H), 1.32 (d, *J* = 6.6 Hz, 6H).

^{13}C{^{1}H} NMR (126 MHz, $CDCl_3$) δ_C: 167.3, 167.1, 142.9, 136.9 135.9, 134.1, 128.4, 127.1, 126.2, 121.4, 51.9, 49.1, 46.4, 39.4, 21.1, 20.7.

FTIR (neat) v/cm^{-1} = 2981,1719, 1639, 1556, 1443, 1372, 1334, 1315, 1276, 1196, 1169, 1042, 978, 909.

HRMS (*m/z*, ESI$^+$): calculated for $C_{18}H_{25}NO_3Br$ ($[M+H]^+$) = 382.1012, found = 382.1014.

8-Bromo-2-hydroxy-*N*,*N*-diisopropyl-1-naphthamide, 1

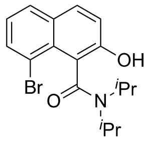

Prepared according to **General Procedure J** with acrylate **S2** (300 mg, 0.856 mmol), LiHMDS (3.39 mL, 1.695 mmol) and $Ir(fppy)_3$ (6.5 mg, 0.00848 mmol) in THF (17.0 mL). Purification *via* trituration with ethyl acetate and washing with hexane afforded title compound **1** as a white solid (205 mg, 78%).

m.p. 230–232 °C

^{1}H NMR (500 MHz, $CDCl_3$) δ_H: 8.84 (1H, s), 7.74 (1H, dd, *J* = 7.4, 1.3 Hz), 7.63 (1H, dd, *J* = 8.1, 1.3 Hz), 7.29 (1H, d, *J* = 8.8 Hz), 7.10 (1H, t, *J* = 7.7 Hz), 6.86 (1H, d, *J* = 8.8 Hz), 3.63 (1H, hept, *J* = 6.7 Hz), 3.53 (1H, hept, *J* = 6.8 Hz), 1.64 (3H, d, *J* = 6.8 Hz), 1.49 (3H, d, *J* = 6.8 Hz), 1.14 (3H, d, *J* = 6.7 Hz), 0.94 (3H, d, *J* = 6.6 Hz).

^{13}C{^{1}H} NMR (126 MHz, $CDCl_3$) δ_C: 170.2, 154.5, 133.2, 130.7, 130.3, 129.3, 128.7, 123.4, 120.1, 118.4, 117.9, 51.9, 46.5, 20.6, 20.5, 19.2, 19.2.

FTIR (neat) ν/cm^{-1} = 2975, 2931, 1590, 1507, 1428, 1338, 1295, 1210, 1162, 1052, 828.

HRMS (m/z, ESI+): calculated for $C_{17}H_{21}BrO_2N$ ($[M+H]^+$) = 350.0750, found = 350.0751.

Scheme S1

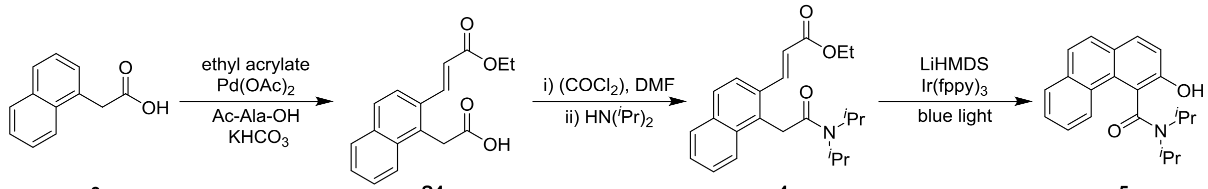

3 **S4** **4** **5**

(*E*)-2-(2-(3-Ethoxy-3-oxoprop-1-en-1-yl)naphthalen-1-yl)acetic acid, S4

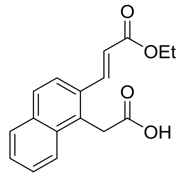

Prepared according to **General Procedure A** with 1-naphthylacetic acid (5.00 g, 26.9 mmol), potassium bicarbonate (5.40 g, 53.8 mmol), (*S*)-*N*-acetylalanine (705 mg, 5.38 mmol), palladium acetate (604 mg, 2.69 mmol) and ethyl acrylate (5.8 mL, 53.8 mmol) in tert-amyl alcohol (20 mL). Purification by column chromatography, eluting with 30% ethyl acetate, 1% acetic acid in petrol, yielded title compound **S4** as a beige solid (7.05 g, 92%).

m.p. 161 °C

^{1}H NMR (500 MHz, $CDCl_3$) δ_H: 8.21 (d, *J* = 15.7 Hz, 1H), 8.05 (d, *J* = 8.5 Hz, 1H), 7.83 (d, *J* = 8.1 Hz, 1H), 7.78 (d, *J* = 8.6 Hz, 1H), 7.66 (d, *J* = 8.7 Hz, 1H), 7.55 (dd, *J* = 8.4, 6.8 Hz, 1H), 7.51 (dd, *J* = 8.0, 6.8 Hz, 1H), 6.48 (d, *J* = 15.7 Hz, 1H), 4.28 (q, *J* = 7.1 Hz, 2H), 4.28 (s, 2H), 1.34 (t, *J* = 7.1 Hz, 3H).

^{13}C{^{1}H} NMR (126 MHz, $CDCl_3$) δ_C: 176.3, 167.0, 142.2, 134.3, 132.6, 131.9, 129.9, 128.8, 128.7, 127.4, 127.0, 124.6, 123.9, 121.7, 60.9, 34.0, 14.4.

FTIR (neat) ν/cm^{-1} = 3060, 1708, 1628, 1368, 1303, 1157, 1038, 979, 816.

HRMS (*m/z*, ESI$^+$): calculated for $C_{17}H_{16}NaO_4$ ($[M+Na]^+$) = 307.0941, found = 307.0944.

Ethyl (*E*)-3-(1-(2-(diisopropylamino)-2-oxoethyl)naphthalen-2-yl)acrylate, 4

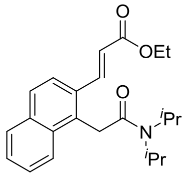

Prepared according to **General Procedure B** with acid **S4** (7.85 g, 27.6 mmol), oxalyl chloride (11.3 mL, 55.2 mmol) and DMF (500 µL) in anhydrous dichloromethane (30 mL) followed by diisopropylamine (7.3 mL, 55.2 mmol) in anhydrous dichloromethane (30 mL). Purification by recrystallisation in hot ethyl acetate. The solid was filtered off to give a white solid (5.76 g) and the filtrate was purified by column chromatography, eluting with 10–20% ethyl acetate in petrol, yielded title compound **4** as a white solid (1.15 g, combined yield = 6.91 g, 68%).

m.p. 147–149 °C

^{1}H NMR (500 MHz, $CDCl_3$) δ_H: 8.09 (d, *J* = 15.7 Hz, 1H), 7.96 (d, *J* = 8.52 Hz, 1H), 7.81 (d, *J* = 7.9, 1.7 Hz, 1H), 7.75 (d, *J* = 8.6 Hz, 1H), 7.65 (d, *J* = 8.7 Hz, 1H), 7.55–7.45 (m, 2H), 6.45 (d, *J* = 15.7 Hz, 1H), 4.27 (q, *J* = 7.1 Hz, 3H), 4.23 (s, 2H), 3.56–3.42 (m, 1H), 1.38 (d, *J* = 6.8 Hz, 6H), 1.34 (t, *J* = 6.6 Hz, 3H), 1.32 (d, *J* = 6.1 Hz, 6H).

^{13}C{^{1}H} NMR (126 MHz, $CDCl_3$) δ_C: 168.5, 166.9, 142.5, 134.3, 133.1, 133.1, 131.4, 128.6 , 127.8, 126.8, 126.5, 124.6, 123.8, 120.8, 60.5, 49.0, 46.2, 35.1, 21.1, 20.7, 14.4.

FTIR (neat) ν/cm^{-1} = 2963, 2933, 1713, 1626, 1438, 1341, 1299, 1278, 1175, 1154, 977, 817.

HRMS (*m/z*, ESI$^+$): calculated for $C_{23}H_{29}NNaO_3$ ([M+Na]$^+$) = 390.2040, found = 390.2028.

3-Hydroxy-*N*,*N*-diisopropylphenanthrene-4-carboxamide, 5

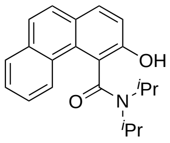

Prepared according to **General Procedure J** with acrylate **4** (300 mg, 0.816 mmol), LiHMDS (3.39 mL, 1.695 mmol) and Ir(fppy)$_3$ (6.5 mg, 0.00848 mmol) in THF (17.0 mL). Purification *via* trituration with ethyl acetate and washing with hexane afforded title compound **5** as a white solid (205 mg, 78%).

m.p. > 225 °C

^{1}H NMR (500 MHz, $CDCl_3$) δ_H: 8.68 (d, *J* = 8.4 Hz, 1H), 7.85 (d, *J* = 7.8 Hz, 1H), 7.65 (d, *J* = 8.4 Hz, 1H), 7.63 (d, *J* = 8.4 Hz, 1H), 7.60 (d, *J* = 8.7 Hz, 1H), 7.56 (dd, *J* = 7.9, 6.9 Hz, 1H), 7.50 (dd, *J* = 8.4, 6.9 Hz, 1H), 7.34 (s, 1H), 7.16 (d, *J* = 8.6 Hz, 1H), 3.54 (hept, *J* = 6.8 Hz, 1H), 3.36 (hept, *J* = 6.6 Hz, 1H), 1.80 (d, *J* = 6.9 Hz, 3H), 1.69 (d, *J* = 6.8 Hz, 3H), 0.86 (d, *J* = 6.6 Hz, 3H), 0.51 (d, *J* = 6.6 Hz, 3H.)

^{13}C{^{1}H} NMR (126 MHz, $CDCl_3$) δ_C: 171.5, 153.3, 133.4, 130.6, 129.0, 128.5, 127.9, 127.2, 127.2, 127.0, 126.9, 125.5, 125.1, 119.4, 118.2, 51.5, 46.7, 21.2, 20.9, 19.3, 19.2.

FTIR (neat) ν/cm^{-1} = 2998, 1596, 1572, 1525, 1475, 1450, 1399, 1286, 1160, 1087, 969, 854.

HRMS (*m/z*, ESI$^+$): calculated for $C_{21}H_{24}NO_2$ ([M+H]$^+$) = 322.1802, found = 322.1803.

Scheme S2

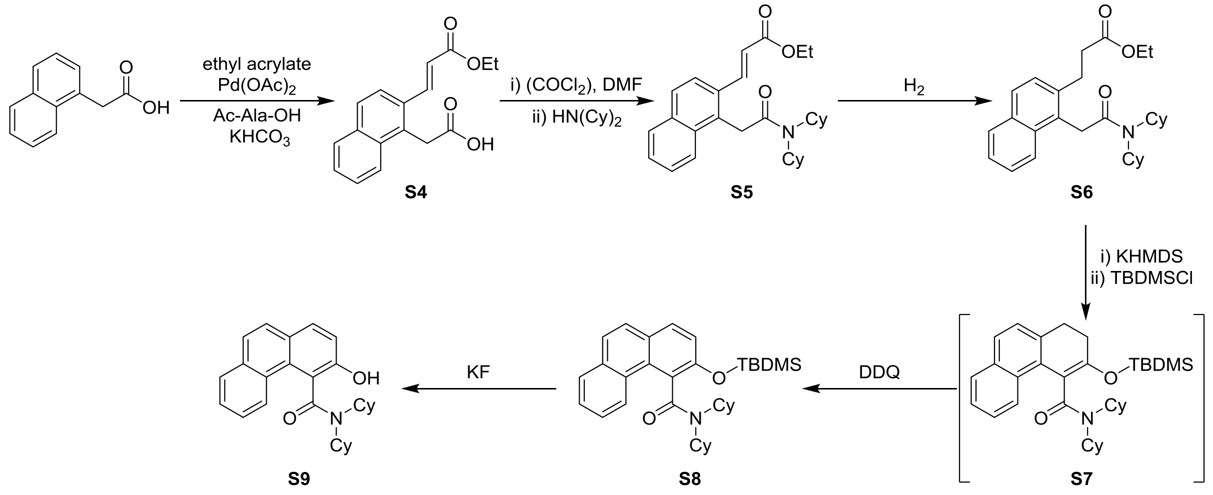

Ethyl (*E*)-3-(1-(2-(dicyclohexylamino)-2-oxoethyl)naphthalen-2-yl)acrylate, S5

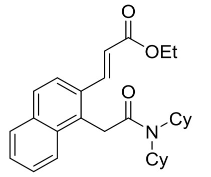

Prepared according to **General Procedure B** with acid **S4** (4.60 g, 16.2 mmol), oxalyl chloride (6.60 mL, 32.4 mmol) and DMF (500 µL) in anhydrous dichloromethane (50 mL) followed by dicyclohexylamine (3.53 mL, 19.4 mmol) in anhydrous dichloromethane (50 mL). Purification by column chromatography, eluting with 10% ethyl acetate in petrol, yielded title compound **S5** as a white solid (1.90 g, 26%).

^{1}H NMR (500 MHz, $CDCl_3$) δ_H: 8.13 (d, *J* = 15.7 Hz, 1H), 8.00 (d, *J* = 8.2 Hz, 1H), 7.81 (dd, *J* = 7.8, 1.6 Hz, 1H), 7.74 (d, *J* = 8.7 Hz, 1H), 7.65 (d, *J* = 8.7 Hz, 1H), 7.55-7.42 (m, 2H), 6.46 (d, *J* = 15.7 Hz, 1H), 4.28 (q, *J* = 7.1 Hz, 2H), 4.25 (s, 2H), 3.74-3.65 (m, 1H), 2.96–2.92 (m, 1H), 2.44–2.40 (m, 2H), 1.86–1.78 (m, 2H), 1.77–1.69 (m, 4H), 1.73–1.57 (m, 3H), 1.56–1.45 (m, 4H), 1.34 (t, *J* = 7.1 Hz, 3H), 1.31–1.03 (m, 5H).

^{13}C{^{1}H} NMR (126 MHz, $CDCl_3$) δ_C: 169.1, 167.0, 142.6, 134.4, 133.4, 133.1, 131.4, 128.7, 127.9, 126.9, 126.7, 124.8, 123.8, 121.0, 60.6, 58.2, 56.6, 36.0, 31.5, 30.0, 26.8, 26.1, 25.5, 25.4, 14.5.

m.p.: 79–81 °C

FTIR (neat) ν/cm^{-1} = 2928, 2852, 1708, 1630, 1432, 1364, 1261, 1261, 1175, 1038, 976, 894, 813, 747.

HRMS (*m/z*, ESI$^+$): calculated for $C_{29}H_{38}NO_3$ ([M+H]$^+$) = 448.2846, found = 448.2835.

Ethyl 3-(1-(2-(dicyclohexylamino)-2-oxoethyl)naphthalen-2-yl)propanoate, S6

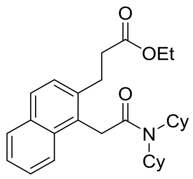

Prepared according to **General Procedure D** with acrylate **S5** (1.85 g, 4.14 mmol) in methanol (10 mL) yielded title compound **S6** as a white solid without need for further purification (1.80 g, 97%).

m.p. 75 °C

^{1}H NMR (600 MHz, $CDCl_3$) δ_H: 7.88 (dd, *J* = 8.5, 1.1 Hz, 1H), 7.78 (dd, *J* = 8.1, 1.3 Hz, 1H), 7.70 (d, *J* = 8.4 Hz, 1H), 7.46 (ddd, *J* = 8.4, 6.7, 1.4 Hz, 1H), 7.40 (ddd, *J* = 8.0, 6.7, 1.1 Hz, 1H), 7.32 (d, *J* = 8.4, 1H Hz, H6), 4.14 (s, 2H), 4.14 (q, *J* = 7.1 Hz, 2H), 3.76–3.72 (m, 1H), 3.14–3.08 (m, 2H), 3.00–2.86 (m, 1H), 2.68–2.61 (m, 2H), 2.51–2.33 (m, 2H), 1.89–1.76 (m, 2H), 1.76–1.69 (m, 4H), 1.69–1.63 (m, 1H), 1.62–1.50 (m, 3H), 1.50–1.44 (m, 2H), 1.24 (t, *J* = 7.2, 5H), 1.21–1.17 (m, 2H), 1.14–1.05 (m, 2H).

^{13}C{^{1}H} NMR (151 MHz, $CDCl_3$) δ_C: 173.2, 169.6, 136.7, 133.3, 132.8, 130.2, 128.7, 127.7, 127.6, 126.3, 125.0, 124.0, 60.6, 58.1, 56.5, 35.6, 31.6, 30.1, 29.5, 26.8, 26.6, 26.2, 25.9, 25.5, 25.4, 14.4.

FTIR (neat) ν/cm^{-1} = 2972, 2931, 1732, 1503, 1453, 1369, 1367, 1268, 794, 738.

HRMS (*m/z*, ESI^{+}): calculated for $C_{29}H_{40}NO_3$ ([M+H]$^{+}$) = 450.3003, found = 450.3008.

3-((*Tert*-butyldimethylsilyl)oxy)-*N*,*N*-dicyclohexylphenanthrene-4-carboxamide, S8

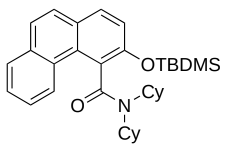

Prepared according to **General Procedure E** with amide **S6** (1.79 g, 3.98 mmol) and KHMDS (16 mL, 0.5 M, 7.97 mmol) in anhydrous THF (10 mL), followed by TBDMSCl (1.20 g, 7.97 mmol). Purification by column chromatography, eluting with 5–10% diethyl ether in petrol, yielded intermediate **S7** as a white solid (1.21 g, 59%).

Then, according to **General Procedure F** with intermediate **S7** (1.19 g, 2.30 mmol) and DDQ (657 mg, 2.99 mmol) in toluene (10 mL) for 30 min. Purification by column chromatography, eluting with 5–10% diethyl ether in petrol, yielded title compound **S8** as a white solid (905 mg, 76%, combined yield = 45%).

m.p. 105–110 °C

^{1}H NMR (500 MHz, $CDCl_3$) δ_H: 9.03 (d, *J* = 8.9 Hz, 1H), 7.81 (dd, *J* =7.8, 1.5 Hz, 1H), 7.73 (d, *J* = 8.7 Hz, 1H), 7.63 (d, *J* = 8.8 Hz, 1H), 7.59 (d, *J* = 8.7 Hz, 1H), 7.53 (ddd, *J* =7.9, 6.9, 1.2 Hz, 1H), 7.48 (ddd, *J* = 8.5, 6.9, 1.6 Hz, 1H), 7.18 (d, *J* = 8.6 Hz, 1H), 3.21–3.03 (m, 1H), 2.96–2.86 (m, 1H), 2.02–1.94 (m, 1H), 1.97–1.86 (m, 2H), 1.86–1.74 (m, 2H), 1.73–1.66 (m, 1H), 1.61–1.52 (m, 1H), 1.48–1.16 (m, 8H), 1.19–1.04 (m, 1H), 1.06 (s, 9H), 0.99–0.93 (m, 1H), 0.88–0.67 (m, 2H), 0.38 (s, 3H), 0.27 (s, 3H).

^{13}C{^{1}H} NMR (126 MHz, $CDCl_3$) δ_C: 170.3, 151.6, 133.2, 129.7, 129.5, 128.4, 128.2, 127.7, 127.6, 127.2, 126.7, 125.8, 125.6, 125.5, 120.0, 60.5, 56.5, 31.4, 31.1, 30.7, 29.1, 27.0, 27.0, 26.2, 25.8, 25.6, 25.5, 25.2, 18.8, −3.4, −3.9.

FTIR (neat) ν/cm^{-1} = 2929, 2855, 1627, 1503, 1453, 1290, 1229, 1098, 946, 839, 731.

HRMS (m/z, ESI$^+$): calculated for $C_{33}H_{46}NO_2Si$ ([M+H]$^+$) = 516.3292, found = 516.3291.

***N*,*N*-Dicyclohexyl-3-hydroxyphenanthrene-4-carboxamide, S9**

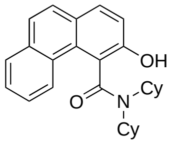

Prepared according to **General Procedure H** with silylated naphthol **S8** (960 mg, 1.86 mmol) and potassium fluoride (216 mg, 3.72 mmol) in methanol (20 mL). Purification by trituration with ethyl acetate and washing with hexane yielded title compound **S9** as a white solid (697 mg, 97%).

m.p. 225 °C

^{1}H NMR (500 MHz, $CDCl_3$) δ_H: 8.74 (d, *J* = 8.4 Hz, 1H), 7.86 (d, *J* = 7.9 Hz, 1H), 7.83 (s, 1H), 7.64 (d, *J* = 8.7 Hz, 1H), 7.62 (d, *J* = 8.5 Hz, 1H), 7.60 (d, *J* = 8.7 Hz, 1H), 7.57 (dd, *J* = 7.9, 6.8, Hz, 1H), 7.50 (dd, *J* = 8.4, 6.9 Hz, 1H), 7.13 (d, *J* = 8.5 Hz), 3.15–3.05 (m, 1H), 3.01–2.83 (m, 3H), 2.05–1.85 (m, 3H), 1.82–1.71 (m, 2H), 1.70–1.63 (m, 1H), 1.55–1.47 (m, 1H), 1.47–1.28 (m, 3H), 1.27–1.21 (m, 2H), 1.21–1.06 (m, 2H), 0.93–0.87 (m, 1H), 0.85–0.72 (m, 1H), 0.63–0.50 (m, 1H), 0.29–0.17 (m, 1H).

^{13}C{^{1}H} NMR (126 MHz, $CDCl_3$) δ_C: 171.8, 153.2, 133.2, 130.3, 128.9, 128.3, 127.8, 127.2, 127.1, 126.7, 126.5, 125.3, 124.7, 119.3, 118.0, 60.6, 56.8, 31.0, 29.9, 29.9, 28.7, 26.7, 25.5. 25.5, 25.3, 25.0.

FTIR (neat) ν/cm^{-1} = 3138, 2927, 2849, 1611, 1590, 1573, 1509, 1472, 1283, 1230, 833, 714.

HRMS (*m/z*, ESI$^+$): calculated for $C_{27}H_{32}NO_2$ ([M+H]$^+$) = 402.2428, found = 402.2423.

Scheme S3

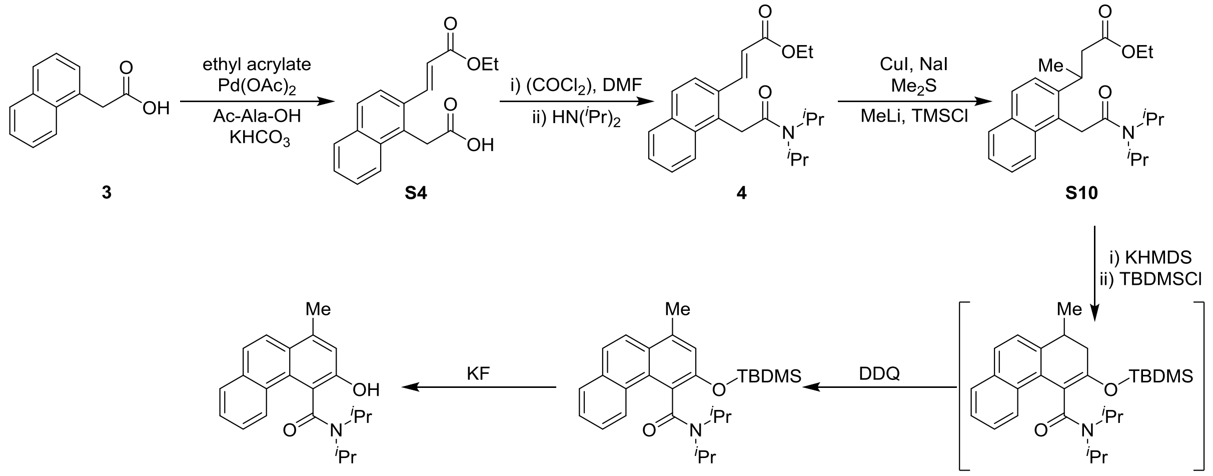

Ethyl 3-(1-(2-(diisopropylamino)-2-oxoethyl)naphthalen-2-yl)butanoate, S10

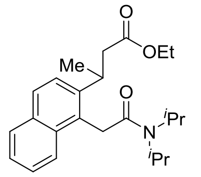

In a flame-dried two-necked flask under argon was added copper iodide (2.80 g, 14.7 mmol), sodium iodide (4.08 g, 27.2 mmol) and dimethyl sulphide (9.0 mL, 0.6 mL/mmol CuI) in anhydrous dichloromethane (30 mL). The solution was cooled to −78 °C and MeLi (8.5 mL, 1.6 M, 13.6 mmol) was added followed by TMSCl (3.45 ml, 27.2 mmol) and stirred for 20 min. Amide **4** (1.00 g, 2.72 mmol) in anhydrous dichloromethane (20 mL) was added and the solution warmed to 0 °C and stirred for 9 h. The reaction was quenched with ammonium chloride and filtered. The organic layer was extracted with ethyl acetate and washed with sodium thiosulfate then brine before being dried with anhydrous sodium sulfate, filtered, and concentrated *in vacuo*. The crude was purified by column chromatography, eluting with 10% ethyl acetate in petrol, to yield title compound **S10** as a white solid (631 mg, 61%).

m.p. 92–94 °C

^{1}H NMR (500 MHz, CDCl$_3$) δ_H: 7.89 (d, *J* = 8.6 Hz, 1H), 7.78 (d, *J* = 8.1 Hz, 1H), 7.76 (d, *J* = 8.6 Hz, 1H), 7.47 (ddd, *J* = 8.4, 6.8, 1H, 1.4 Hz), 7.40 (ddd, *J* = 8.0, 6.8, 1.1 Hz, 1H), 7.39 (d, *J* = 8.6 Hz, 1H), 4.36 (hept, *J* = 6.7 Hz, 1H), 4.28 (d, *J* = 16.7 Hz, 1H), 4.19 (d, *J* = 16.8 Hz, 1H), 4.07 (qd, *J* = 7.1, 2.1 Hz, 2H), 3.63 (dp, *J* = 8.4, 6.7 Hz, 1H), 3.48 (hept, *J* = 6.8 Hz, 1H), 2.72 (dd, *J* = 15.5, 6.4 Hz, 1H), 2.61 (dd, *J* = 15.5, 8.4 Hz, 1H), 1.39 (d, *J* = 3.7 Hz, 3H), 1.38 (d, *J* = 3.7 Hz, 3H), 1.34 (d, *J* = 6.8 Hz, 3H), 1.34 (d, *J* = 6.6 Hz, 6H), 1.17 (t, *J* = 7.1 Hz, 3H).

^{13}C{^{1}H} NMR (126 MHz, CDCl$_3$) δ_C: 172.7, 169.1, 141.5, 133.3, 132.6, 129.2, 128.6, 127.8, 126.2, 124.9, 124.2, 123.7, 60.4, 48.9, 46.1, 42.7, 34.4, 32.3, 21.5, 21.2 21.1, 20.8, 20.8, 14.3.

FTIR (neat) v/cm^{-1} = 2967, 1731, 1643, 1446, 1371, 1446, 1371, 1330, 1176, 1154, 1040, 820, 745.

HRMS (*m/z*, ESI^{+}): calculated for $C_{24}H_{34}O_3N$ ([M+H]$^{+}$) = 384.2533, found = 384.2541.

3-((*Tert*-butyldimethylsilyl)oxy)-*N*,*N*-diisopropyl-1-methylphenanthrene-4-carboxamide, S12

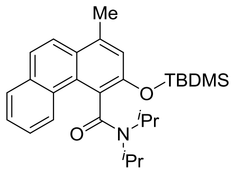

Prepared according to **General Procedure E** with amide **S10** (682 mg, 1.78 mmol) and KHMDS (7.1 mL, 0.5 M, 3.56 mmol) in anhydrous THF (5 mL), followed by TBDMSCl (536 mg, 3.56 mmol). Purification by column chromatography, eluting with 5% diethyl ether in petrol, yielded intermediate **S11** as a white solid (181 mg, 23%).

Then, according to **General Procedure F** with intermediate **S11** (200 mg, 0.458 mmol) and DDQ (135 mg, 0.595 mmol) in toluene (10 mL) for 30 min. Purification by column chromatography, eluting with 5–10% diethyl ether in petrol, yielded title compound **S12** as a white solid (158 mg, 36%, combined yield = 8%).

m.p. 166–167 °C

^{1}H NMR (400 MHz, $CDCl_3$) δ_H: 9.03 (d, *J* = 8.5 Hz, 1H), 7.86 (d, *J* = 9.0 Hz, 1H), 7.82 (d, *J* = 7.8 Hz, 1H), 7.64 (d, *J* = 9.0 Hz, 1H), 7.53 (t, *J* = 7.0 Hz, 1H), 7.47 (dd, *J* = 8.5, 6.9 Hz, 1H), 7.07 (s, 1H), 3.56 (hept, *J* = 6.6 Hz, 1H), 3.38 (hept, *J* = 6.7 Hz, 1H), 2.72 (s, 3H), 1.80 (d, *J* = 6.9 Hz, 3H), 1.72 (d, *J* = 6.8 Hz, 3H), 1.07 (s, 9H), 0.99 (d, *J* = 6.7 Hz, 3H), 0.54 (d, *J* = 6.7 Hz, 3H), 0.40 (s, 3H), 0.29 (s, 3H).

^{13}C{^{1}H} NMR (101 MHz, $CDCl_3$) δ_C: 170.3, 151.1, 135.9, 133.0, 129.9, 128.5, 128.3, 127.8, 126.6, 126.5, 125.5, 125.5, 124.0, 122.8, 121.7, 51.2, 46.2, 26.2, 21.8, 20.9, 20.8, 20.1, 19.3, 18.8, −3.4, −3.8.

FTIR (neat) ν/cm^{-1} = 2930, 1623, 1461, 1372, 1328, 1151, 834,730.

HRMS (*m/z*, ESI$^+$): calculated for $C_{28}H_{40}NO_2Si$ ($[M+H]^+$) = 450.2823, found = 450.2814.

3-Hydroxy-*N*,*N*-diisopropyl-1-methylphenanthrene-4-carboxamide, S13

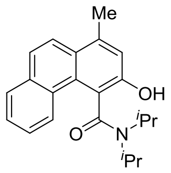

Prepared according to **General Procedure H** with silylated naphthol **S12** (213 mg, 0.490 mmol) and potassium fluoride (57 mg, 0.979 mmol) in methanol (5.0 mL). Purification by trituration with ethyl acetate and washing with hexane yielded title compound **S13** as a white solid (57 mg, 36%).

m.p. >200 °C

^{1}H NMR (500 MHz, $CDCl_3$) δ_H: 8.67–8.61 (1H, m), 7.86 (1H, d, *J* = 7.9 Hz), 7.84 (1H, d, *J* = 9.0 Hz), 7.64 (1H, d, *J* = 9.0 Hz), 7.56 (1H, dd, *J* = 8.0, 6.9 Hz), 7.52–7.44 (1H, m), 7.41 (1H, s), 7.05 (1H, s), 3.49 (1H, hept, *J* = 6.8 Hz), 3.30 (1H, hept, *J* = 6.6 Hz), 2.60 (3H, s), 1.77 (3H, d, *J* = 6.8 Hz), 1.68 (3H, d, *J* = 6.8, Hz), 0.82 (3H, d, *J* = 6.7 Hz), 0.39 (3H, d, *J* = 6.7 Hz).

^{13}C{^{1}H} NMR (126 MHz, $CDCl_3$) δ_C: 171.8, 153.0, 137.4, 133.1, 129.3, 128.4, 128.3, 127.7, 127.0, 125.6, 125.4, 124.8, 123.0, 119.4, 117.1, 51.4, 46.7, 21.3, 21.1, 20.2, 19.1, 19.0.

FTIR (neat) v/cm^{-1} = 2973, 1581, 1525, 1506, 1461, 1369, 1340, 1311, 1287, 1208, 1160, 1107, 1062, 1031, 908, 863, 816, 744.

HRMS (*m/z*, ESI$^+$): calculated for $C_{22}H_{26}O_2N$ ($[M+H]^+$) = 336.1958, found = 336.1956.

Scheme S4

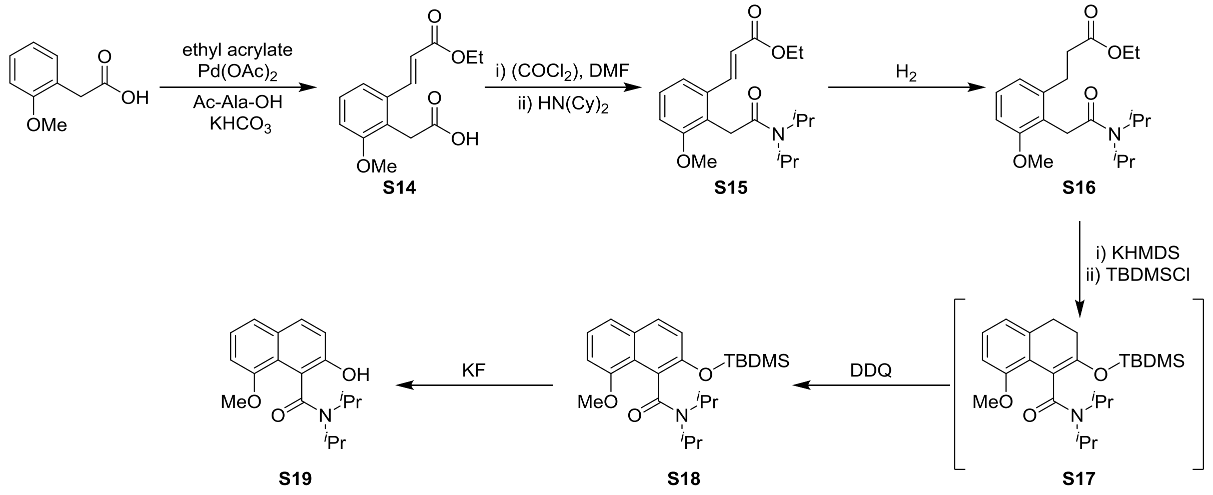

(*E*)-2-(2-(3-Ethoxy-3-oxoprop-1-en-1-yl)-6-methoxyphenyl)acetic acid, S14

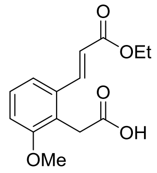

Prepared according to **General Procedure A** with 2-methoxyphenylacetic acid (4.70 g, 28.3 mmol), potassium bicarbonate (5.70 g, 56.6 mmol), (*S*)-*N*-acetylalanine (742 mg, 5.66 mmol), palladium acetate (635 mg, 2.83 mmol) and ethyl acrylate (6.2 mL, 56.6 mmol) in *tert*-amyl alcohol (50 mL). Purification by column chromatography, eluting with 40% ethyl acetate, 1% acetic acid in petrol, yielded title compound **S14** as a pale orange solid (6.44 g, 86%).

m.p. 96–98 °C

^{1}H NMR (500 MHz, $CDCl_3$) δ_H: 7.90 (d, *J* = 15.7 Hz, 1H), 7.27 (t, *J* = 8.0 Hz, 1H), 7.18 (d, *J* = 7.9 Hz, 1H), 6.91 (d, *J* = 8.2 Hz, 1H), 6.35 (d, *J* = 15.7 Hz, 1H), 4.26 (q, *J* = 7.2 Hz, 2H), 3.85 (s, 2H), 3.83 (s, 3H), 1.33 (t, *J* = 7.1 Hz, 3H).

^{13}C{^{1}H} NMR (126 MHz, $CDCl_3$) δ_C: 177.3, 166.9, 158.0, 141.7, 135.5, 128.7, 122.3, 121.5, 119.1, 111.7, 60.8, 55.9, 31.5, 14.4.

FTIR (neat) ν/cm^{-1} = 2981, 1706, 1634, 1578, 1473, 1369, 1258, 1178, 1092, 1032, 976, 789, 731.

HRMS (*m/z*, ESI$^+$): calculated for $C_{14}H_{16}NaO_5$ ([M+Na]$^+$) = 287.0890, found = 287.0888.

Ethyl (*E*)-3-(2-(2-(diisopropylamino)-2-oxoethyl)-3-methoxyphenyl)acrylate, S15

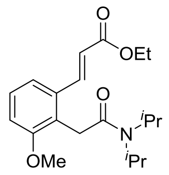

Prepared according to **General Procedure B** with acid **S14** (3.00 g, 11.4 mmol), oxalyl chloride (4.6 mL, 22.7 mmol) and DMF (0.5 mL) in anhydrous dichloromethane (20 mL) followed by diisopropylamine (2.3 ml, 22.7 mmol) in anhydrous dichloromethane (20 mL). Purification by column chromatography, eluting with 10–20% ethyl acetate in petrol, yielded title compound **S15** as a white solid (1.96 g, 50%).

m.p. 121–123 °C

^{1}H NMR (500 MHz, $CDCl_3$) δ_H: 7.84 (d, *J* = 15.8 Hz, 1H), 7.22 (t, *J* = 7.9 Hz, 1H), 7.19 (d, *J* = 7.9 Hz, 1H), 6.89 (d, *J* = 7.9 Hz, 1H), 6.33 (d, *J* = 15.8 Hz, 1H), 4.23 (q, *J* = 7.1 Hz, 2H), 4.21–4.09 (m, 1H), 3.81 (s, 3H), 3.80 (s, 2H), 3.51–3.47 (m, 1H), 1.37 (d, *J* = 6.8 Hz, 6H), 1.31 (t, *J* = 7.1 Hz, 3H), 1.27 (d, *J* = 6.7 Hz, 6H).

^{13}C{^{1}H} NMR (126 MHz, $CDCl_3$) δ_C: 169.2, 167.0, 157.7, 142.6, 135.7, 127.8, 125.4, 120.6, 119.1, 111.8, 60.5, 55.9, 48.7, 46.0, 32.2, 21.1, 20.8, 14.5.

FTIR (neat) v/cm^{-1} = 2967, 1710, 1636, 1578, 1473, 1441, 1369, 1333, 1256, 1177, 1072, 1041, 980, 795.

HRMS (m/z, ESI$^+$): calculated for $C_{20}H_{29}NNaO_4$ ($[M+Na]^+$) = 370.1989, found = 370.1989.

Ethyl 3-(2-(2-(diisopropylamino)-2-oxoethyl)-3-methoxyphenyl)propanoate, S16

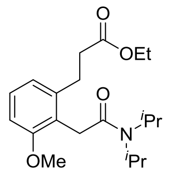

Prepared according to **General Procedure D** with acrylate **S15** (1.93 g, 5.55 mmol) and Pd/C (193 mg, 10% w/w) in methanol (30 mL) to yield title compound **S16** as a white solid without need for further purification (1.90 g, 99%).

m.p. 84–86 °C

^{1}H NMR (500 MHz, $CDCl_3$) δ_H: 7.14 (t, *J* = 8.0 Hz, 1H), 6.80 (d, *J* = 7.7 Hz, 1H), 6.74 (d, *J* = 8.2 Hz, 1H), 4.19 (hept, *J* = 6.7 Hz, 1H), 4.12 (q, *J* = 7.1 Hz, 2H), 3.77 (s, 3H), 3.69 (s, 2H), 3.50–3.44 (m, 1H), 2.97–2.87 (m, 2H), 2.62–2.53 (m, 2H), 1.35 (d, *J* = 6.9 Hz, 6H), 1.25 (d, *J* = 6.8 Hz, 6H), 1.23 (t, *J* = 7.1 Hz, 3H).

^{13}C{^{1}H} NMR (126 MHz, $CDCl_3$) δ_C: 173.3, 169.8, 157.7, 140.8, 127.5, 123.9, 121.3, 108.7, 60.4, 55.7, 48.6, 45.9, 35.4, 32.3, 28.4, 21.0, 20.8, 14.3.

FTIR (neat) ν/cm^{-1} = 2968, 1725, 1644, 1583, 1435, 1372, 1335, 1282, 1257, 1192, 1159, 1086, 1044, 1026, 800, 775, 758.

HRMS (m/z, ESI$^+$): calculated for $C_{20}H_{32}NO_4$ ([M+H]$^+$) = 350.2326, found = 350.2322.

2-((*Tert*-butyldimethylsilyl)oxy)-*N*,*N*-diisopropyl-8-methoxy-1-naphthamide, S18

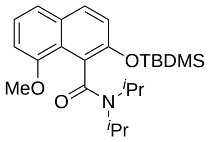

Prepared according to **General Procedure E** with amide **S16** (1.85 g, 5.29 mmol) and KHMDS (21.2 mL, 0.5 M, 10.6 mmol) in anhydrous THF (10 mL), followed by TBDMSCl (1.60 g, 10.6 mmol). Purification by column chromatography, eluting with 10% diethyl ether in petrol, yielded intermediate **S17** as a white solid (1.22 g, 55%).

Then, according to **General Procedure F** with intermediate **S17** (1.20 g, 2.87 mmol) and DDQ (486 mg, 2.21 mmol) in toluene (10 mL) for 30 min. Purification by column chromatography, eluting with 10% diethyl ether in petrol, yielded title compound **S18** as a white solid (403 mg, 34%, combined yield = 19%).

m.p. 159-162 °C

^{1}H NMR (500 MHz, $CDCl_3$) δ_H: 7.63 (d, *J* = 8.9 Hz, 1H), 7.34 (d, *J* = 8.2 Hz, 1H), 7.24 (t, *J* = 7.9 Hz, 1H), 7.07 (d, *J* = 8.9 Hz, 1H), 6.80 (d, *J* = 7.7 Hz, 1H), 3.88 (s, 3H), 3.54 (h, *J* = 6.7 Hz, 2H), 1.64 (d, *J* = 6.8 Hz, 6H), 1.07 (d, *J* = 6.6 Hz, 3H), 1.01 (s, 9H), 0.93 (d, *J* = 6.7 Hz, 3H), 0.33 (s, 3H), 0.21 (s, 3H).

^{13}C{^{1}H} NMR (126 MHz, $CDCl_3$) δ_C: 168.6, 155.3, 150.0, 130.8, 128.8, 124.1, 123.2, 122.7, 121.5, 120.8, 106.3, 55.4, 50.9, 45.7, 26.2, 21.7, 21.1, 20.4, 19.9, 18.8, −3.4, −3.8.

FTIR (neat) ν/cm^{-1} = 2929, 1630, 1461, 1313, 1255, 989, 830, 779.

HRMS (*m/z*, ESI$^+$): calculated for $C_{24}H_{38}NO_3Si$ ([M+H]$^+$) = 416.2616, found = 416.2624.

2-Hydroxy-*N*,*N*-diisopropyl-8-methoxy-1-naphthamide, S19

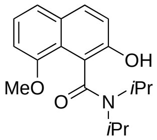

Prepared according to **General Procedure H** with silylated naphthol **S18** (380 mg 0.916 mmol) and potassium fluoride (100 mg, 1.83 mmol) in methanol (5.0 mL). Purification by trituration with ethyl acetate and washing with hexane yielded title compound **S19** as a white solid (120 mg, 44%).

m.p. > 230 °C

^{1}H NMR (700 MHz, $CDCl_3$) δ_H: 7.51 (s, 1H), 7.39 (d, *J* = 8.7 Hz, 1H), 7.30 (d, *J* = 8.1 Hz, 1H), 7.21 (t, *J* = 7.9 Hz, 1H), 6.89 (d, *J* = 8.8 Hz, 1H), 6.79 (d, *J* = 7.5 Hz, 1H), 3.90 (s, 3H), 3.65 (hept, *J* = 6.7 Hz, 1H), 3.53 (hept, *J* = 6.9 Hz, 1H), 1.66 (d, *J* = 6.8 Hz, 3H), 1.60 (d, *J* = 6.8 Hz, 3H), 0.99 (d, *J* = 6.8 Hz, 6H).

^{13}C{^{1}H} NMR (176 MHz, $CDCl_3$) δ_C: 170.7, 154.6, 151.6, 130.0, 129.5, 123.5, 122.6, 121.0, 119.6, 116.9, 105.8, 55.3, 51.4, 46.1, 20.8, 20.7, 20.6, 19.6.

FTIR (neat) ν/cm^{-1} = 2977, 1575, 1516, 1430, 1366, 1340, 1215, 1215, 1115, 1050, 1030, 821, 738.

HRMS (*m/z*, ESI$^+$): calculated for $C_{18}H_{24}O_3N$ ([M+H]$^+$) = 302.1751, found = 302.1751.

Scheme S5

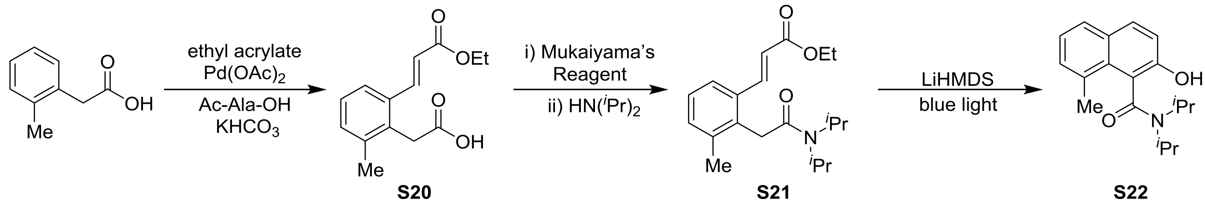

(*E*)-2-(2-(3-Ethoxy-3-oxoprop-1-en-1-yl)-6-methylphenyl)acetic acid, S20

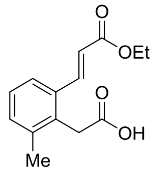

Prepared according to **General Procedure A** with 2-methylphenylacetic acid (2.00 g, 13.0 mmol), potassium bicarbonate (5.60 g, 56.0 mmol), (*S*)-*N*-acetylalanine (734 mg, 5.60 mmol), palladium acetate (629 mg, 2.80 mmol) and ethyl acrylate (2.5 mL, 26.0 mmol) in *tert*-amyl alcohol (50 mL). Purification by column chromatography, eluting with ethyl acetate, yielded title compound **S20** as a beige solid (1.70 g, 54%).

m.p. 124–125 °C

^{1}H NMR (500 MHz, $CDCl_3$) δ_H: 8.00 (d, *J* = 15.7 Hz, 1H), 7.42 (dd, *J* = 6.9, 2.2 Hz, 1H), 7.27–7.17 (m, 2H), 6.33 (d, *J* = 15.7 Hz, 1H), 4.26 (q, *J* = 7.2 Hz, 2H), 3.84 (s, 2H), 2.36 (s, 3H), 1.33 (t, *J* = 7.1 Hz, 3H).

$^{13}C\{^1H\}$ NMR (126 MHz, $CDCl_3$) δ_C: 176.5, 167.0, 142.6, 138.2, 134.9, 132.1, 131.6, 127.9, 125.1, 121.3, 60.8, 34.8, 20.4, 14.4.

FTIR (neat) ν/cm^{-1} = 3071, 2987, 2942, 2873, 1706, 1690, 1633, 1590, 1472.

HRMS (m/z, ESI$^+$): calculated for $C_{14}H_{16}NaO_4$ ($[M+Na]^+$) = 271.0940, found = 271.0941.

Ethyl (*E*)-3-(2-(2-(diisopropylamino)-2-oxoethyl)-3-methylphenyl)acrylate, S21

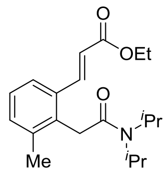

Prepared according to **General Procedure C** with **S20** (218 mg, 0.880 mmol), 2-chloro-1-methylpyridinium iodide (337 mg, 1.32 mmol) and diisopropylamine (0.23 mL, 2.64 mmol) in dichloromethane (6 mL). Purification by column chromatography, eluting with 40–100% ethyl acetate in petrol, yielded title compound **S21** as an off-white solid (216 mg, 74%).

m.p. 112–115 °C

^{1}H NMR (400 MHz, $CDCl_3$) δ_H: 7.85 (d, *J* = 15.7 Hz, 1H), 7.39 (dd, *J* = 7.4, 1.6 Hz, 1H), 7.23–7.11 (m, 2H), 6.29 (d, *J* = 15.7 Hz, 1H), 4.23 (q, *J* = 7.1 Hz, 2H), 4.16 (hept, *J* = 7.6 Hz, 1H), 3.74 (s, 2H), 3.47 (hept, *J* = 7.5 Hz, 1H), 2.29 (s, 3H), 1.39 (d, *J* = 6.8 Hz, 6H), 1.35–1.27 (m, 9H).

^{13}C{^{1}H} NMR (101 MHz, $CDCl_3$) δ_C: 168.2, 167.0, 143.3, 137.8, 134.8, 134.7, 131.9, 127.0, 124.9, 120.5, 60.5, 48.8, 46.2, 35.7, 21.1, 20.8, 20.4, 14.5.

FTIR (neat) ν/cm^{-1} = 2969, 2870, 2161, 2032, 1798, 1594,1476, 1460, 1444.

HRMS (m/z, ESI$^+$): calculated for $C_{20}H_{29}NNaO_3$ ([M+Na]$^+$) = 354.2044, found = 354.2040.

2-Hydroxy-*N*,*N*-diisopropyl-8-methyl-1-naphthamide, S22

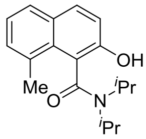

Prepared according to **General Procedure J** with acrylate **S21** (99 mg, 0.300 mmol) and LiHMDS (0.630 mL, 0.630 mmol) in THF (6.0 mL). Purification by column chromatography, eluting with 30–50% ethyl acetate in petrol, yielded title compound **S22** as a white solid (58 mg, 68%).

m.p. 204–206 °C

^{1}H NMR (500 MHz, $CDCl_3$) δ_H: 8.63 (s, 1H), 7.51 (dd, *J* = 8.0, 1.5 Hz, 1H), 7.29 (d, *J* = 8.9 Hz, 1H), 7.21 (dt, *J* = 7.0, 1.3 Hz, 1H), 7.15 (dd, *J* = 8.0, 7.0 Hz, 1H), 6.73 (d, *J* = 8.8 Hz, 1H), 3.69 (hept, *J* = 6.6 Hz, 1H), 3.50 (hept, *J* = 6.8 Hz, 1H), 2.70 (s, 3H), 1.61 (d, *J* = 6.8 Hz, 3H), 1.49 (d, *J* = 6.8 Hz, 3H), 1.01 (d, *J* = 6.6 Hz, 3H), 0.93 (d, *J* = 6.5 Hz, 3H).

^{13}C{^{1}H} NMR (126 MHz, $CDCl_3$) δ_C: 172.3, 152.8, 132.8, 130.7, 130.0, 129.4, 129.3, 127.2, 122.7, 118.8, 118.2, 51.7, 46.4, 22.1, 20.4, 19.7, 19.6.

FTIR (neat) ν/cm^{-1} = 3032, 2969, 2869, 2161, 2032, 1978, 1594, 1575, 1460, 1444, 1434.

HRMS (m/z, ESI$^+$): calculated for $C_{18}H_{23}NNaO_2$ ($[M+Na]^+$) = 308.1619, found = 308.1621.

Scheme S6

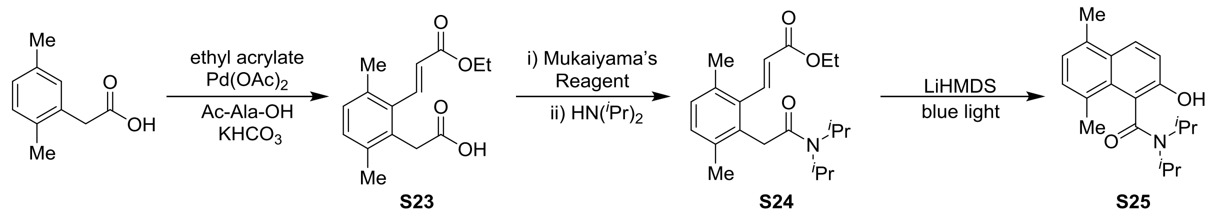

(*E*)-2-(2-(3-ethoxy-3-oxoprop-1-en-1-yl)-3,6-dimethylphenyl)acetic acid, S23

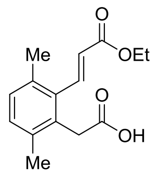

Prepared according to **General Procedure A** with (2,5-dimethylphenyl)acetic acid (2.00 g, 12.0 mmol), potassium bicarbonate (2.40 g, 24.0 mmol), (*S*)-*N*-acetylalanine (315 mg, 24.0 mmol), palladium acetate (269 mg, 1.20 mmol) and ethyl acrylate (2.4 mL, 24.0 mmol) in *tert*-amyl alcohol (50 mL). Purification by column chromatography, eluting with ethyl acetate, yielded title compound **S23** as a yellow oil (2.99 g, 95%).

^{1}H NMR (400 MHz, $CDCl_3$) δ_H: 7.88 (d, *J* = 16.3 Hz, 1H), 7.23–7.05 (m, 2H), 6.07 (d, *J* = 16.3 Hz, 1H), 4.33 (q, *J* = 7.2 Hz, 2H), 3.80 (s, 2H), 2.35 (s, 3H), 2.32 (s, 3H), 1.39 (t, *J* = 7.1 Hz, 3H).

^{13}C{^{1}H} NMR (101 MHz, $CDCl_3$) δ_C: 177.3, 166.5, 144.0, 135.8, 135.2, 134.0, 130.2, 130.2, 129.4, 125.3, 60.8, 36.2, 20.8, 20.0, 14.3.

FTIR (neat) ν/cm^{-1} = 3166, 2982, 2935, 2875, 1736, 1713, 1642, 1464.

HRMS (m/z, ESI$^+$): calculated for $C_{15}H_{18}O_4Na$ ($[M+Na]^+$) = 285.1097, found = 285.1100.

Ethyl (*E*)-3-(2-(2-(diisopropylamino)-2-oxoethyl)-3,6-dimethylphenyl)acrylate, S24

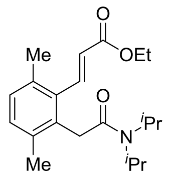

Prepared according to **General Procedure C** with **S23** (231 mg, 0.880 mmol), 2-chloro-1-methylpyridinium iodide (337 mg, 1.32 mmol) and diisopropylamine (0.36 mL, 2.64 mmol) in dichloromethane (6 mL). Purification by column chromatography, eluting with 20–40% ethyl acetate in petrol, yielded title compound **S24** as an off-white solid (258 mg, 85%).

m.p. 115–117 °C

^{1}H NMR (400 MHz, $CDCl_3$) δ_H: 7.73 (d, *J* = 16.3 Hz, 1H), 7.00 (d, *J* = 7.7 Hz, 1H), 6.95 (d, *J* = 7.8 Hz, 1H), 5.94 (d, *J* = 16.3 Hz, 1H), 4.20 (q, *J* = 7.1 Hz, 2H), 4.12–3.99 (m, 1H), 3.54 (s, 2H), 3.45–3.33 (m, 1H), 2.21 (s, 3H), 2.18 (s, 3H), 1.38 (d, *J* = 6.8 Hz, 6H), 1.26 (t, *J* = 7.1 Hz, 3H), 1.19 (d, *J* = 6.7 Hz, 6H).

$^{13}C\{^{1}H\}$ NMR (101 MHz, $CDCl_3$) δ_C: 168.3, 166.2, 144.0, 135.0, 134.9, 133.4, 132.8, 129.8, 128.2, 124.1, 60.1, 48.3, 45.7, 36.9, 20.6, 20.3, 19.7, 14.1.

FTIR (neat) ν/cm^{-1} = 2970, 2923, 2161, 2031, 1978, 1710, 1644, 1439.

HRMS (m/z, ESI^{+}): calculated for $C_{21}H_{31}NNaO_3$ ([M+Na]$^{+}$) = 368.2201, found = 368.2202.

2-Hydroxy-*N*,*N*-diisopropyl-5,8-dimethyl-1-naphthamide, S25

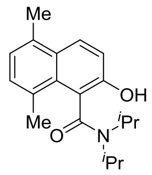

Prepared according to **General Procedure J** with acrylate **S24** (104 mg, 0.300 mmol) and LiHMDS (0.630 mL, 0.630 mmol) in THF (6.0 mL). Purification by column chromatography, eluting with 30–50% ethyl acetate in petrol, yielded title compound **S25** as an off-white solid (67 mg, 75%).

m.p. 224 °C (dec.)

^{1}H NMR (500 MHz, $CDCl_3$) δ_H: 7.86 (s, 1H), 7.51 (d, *J* = 9.1 Hz, 1H), 7.13 (d, *J* = 7.1 Hz, 1H), 7.04 (d, *J* = 7.1 Hz, 1H), 6.80 (d, *J* = 9.1 Hz, 1H), 3.61 (hept, *J* = 6.7 Hz, 1H), 3.54 (hept, *J* = 6.8 Hz, 1H), 2.69 (s, 3H), 2.56 (s, 3H), 1.62 (d, *J* = 6.9 Hz, 3H), 1.57 (d, *J* = 6.7 Hz, 3H), 1.02 (d, *J* = 6.7 Hz, 3H), 0.98 (d, *J* = 6.6 Hz, 3H).

^{13}C{^{1}H} NMR (126 MHz, $CDCl_3$) δ_C: 172.1, 152.1, 132.7, 131.1, 130.2, 129.3, 128.4, 126.6, 124.3, 119.3, 118.3, 51.6, 46.5, 22.1, 20.6, 20.6, 20.1, 19.7, 19.5.

FTIR (neat) ν/cm^{-1} = 3161, 2929, 2868, 2160, 2031, 1977, 1586, 1523, 1440, 1409.

HRMS (m/z, ESI$^+$): calculated for $C_{19}H_{25}NNaO_2$ ([M+Na]$^+$) = 322.1783, found = 322.1785.

Scheme S7

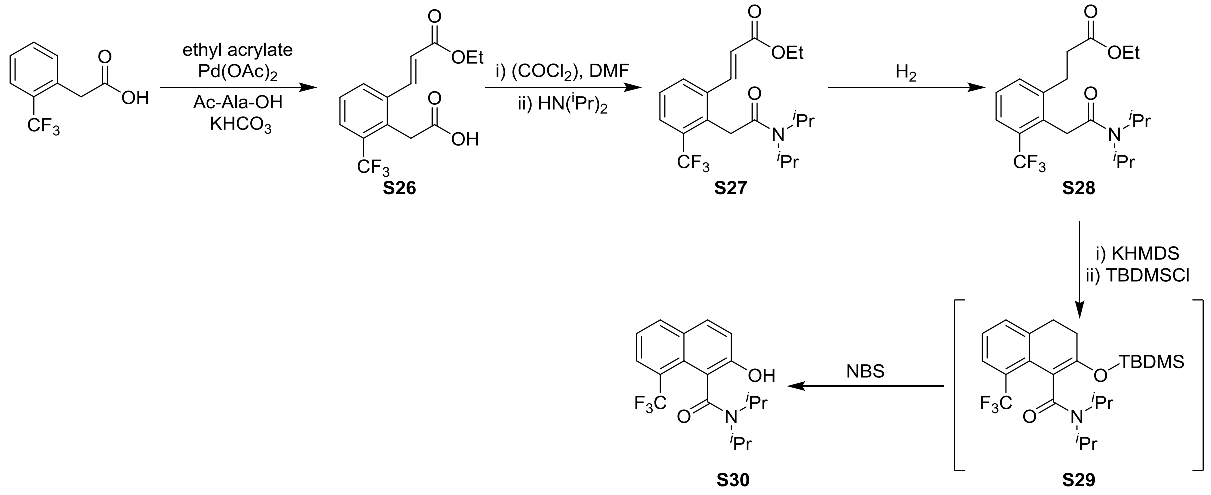

(*E*)-2-(2-(3-Ethoxy-3-oxoprop-1-en-1-yl)-6-(trifluoromethyl)phenyl)acetic acid, S26

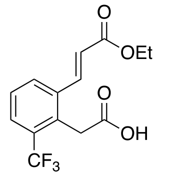

Prepared according to **General Procedure A** with 2-(trifluoromethyl)phenylacetic acid (1.00 g, 4.90 mmol), potassium bicarbonate (981 mg, 9.80 mmol), (*S*)-*N*-acetylalanine (64 mg, 0.490 mmol), palladium acetate (55 mg, 0.245 mmol) and ethyl acrylate (1.1 mL, 9.80 mmol) in *tert*-amyl alcohol (20 mL). Purification by column chromatography, eluting with 30% ethyl acetate, 1% acetic acid in petrol, yielded title compound **S26** as a beige solid (1.34 g, 92%).

m.p. 122–125 °C

^{1}H NMR (500 MHz, $CDCl_3$) δ_H: 7.90 (d, *J* = 15.7 Hz, 1H), 7.74 (d, *J* = 7.9 Hz, 1H), 7.71 (d, *J* = 7.8, 1.2 Hz, 1H), 7.44 (t, *J* = 7.9 Hz, 1H), 6.37 (d, *J* = 15.7 Hz, 1H), 4.27 (q, *J* = 7.1 Hz, 2H), 4.01 (s, 2H), 1.33 (t, *J* = 7.1 Hz 3H).

^{13}C{^{1}H} NMR (126 MHz, $CDCl_3$) δ_C: 174.2, 166.4, 140.7, 137.4, 131.1, 130.8, 130.4 (q, *J* = 29.7 Hz), 128.2, 127.4 (q, *J* = 5.8 Hz), 124.2 (q, *J* = 274.2 Hz), 123.4, 61.1, 34.2, 14.4.

^{19}F NMR (470 MHz, $CDCl_3$) δ_F: –59.7.

FTIR (neat) ν/cm^{-1} = 2987, 1715, 1455, 1396, 1325, 1161, 1121, 806.

HRMS (*m/z*, ESI$^+$): calculated for $C_{14}H_{13}FO_4$ ([M+H]$^+$) = 325.0658, found = 325.0660.

Ethyl (*E*)-3-(2-(2-(diisopropylamino)-2-oxoethyl)-3-(trifluoromethyl)phenyl)acrylate, S27

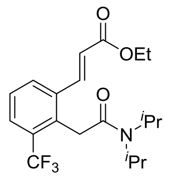

Prepared according to **General Procedure B** with acid **S26** (3.50 g, 11.6 mmol), oxalyl chloride (4.7 mL, 23.2 mmol) and DMF (500 µL) in anhydrous dichloromethane (20 mL) followed by diisopropylamine (3.00 mL, 23.2 mmol) in anhydrous dichloromethane (20 mL). Purification by column chromatography, eluting with 10% ethyl acetate in petrol, yielded title compound **S27** as a white solid (2.87 g, 64%).

m.p. 101–103 °C

^{1}H NMR (400 MHz, $CDCl_3$) δ_H: 7.80 (d, *J* = 15.7 Hz, 1H), 7.71 (d, *J* = 7.8 Hz, 1H), 7.67 (d, *J* = 7.9 Hz, 1H), 7.37 (t, *J* = 7.9 Hz, 1H), 6.34 (d, *J* = 15.8 Hz, 1.4, 1H), 4.24 (q, *J* = 7.2 Hz, 2H), 4.12 (hept, *J* = 7.0 Hz, 1H), 3.89 (s, 2H), 3.46 (hept, *J* = 6.6 Hz, 1H), 1.36 (d, *J* = 6.7 Hz, 6H), 1.34–1.27 (m, 9H).

^{13}C{^{1}H} NMR (126 MHz, $CDCl_3$) δ_C: 167.1, 166.5, 141.7, 137.4, 134.4, 130.4, 130.1 (q, *J* = 29.7 Hz), 127.2, 127.2 (q, *J* = 5.8 Hz), 124.7 (q, *J* = 274.2 Hz), 122.3, 60.7, 49.0, 46.2, 35.4, 21.0, 20.6, 14.4.

^{19}F NMR (377 MHz, $CDCl_3$) δ_F: –59.4.

FTIR (neat) ν/cm^{-1} = 2980, 1717, 1647, 1454, 1371, 1320, 1276, 1155, 1114, 1042, 956, 807.

HRMS (*m/z*, ESI$^+$): calculated for $C_{20}H_{26}F_3NNaO_3$ ([M+Na]$^+$) = 408.1757, found = 408.1751.

Ethyl 3-(2-(2-(diisopropylamino)-2-oxoethyl)-3-(trifluoromethyl)phenyl)propanoate, S28

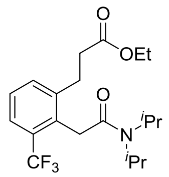

Prepared according to **General Procedure D** with acrylate **S27** (2.84 g, 7.37 mmol) and Pd/C (284 mg, 10% w/w) in methanol (20 mL), to yield title compound **S28** as a white solid without need for further purification (2.83 g, 99%).

m.p. 60–62 °C

^{1}H NMR (500 MHz, $CDCl_3$) δ_H: 7.52 (d, J = 7.7 Hz, 1H), 7.39 (d, J = 7.6 Hz, 1H), 7.29 (t, J = 7.8, 1H), 4.20–4.12 (m, 1H), 4.12 (q, J = 7.1 Hz, 2H), 3.86 (s, 2H), 3.44 (hept, J = 6.3 Hz, 1H), 2.96–2.89 (m, 2H), 2.68–2.60 (m, 2H), 1.35 (d, J = 6.8 Hz, 6H), 1.30 (d, J = 6.7 Hz, 6H), 1.23 (t, J = 7.1, 3H).

^{13}C{^{1}H} NMR (126 MHz, $CDCl_3$) δ_C: 173.1, 167.8, 142.2, 133.4, 132.5, 129.6 (q, J = 28.8 Hz), 127.0, 124.8 (q, J = 274.2 Hz), 124.3 (q, J = 5.9 Hz), 60.6, 48.9, 46.1, 35.1, 34.9, 27.9, 20.9, 20.6, 14.3.

^{19}F NMR (377 MHz, $CDCl_3$) δ –59.40.

FTIR (neat) ν/cm^{-1} = 2978, 1728, 1644, 1317, 1158, 1117, 907, 727.

HRMS (*m/z*, ESI$^+$): calculated for $C_{20}H_{29}F_3NO_3$ ([M+H]$^+$) = 388.2094, found = 388.2089.

2-Hydroxy-*N*,*N*-diisopropyl-8-(trifluoromethyl)-1-naphthamide, S30

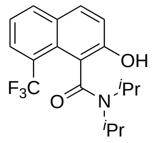

Prepared according to **General Procedure E** with **28** (2.79 g, 7.20 mmol) and KHMDS (29 mL, 0.5 M, 14.4 mmol) in anhydrous THF (30 mL) then TBDMSCl (2.17 g, 14.4 mmol). Purification by column chromatography, eluting with 5–10% diethyl ether in petrol, yielded intermediate **29** as a white solid (1.53 g, 47%)

Then, according to **General Procedure G** with **29** (1.10 g, 2.41 mmol) and *N*-bromosuccinamide (516 mg, 2.90 mmol) in benzene (20 mL). Purification by column chromatography, eluting with 20% ethyl acetate in petrol, yielded title compound **30** as a white solid (496 mg, 61%, combined yield = 29%).

m.p. 195–197 °C

^{1}H NMR (500 MHz, $CDCl_3$) δ_H: 8.34 (s, 1H), 7.94 (d, *J* = 7.5 Hz, 1H), 7.88 (d, *J* = 8.2 Hz, 1H), 7.52 (d, *J* = 8.8 Hz, 1H), 7.36 (t, *J* = 7.7 Hz, 1H), 7.07 (d, *J* = 8.8 Hz, 1H), 3.49 (hept, *J* = 6.8 Hz, 1H), 3.30 (hept, *J* = 6.6 Hz, 1H), 1.58 (d, *J* = 6.9 Hz, 3H), 1.52 (d, *J* = 6.7 Hz, 3H), 0.97 (d, *J* = 6.6 Hz, 3H), 0.84 (d, *J* = 6.6 Hz, 3H).

^{13}C{^{1}H} NMR (126 MHz, $CDCl_3$) δ_C: 169.4, 155.0, 134.1, 131.9, 129.7, 128.1 (q, *J* = 7.0 Hz), 126.4, 124.9 (d, *J* = 272.8 Hz), 123.6 (q, *J* = 30.8 Hz), 121.7, 119.9, 117.2, 51.4, 46.7, 20.7, 20.6, 20.0, 19.7.

^{19}F NMR (470 MHz, $CDCl_3$) δ_F: −55.2.

FTIR (neat) ν/cm^{-1} = 2918, 2850, 1579, 1515, 1439, 1382, 1300, 1130, 1054, 839, 737.

HRMS (*m/z*, ESI$^+$): calculated for $C_{18}H_{21}O_2NF_3$ ([M+H]$^+$) = 340.1519, found = 340.1517.

Scheme S8

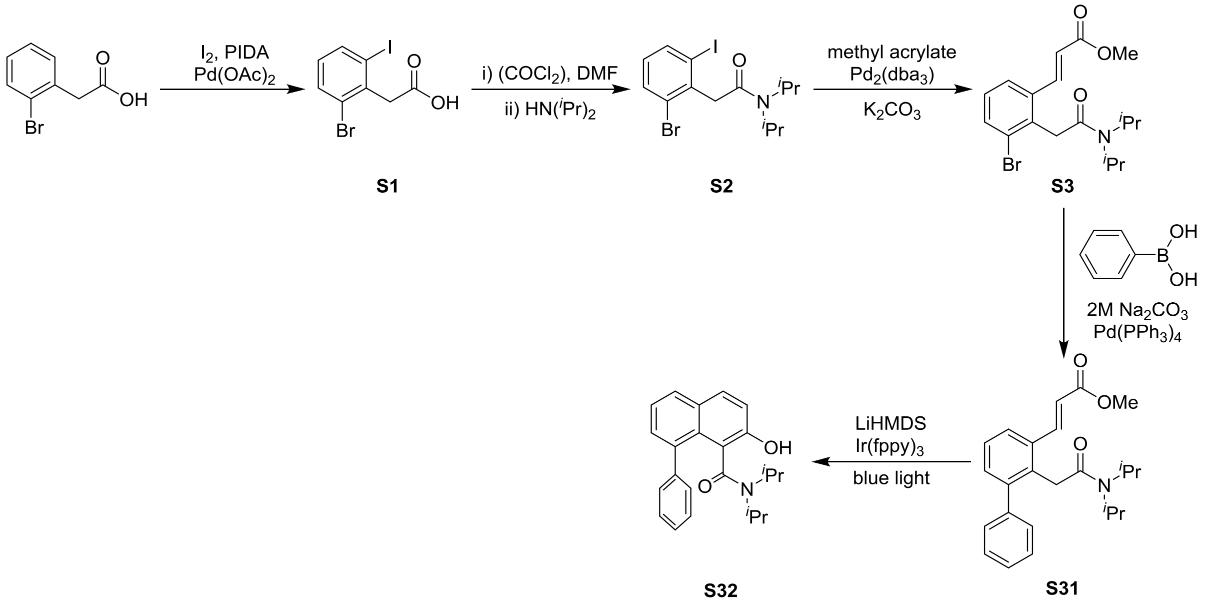

Methyl (*E*)-3-(2-(2-(diisopropylamino)-2-oxoethyl)-[1,1'-biphenyl]-3-yl)acrylate, S31

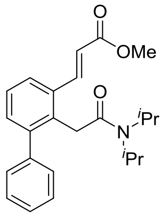

Prepared according to **General Procedure I** with **S3** (80 mg, 0.209 mmol), phenylboronic acid (28 mg, 0.230 mmol), sodium carbonate (67 mg, 0.627 mmol), palladium acetate (2.4 mg, 0.0100 mmol) and TBAB (51 mg, 0.209 mmol). Purification by column chromatography, eluting with 10–15% ethyl acetate in petrol, yielded title compound **S31** as a white solid (61 mg, 77%).

m.p. 120–123 °C

^{1}H NMR (500 MHz, $CDCl_3$) δ_H: 7.87 (d, *J* = 15.7 Hz, 1H), 7.59 (dd, *J* = 7.6 , 1.6 Hz, 1H), 7.40–7.32 (m, 3H), 7.32–7.29 (m, 3H), 7.28 (dd, *J* = 7.6, 1.7 Hz, 1H), 6.38 (d, *J* = 15.8, 1H), 3.84 (hept, *J* = 6.7 Hz, 1H), 3.78 (s, 3H), 3.61 (s, 2H), 3.39 (hept, *J* = 6.2, 1H), 1.39 (d, *J* = 6.7 Hz, 6H), 1.07 (d, *J* = 6.6 Hz, 6H).

^{13}C NMR (126 MHz, $CDCl_3$) δ_C: 168.8, 167.5, 143.6, 141.7, 135.3, 133.8, 131.6, 129.3, 128.3, 127.3, 126.9, 126.1, 120.1, 51.7, 48.6, 46.1, 37.1, 20.8, 20.6.

FTIR (neat) ν_{max}/cm^{-1} = 3059, 2999, 2066, 1719, 1637, 1440, 1334, 1274, 1166, 1042, 763, 705.

HRMS (ESI$^+$) calculated for $C_{24}H_{29}NO_3Na^+$ = 402.2040 ([M+Na]$^+$), found = 402.2041.

2-Hydroxy-*N*,*N*-diisopropyl-8-phenyl-1-naphthamide, S32

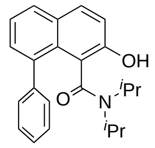

Prepared according to **General Procedure J** with acrylate **S31** (163 mg, 0.430 mmol), LiHMDS (0.9 mL, 0.902 mmol) and Ir(fppy)$_3$ (3.3 mg, 0.00430 mmol) in THF (4.9 mL). Purification by trituration with ethyl acetate yielded title compound **S32** as a white solid (100 mg, 67%).

m.p. 214–217 °C

^{1}H NMR (500 MHz, CDCl$_3$) δ_H: 7.80 (d, *J* = 8.8 Hz, 1H), 7.78–7.72 (m, 1H), 7.63–7.57 (m, 2H), 7.51 (s, 1H), 7.43–7.34 (m, 3H), 7.35–7.28 (m, 2H), 7.25 (d, *J* = 8.9 Hz, 1H), 3.23 (hept, *J* = 6.7 Hz, 1H), 3.07 (hept, *J* = 6.8 Hz, 1H), 1.39 (d, *J* = 6.7 Hz, 3H), 0.86 (d, *J* = 6.6 Hz, 3H), 0.62 (d, *J* = 6.7 Hz, 3H), 0.58 (d, *J* = 6.9 Hz, 3H).

^{13}C{^{1}H} NMR (126 MHz, CDCl$_3$) δ_C: 170.0, 154.0, 140.9, 138.2, 132.3 (br), 131.9, 131.5, 129.5, 128.9 (br), 128.4, 128.4, 128.3 (br), 127.9 (br), 127.7, 123.6, 118.8, 117.2, 50.7, 45.9, 21.3, 21.0, 19.5, 18.9.

FTIR (neat) ν/cm^{-1} = 3100, 3051, 2929, 2850, 1575, 1511, 1443, 1365, 1336, 1292, 833, 760, 700.

HRMS (m/z, ESI$^+$): calculated for $C_{23}H_{26}NO_2$ ([M+H]$^+$) = 348.1958, found = 348.1959.

Scheme S9

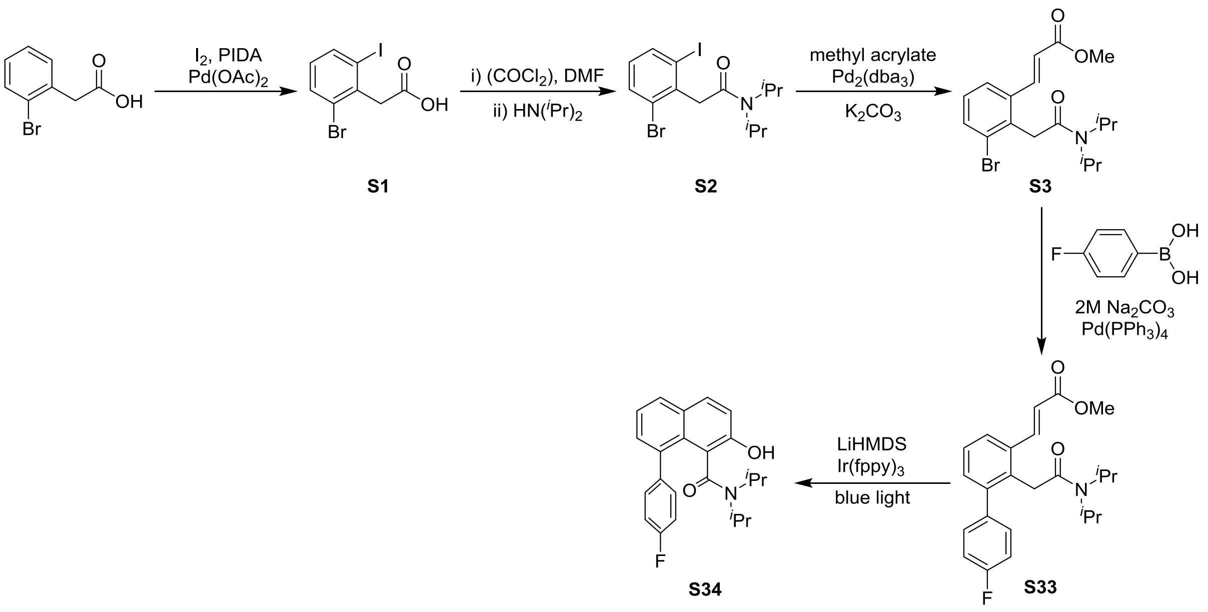

Ethyl (*E*)-3-(2-(2-(diisopropylamino)-2-oxoethyl)-4'-fluoro-[1,1'-biphenyl]-3-yl)acrylate, S33

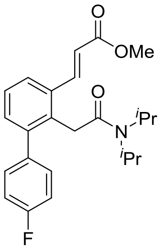

Prepared according to **General Procedure I** with **S3** (100 mg, 0.262 mmol), *p*-fluorophenylboronic acid (41 mg, 0.288 mmol), sodium carbonate (83 mg, 0.785 mmol), palladium acetate (2.9 mg, 0.0130 mmol) and TBAB (63 mg, 0.262 mmol). Purification by column chromatography, eluting with 10–15% ethyl acetate in petrol, yielded title compound **S33** as a white solid (96 mg, 93%).

m.p. 136–138 °C

^{1}H NMR (500 MHz, $CDCl_3$) δ_H = 7.85 (d, *J* = 15.8 Hz, 1H), 7.58 (dd, *J* = 7.7, 1.4 Hz, 1H), 7.34–7.27 (m, 3H), 7.25 (dd, *J* = 7.7, 1.4 Hz, 1H), 7.07 (t, *J* = 8.7 Hz, 2H), 6.38 (d, *J* = 15.7 Hz, 1H), 3.87 (hept, *J* = 6.7 Hz, 1H), 3.78 (s, 3H), 3.58 (s, 2H), 3.48–3.38 (m, 1H), 1.39 (d, *J* = 6.8 Hz, 6H), 1.11 (d, *J* = 6.7 Hz, 6H).

^{13}C NMR (126 MHz, $CDCl_3$) δ_C 168.7, 167.4, 162.4 (d, *J* = 246.2 Hz), 143.3, 142.6, 137.6 (d, *J* = 3.3 Hz), 135.3, 133.9, 131.6, 131.0 (d, *J* = 7.9 Hz), 127.0, 126.3, 120.4, 115.1 (d, *J* = 21.1 Hz), 51.8, 48.7, 46.1, 37.1, 20.9, 20.6.

^{19}F NMR (471 MHz, $CDCl_3$) δ_F = –115.4 (tt, *J* = 8.8, 5.5 Hz).

FTIR (neat) ν_{max}/cm^{-1} = 2980, 2971, 2889, 1718, 1634, 1611, 1523, 1444, 1274, 1166, 823, 782, 728.

HRMS (ESI$^+$) calculated for $C_{24}H_{28}FNO_3Na$ ($[M+Na]^+$) = 420.1945, found = 420.1943.

8-(4-Fluorophenyl)-2-hydroxy-*N*,*N*-diisopropyl-1-naphthamide, S34

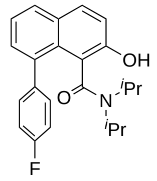

Prepared according to **General Procedure I** with acrylate **S33** (95 mg, 0.239 mmol), LiHMDS (0.5 mL, 0.500 mmol) and Ir(fppy)$_3$ (1.8 mg, 0.0024 mmol) in THF (4.8 mL). Purification by column chromatography, eluting with 10% diethyl ether in dichloromethane, yielded title compound **S34** as a white solid (52 mg, 59%).

m.p. 224–226 °C

^{1}H NMR (500 MHz, CDCl$_3$) $\delta_{H:}$ 7.80 (d, *J* = 8.8 Hz, 1H), 7.76 (dd, *J* = 7.9, 1.6 Hz, 1H), 7.55 (brs, 1H), 7.48 (brs, 1H), 7.44 (s, 1H), 7.37 (t, *J* = 7.5 Hz, 1H), 7.34 (dd, *J* = 7.1, 1.5 Hz, 1H), 7.26 (d, *J* = 8.8 Hz, 1H), 7.11 (brs, 1H), 7.04 (brs, 1H), 3.20 (hept, *J* = 6.7 Hz, 1H), 3.11 (hept, *J* = 6.9 Hz, 1H), 1.41 (d, *J* = 6.7 Hz, 3H), 0.85 (d, *J* = 6.7 Hz, 3H), 0.70 (d, *J* = 6.9 Hz, 3H), 0.64 (d, *J* = 6.7 Hz, 3H).

^{13}C NMR (126 MHz, CDCl$_3$) δ_H: 170.0, 162.9 (d, *J* = 246.6 Hz), 153.9, 137.1, 136.9 (d, *J* = 3.3 Hz), 134.0 (br), 131.8, 131.5, 130.3 (br), 129.5, 128.6, 128.3, 123.6, 118.9, 117.2, 115.1 (br), 114.8 (br), 50.7, 46.0, 21.2, 21.0, 19.5, 19.0.

^{19}F NMR (376 MHz, CDCl$_3$) δ_F = −115.6 (tt, *J* = 8.7, 5.4 Hz).

FTIR (neat) ν_{max}/cm^{-1} = 3052, 2973, 2930, 1602, 1575, 1506, 1382, 1338, 1157, 828, 726.

HRMS (*m/z,* ESI$^+$): calculated for $C_{23}H_{25}FNO_2$ ([M+H])$^+$ = 366.1863, mass found = 366.1865.

Scheme S10

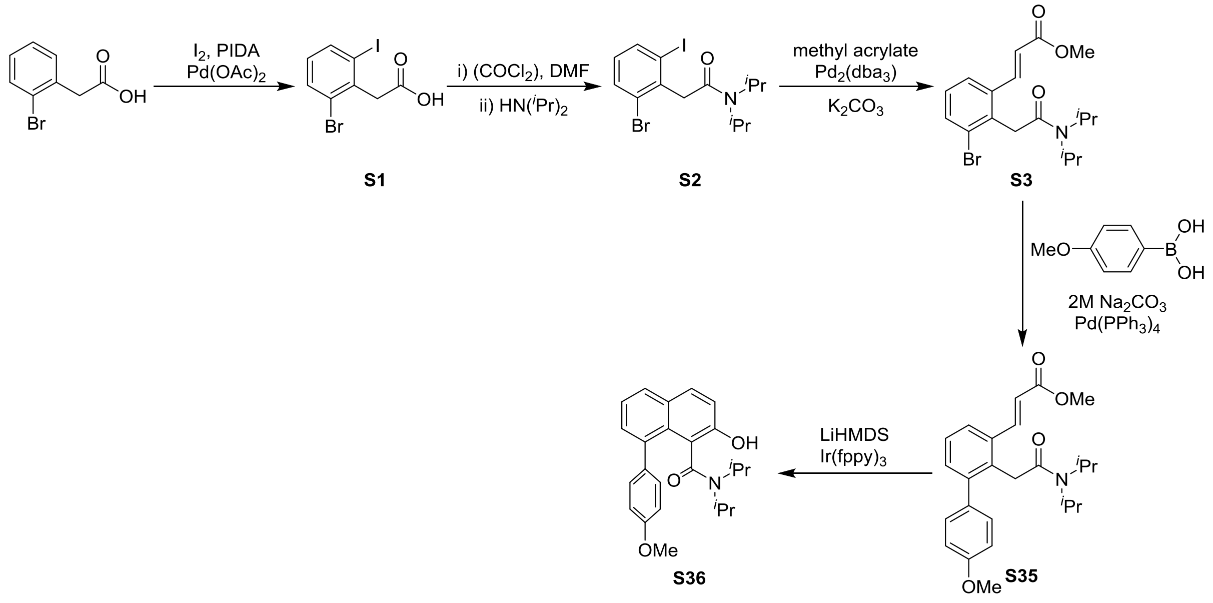

Ethyl (*E*)-3-(2-(2-(diisopropylamino)-2-oxoethyl)-4'-methoxy-[1,1'-biphenyl]-3-yl)acrylate, S35

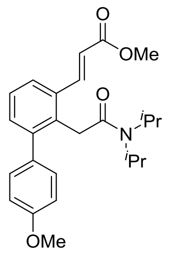

Prepared according to **General Procedure I** with **S3** (150 mg, 0.392 mmol), *p*-methoxy-phenylboronic acid (66 mg, 0.432 mmol), sodium carbonate (124 mg, 1.18 mmol), palladium acetate (4.4 mg, 0.0196 mmol), TBAB (95 mg, 0.392 mmol). Purification by column chromatography, eluting with 10–15% ethyl acetate in petrol, yielded title compound **S35** as a white solid (151 mg, 94%).

m.p. 119–121 °C

^{1}H NMR (400 MHz, $CDCl_3$) δ_H: 7.89 (d, *J* = 15.8 Hz, 1H), 7.58 (dd, *J* = 7.1, 2.1 Hz, 1H), 7.37–7.21 (m, 4H), 6.94 (d, *J* = 8.7 Hz, 2H), 6.40 (d, *J* = 15.8 Hz, 1H), 3.97–3.86 (m, 1H), 3.86 (s, 3H), 3.80 (s, 3H), 3.65 (s, 2H), 3.52–3.33 (m, 1H), 1.43 (d, *J* = 6.7 Hz, 6H), 1.13 (d, *J* = 6.6 Hz, 6H).

^{13}C NMR (101 MHz, $CDCl_3$) δ_C: 168.8, 167.3, 158.9, 143.5, 143.1, 135.1, 133.9, 133.9, 131.6, 130.3, 126.7, 125.7, 119.9, 113.6, 55.3, 51.6, 48.5, 45.9, 37.0, 20.7, 20.5.

FTIR (neat) ν_{max}/cm^{-1} = 2998, 2965, 2933, 2837, 1717, 1634, 1610, 1513, 1442, 1244, 1213, 1166, 1038, 837, 729.

HRMS (ESI$^+$) calculated for $C_{25}H_{32}NO_4$([M+H]$^+$) = 410.2326, found = 410.2326.

2-Hydroxy-*N*,*N*-diisopropyl-8-(4-methoxyphenyl)-1-naphthamide, S36

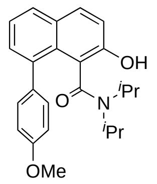

Prepared according to **General Procedure J** with acrylate **S35** (59 mg, 0.144 mmol) and LiHMDS (0.3 mL, 0.300 mmol) in THF (4.9 mL). Purification by column chromatography, eluting with 10% diethyl ether in dichloromethane, yielded title compound **S36** as a white solid (29 mg, 54%).

m.p. 230–233 °C

^{1}H NMR (500 MHz, $CDCl_3$) δ_H: 7.78 (d, *J* = 8.8 Hz, 1H), 7.73 (dd, *J* = 7.0, 2.4 Hz, 1H), 7.59 (s, 1H), 7.49 (brs, 1H), 7.44 (brs, 1H), 7.40–7.32 (m, 2H), 7.23 (d, *J* = 8.8 Hz, 1H), 6.96 (brs, 1H), 6.92–6.87 (brs, 1H), 3.82 (s, 3H), 3.22 (hept, *J* = 6.6 Hz, 1H), 3.09 (hept, *J* = 6.5, 6.0 Hz, 1H), 1.41 (d, *J* = 6.7 Hz, 3H), 0.86 (d, *J* = 6.7 Hz, 3H), 0.68 (d, *J* = 6.8 Hz, 3H), 0.62 (d, *J* = 6.7 Hz, 3H).

^{13}C NMR (126 MHz, $CDCl_3$) δ_C: 170.2, 159., 153.8, 137.9, 133.4, 133.4 (br), 131.4, 130.0 (br), 129.6, 128.6, 128.0, 123.6, 118.8, 117.3, 114.8 (br), 112.3 (br), 55.6, 50.6, 45.9, 21.2, 21.0, 19.7, 18.9.

FTIR (neat) ν_{max}/cm^{-1} = 2957, 2921, 2851, 1589, 1504, 1463, 1442, 1337, 1296, 1171, 1035, 828, 718, 640.

HRMS (*m/z,* ESI^{+}): calculated for $C_{24}H_{28}NO_3$ ($[M+H]^+$) = 378.2064, mass found = 378.2070.

Scheme S11

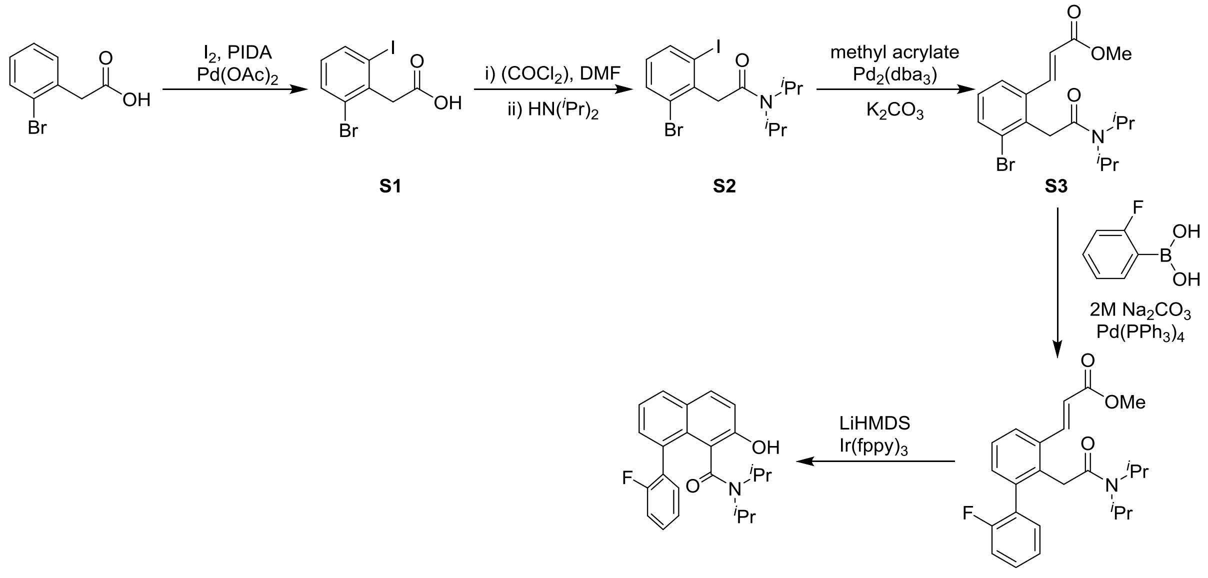

Ethyl (*E*)-3-(2-(2-(diisopropylamino)-2-oxoethyl)-2'-fluoro-[1,1'-biphenyl]-3-yl)acrylate, S37

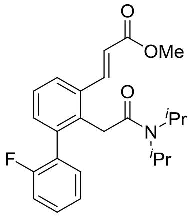

Prepared according to **General Procedure I** with **S3** (150 mg, 0.392 mmol), *o*-fluorophenylboronic acid (60 mg, 0.432 mmol), sodium carbonate (125 mg, 1.18 mmol), palladium acetate (4.4 mg, 0.0200 mmol) and TBAB (95 mg, 0.392 mmol). Purification by column chromatography, eluting with 10–15% ethyl acetate in petrol, yielded title compound **S37** as a white solid (116 mg, 74%).

m.p. 105–108 °C

^{1}H NMR (400 MHz, CDCl$_3$) δ_H: 7.87 (d, *J* = 15.7 Hz, 1H), 7.58 (dd, *J* = 7.6, 1.6 Hz, 1H), 7.36–7.20 (m, 4H), 7.15–7.04 (m, 2H), 6.35 (d, *J* = 15.8 Hz, 1H), 3.87–3.75 (m, 1H), 3.75 (s, 3H), 3.67 (d, *J* = 16.4 Hz, 1H), 3.44 (d, *J* = 16.5, 1H), 3.31 (hept, *J* = 6.4 Hz, 1H), 1.34–1.25 (m, 6H), 1.17–1.05 (m, 3H), 0.89 (d, *J*=6.6, 3H).

^{13}C NMR (101 MHz, CDCl$_3$) δ_C: 168.2, 167.3, 159.5 (d, *J* = 245.7 Hz), 143.3, 136.9, 135.4, 134.6, 132.3 (d, *J* = 3.2 Hz,), 131.9, 129.5 (d, *J* = 7.9 Hz), 128.6 (d, *J* = 16.3), 126.8, 126.7, 124.1 (d, *J* = 3.7 Hz), 120.2, 115.6 (d, *J* = 22.2 Hz), 51.7, 48.5, 46.0, 36.8, 20.7, 20.5.

^{19}F NMR (470 MHz, CDCl$_3$) δ = –114.8 (ddd, *J* = 9.2, 7.7, 5.4 Hz).

FTIR (neat) ν_{max}/cm^{-1} = 2999, 2965, 2929, 2873, 1718, 1635, 1440, 1370, 1276, 1166, 761, 729, 707.

HRMS (ESI$^+$) calculated for $C_{24}H_{28}FNNaO_3$ ([M+Na]$^+$) = 420.1945, found = 420.1939.

8-(2-Fluorophenyl)-2-hydroxy-*N*,*N*-diisopropyl-1-naphthamide, S38

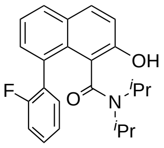

Prepared according to **General Procedure J** with acrylate **S37** (116 mg, 0.292 mmol) and LiHMDS (0.610 mL, 0.610 mmol) in THF (5.8 mL). Purification by column chromatography, eluting with 10% diethyl ether in dichloromethane, yielded title compound **S38** as a 3:2 mixture of diastereomers (67 mg, 62%).

m.p. 238–240 °C

^{1}H NMR (500 MHz, $CDCl_3$) δ_H: 8.07 (s, 0.4H), 7.92 (s, 1H), 7.74 (t, *J* = 8.8 Hz, 1H), 7.60 (d, *J* = 8.8 Hz, 0.6H), 7.50–7.40 (m, 1H), 7.43–7.31 (m, 2H), 7.34–7.22 (m, 1H), 7.19–7.04 (m, 2.6H), 6.92 (d, *J* = 8.8 Hz, 0.4H), 3.40–3.24 (m, 1H), 3.19–3.05 (m, 1H), 1.38–1.29 (m, 3H), 0.93–0.81 (m, 3H), 0.77 (d, *J* = 6.6 Hz, 1.2H), 0.74–0.66 (m, 4.8H).

^{13}C{^{1}H} NMR (126 MHz, $CDCl_3$) δ_C: 170.2, 169.9, 161.5 (d, *J* = 248.9 Hz), 159.7 (d, *J* = 244.3 Hz), 153.6, 153.4, 153.4, 135.3, 135.3, 132.1, 132.1, 132.0, 131.9, 131.8, 131.4, 131.3, 130.7, 130.7, 130.1, 130.0, 129.7, 129.6, 129.5, 129.4, 129.3, 129.3, 129.2, 129.1, 129.0, 128.8, 128.4, 128.3, 123.6, 123.5, 123.5, 123.5, 122.8, 122.7, 119.5, 119.1, 118.6, 117.8, 115.9, 115.7, 115.7, 115.5, 50.9, 50.8, 46.0, 46.0, 20.8, 20.5, 20.5, 20.4, 20.0, 19.7, 19.6, 19.6.

^{19}F NMR (470 MHz, $CDCl_3$) δ_F: −108.1– −108.3 (m), −114.5– −114.7 (m)

FTIR (neat) ν/cm^{-1} = 3054, 2971, 2924, 2753, 2657, 1600, 1573, 1512, 1445, 1365, 1337, 1297, 1209, 908, 831, 757, 728.

HRMS (m/z, ESI$^+$): calculated for $C_{23}H_{25}FNO_2$ ($[M+H]^+$) = 366.1864, found = 366.1865.

Scheme S12

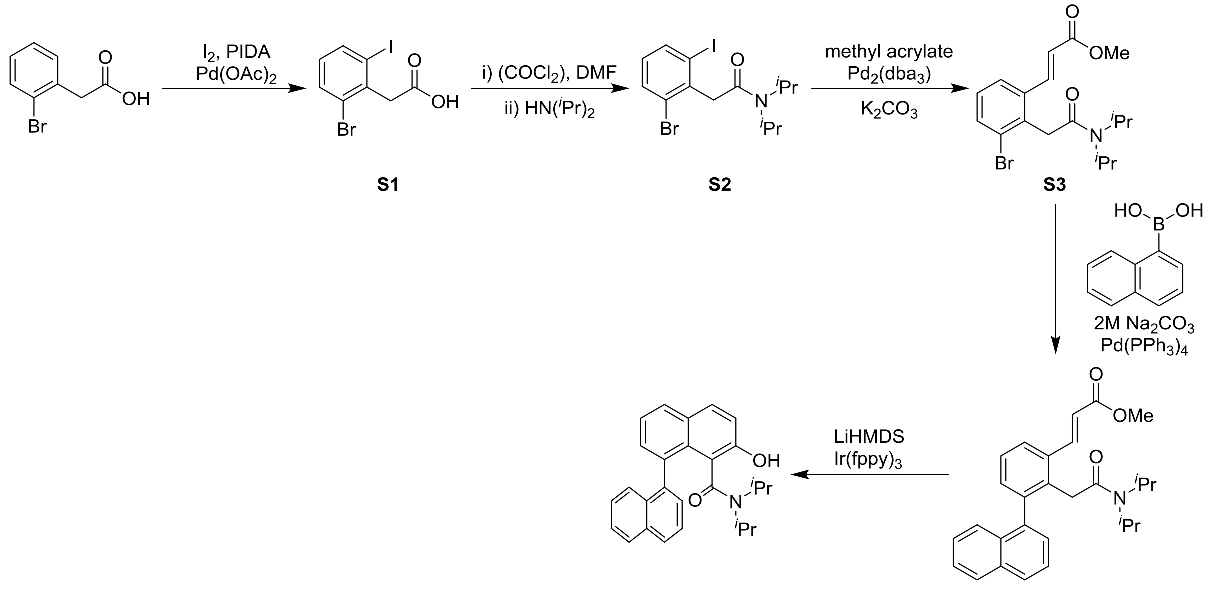

Methyl (*E*)-3-(2-(2-(diisopropylamino)-2-oxoethyl)-3-(naphthalen-1-yl)phenyl)acrylate, S39

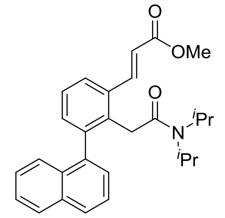

Prepared according to **General Procedure I** with **S3** (150 mg, 0.392 mmol), 1-naph- thylboronic acid (74 mg, 0.432 mmol), sodium carbonate (125 mg, 1.18 mmol), palladium acetate (4.4 mg, 0.0200 mmol) and TBAB (95 mg, 0.392 mmol). Purification by column chromatography, eluting with 10–15% ethyl acetate in petrol, yielded title compound **S39** as a white solid (128 mg, 76%).

m.p. 138 °C

^{1}H NMR (500 MHz, CDCl$_3$) δ_H 7.93 (d, *J* = 15.8 Hz, 1H), 7.87 (d, *J* = 8.4 Hz, 1H), 7.85 (d, *J* = 8.0 Hz, 1H), 7.70 (d, *J* = 7.7 Hz, 1H), 7.55–7.43 (m, 3H), 7.40–7.33 (m, 3H), 7.29 (d, *J* = 7.5 Hz, 1H), 6.46 (d, *J* = 15.8 Hz, 1H), 3.79 (s, 3H), 3.56 (d, *J* = 16.4 Hz, 1H), 3.54–3.47 (m, 1H), 3.33 (d, *J* = 16.4 Hz, 1H), 3.26–3.19 (m, 1H), 1.29 (d, *J* = 6.8 Hz, 3H), 1.20 (d, *J* = 6.7 Hz, 3H), 0.82 (d, *J* = 6.6 Hz, 3H), 0.72 (d, *J* = 6.6 Hz, 3H).

^{13}C NMR (126 MHz, CDCl$_3$) δ_C: 168.2, 167.5, 143.6, 141.4, 138.8, 135.5, 135.2, 133.6, 132.2, 128.1, 127.8, 127.4, 126.8, 126.4, 126.4, 126.3, 125.9, 125.4, 119.8, 51.7, 48.2, 45.8, 36.5, 20.5, 20.5, 20.4, 20.3.

FTIR (neat) ν/cm^{-1} = 3057, 2999, 2965, 1716, 1635, 1442, 1333, 1314, 1275, 1165, 1165, 979, 799, 780, 728.

HRMS (ESI$^+$) calculated for $C_{28}H_{31}NO_3Na$ ([M+Na]$^+$) = 452.2196, found = 452.2189.

7-Hydroxy-*N*,*N*-diisopropyl-[1,1'-binaphthalene]-8-carboxamide, S40

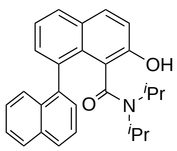

Prepared according to **General Procedure J** with acrylate **S39** (110 mg, 0.151 mmol) and LiHMDS (0.540 mL, 0.540 mmol) in THF (5.1 mL). Purification by column chromatography, eluting with 10% diethyl ether in dichloromethane, yielded title compound **S40** as a 2:1 mixture of diastereomers (61 mg, 59%).

m.p. 225 °C

^{1}H NMR (500 MHz, $CDCl_3$) δ_H: 7.94–7.76 (m, 4H), 7.74 (d, *J* = 8.9 Hz, 0.33H), 7.64 (t, *J* = 8.5 Hz), 7.56–7.46 (m, 1H), 7.49–7.41 (m, 1H), 7.44–7.37 (m, 2H), 7.39–7.31 (m, 1H), 7.23 (t, *J* = 8.7 Hz, 0.33H), 7.12 (d, *J* = 9.0 Hz, 0.33H), 7.06 (d, *J* = 8.8 Hz, 0.67H), 3.41 (hept, *J* = 5.4 Hz, 0.33H), 3.23 (hept, *J* = 6.7 Hz, 0.67H), 2.98 (hept, *J* = 6.6 Hz, 0.33H), 2.81 (hept, *J* = 6.1 Hz, 0.67H), 1.26 (d, *J* = 6.7 Hz, 2H), 1.13 (d, *J* = 6.7 Hz, 1H), 0.99 (d, *J* = 6.6 Hz, 1H), 0.72 (d, *J* = 6.6 Hz, 1H), 0.65 (d, *J* = 6.6 Hz, 2H), 0.38 (d, *J* = 6.9 Hz, 1H), 0.32 (d, *J* = 6.9 Hz, 2H), 0.01 (d, *J* = 6.6 Hz, 2H).

^{13}C NMR (126 MHz, $CDCl_3$) δ_C: 170.2, 168.7, 153.1, 153.0, 139.9, 137.0, 136.6, 135.3, 134.2, 133.7, 133.4, 132.8, 132.7, 132.2, 131.5, 131.2, 131.1, 130.2, 129.9, 129.7, 129.1, 129.0, 128.8, 128.7, 128.5, 128.0, 127.9, 127.7, 126.8, 126.0, 125.9, 125.6, 125.5, 125.4, 124.9, 123.3, 122.7, 119.2, 119.1, 119.1, 119.0, 50.7, 50.5, 45.7, 45.6, 20.7, 20.6, 20.4, 20.4, 20.2, 20.1, 19.7, 19.6.

FTIR (neat) ν/cm^{-1} = 3044, 2996, 2972, 2932, 1591, 1566, 1510, 1397, 1362, 1333, 1275, 833, 779.

HRMS (m/z, ESI-): calculated for $C_{27}H_{28}NO_2$ ($[M+H]^+$) = 398.2115, found = 398.2115.

1.6.2 Enantioselective *O*-Alkylation

The enantioselective *O*-alkylation was carried out according to the general schemes below.

Scheme S13

Asymmetric reaction conditions according to General Procedure L

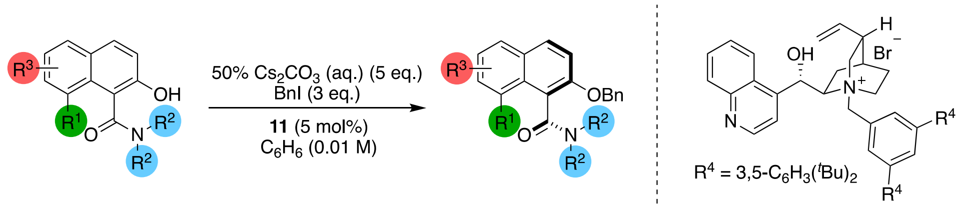

11

Scheme S14

Racemic reaction conditions according to General Procedure K

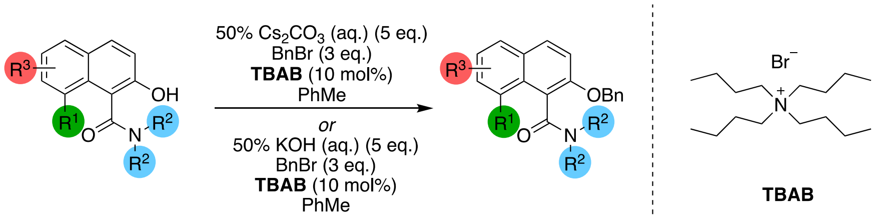

TBAB

3-(Benzyloxy)-*N*,*N*-diisopropylphenanthrene-4-carboxamide, 6

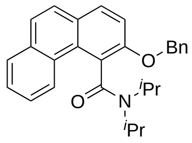

Asymmetric: Prepared according to **General Procedure L** with **5** (64 mg, 0.200 mmol), benzyl iodide (130 mg, 0.600 mmol), **11** (8.4 mg, 0.0100 mmol) and 50% w/w aqueous caesium carbonate (385 µL, 1.00 mmol) in benzene (20.0 mL). Purification by column chromatography, eluting with 5–10% ethyl acetate in petrol, yielded title compound **6** (76 mg, 93%, 97:3 e.r.).

Racemic: Prepared according to **General Procedure K** with **5** (55 mg, 0.171 mmol), benzyl bromide (61 µL, 0.513 mmol), TBAB (5.5 mg, 0.0171 mmol) and 50% w/w aqueous potassium hydroxide (32 µL, 0.856 mmol) in toluene (1.7 mL). Purification by column chromatography eluting with 10% ethyl acetate in petrol afforded title compound **6** (55 mg, 78%).

m.p. 151–153 °C

^{1}H NMR (500 MHz, $CDCl_3$) δ_H: 9.00 (d, *J* = 8.5 Hz, 1H), 7.84 (dd, *J* = 7.8, 1.6 Hz, 2H), 7.83 (d, *J* = 8.7 Hz, 1H), 7.65 (d, *J* = 8.8 Hz, 1H), 7.61 (d, *J* = 8.7 Hz, 1H), 7.57 (ddd, *J* = 7.9, 6.9, 1.3 Hz, 1H), 7.54–7.49 (m, 3H), 7.42–7.37 (m, 2H), 7.36 (d, *J* = 8.8 Hz, 1H), 7.35–7.31 (m, 1H), 5.25 (d, *J* = 11.3 Hz, 1H), 5.17 (d, *J* = 11.3 Hz, 1H), 3.64 (hept, *J* = 6.6 Hz, 1H), 3.53 (hept, *J* = 6.9 Hz, 1H), 1.82 (d, *J* = 6.9 Hz, 3H), 1.50 (d, *J* = 6.8 Hz, 3H), 0.97 (d, *J* = 6.7 Hz, 3H), 0.77 (d, *J* = 6.7 Hz, 3H).

^{13}C{^{1}H} NMR (126 MHz, $CDCl_3$) δ_C: 169.7, 154.3, 136.8, 133.4, 130.2, 129.5, 128.6, 128.5, 128.1, 128.1, 128.0, 128.0, 127.3, 127.0, 126.9, 125.8, 125.7, 124.4, 113.9, 71.9, 51.3, 46.2, 20.6, 20.6, 20.4, 19.6.

FTIR (neat) ν/cm^{-1} = 2973, 1619, 1503, 1453, 1369, 1293, 1211, 1095, 831, 735.

HRMS (*m/z*, ESI$^+$): calculated for $C_{28}H_{30}O_2N$ ([M+H]$^+$) = 412.2271, found = 412.2271.

$[\alpha]_D^{25}$ = −78.8° (97:3 e.r, c = 0.5, $CHCl_3$).

3-(Benzyloxy)-*N*,*N*-dicyclohexylphenanthrene-4-carboxamide, 12

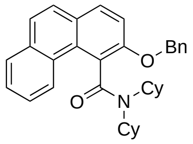

Asymmetric: Prepared according to **General Procedure L** with **S9** (80 mg, 0.200 mmol), benzyl iodide (130 mg, 0.600 mmol), **11** (8.4 mg, 0.0100 mmol) and 50% w/w aqueous caesium carbonate (385 µL, 1.00 mmol) in benzene (20.0 mL). Purification by column chromatography, eluting with 10–20% ethyl acetate in petrol, yielded title compound **12** as a white solid (85 mg, 87%, 98:2 e.r.).

Racemic: Prepared according to **General Procedure K** with **S9** (30 mg, 0.0747 mmol), benzyl bromide (27 µL, 0.224 mmol), TBAB (2.4 mg, 0.00747 mmol) and 50% w/w aqueous potassium hydroxide (28 µL, 0.374 mmol) in toluene (750 µL). Purification by column chromatography, eluting with 10–20% ethyl acetate in petrol, yielded title compound **12** as a white solid (35 mg, 95%).

m.p. 70–72 °C

^{1}H NMR (700 MHz, $CDCl_3$) δ_H: 9.01 (d, *J* = 8.5 Hz, 1H), 7.83 (d, *J* = 8.1 Hz, 1H), 7.82 (d, *J* = 8.4 Hz, 1H), 7.65 (d, *J* = 8.7 Hz, 1H), 7.60 (d, *J* = 8.7 Hz,1H), 7.59–7.52 (m, 1H), 7.53–7.47 (m, 3H), 7.37 (t, *J* = 7.5 Hz, 2H), 7.35–7.29 (m, 2H), 5.25 (d, *J* = 11.5 Hz, 1H), 5.16 (d, *J* = 11.5 Hz, 1H), 3.20–3.13 (m, 1H), 3.11–3.05 (m, 1H), 3.06–2.94 (m, 1H), 2.82–2.73 (m, 1H), 1.98–1.92 (m, 2H), 1.80–1.74 (m, 1H), 1.74–1.69 (m, 1H), 1.70–1.63 (m, 1H), 1.57–1.51 (m, 1H), 1.48–1.41 (m, 1H), 1.41–1.29 (m, 4H), 1.30–1.18 (m, 4H), 0.89–0.80 (m, 1H), 0.81–0.71 (m, 1H), 0.53–0.44 (m, 1H).

^{13}C{^{1}H} NMR (176 MHz, $CDCl_3$) δ_C: 170.0, 154.2, 137.0, 133.4, 130.2, 129.5, 128.6, 128.5, 128.2, 128.0, 128.0, 127.9, 127.3, 127.3, 126.9, 125.8, 125.7, 124.7, 114.0, 72.0, 60.5, 56.5, 31.0, 29.9, 29.2, 27.0, 26.8, 25.8, 25.7, 25.6, 25.3.

FTIR (neat) ν/cm^{-1} = 2929, 2853, 1629, 1503, 1453, 1314, 1268, 1104, 997, 831, 733.

HRMS (*m/z*, ESI$^+$): calculated for $C_{34}H_{38}NO_2$ ([M+H]$^+$) = 492.2897, found = 492.2894.

$[\alpha]_D^{25}$ = −65.8° (98:2 e.r, c = 0.5, $CHCl_3$).

3-(Benzyloxy)-*N*,*N*-diisopropyl-1-methylphenanthrene-4-carboxamide, 13

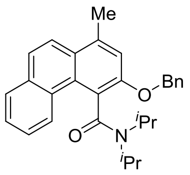

Asymmetric: Prepared according to **General Procedure L** with **S13** (52 mg, 0.155 mmol), benzyl iodide (101 mg, 0.465 mmol), **11** (6.5 mg, 0.00775 mmol) and 50% w/w aqueous caesium carbonate (298 µL, 0.776 mmol) in benzene (15.5 mL). Purification by column chromatography, eluting with 10–20% ethyl acetate in petrol, yielded title compound **13** as a white solid (55 mg, 83%, 97:3 e.r.).

Racemic: Prepared according to **General Procedure K** with **S13** (1 mg, 0.00298 mmol), benzyl bromide (1 µL, 0.0149 mmol), TBAB (1 mg, 0.000298 mmol) and 50% w/w aqueous potassium hydroxide (28 µL, 0.374 mmol) in toluene (100 µL). Purification on an analytical scale by small-scale preparative TLC (20% ethyl acetate in petrol).

m.p. 130°C

^{1}H NMR (500 MHz, $CDCl_3$) δ_H: 9.01 (d, *J* = 8.5 Hz, 1H), 7.89 (d, *J* = 9.1 Hz, 1H), 7.84 (d, *J* = 7.8 Hz, 1H), 7.66 (d, *J* = 9.1 Hz, 1H), 7.59–7.54 (m, 1H), 7.54 – 7.46 (m, 3H), 7.38 (t, *J* = 7.3 Hz, 2H), 7.36–7.29 (m, 1H), 7.25 (s, 1H), 5.25 (d, *J* = 11.3 Hz, 1H), 5.16 (d, *J* = 11.3 Hz, 1H), 3.62 (hept, *J* = 6.7 Hz, 1H), 3.50 (hept, *J* = 6.8 Hz, 1H), 2.77 (s, 3H), 1.79 (d, *J* = 6.8 Hz, 3H), 1.48 (d, *J* = 6.8 Hz, 3H), 0.94 (d, *J* = 6.7 Hz, 3H), 0.73 (d, *J* = 6.7 Hz, 3H).

^{13}C{^{1}H} NMR (126 MHz, $CDCl_3$) δ_C: 170.0, 153.7, 137.0, 136.6, 133.1, 129.9, 128.5, 128.5, 128.3, 128.0, 128.0, 127.4, 126.8, 126.8, 125.7, 125.6, 122.9, 122.7, 115.7, 72.0, 51.3, 46.2, 21.1, 20.6, 20.5, 20.5, 19.5.

FTIR (neat) v/cm^{-1} = 2972, 1624, 1588, 1504, 1368, 1326, 1272, 1114, 1065, 1036, 733.

HRMS (*m/z*, ESI$^+$): calculated for $C_{29}H_{32}NO_2$ ($[M+H]^+$) = 426.2428, found = 426.2424.

$[\alpha]_D^{25}$ = −66.9° (97:3 e.r, c = 0.5, $CHCl_3$).

2-(Benzyloxy)-*N*,*N*-diisopropyl-8-methoxy-1-naphthamide, 14

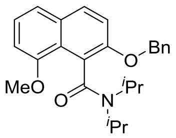

Asymmetric: Prepared according to **General Procedure L** with **S19** (60 mg, 0.200 mmol), benzyl iodide (130 mg, 0.600 mmol), **11** (8.4 mg, 0.0100 mmol) and 50% w/w aqueous caesium carbonate (385 µL, 1.00 mmol) in benzene (20.0 mL). Purification by column chromatography, eluting with 10–20% ethyl acetate in petrol, yielded title compound **14** as a white solid (62 mg, 80%, 96:4 e.r.).

Racemic: Prepared according to **General Procedure K** with **S19** (20 mg, 0.0664 mmol), benzyl bromide (32 µL, 0.332 mmol), TBAB (3.0 mg, 0.00664 mmol) and 50% w/w aqueous potassium hydroxide (28 µL, 0.374 mmol) in toluene (664 µL). Purification on an analytical scale by small-scale preparative TLC (30% ethyl acetate in petrol).

mp: 133–135 °C

^{1}H NMR (500 MHz, $CDCl_3$) δ_H: 7.73 (d, *J* = 8.9 Hz, 1H), 7.53–7.44 (m, 2H), 7.40–7.33 (m, 3H), 7.32–7.27 (m, 2H), 7.27–7.24 (m, 1H), 6.82 (dd, *J* = 7.6, 1.0 Hz, 1H), 5.21 (d, *J* = 11.5 Hz, 1H), 5.13 (d, *J* = 11.6 Hz, 1H), 3.91 (s, 3H), 3.70 (hept, *J* = 6.7 Hz, 1H), 3.45 (hept, *J* = 6.8 Hz, 1H), 1.65 (d, *J* = 6.8 Hz, 3H), 1.46 (d, *J* = 6.8 Hz, 3H), 1.00 (d, *J* = 6.9 Hz, 3H), 0.99 (d, *J* = 6.8 Hz, 3H).

^{13}C{^{1}H} NMR (126 MHz, $CDCl_3$) δ_C: 168.2, 155.2, 152.6, 137.1, 130.9, 129.2, 128.3, 127.8, 127.8, 124.1, 123.0, 121.8, 120.7, 115.8, 106.0, 72.1, 55.3, 50.9, 45.5, 20.6, 20.4, 19.9.

FTIR (neat) v/cm^{-1} = 2972, 1630, 1595, 1513, 1461, 1365, 1337, 1315, 1258, 1217, 1114, 1039, 821, 733, 696.

HRMS (*m/z*, ESI$^+$): calculated for $C_{25}H_{30}NO_3N$ ([M+H]$^+$) = 392.2220, found = 392.2223.

$[\alpha]_D^{25}$ = −75.2° (96:4 e.r, c = 0.5, $CHCl_3$).

2-(Benzyloxy)-*N*,*N*-diisopropyl-8-methyl-1-naphthamide, 15

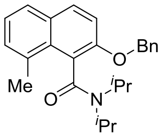

Asymmetric: Prepared according to **General Procedure L** with **S22** (57 mg, 0.200 mmol), benzyl iodide (130 mg, 0.600 mmol), **11** (8.4 mg, 0.0100 mmol) and 50% w/w aqueous caesium carbonate (385 µL, 1.00 mmol) in benzene (20.0 mL). Purification by column chromatography, eluting with 10–20% ethyl acetate in petrol, yielded title compound **15** as a white solid (54 mg, 72%, 98:2 e.r.).

Racemic: Prepared according to **General Procedure K** with **S22** (1 mg, 0.00350 mmol), benzyl bromide (1 µL, 0.0105 mmol), TBAB (0.5 mg, 0.00144 mmol) and 50% w/w aqueous potassium hydroxide (1 µL, 0.0270 mmol) in toluene (100 µL). Purification on an analytical scale by small-scale preparative TLC (30% ethyl acetate in petrol).

m.p. 134 °C

^{1}H NMR (500 MHz, $CDCl_3$) δ_H: 7.79 (d, *J* = 9.0 Hz, 1H), 7.64 (dd, *J* = 8.0, 1.6 Hz, 1H), 7.47 (d, *J* = 6.9 Hz, 2H), 7.36 (t, *J* = 7.6 Hz, 2H), 7.35–7.29 (m, 1H), 7.29–7.22 (m, 3H), 5.18 (d, *J* = 11.4 Hz, 1H), 5.13 (d, *J* = 11.4 Hz, 1H), 3.69 (hept, *J* = 6.6 Hz, 1H), 3.46 (hept, *J* = 6.9 Hz, 1H), 2.78 (s, 3H), 1.59 (d, *J* = 6.8 Hz, 3H), 1.40 (d, *J* = 6.8 Hz, 3H), 1.05–1.00 (m, 6H).

^{13}C{^{1}H} NMR (126 MHz, $CDCl_3$) δ_C: 169.4, 153.5, 137.0, 134.0, 130.9, 130.6, 130.1, 129.9, 128.5, 128.1, 128.1, 127.3, 124.0, 123.4, 114.8, 72.4, 51.2, 46.1, 22.3, 20.7, 20.2, 19.9, 19.8.

FTIR (neat) ν/cm^{-1} = 2972, 1626, 1511, 1433, 1368, 1310, 1247, 1040, 821, 735, 695.

HRMS (*m/z*, ESI^{+}): calculated for $C_{25}H_{30}NO_2$ ([M+H]$^{+}$) = 376.2271, found = 376.2269.

$[\alpha]_D^{25}$ = −167.8° (98:2 e.r, c = 0.5, $CHCl_3$).

2-(Benzyloxy)-*N*,*N*-diisopropyl-5,8-dimethyl-1-naphthamide, 16

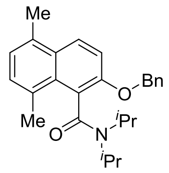

Asymmetric: Prepared according to **General Procedure L** with **S25** (56 mg, 0.187 mmol), benzyl iodide (122 mg, 0.561 mmol), **11** (7.8 mg, 0.00935 mmol) and 50% aqueous caesium carbonate (360 µL, 0.935 mmol) in benzene (18.7 mL). Purification by column chromatography, eluting with 10–20% ethyl acetate in petrol, yielded title compound **S25** as a white solid (62 mg, 80%, 99:1 e.r.).

Racemic: Prepared according to **General Procedure K** with **S25** (1 mg, 0.00334 mmol), benzyl bromide (1.2 µL, 0.0100 mmol), TBAB (0.1 mg, 0.000334 mmol) and 50% aqueous potassium hydroxide (1 µL, 0.0269 mmol) in toluene (100 µL). Purification by small-scale preparative TLC (30% ethyl acetate in petrol).

m.p. 127–128 °C

^{1}H NMR (500 MHz, $CDCl_3$) δ_H: 7.99 (d, *J* = 9.2 Hz, 1H), 7.48 (d, *J* = 6.9 Hz, 2H), 7.40–7.34 (m, 2H), 7.34–7.28 (m, 2H), 7.17 (d, *J* = 6.8 Hz, 1H), 7.10 (d, *J* = 6.8 Hz, 1H), 5.20 (d, *J* = 11.4 Hz, 1H), 5.14 (d, *J* = 11.4 Hz, 1H), 3.66 (hept, *J* = 6.7 Hz, 1H), 3.46 (hept, *J* = 6.8 Hz, 1H), 2.76 (s, 3H), 2.64 (s, 3H), 1.58 (d, *J* = 6.9 Hz, 3H), 1.40 (d, *J* = 6.8 Hz, 3H), 1.02 (d, *J* = 6.7 Hz, 3H), 1.02 (d, *J* = 6.6 Hz, 3H).

^{13}C{^{1}H} NMR (126 MHz, $CDCl_3$) δ_C: 169.6, 153.3, 137.0, 132.8, 132.3, 130.3, 129.8, 129.5, 128.5, 128.1, 128.0, 126.8, 125.1, 123.9, 114.3, 72.2, 51.2, 46.0, 22.4, 20.7, 20.2, 20.2, 19.9, 19.8.

FTIR (neat) ν/cm^{-1} = 2967, 1629, 1519, 1439, 1358, 1315, 1255, 1102, 1039, 827, 737.

HRMS (*m/z*, ESI$^+$): calculated for $C_{26}H_{32}NO_2$ ($[M+H]^+$) = 390.2428, found = 390.2414.

$[\alpha]_D^{25}$ = −199.0° (99:1 e.r, c = 0.5, $CHCl_3$).

2-(Benzyloxy)-*N*,*N*-diisopropyl-8-(trifluoromethyl)-1-naphthamide, 17

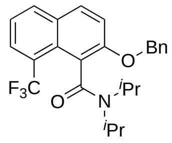

Asymmetric: Prepared according to **General Procedure L** with **S30** (68 mg, 0.200 mmol), benzyl iodide (130 mg, 0.600 mmol), **11** (8.4 mg, 0.0100 mmol) and 50% w/w aqueous caesium carbonate (385 µL, 1.00 mmol) in benzene (20.0 mL). Purification by column chromatography, eluting with 10–20% ethyl acetate in petrol, yielded title compound **17** as a white solid (85 mg, 99%, 95:5 e.r.).

Racemic: Prepared according to **General Procedure K** with **S30** (15 mg, 0.0442 mmol), benzyl bromide (16 µL, 0.133 mmol), TBAB (2 mg, 0.00442 mmol) and 50% w/w aqueous potassium hydroxide (21 µL, 0.221 mmol) in toluene (442 µL). Purification *via* small-scale preparative TLC (30% ethyl acetate in petrol).

m.p. 202–204 °C

^{1}H NMR (500 MHz, $CDCl_3$) δ_H: 8.00 (d, *J* = 7.1 Hz, 1H), 7.97 (d, *J* = 8.1 Hz, 1H), 7.88 (d, *J* = 9.0 Hz, 1H), 7.47 (d, *J* = 8.0 Hz, 2H), 7.41–7.38 (m, 2H), 7.37–7.34 (m, 2H), 7.34–7.29 (m, 1H), 5.20 (d, *J* = 11.4 Hz, 1H), 5.15 (d, *J* = 11.4 Hz, 1H), 3.45 (hept, *J* = 13.4, 6.7 Hz, 1H), 3.39 (hept, *J* = 6.8 Hz, 1H), 1.55 (d, *J* = 6.8 Hz, 3H), 1.31–1.21 (m, 4H), 0.99 (d, *J* = 6.0 Hz, 3H), 0.97 (d, *J* = 6.3 Hz, 3H).

^{13}C{^{1}H} NMR (126 MHz, $CDCl_3$) δ_C: 166.4, 155.6, 136.4, 134.5, 131.9, 130.9, 128.6, 128.5 (q, *J* = 7.7 Hz), 128.4, 128.3, 126.7, 124.6 (q, *J* = 273.3 Hz), 124.5 (q, *J* = 30.9 Hz), 123.0, 122.3, 115.9, 72.5, 50.8, 46.2, 20.7, 20.4, 19.8.

^{19}F NMR (377 MHz, $CDCl_3$) δ_F –55.0.

FTIR (neat) ν/cm^{-1} = 2974, 1632, 1515, 1437, 1295, 1258, 1146, 1059, 1013, 825.

HRMS (*m/z*, ESI$^+$): calculated for $C_{25}H_{27}F_3NO_2$ ([M+H]$^+$) = 430.1988, found = 430.1974.

$[\alpha]_D^{25}$ = –38.6° (95:5 e.r, c = 0.5, $CHCl_3$).

2-(Benzyloxy)-*N*,*N*-diisopropyl-8-phenyl-1-naphthamide, 18

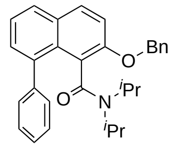

Asymmetric: Prepared according **to General Procedure L** with **S32** (80 mg, 0.200 mmol), benzyl iodide (130 mg, 0.600 mmol), **11** (8.4 mg, 0.0100 mmol) and 50% w/w aqueous caesium carbonate (385 µL, 1.00 mmol) in benzene (20.0 mL). Purification by column chromatography, eluting with 10–20% ethyl acetate in petrol, yielded title compound **18** as a white solid (67 mg, 79%, 98:2 e.r.).

Racemic: Prepared according to **General Procedure K** with **S32** (5 mg, 0.0144 mmol), benzyl bromide (5 µL, 0.0432 mmol), TBAB (0.5 mg, 0.00144 mmol) and 50% w/w aqueous potassium hydroxide (3 µL, 0.0720 mmol) in toluene (100 µL). Purification by small-scale preparative TLC (30% ethyl acetate in petrol).

m.p. 69–71 °C

^{1}H NMR (700 MHz, $CDCl_3$) δ_H: 7.88 (d, *J* = 9.0 Hz, 1H), 7.80 (dd, *J* = 8.2, 1.4 Hz, 1H), 7.63–7.56 (m, 1H), 7.50–7.45 (m, 2H), 7.45–7.42 (m, 1H), 7.38–7.27 (m, 8H), 7.22 (dd, *J* = 7.1, 1.5 Hz, 1H), 5.21 (d, *J* = 11.3 Hz, 1H), 5.09 (d, *J* = 11.3 Hz, 1H), 3.26 (hept, *J* = 6.7 Hz, 1H), 2.98 (hept, *J* = 6.8 Hz, 1H), 1.09 (d, *J* = 6.8 Hz, 3H), 0.84 (d, *J* = 6.6 Hz, 3H), 0.77 (d, *J* = 6.6 Hz, 3H), 0.59 (d, *J* = 6.9 Hz, 3H).

^{13}C{^{1}H} NMR (176 MHz, $CDCl_3$) δ_C: 167.1, 154.1, 141.0, 139.5, 136.9, 133.9, 132.4, 130.8, 130.4, 129.8, 128.7, 128.6, 128.4, 128.1, 128.0, 127.4, 127.3, 126.9, 124.2, 123.4, 115.8, 72.5, 50.4, 45.4, 20.6, 20.5, 19.8, 19.8.

FTIR (neat) ν/cm^{-1} = 2973, 2929, 1633, 1510, 1448, 1367, 1304, 1255, 823, 761, 689.

HRMS (m/z, ESI$^+$): calculated for $C_{30}H_{32}NO_2$ ($[M+H]^+$) = 438.2428, found = 438.2420.

[α]25= −10.1° (98:2 e.r., c = 0.5, CHCl).

2-(Benzyloxy)-8-(4-fluorophenyl)-*N*,*N*-diisopropyl-1-naphthamide, 19

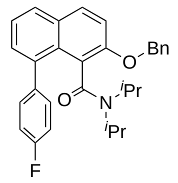

Asymmetric: Prepared according to **General Procedure L** with **S34** (73 mg, 0.200 mmol), benzyl iodide (130 mg, 0.600 mmol), **11** (8.4 mg, 0.00100 mmol) and 50% w/w aqueous caesium carbonate (385 µL, 1.00 mmol) in benzene (20.0 mL). Purification by column chromatography, eluting with 20% ethyl acetate in petrol, yielded title compound **19** as a white solid (63 mg, 69%, 98:2 e.r.).

Racemic: Prepared according to **General Procedure K** with **S34** (1 mg, 0.00298 mmol), benzyl bromide (1 µL, 0.0149 mmol), TBAB (1 mg, 0.000298 mmol) and 50% w/w aqueous potassium hydroxide (28 µL, 0.374 mmol) in toluene (100 µL). Purification on an analytical scale by small-scale preparative TLC (20% ethyl acetate in petrol).

m.p. 126–128 °C

^{1}H NMR (500 MHz, $CDCl_3$) δ_H: 7.89 (d, *J* = 9.0 Hz, 1H), 7.80 (dd, *J* = 8.2, 1.4 Hz, 1H), 7.60–7.54 (m, 1H), 7.46 (d, *J* = 6.9 Hz, 2H), 7.41-7.37 (m, 1H), 7.37–7.31 (m, 4H), 7.31–7.27 (m, 1H), 7.19 (dd, *J* = 7.1, 1.5 Hz, 1H), 7.06–6.97 (m, 2H), 5.20 (d, *J* = 11.3 Hz, 1H), 5.09 (d, *J* = 11.3 Hz, 1H), 3.25 (hept, *J* = 6.7 Hz, 1H), 3.02 (hept, *J* = 6.9 Hz, 1H), 1.10 (d, *J* = 6.8 Hz, 3H), 0.83 (d, *J* = 6.7 Hz, 3H), 0.78 (d, *J* = 6.6 Hz, 3H), 0.71 (d, *J* = 6.8 Hz, 3H).

^{13}C{^{1}H} NMR (126 MHz, $CDCl_3$) δ_C: 167.3, 162.9 (d, *J* = 245.5 Hz), 154.1, 138.3, 136.9 (d, *J* = 3.3 Hz), 136.8, 135.7 (d, *J* = 8.2 Hz), 132.4, 131.1 (d, *J* = 7.9 Hz), 130.8, 130.4, 128.8, 128.7 , 128.4, 128.1, 128.0, 123.9, 123.4, 115.7, 114.0 (d, *J* = 21.5 Hz), 113.8 (d, *J* = 21.3 Hz), 72.4, 50.4, 45.5, 20.6, 20.5, 19.8, 19.7).

^{19}F NMR (377 MHz, $CDCl_3$) δ_F : −116.2.

FTIR (neat) ν/cm^{-1} = 2974, 2931, 1628, 1505, 1434, 1306, 1253, 1220, 1158, 1048, 826, 729.

HRMS (*m/z*, ESI^{+}): calculated for $C_{30}H_{31}FNO_2$ ($[M+H]^+$) = 456.2333, found = 456.2327.

$[\alpha]_D^{25}$ = −16.3° (98:2 e.r, c = 0.5, $CHCl_3$).

2-(Benzyloxy)-*N*,*N*-diisopropyl-8-(4-methoxyphenyl)-1-naphthamide, 20

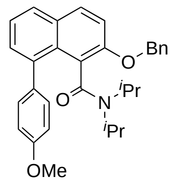

Asymmetric: Prepared according to **General Procedure L** with **S36** (68 mg, 0.180 mmol), benzyl iodide (118 mg, 0.540 mmol), **11** (7.6 mg, 0.00901 mmol) and 50% w/w aqueous caesium carbonate (347 µL, 0.901 mmol) in benzene (18.0 mL). Purification by column chromatography, eluting with 10–20% ethyl acetate in petrol, yielded title compound **20** as a white solid (45 mg, 53%, 98:2 e.r.).

Racemic: Prepared according to **General Procedure K** with **S36** (1 mg, 0.00265 mmol), benzyl bromide (1 µL, 0.00825 mmol), TBAB (0.5 mg, 0.00155 mmol) and 50% w/w aqueous potassium hydroxide (1 µL, 0.0269 mmol) in toluene (100 µL). Purification on an analytical scale by small-scale preparative TLC (30% ethyl acetate in petrol).

m.p. 66–68 °C

^{1}H NMR (500 MHz, $CDCl_3$) δ_H: 7.87 (d, *J* = 9.0 Hz, 1H), 7.78 (dd, *J* = 8.2, 1.4 Hz, 1H), 7.55–7.48 (m, 1H), 7.51–7.43 (m, 2H), 7.38–7.31 (m, 5H), 7.31–7.27 (m, 1H), 7.22 (dd, *J* = 7.1, 1.4 Hz, 1H), 6.87 (d, *J* = 8.8 Hz, 2H), 5.21 (d, *J* = 11.3 Hz, 1H), 5.09 (d, *J* = 11.3 Hz, 1H), 3.82 (s, 3H), 3.24 (hept, *J* = 6.7 Hz, 1H), 3.01 (hept, *J* = 6.9 Hz, 1H), 1.11 (d, *J* = 6.7 Hz, 3H), 0.84 (d, *J* = 6.7 Hz, 3H), 0.76 (d, *J* = 6.6 Hz, 3H), 0.69 (d, *J* = 6.9 Hz, 3H).

^{13}C{^{1}H} NMR (126 MHz, $CDCl_3$) δ_C: 167.3, 159.5, 154.1, 139.2, 137.0, 135.0, 133.3, 132.3, 130.8, 130.8, 130.5, 129.0, 128.4, 128.4, 128.1, 128.0, 124.3 123.5, 115.8, 113.7, 111.6, 72.5, 55.5, 50.4, 45.4, 20.7, 20.5, 19.8.

FTIR (neat) ν/cm^{-1} = 2972, 1631, 1507, 1434, 1306, 1246, 1037, 825, 732, 697.

HRMS (*m/z*, ESI$^+$): calculated for $C_{31}H_{34}NO_3$ ($[M+H]^+$) = 468.2533, found = 468.2527.

$[\alpha]_D^{25}$ = −11.5° (98:2 e.r, c = 0.5, $CHCl_3$).

2-(Benzyloxy)-8-(2-fluorophenyl)-*N*,*N*-diisopropyl-1-naphthamide, 21

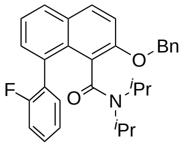

Asymmetric: Prepared according to **General Procedure L** with **S38** (72 mg, 0.200 mmol), benzyl iodide (130 mg, 0.600 mmol), **11** (8.4 mg, 0.0100 mmol) and 50% w/w aqueous caesium carbonate (385 µL, 1.00 mmol) in benzene (20.0 mL). Purification by column chromatography, eluting with 10–20% ethyl acetate in petrol, yielded title compound **21** as a 2:1 mixture of diastereomers (65 mg, 71%, maj. = 96:4 e.r, min. = 96:4 e.r.).

Racemic: Prepared according to **General Procedure K** with **S38** (1 mg, 0.00270 mmol), benzyl bromide (1 µL, 0.0821 mmol), TBAB (0.5 mg, 0.00144 mmol) and 50% w/w aqueous potassium hydroxide (1 µL, 0.0270 mmol) in toluene (100 µL). Purification by small-scale preparative TLC (30% ethyl acetate in petrol).

^{1}H NMR (500 MHz, $CDCl_3$) δ_H: 7.92–7.86 (m, 1H), 7.86–7.79 (m, 1H), 7.54 (td, *J* = 7.8, 1.8 Hz, 0.67H), 7.44 (d, *J* = 6.9 Hz, 2H), 7.40–7.27 (m, 7H), 7.21 (dd, *J* = 7.0, 1.4 Hz, 0.33H), 7.16–7.08 (m, 1H,) 7.08–7.00 (m, 1H), 5.18 (d, *J* = 11.3 Hz, 1H), 5.08 (t, *J* = 11.4 Hz, 1H), 3.35 (hept, *J* = 6.6 Hz, 0.67H), 3.24 (hept, *J* = 6.6 Hz, 0.33H), 3.15–2.89 (m, 1H), 1.11–1.02 (m, 3H), 0.94–0.84 (m, 3H), 0.84–0.78 (m, 3H), 0.67–0.59 (m, 3H).

^{13}C{^{1}H} NMR (126 MHz, $CDCl_3$) δ_C: 167.2, 166.6, 161.8 (d, *J* = 248.8 Hz) 160.0 (d, *J* = 243.2 Hz), 154.2, 154.0, 136.8, 136.4, 136.4, 132.8, 132.6, 132.0, 131.9, 131.9, 131.8, 131.0, 130.8, 130.7, 130.1, 130.0, 130.0, 129.7, 129.5, 129.4, 129.3, 129.3, 129.3, 129.1, 128.5, 128.4, 128.3, 128.3, 128.2, 128.0, 128.0, 123.8, 123.6, 123.4, 123.3, 123.2, 123.1, 123.0, 123.0, 115.8, 115.6, 115.51 (d, *J* = 22.3 Hz), 115.02 (d, *J* = 22.4 Hz), 72.4, 72.4, 50.7, 50.3, 45.6, 45.5, 21.0, 20.8, 20.5, 20.4, 20.3, 20.2, 19.6, 19.5.

^{19}F NMR (377 MHz, $CDCl_3$) δ_F = –107.7 (ddd, *J* = 9.8, 7.4, 5.0 Hz), –113.8 (td, *J* = 8.7, 5.4 Hz).

7-(Benzyloxy)-*N*,*N*-diisopropyl-[1,1'-binaphthalene]-8-carboxamide, 22

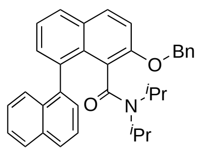

Asymmetric: Prepared according to **General Procedure L** with **S40** (80 mg, 0.200 mmol), benzyl iodide (130 mg, 0.600 mmol), **11** (8.4 mg, 0.0100 mmol) and 50% w/w aqueous caesium carbonate (385 µL, 1.00 mmol) in benzene (20.0 mL). Purification by column chromatography, eluting with 10–20% ethyl acetate in petrol, yielded title compound **22** as a 3:2 mixture of diastereomers (74 mg, 76%, maj. = 98:2 e.r, min. = 96:4 e.r.).

Racemic: Prepared according to **General Procedure K** with **S40** (1 mg, 0.00252 mmol), benzyl bromide (1 µL, 0.0756 mmol), TBAB (0.5 mg, 0.00144 mmol) and 50% w/w aqueous potassium hydroxide (1 µL, 0.0270 mmol) in toluene (100 µL). Purification on an analytical scale by small-scale preparative TLC (30% ethyl acetate in petrol).

^{1}H NMR (500 MHz, $CDCl_3$) δ_H: 7.93 (dd, *J* = 9.0, 5.6 Hz, 1H), 7.91–7.81 (m, 3H), 7.81–7.77 (m, 0.4H), 7.75 (dd, *J* = 7.2, 1.2 Hz, 0.6H), 7.61 (dd, *J* = 8.5, 1.0 Hz, 0.6H), 7.50 (dd, *J* = 8.2, 7.1 Hz, 0.6H), 7.46 – 7.26 (m, 10.4H),7.20 (dd, *J* = 7.0, 1.5 Hz, 0.4H), 5.17 (d, *J* = 11.4 Hz, 0.6H), 5.11 (d, *J* = 11.3 Hz, 0.4H), 5.07 (d, *J* = 11.4 Hz, 0.6H), 5.01 (d, *J* = 11.3 Hz, 0.4H), 3.55 (hept, *J* = 6.6 Hz, 0.4H), 3.17 (hept, *J* = 6.6 Hz, 0.6H), 2.88 (hept, *J* = 6.8 Hz, 0.4H), 2.70 (hept, *J* = 6.8 Hz, 0.6H), 1.01 (d, *J* = 6.6 Hz, 1.2H), 0.97 (d, *J* = 6.8 Hz, 1.8H), 0.87 (d, *J* = 6.5 Hz, 1.2H), 0.83 (d, *J* = 6.7 Hz, 1.2H), 0.71 (d, *J* = 6.5 Hz, 1.8H), 0.31 (d, *J* = 6.9 Hz, 1.8H), 0.27 (d, *J* = 6.9 Hz, 1.2H), −0.14 (d, *J* = 6.5 Hz, 1.8H).

$^{13}C\{^{1}H\}$ NMR (126 MHz, $CDCl_3$) δ_C: 167.3, 166.2, 154.1, 140.4, 137.4, 136.9, 136.7, 136.5, 134.7, 133.9, 133.7, 133.3, 133.2, 132.5, 132.2, 131.0, 131.0, 130.9, 130.4, 130.3, 129.9, 129.0, 128.9, 128.5, 128.4, 128.4, 128.4, 128.3, 128.2, 128.1, 128.1, 128.0, 127.8, 127.7, 127.4, 126.9, 125.8, 125.7, 125.6, 125.3, 125.1, 124.5, 124.4, 123.9, 123.4, 123.2, 115.8, 115.1, 72.5, 72.1, 50.4, 50.3, 45.4, 45.2, 21.1, 20.7, 20.4, 20.4, 20.3, 19.8, 19.5, 19.3.

FTIR (neat) ν/cm^{-1} = 2980, 2857, 1633, 1502, 1452, 1272, 1228, 1113, 834, 737.

HRMS (*m/z*, ESI$^+$): calculated for $C_{34}H_{34}NO_2$ ($[M+H]^+$): 488.2584, found 488.2580.

1.6.3 Derivatization of the *O*-alkylation product

***N*,*N*-Diisopropyl-5,8-dimethyl-2-(triethylsilyl)-1-naphthamide, 23**

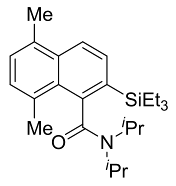

Asymmetric: A flame-dried Schlenk flask containing a stirring bar was charged with **16** (41 mg, 0.100 mmol, 95:5 e.r.). The flask was introduced in a nitrogen atmosphere glovebox where 26 µL of a freshly prepared solution of $Ni(cod)_2$ in degassed anhydrous toluene (0.1 M, 10 mol%), $Et_3SiBpin$ (93 µL, 1.3 equiv.)[6], ^{t}BuOK (64 mg, 2.2 equiv.) and toluene (1.3 mL) were then added sequentially. The reaction mixture was stirred at room temperature for 3 h. The reaction crude was allowed to cool down to room temperature, concentrated *in vacuo*, and purified by column chromatography (40% diethyl ether in petrol), yielded title compound **23** as a colourless liquid (26 mg, 63%, 95:5 e.r.).

Racemic: Prepared using the same procedure as its asymmetric version with *rac*-**16** (41 mg, 0.100 mmol). Purification by column chromatography (40% diethyl ether in petrol), yielded title compound **23** as a colourless liquid (25 mg, 59%)

^{1}H NMR (500 MHz, $CDCl_3$) δ_H: 7.40 (d, *J* = 9.1 Hz, 1H), 7.02 (d, *J* = 6.9 Hz, 1H), 6.93 (d, *J* = 7.1 Hz, 1H), 6.69 (d, *J* = 9.1 Hz, 1H), 3.50 (m, 1H), 3.43 (m, 1H), 2.58 (s, 3H), 2.45 (s, 3H), 1.51 (d, *J* = 6.9 Hz, 3H), 1.47 (d, *J* = 6.8 Hz, 3H), 0.91 (d, *J* = 6.7 Hz, 3H), 0.87 (d, *J* = 6.6 Hz, 3H), 0.66 (t, *J* = 7.8 Hz, 15H), 0.53 (q, *J* = 7.8 Hz, 10H)

^{13}C{^{1}H} NMR (126 MHz, $CDCl_3$) δ_C: 169.9, 133.5, 130.5, 129.0, 128.0, 127.1, 126.2, 124.4, 122.1, 117.1, 116.1, 49.4, 44.3, 19.9, 18.4, 18.4, 17.9, 17.5, 17.3, 5.5, 1.5.

FTIR (neat) ν/cm^{-1} = 2931, 2857, 2155, 2026, 1968, 1585, 1520, 1443, 1411.

HRMS (*m/z*, ESI^{+}): calculated for $C_{25}H_{40}NOSi$ ($[M+H]^{+}$) = 367.2801, found = 367.2800.

$[\alpha]_D^{25}$ = −145.2° (95:5 e.r, c = 0.5, $CHCl_3$).

1.7 References

[1] A. B. Pangborn, M. A. Giardello, R. H. Grubbs, R. K. Rosen, F. J. Timmers, *Organometallics* **1996**, 15, 1518.

[2] D. B. G. Williams, M. Lawton, *J. Org. Chem.* **2010**, 75, 8351.

[3] D.-H. Wang, K. M. Engle, B.-F. Shi, J.-Q. Yu, *Science* **2010**, *327*, 315.

[4] a) R. Ceccarelli, S. Insogna, M. Bella, *Org. Biomol. Chem.* **2006**, 4, 4281; b) M. M. Lian, Z. Li, J. A. Du, Q. W. Meng, Z. X. Gao, Eur. *J. Org. Chem.* **2010**, 6525; c) T. Perrard, J. C. Plaquevent, J. R. Desmurs, D. Hebrault, *Org. Lett.* **2000**, 2, 2959.

[5] T.-S. Mei, D.-H. Wang, J.-Q. Yu*, Org. Lett.* **2010**, 12, 3140.

[6] $Et_3SiBpin$ was prepared according to the procedure reported by Hartwig *et al*., *Organometallics*, **2008**, *27*, 6013-6017.

1.8 NMR Spectra and HPLC Traces

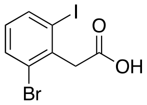

S1

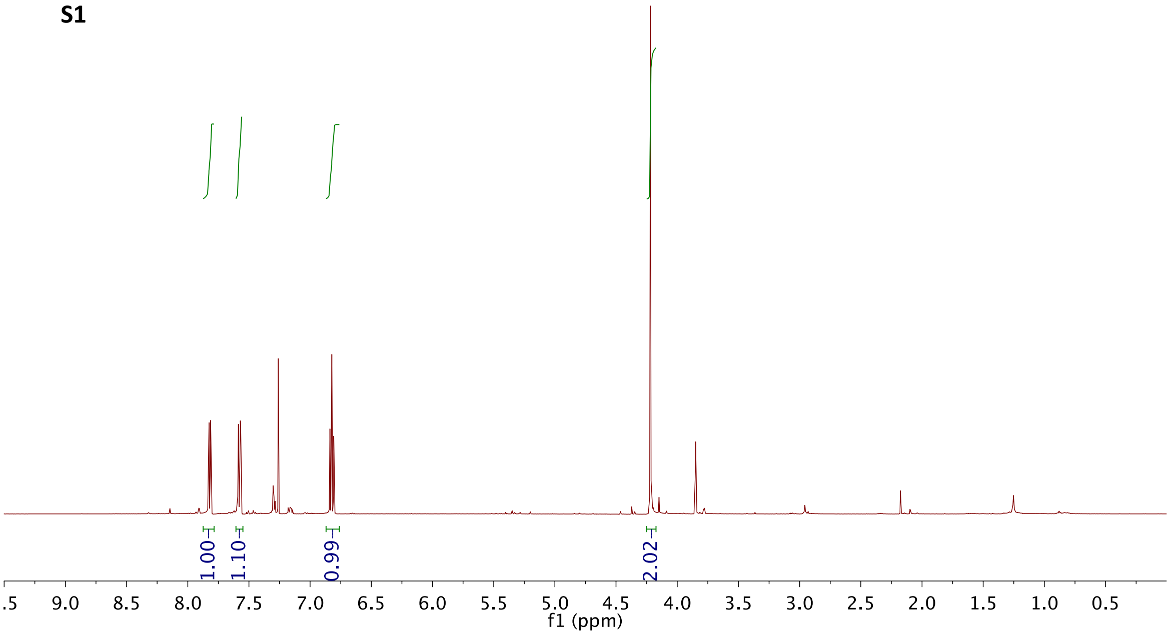

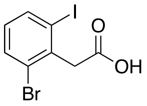

S1

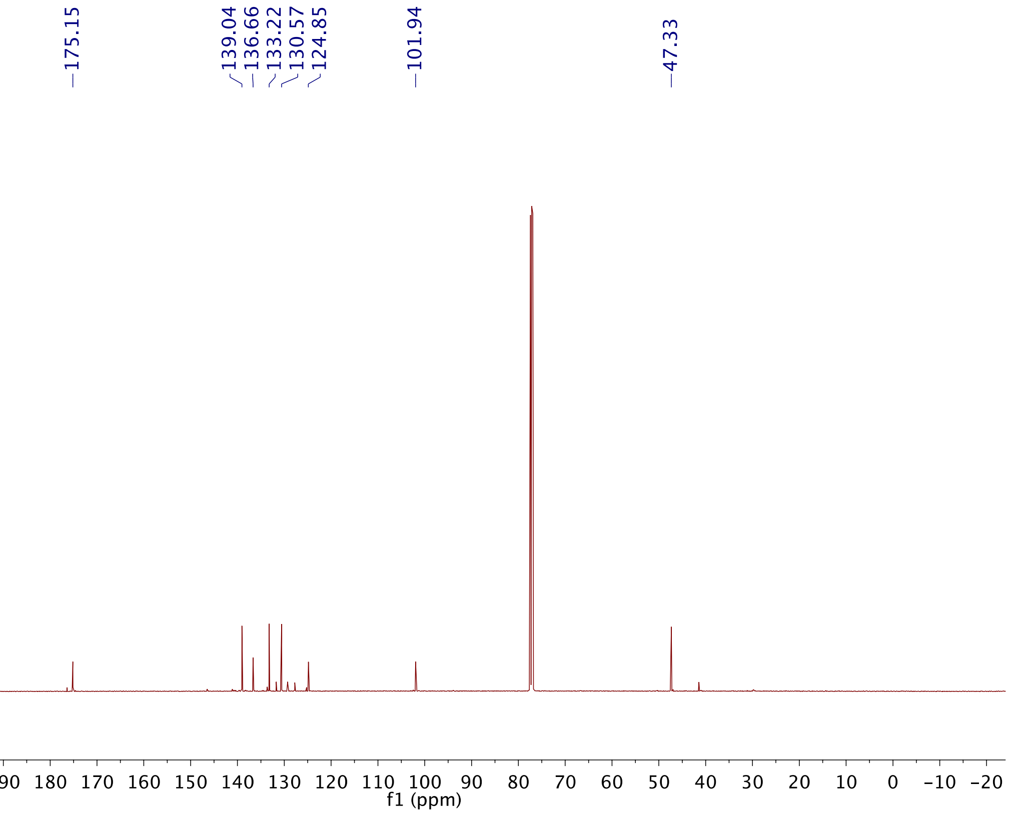

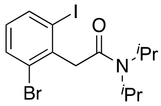

I
O
N
iPr
iPr
Br

S2

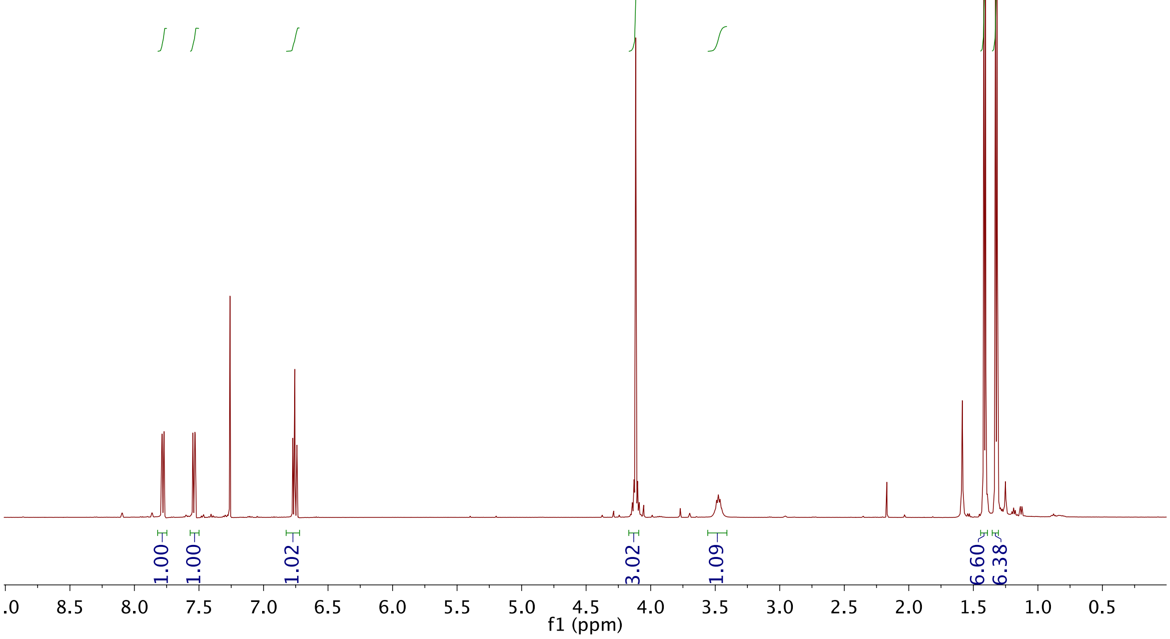

1.00
1.00
1.02
3.02
1.09
6.60
6.38
f1 (ppm)

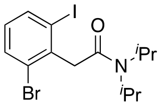

I
O
N
iPr
iPr
Br

S2

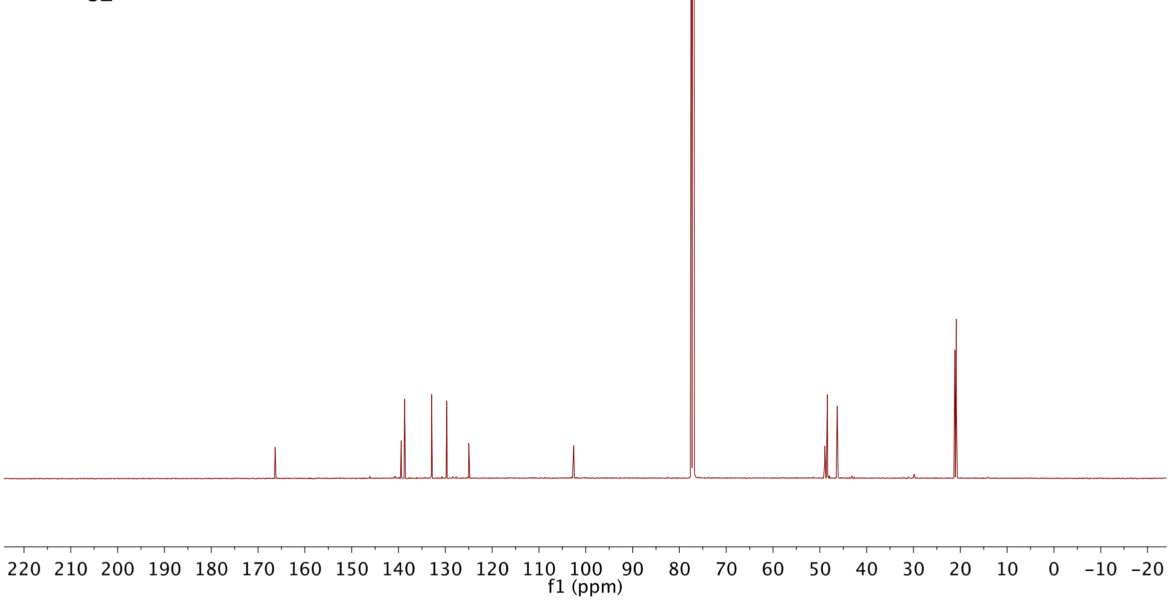

166.33
139.44
138.69
132.93
129.66
124.97
102.59
48.94
48.38
46.30
21.16
20.86
f1 (ppm)

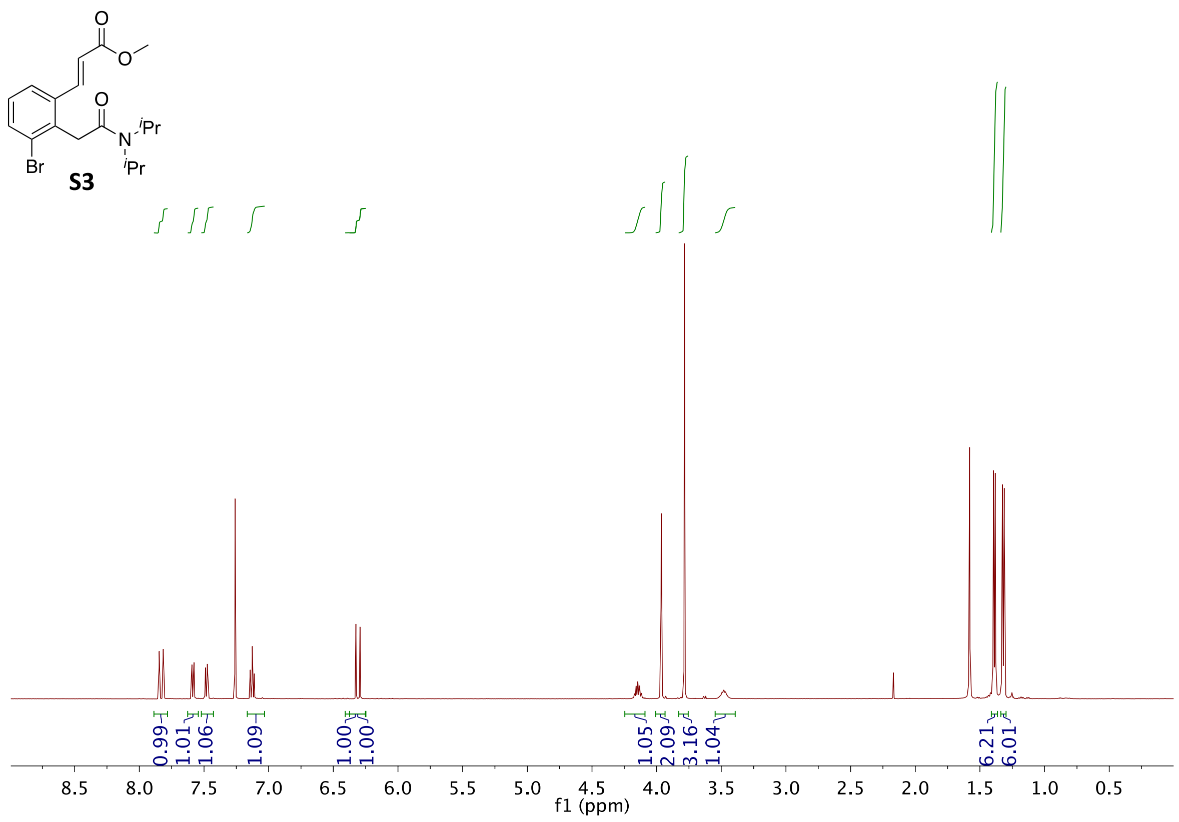

Br
N
iPr
iPr
S3
0.99
1.01
1.06
1.09
1.00
1.00
1.05
2.09
3.16
1.04
6.21
6.01
8.5 8.0 7.5 7.0 6.5 6.0 5.5 5.0 4.5 4.0 3.5 3.0 2.5 2.0 1.5 1.0 0.5
f1 (ppm)

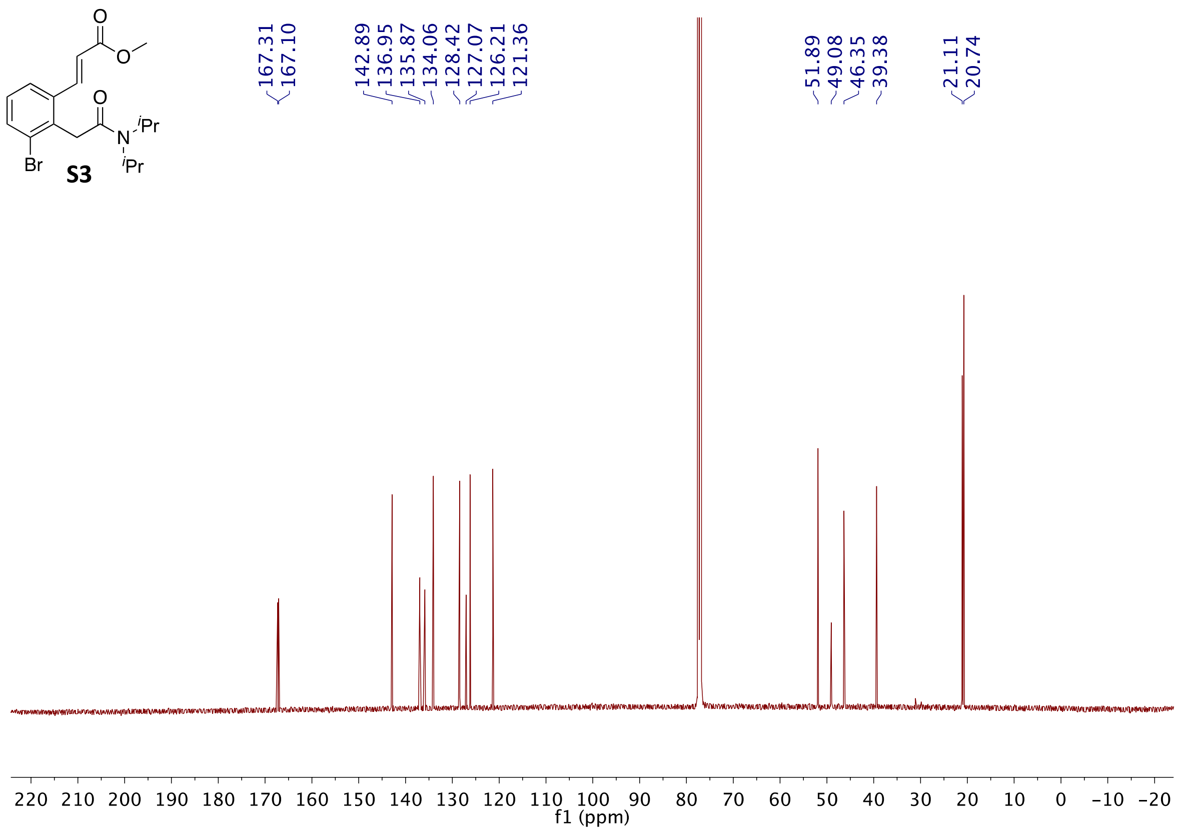

Br
S3
N
iPr
iPr
167.31
167.10
142.89
136.95
135.87
134.06
128.42
127.07
126.21
121.36
51.89
49.08
46.35
39.38
21.11
20.74
220 210 200 190 180 170 160 150 140 130 120 110 100 90 80 70 60 50 40 30 20 10 0 −10 −20
f1 (ppm)

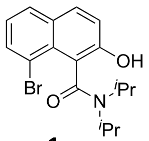

1

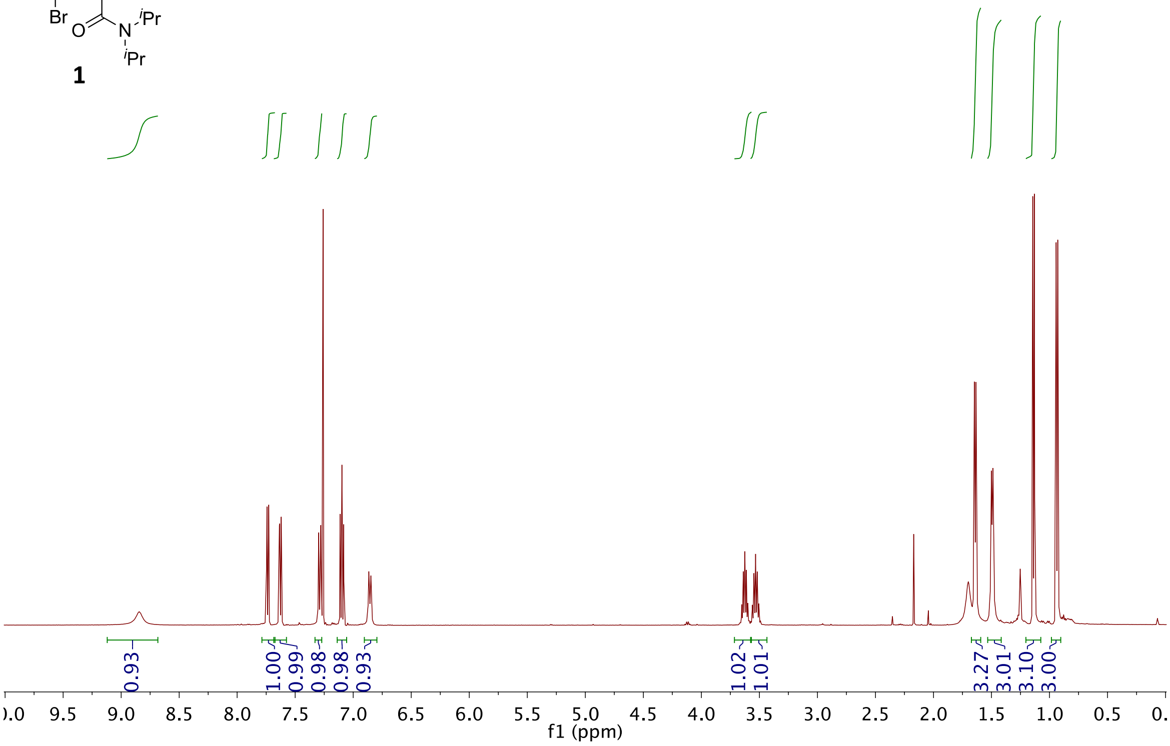
0.93
1.00
0.99
0.98
0.98
0.93
1.02
1.01
3.27
3.01
3.10
3.00
f1 (ppm)

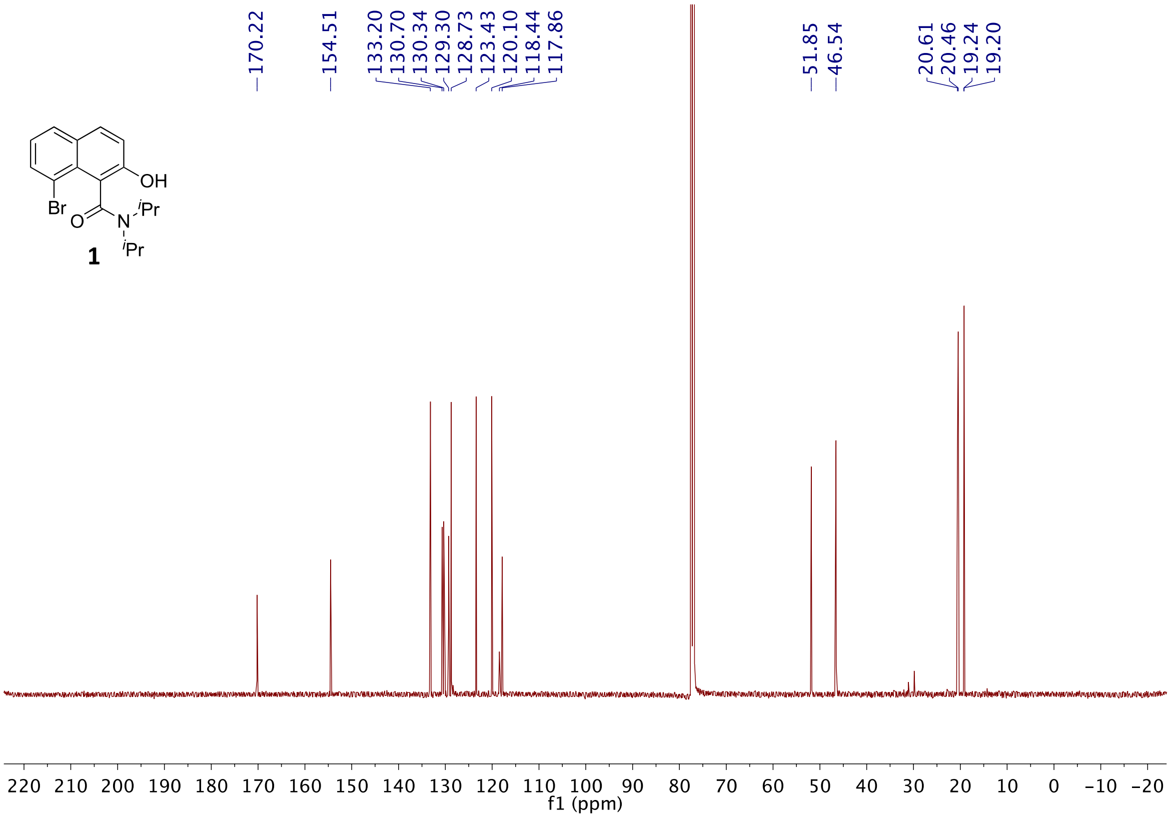
170.22
154.51
133.20
130.70
130.34
129.30
128.73
123.43
120.10
118.44
117.86
51.85
46.54
20.61
20.46
19.24
19.20
f1 (ppm)

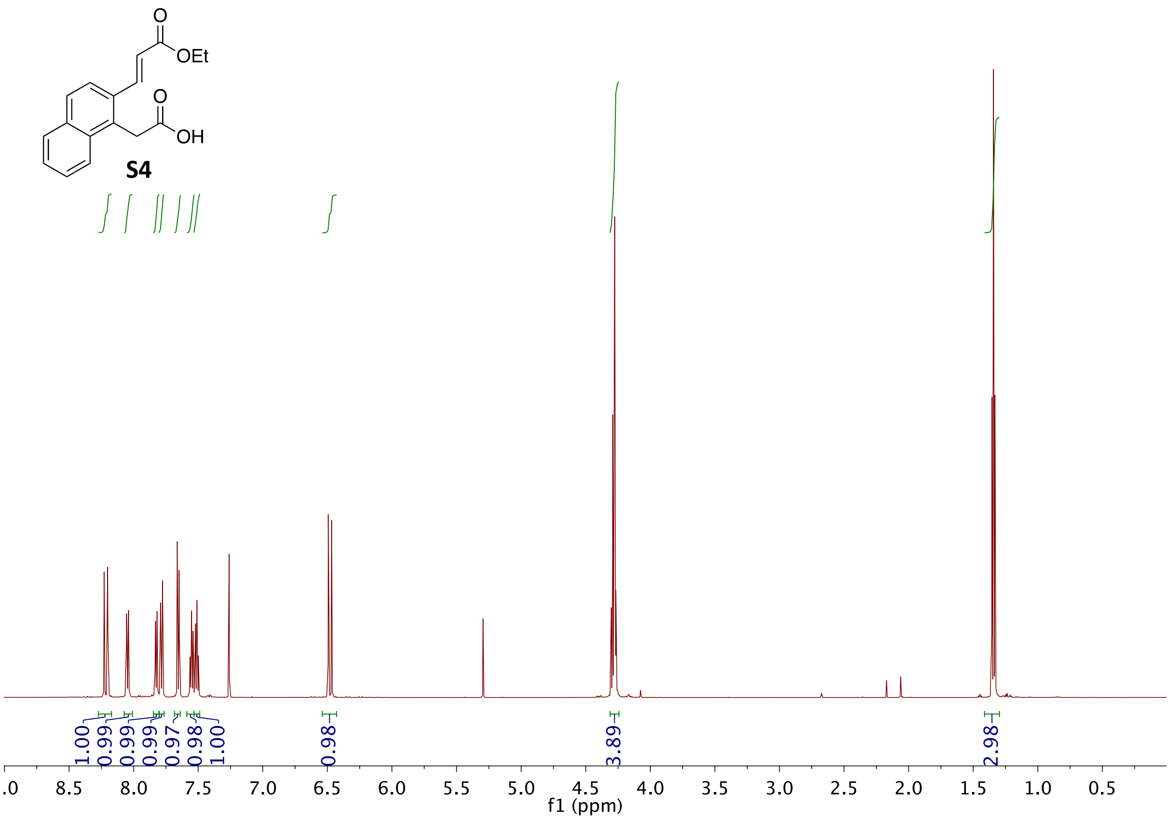
OEt
OH
S4
1.00
0.99
0.99
0.99
0.97
0.98
1.00
0.98
3.89
2.98
.0 8.5 8.0 7.5 7.0 6.5 6.0 5.5 5.0 4.5 4.0 3.5 3.0 2.5 2.0 1.5 1.0 0.5
f1 (ppm)

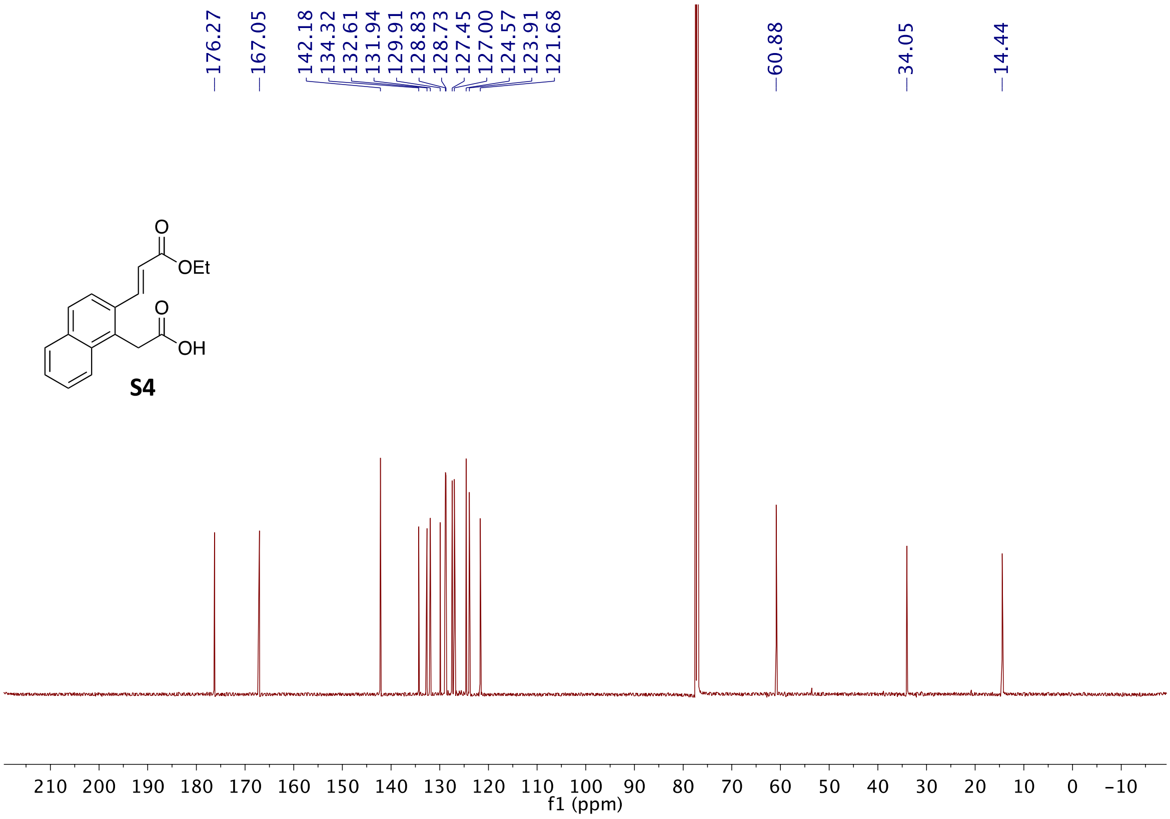
176.27
167.05
142.18
134.32
132.61
131.94
129.91
128.83
128.73
127.45
127.00
124.57
123.91
121.68
60.88
34.05
14.44
OEt
OH
S4
210 200 190 180 170 160 150 140 130 120 110 100 90 80 70 60 50 40 30 20 10 0 -10
f1 (ppm)

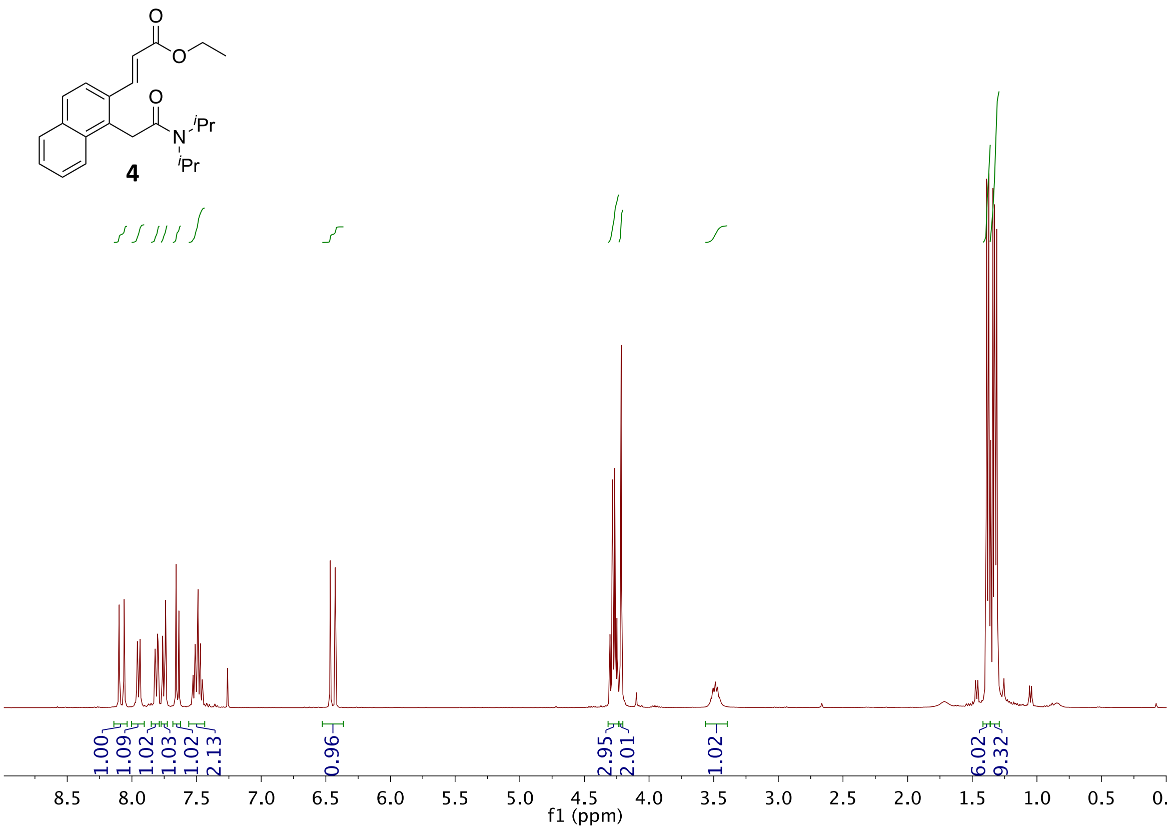

O
O
O
N
iPr
iPr
4
1.00
1.09
1.02
1.03
1.02
2.13
0.96
2.95
2.01
1.02
6.02
9.32
8.5 8.0 7.5 7.0 6.5 6.0 5.5 5.0 4.5 4.0 3.5 3.0 2.5 2.0 1.5 1.0 0.5 0.
f1 (ppm)

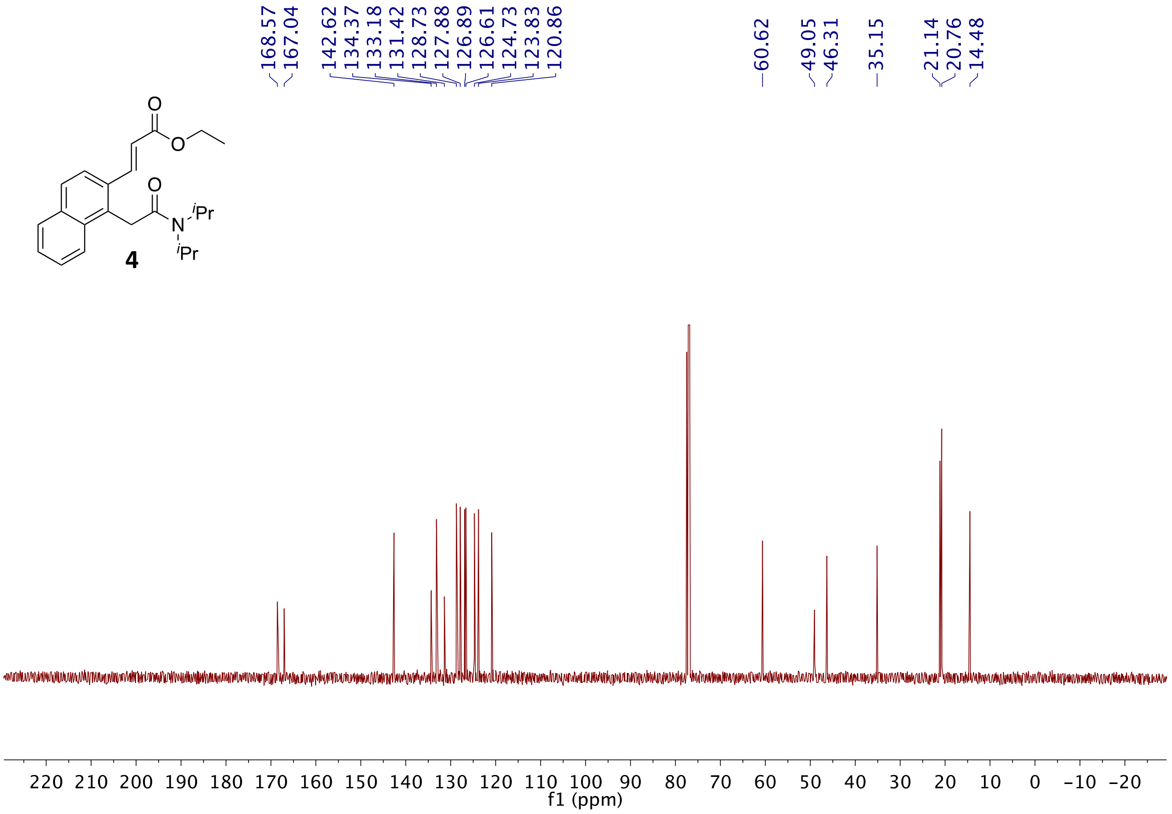

168.57
167.04
142.62
134.37
133.18
131.42
128.73
127.88
126.89
126.61
124.73
123.83
120.86
60.62
49.05
46.31
35.15
21.14
20.76
14.48
O
O
O
N
iPr
iPr
4
220 210 200 190 180 170 160 150 140 130 120 110 100 90 80 70 60 50 40 30 20 10 0 −10 −20
f1 (ppm)

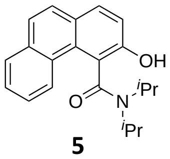
OH
N
O
iPr
iPr

5

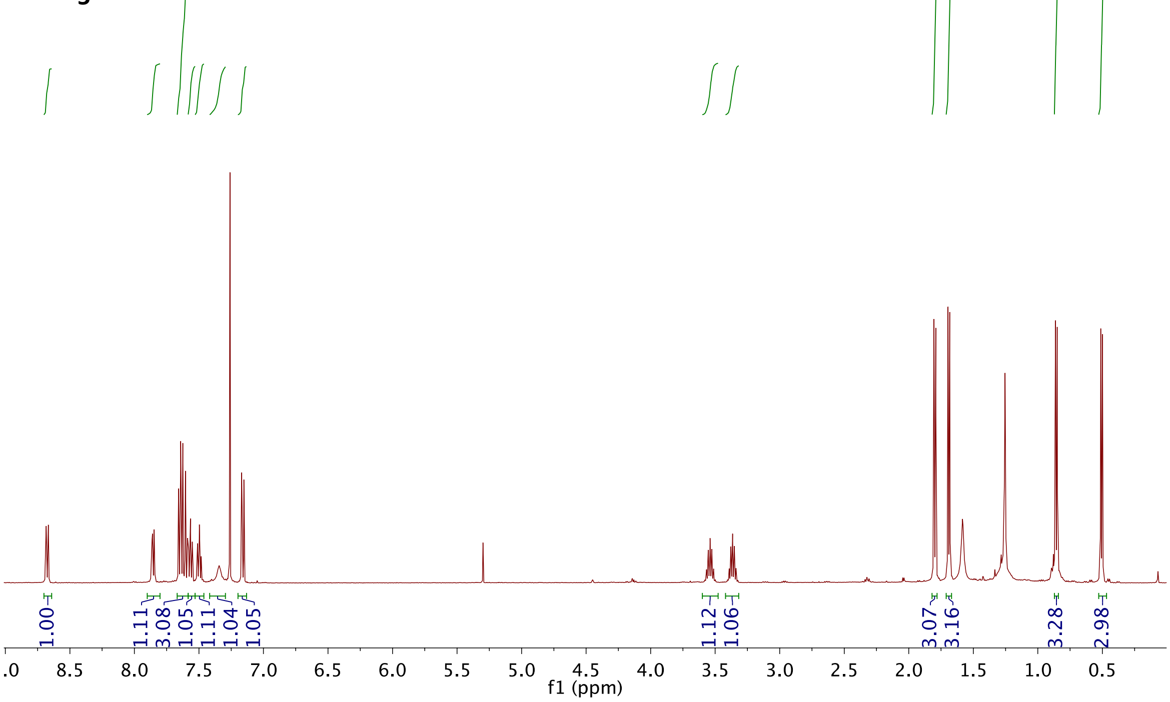
1.00
1.11
3.08
1.05
1.11
1.04
1.05
1.12
1.06
3.07
3.16
3.28
2.98
.0 8.5 8.0 7.5 7.0 6.5 6.0 5.5 5.0 4.5 4.0 3.5 3.0 2.5 2.0 1.5 1.0 0.5
f1 (ppm)

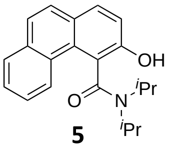
OH
N
O
iPr
iPr

5

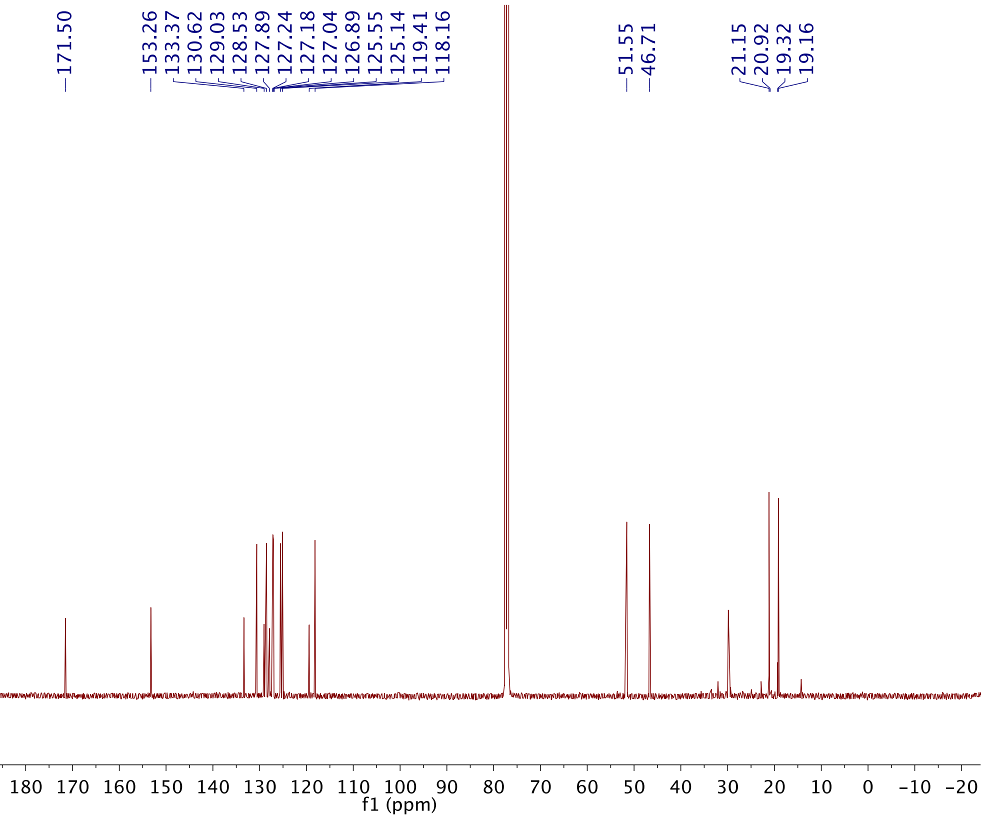
171.50
153.26
133.37
130.62
129.03
128.53
127.89
127.24
127.18
127.04
126.89
125.55
125.14
119.41
118.16
51.55
46.71
21.15
20.92
19.32
19.16
220 210 200 190 180 170 160 150 140 130 120 110 100 90 80 70 60 50 40 30 20 10 0 −10 −20
f1 (ppm)

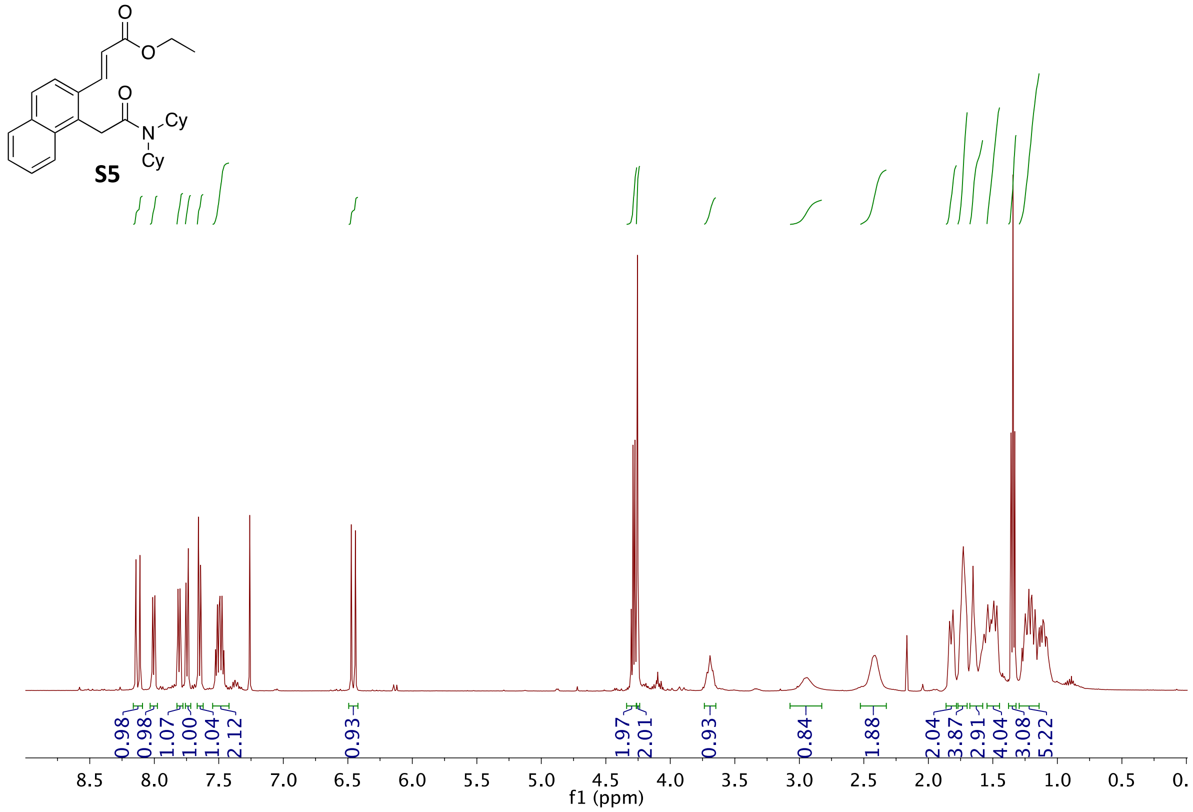

O
O
O
N
Cy
Cy
S5
0.98
0.98
1.07
1.00
1.04
2.12
0.93
1.97
2.01
0.93
0.84
1.88
2.04
3.87
2.91
4.04
3.08
5.22
8.5 8.0 7.5 7.0 6.5 6.0 5.5 5.0 4.5 4.0 3.5 3.0 2.5 2.0 1.5 1.0 0.5 0.
f1 (ppm)

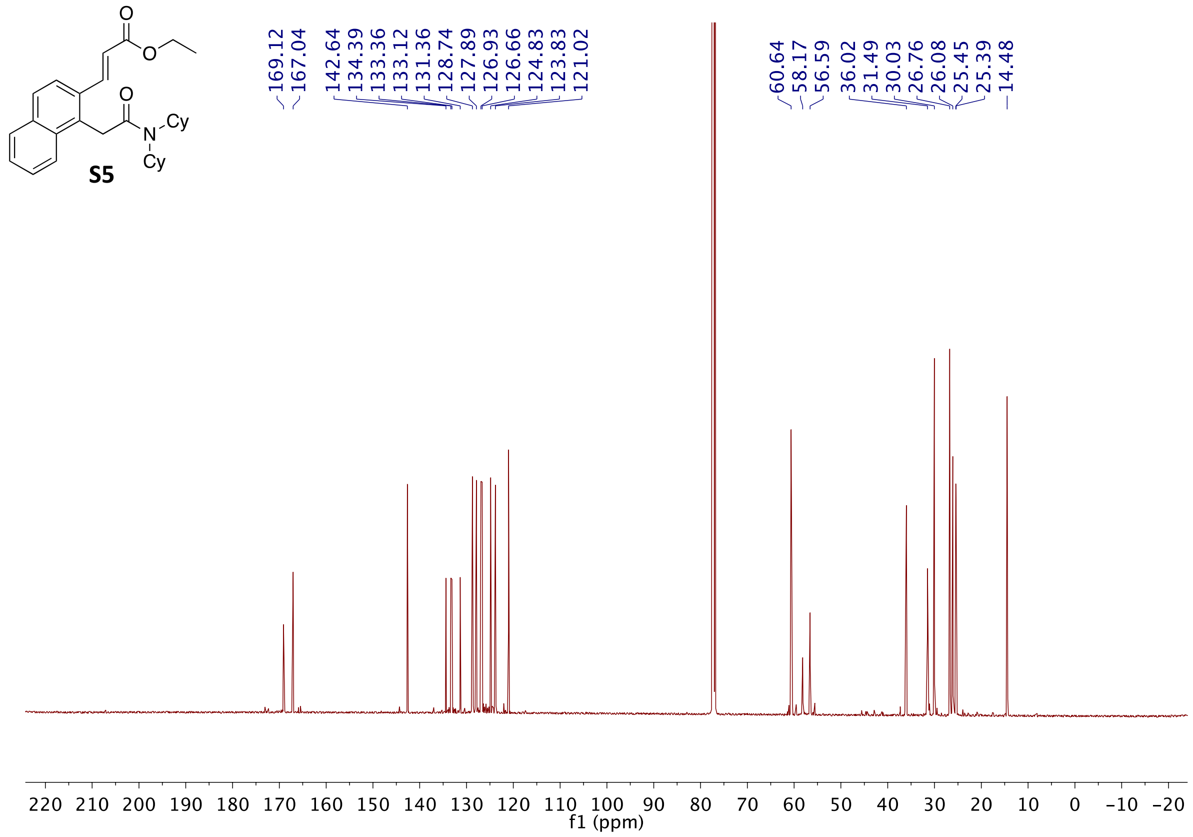

O
O
O
N
Cy
Cy
S5
169.12
167.04
142.64
134.39
133.36
133.12
131.36
128.74
127.89
126.93
126.66
124.83
123.83
121.02
60.64
58.17
56.59
36.02
31.49
30.03
26.76
26.08
25.45
25.39
14.48
220 210 200 190 180 170 160 150 140 130 120 110 100 90 80 70 60 50 40 30 20 10 0 −10 −20
f1 (ppm)

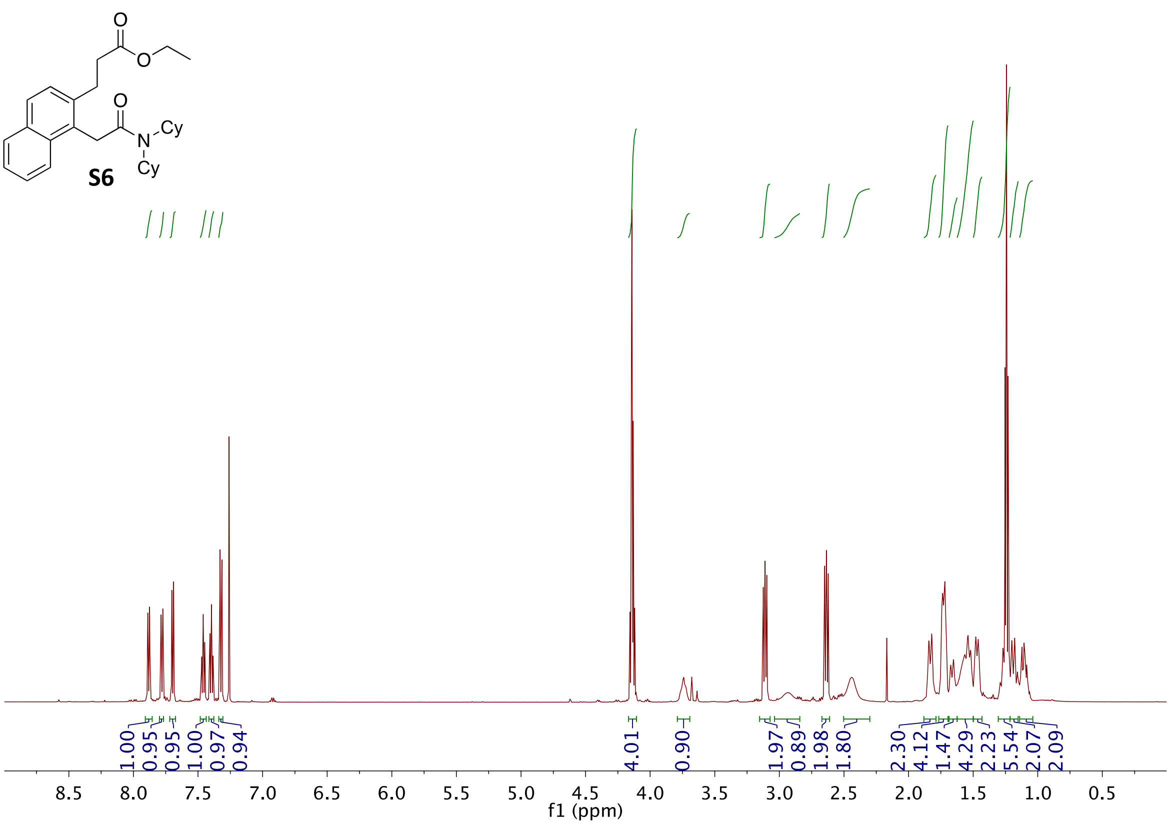
S6
1.00
0.95
0.95
1.00
0.97
0.94
4.01
0.90
1.97
0.89
1.98
1.80
2.30
4.12
1.47
4.29
2.23
5.54
2.07
2.09
8.5 8.0 7.5 7.0 6.5 6.0 5.5 5.0 4.5 4.0 3.5 3.0 2.5 2.0 1.5 1.0 0.5
f1 (ppm)

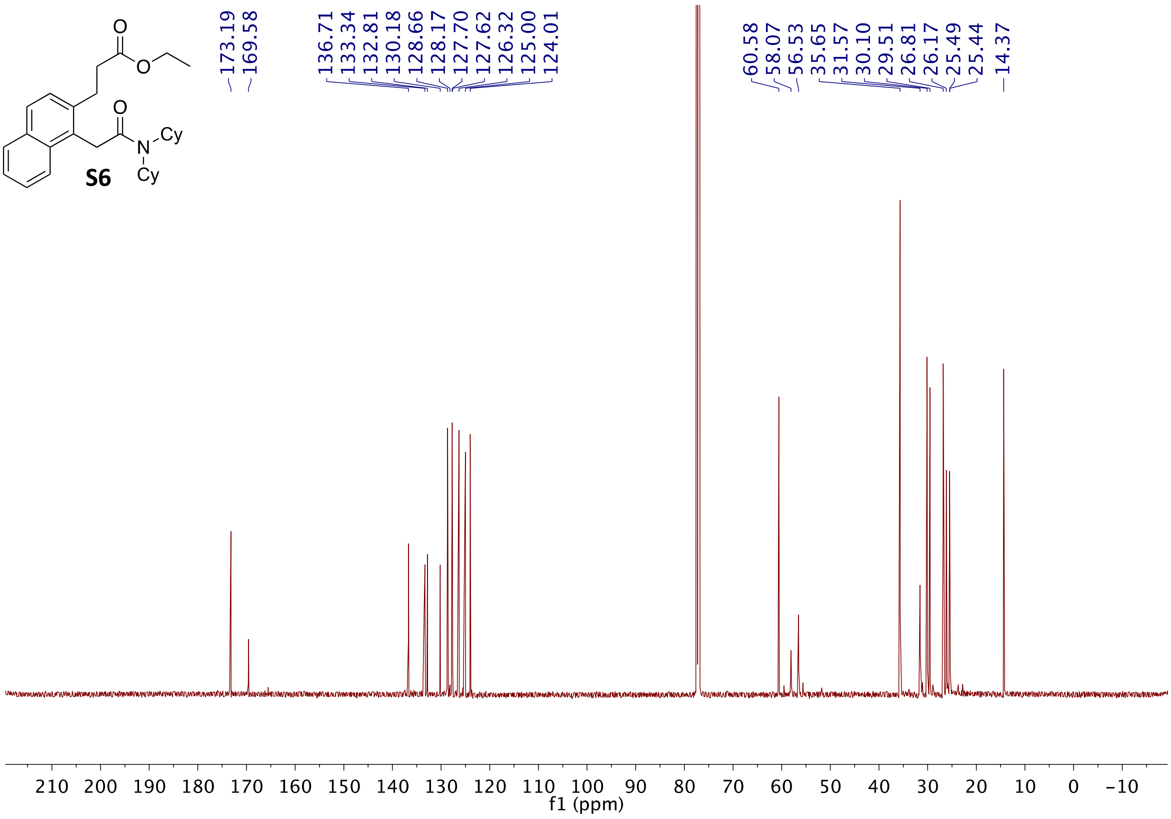
S6
173.19
169.58
136.71
133.34
132.81
130.18
128.66
128.17
127.70
127.62
126.32
125.00
124.01
60.58
58.07
56.53
35.65
31.57
30.10
29.51
26.81
26.17
25.49
25.44
14.37
210 200 190 180 170 160 150 140 130 120 110 100 90 80 70 60 50 40 30 20 10 0 −10
f1 (ppm)

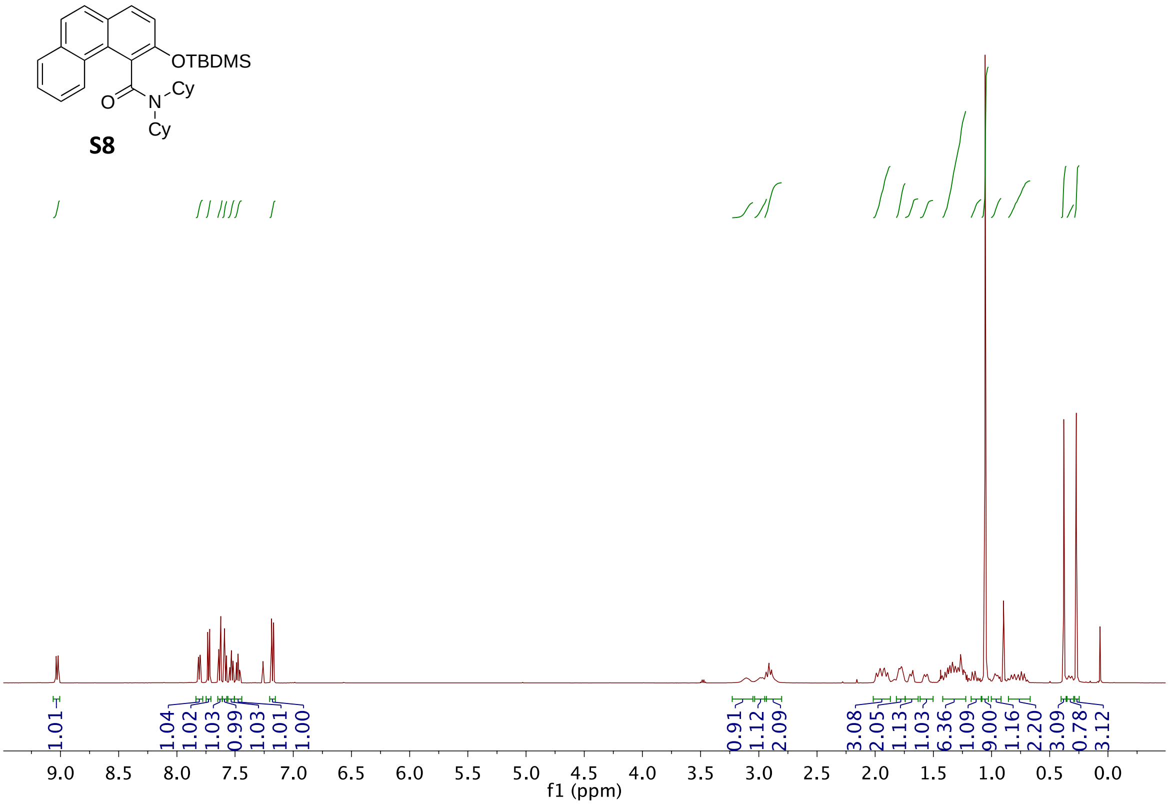
OTBDMS
O
N
Cy
Cy
S8
1.01
1.04
1.02
1.03
0.99
1.03
1.01
1.00
0.91
1.12
2.09
3.08
2.05
1.13
1.03
6.36
1.09
9.00
1.16
2.20
3.09
0.78
3.12
9.0 8.5 8.0 7.5 7.0 6.5 6.0 5.5 5.0 4.5 4.0 3.5 3.0 2.5 2.0 1.5 1.0 0.5 0.0
f1 (ppm)

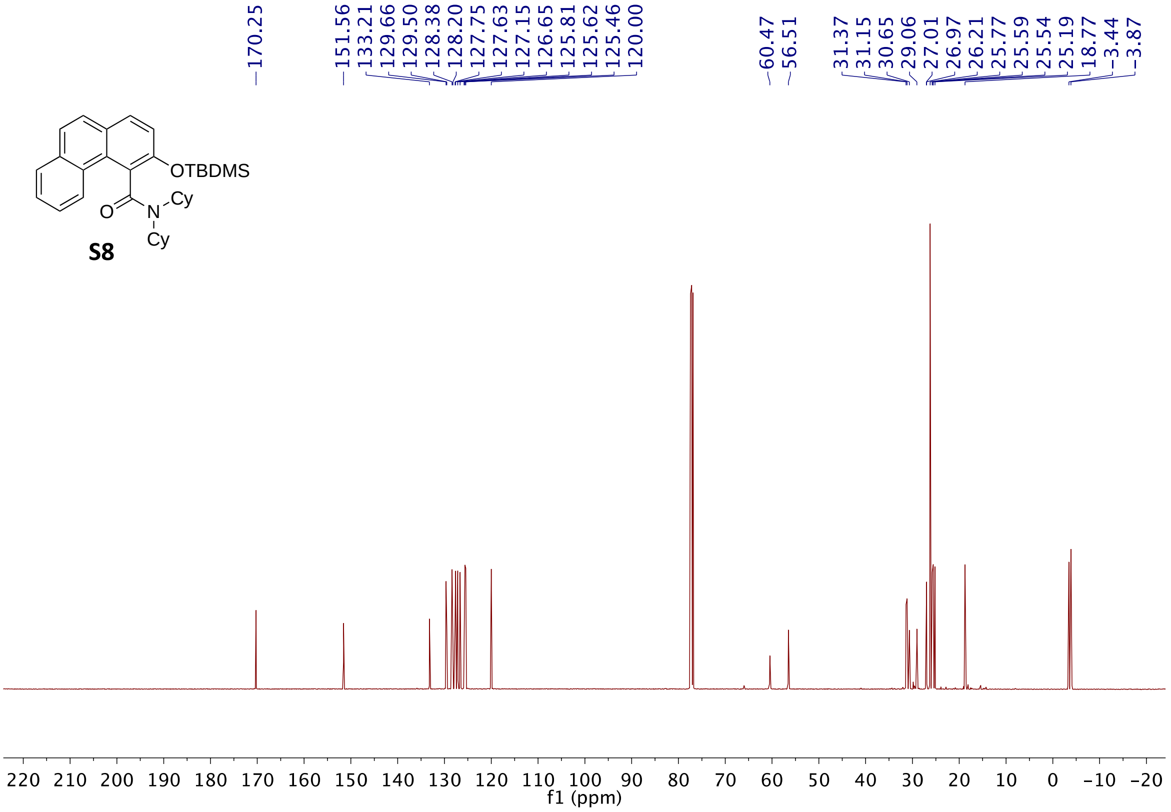
170.25
151.56
133.21
129.66
129.50
128.38
128.20
127.75
127.63
127.15
126.65
125.81
125.62
125.46
120.00
60.47
56.51
31.37
31.15
30.65
29.06
27.01
26.97
26.21
25.77
25.59
25.54
25.19
18.77
-3.44
-3.87
OTBDMS
O
N
Cy
Cy
S8
220 210 200 190 180 170 160 150 140 130 120 110 100 90 80 70 60 50 40 30 20 10 0 -10 -20
f1 (ppm)

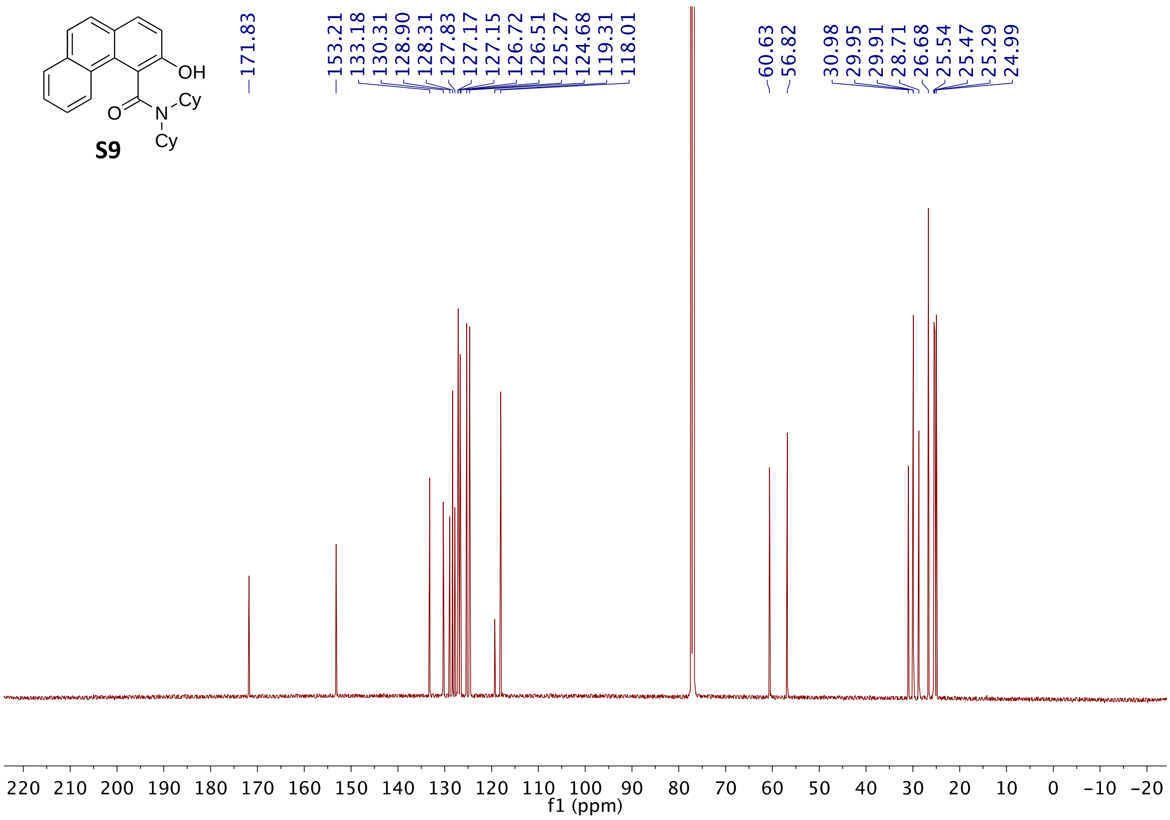
OH
O
N
Cy
Cy
S9
171.83
153.21
133.18
130.31
128.90
128.31
127.83
127.17
127.15
126.72
126.51
125.27
124.68
119.31
118.01
60.63
56.82
30.98
29.95
29.91
28.71
26.68
25.54
25.47
25.29
24.99
220 210 200 190 180 170 160 150 140 130 120 110 100 90 80 70 60 50 40 30 20 10 0 −10 −20
f1 (ppm)

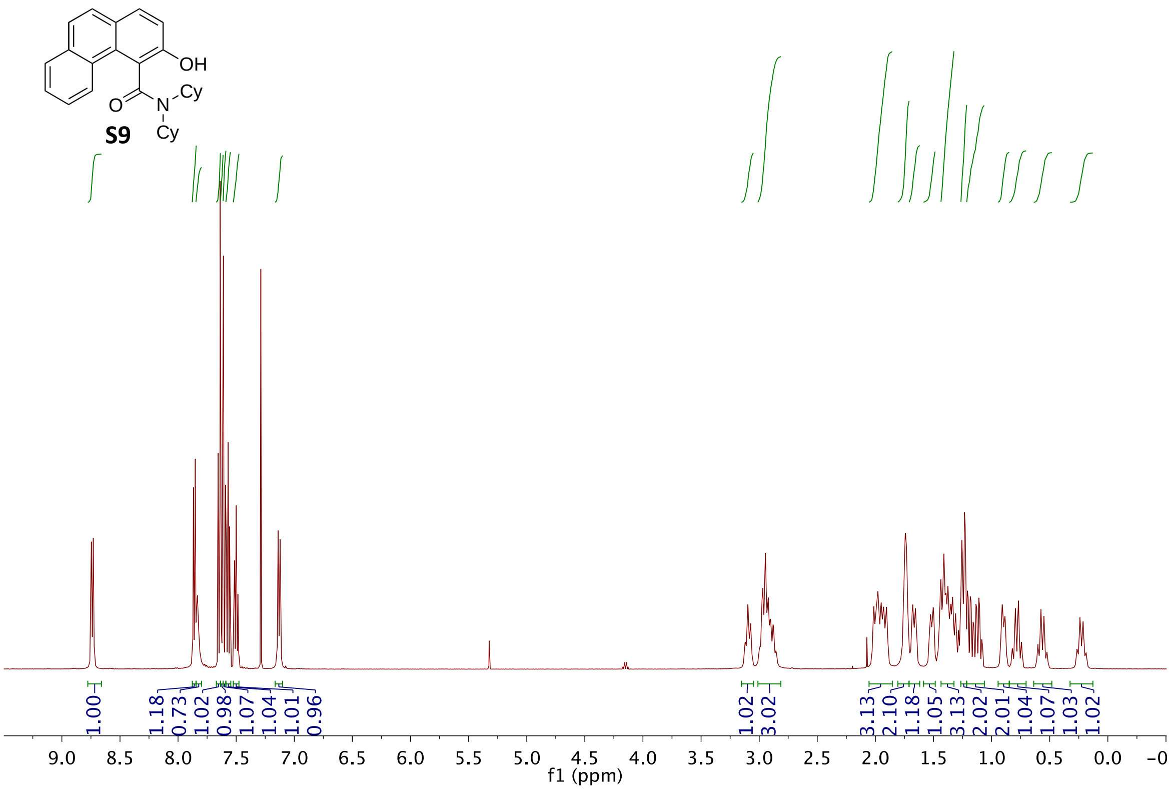
OH
O
N
Cy
Cy
S9
1.00
1.18
0.73
1.02
0.98
1.07
1.04
1.01
0.96
1.02
3.02
3.13
2.10
1.18
1.05
3.13
2.02
2.01
1.04
1.07
1.03
1.02
9.0 8.5 8.0 7.5 7.0 6.5 6.0 5.5 5.0 4.5 4.0 3.5 3.0 2.5 2.0 1.5 1.0 0.5 0.0 −0
f1 (ppm)

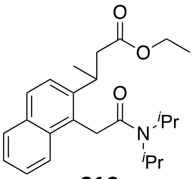

S10

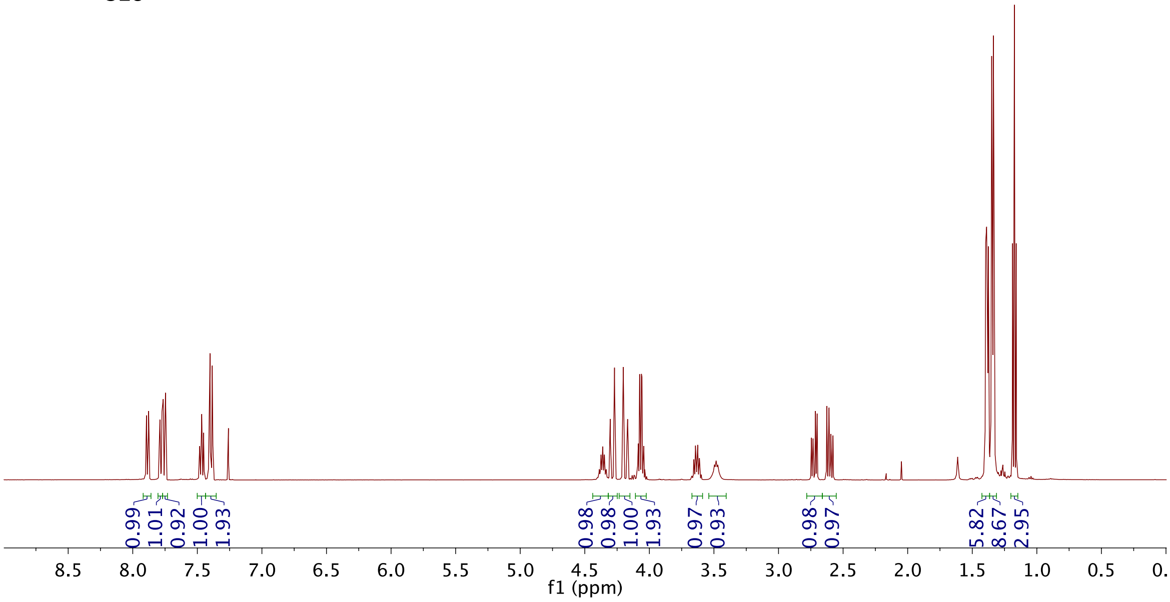
0.99
1.01
0.92
1.00
1.93
0.98
0.98
1.00
1.93
0.97
0.93
0.98
0.97
5.82
8.67
2.95
8.5 8.0 7.5 7.0 6.5 6.0 5.5 5.0 4.5 4.0 3.5 3.0 2.5 2.0 1.5 1.0 0.5 0.
f1 (ppm)

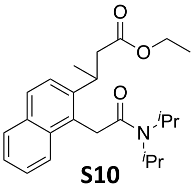

S10

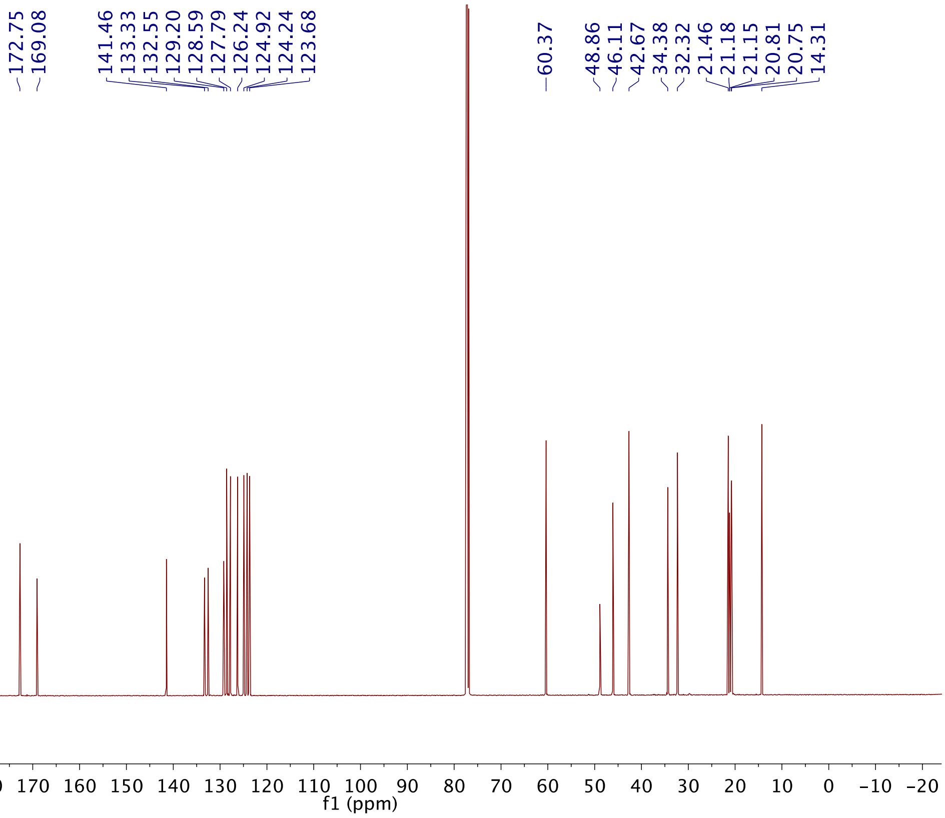
172.75
169.08
141.46
133.33
132.55
129.20
128.59
127.79
126.24
124.92
124.24
123.68
60.37
48.86
46.11
42.67
34.38
32.32
21.46
21.18
21.15
20.81
20.75
14.31
220 210 200 190 180 170 160 150 140 130 120 110 100 90 80 70 60 50 40 30 20 10 0 −10 −20
f1 (ppm)

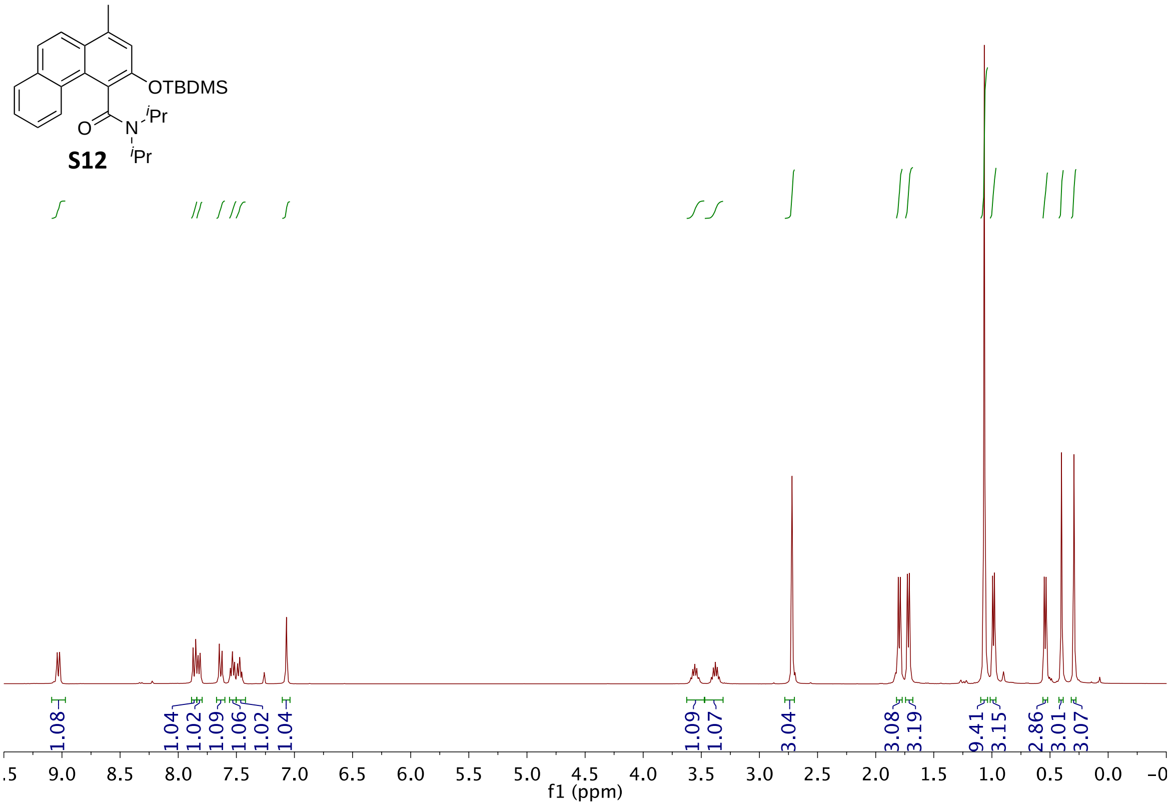
OTBDMS
O
N
^{i}Pr
^{i}Pr
S12
1.08
1.04
1.02
1.09
1.06
1.02
1.04
1.09
1.07
3.04
3.08
3.19
9.41
3.15
2.86
3.01
3.07
f1 (ppm)

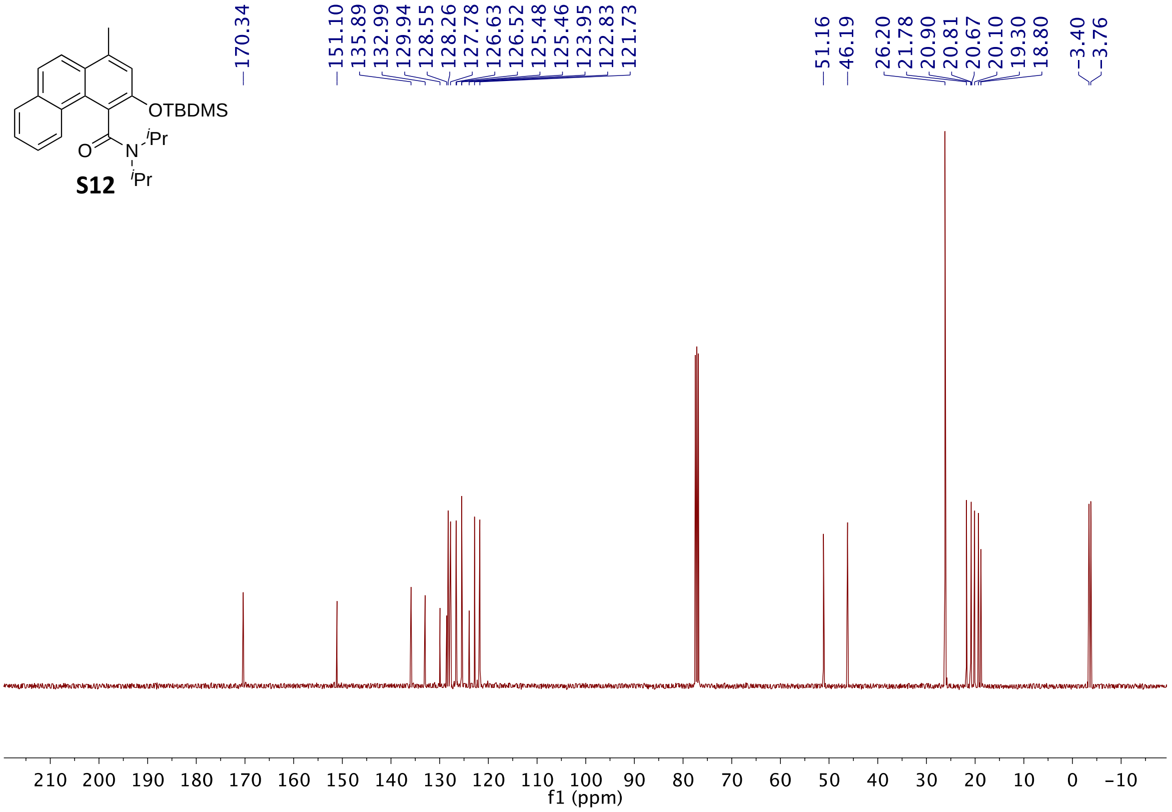
OTBDMS
O
N
^{i}Pr
^{i}Pr
S12
170.34
151.10
135.89
132.99
129.94
128.55
128.26
127.78
126.63
126.52
125.48
125.46
123.95
122.83
121.73
51.16
46.19
26.20
21.78
20.90
20.81
20.67
20.10
19.30
18.80
-3.40
-3.76
f1 (ppm)

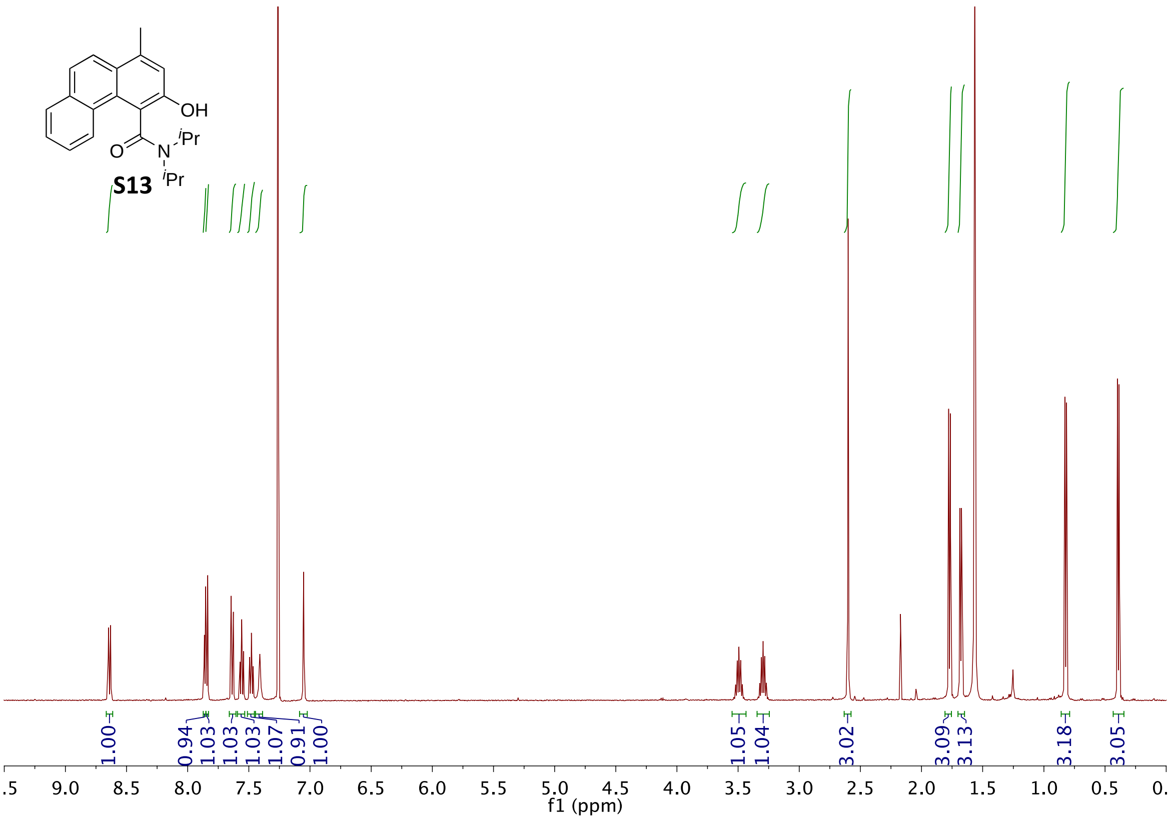
OH
iPr
N
O
iPr
S13
1.00
0.94
1.03
1.03
1.03
1.07
0.91
1.00
1.05
1.04
3.02
3.09
3.13
3.18
3.05
f1 (ppm)

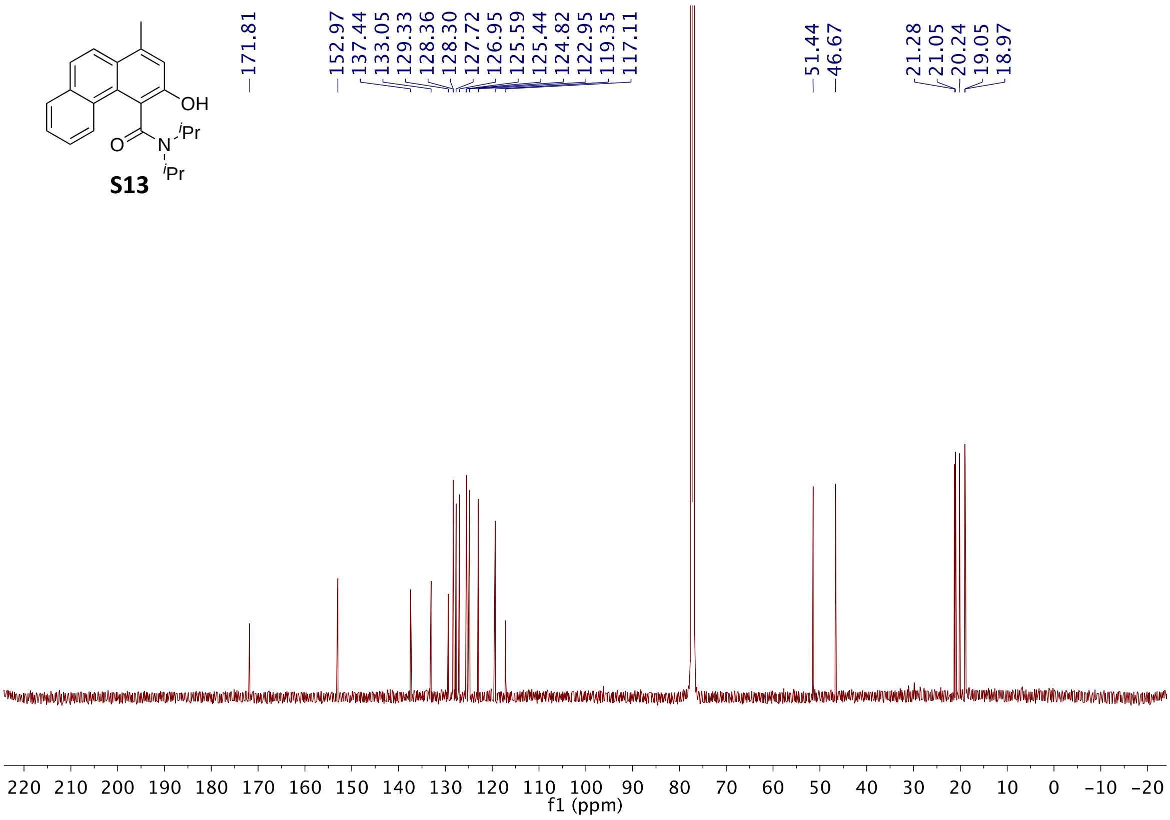
171.81
152.97
137.44
133.05
129.33
128.36
128.30
127.72
126.95
125.59
125.44
124.82
122.95
119.35
117.11
51.44
46.67
21.28
21.05
20.24
19.05
18.97
OH
iPr
N
O
iPr
S13
f1 (ppm)

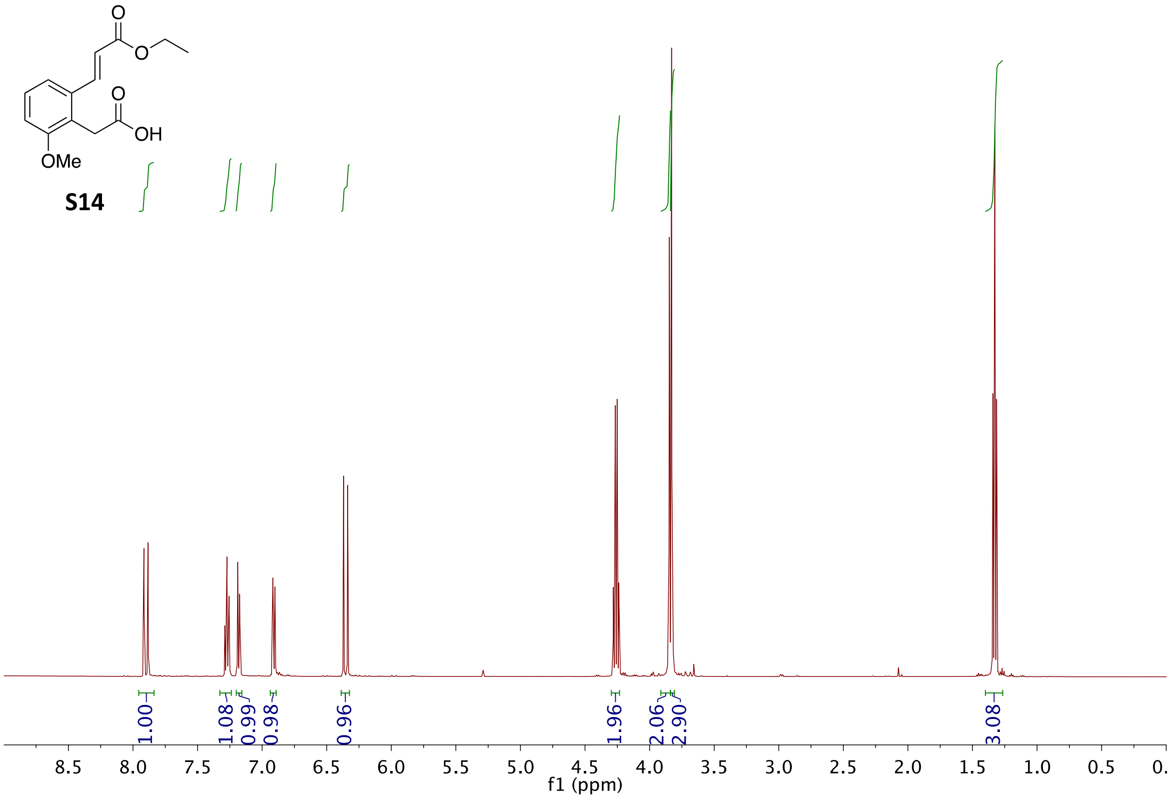
OMe
OH
O
S14
1.00
1.08
0.99
0.98
0.96
1.96
2.06
2.90
3.08
8.5 8.0 7.5 7.0 6.5 6.0 5.5 5.0 4.5 4.0 3.5 3.0 2.5 2.0 1.5 1.0 0.5 0.
f1 (ppm)

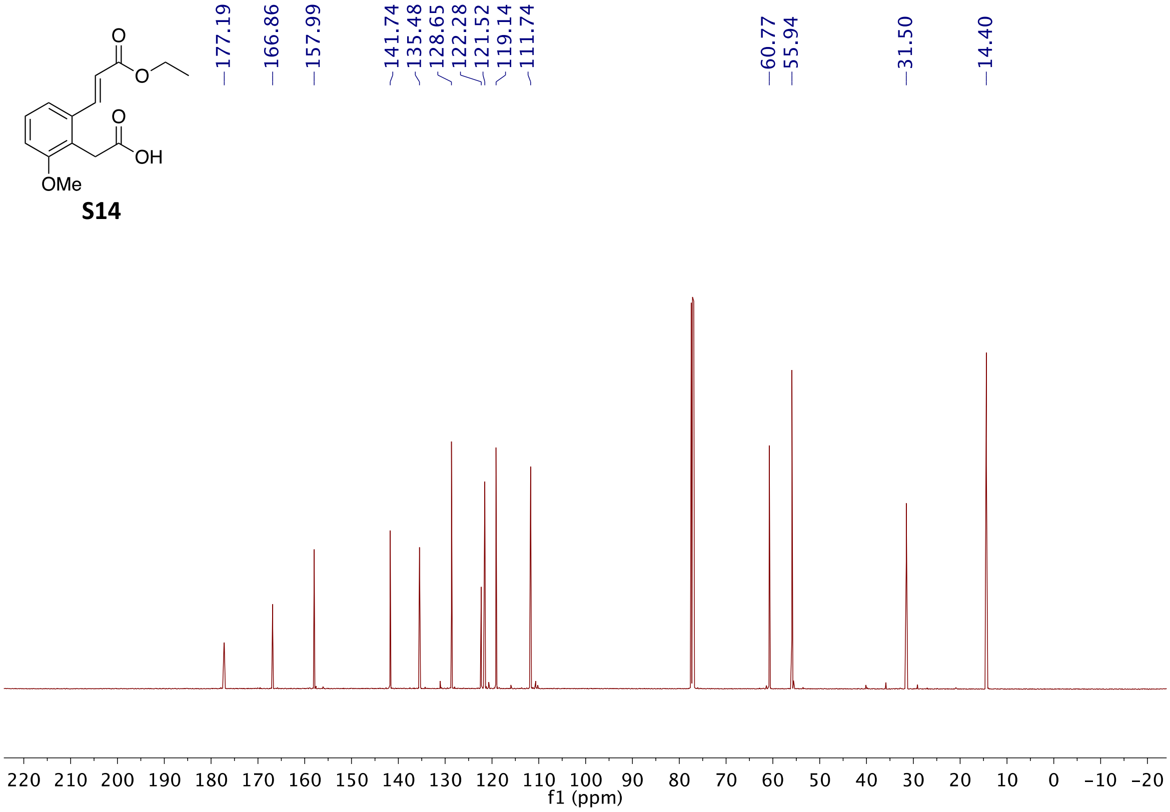
OMe
OH
O
S14
177.19
166.86
157.99
141.74
135.48
128.65
122.28
121.52
119.14
111.74
60.77
55.94
31.50
14.40
220 210 200 190 180 170 160 150 140 130 120 110 100 90 80 70 60 50 40 30 20 10 0 −10 −20
f1 (ppm)

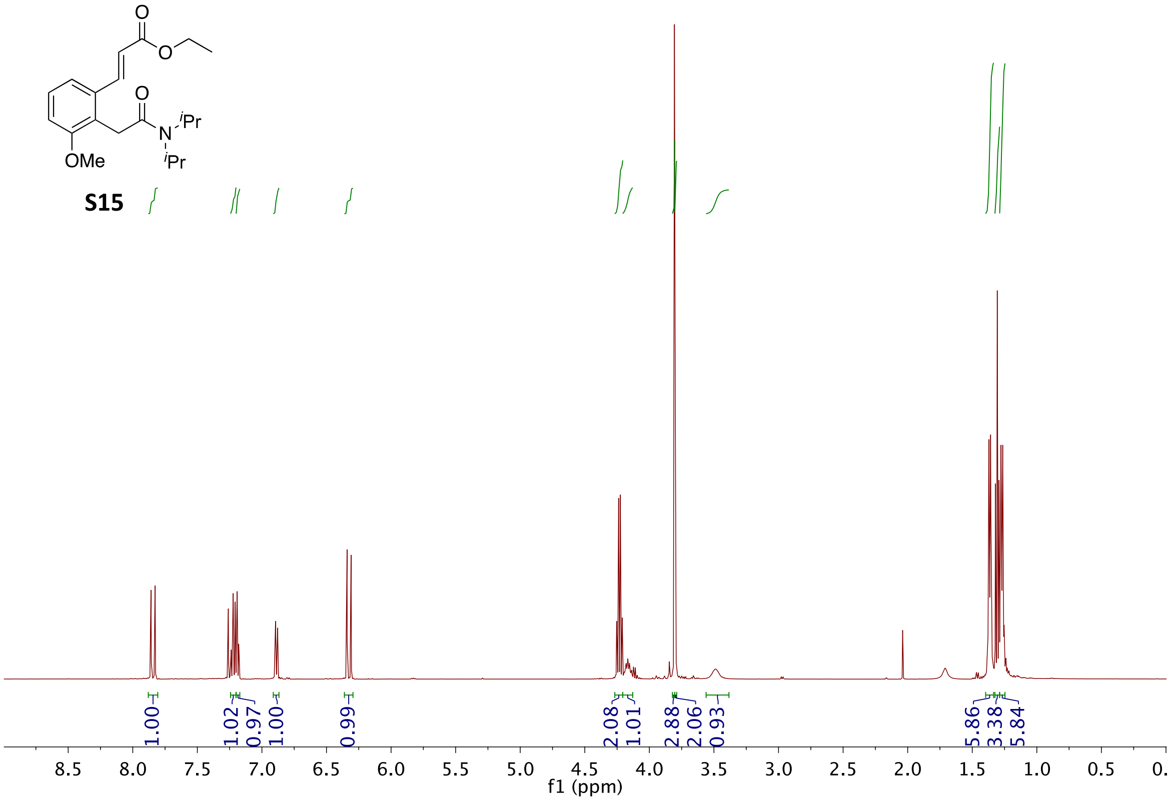

OMe
N
iPr
S15
1.00
1.02
0.97
1.00
0.99
2.08
1.01
2.88
2.06
0.93
5.86
3.38
5.84
8.5 8.0 7.5 7.0 6.5 6.0 5.5 5.0 4.5 4.0 3.5 3.0 2.5 2.0 1.5 1.0 0.5
f1 (ppm)

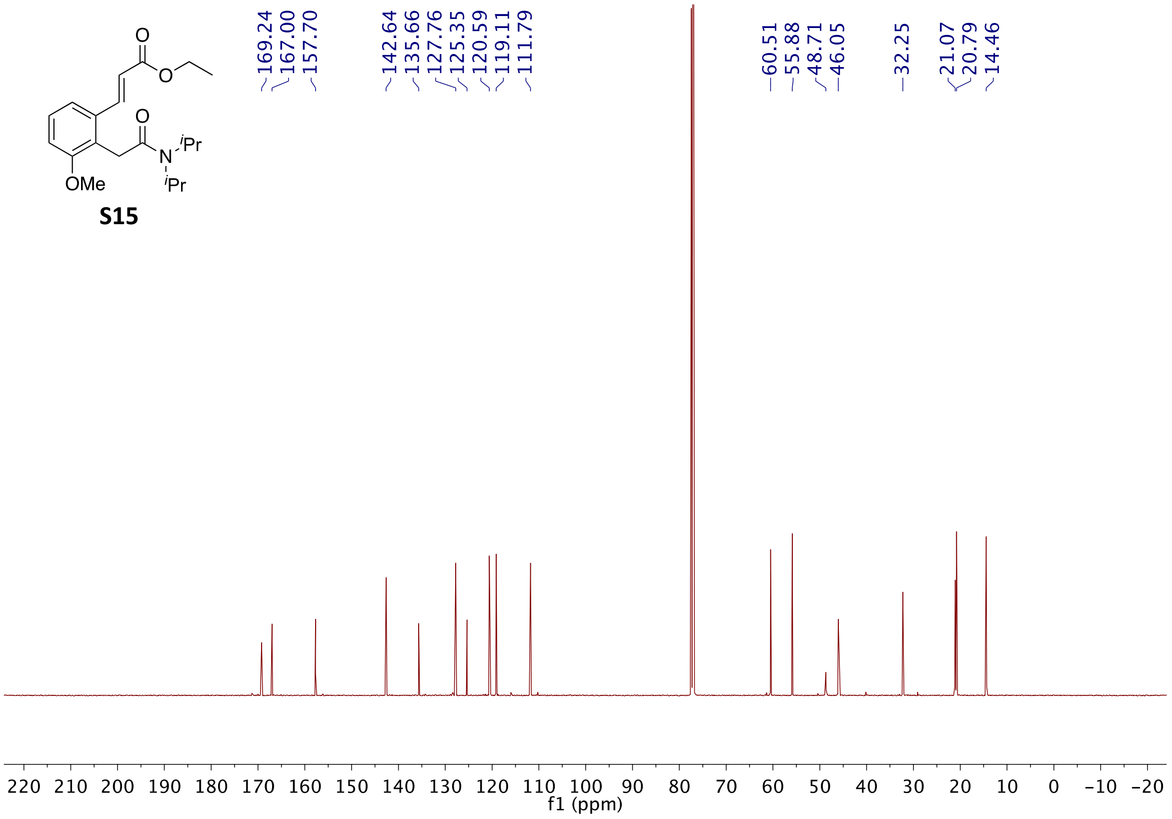

169.24
167.00
157.70
142.64
135.66
127.76
125.35
120.59
119.11
111.79
60.51
55.88
48.71
46.05
32.25
21.07
20.79
14.46
OMe
N
iPr
S15
220 210 200 190 180 170 160 150 140 130 120 110 100 90 80 70 60 50 40 30 20 10 0 −10 −20
f1 (ppm)

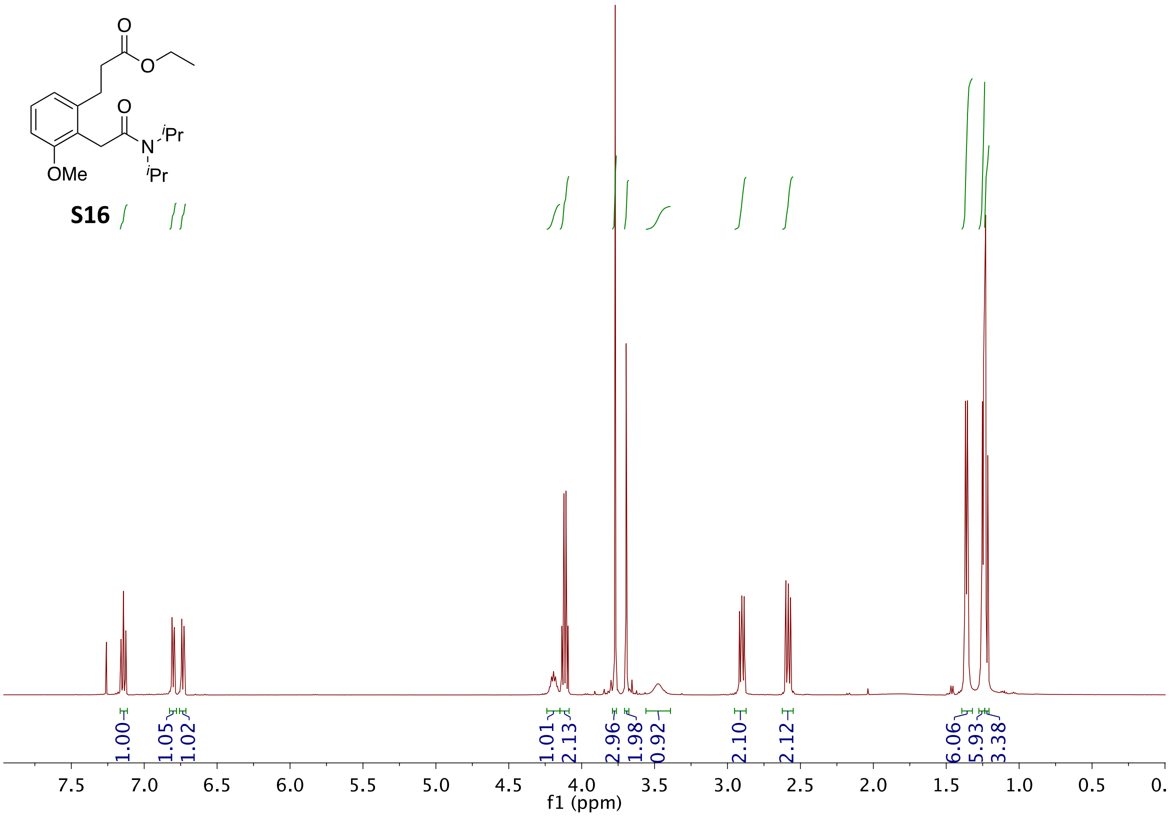
S16
1.00
1.05
1.02
1.01
2.13
2.96
1.98
0.92
2.10
2.12
6.06
5.93
3.38
7.5 7.0 6.5 6.0 5.5 5.0 4.5 4.0 3.5 3.0 2.5 2.0 1.5 1.0 0.5 0.
f1 (ppm)

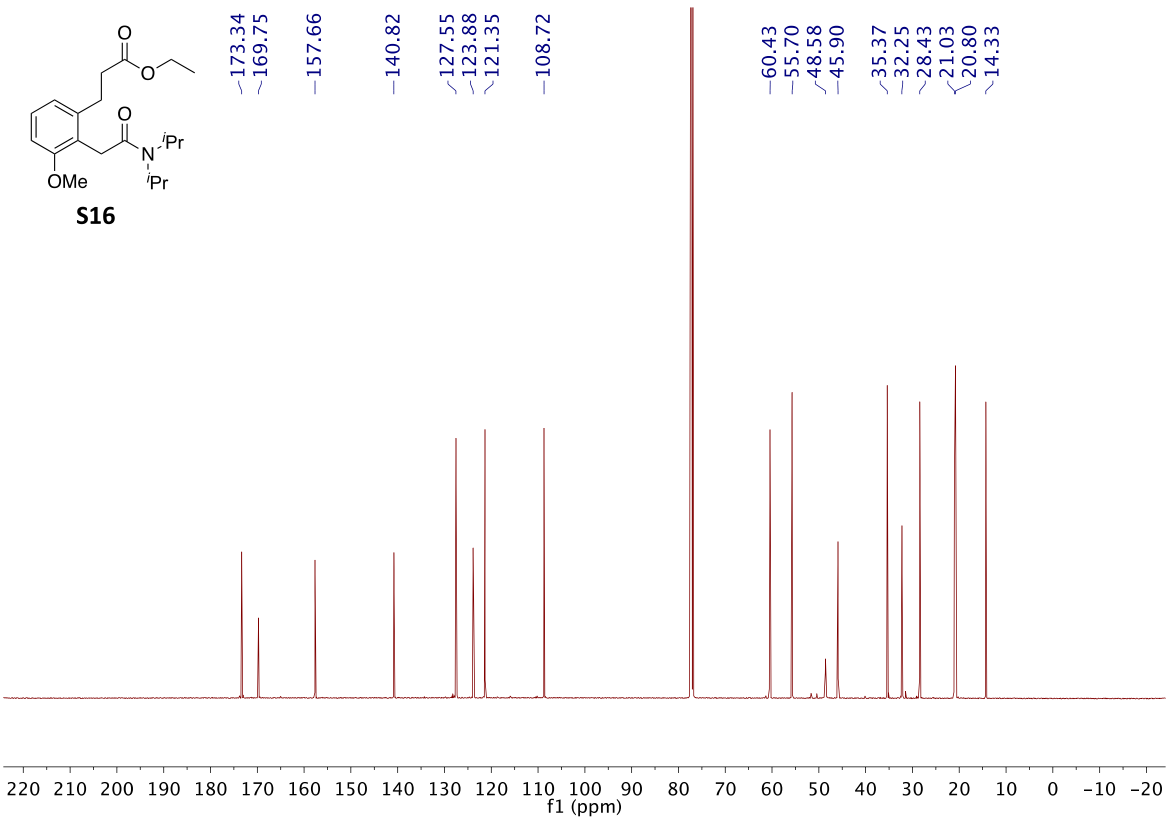
S16
173.34
169.75
157.66
140.82
127.55
123.88
121.35
108.72
60.43
55.70
48.58
45.90
35.37
32.25
28.43
21.03
20.80
14.33
220 210 200 190 180 170 160 150 140 130 120 110 100 90 80 70 60 50 40 30 20 10 0 -10 -20
f1 (ppm)

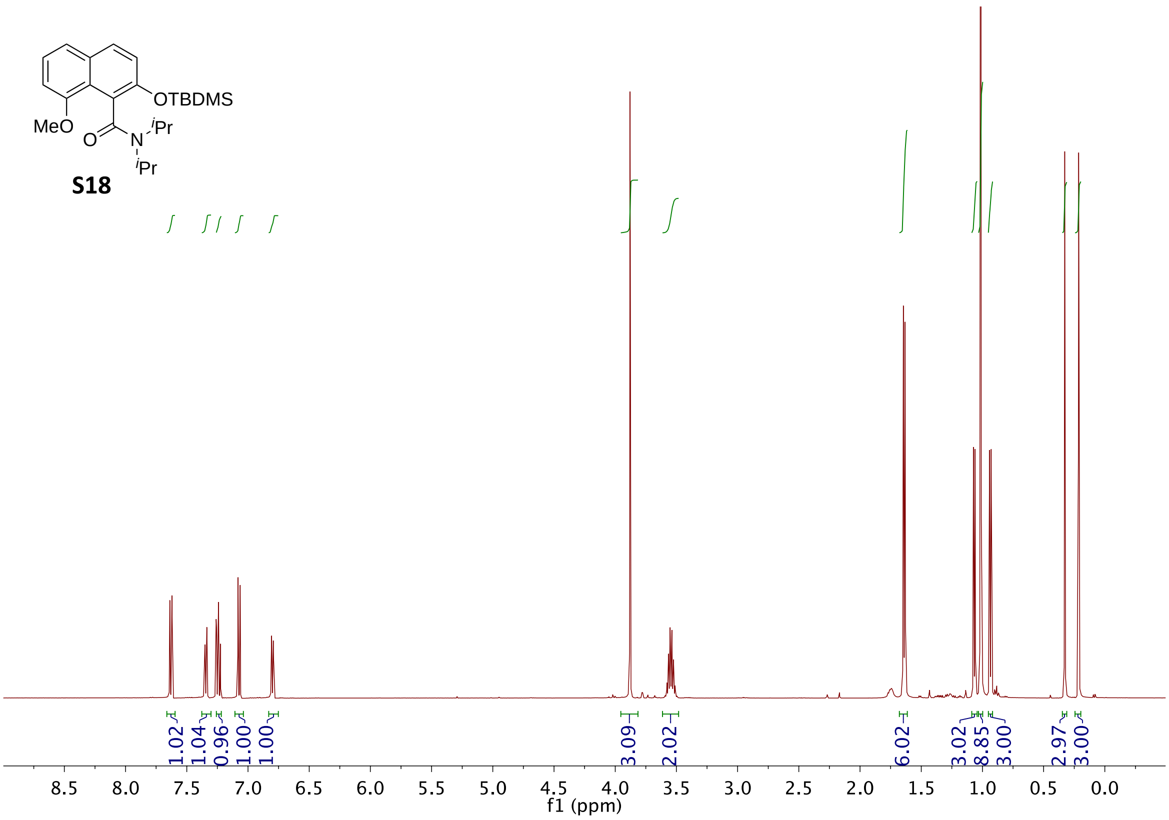
OTBDMS
MeO
O
N
^{i}Pr
^{i}Pr
S18
1.02
1.04
0.96
1.00
1.00
3.09
2.02
6.02
3.02
8.85
3.00
2.97
3.00
8.5 8.0 7.5 7.0 6.5 6.0 5.5 5.0 4.5 4.0 3.5 3.0 2.5 2.0 1.5 1.0 0.5 0.0
f1 (ppm)

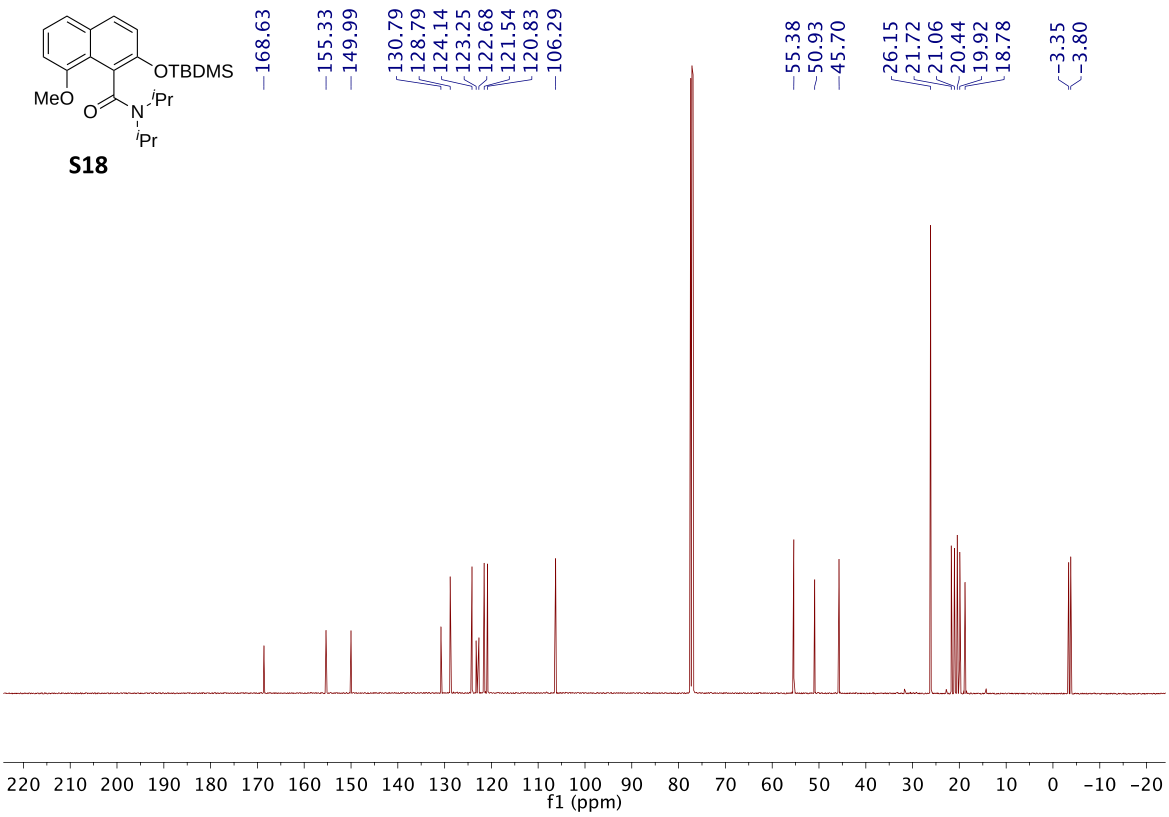
OTBDMS
MeO
O
N
^{i}Pr
^{i}Pr
S18
168.63
155.33
149.99
130.79
128.79
124.14
123.25
122.68
121.54
120.83
106.29
55.38
50.93
45.70
26.15
21.72
21.06
20.44
19.92
18.78
-3.35
-3.80
220 210 200 190 180 170 160 150 140 130 120 110 100 90 80 70 60 50 40 30 20 10 0 -10 -20
f1 (ppm)

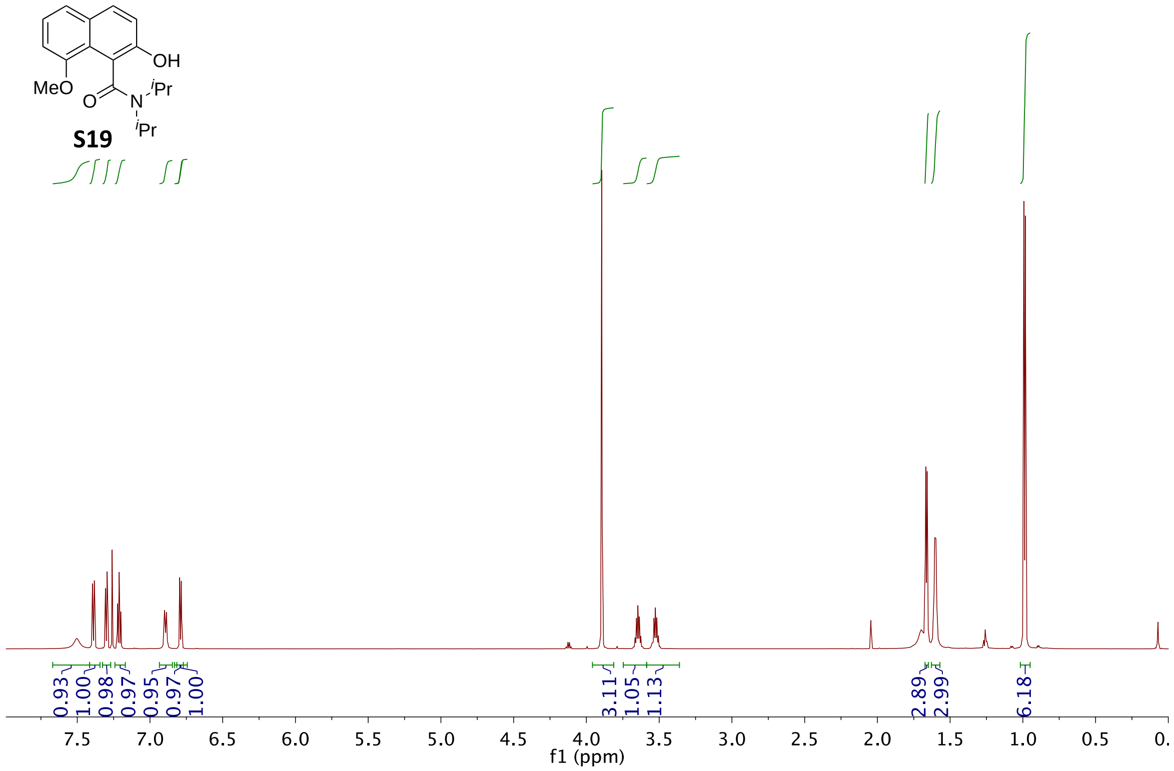
MeO
OH
O
N
iPr
iPr
S19
0.93
1.00
0.98
0.97
0.95
0.97
1.00
3.11
1.05
1.13
2.89
2.99
6.18
7.5 7.0 6.5 6.0 5.5 5.0 4.5 4.0 3.5 3.0 2.5 2.0 1.5 1.0 0.5 0.
f1 (ppm)

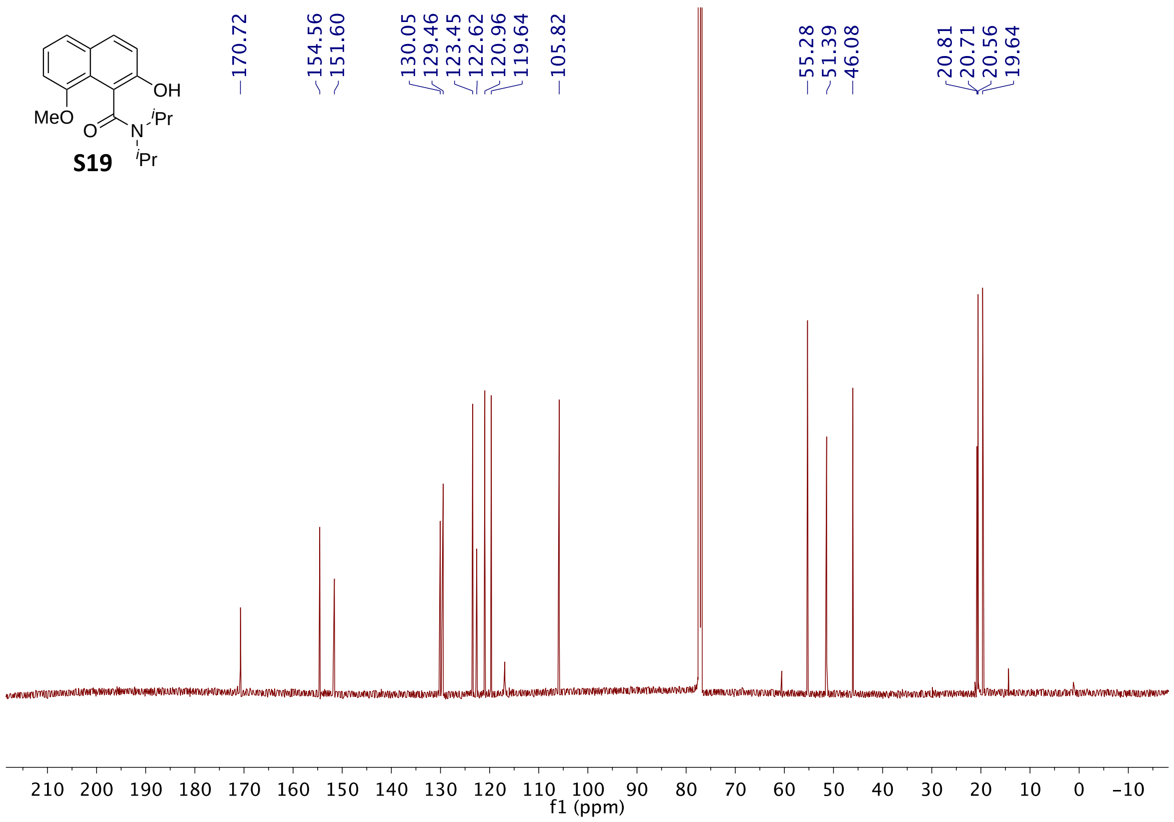
MeO
OH
O
N
iPr
iPr
S19
170.72
154.56
151.60
130.05
129.46
123.45
122.62
120.96
119.64
105.82
55.28
51.39
46.08
20.81
20.71
20.56
19.64
210 200 190 180 170 160 150 140 130 120 110 100 90 80 70 60 50 40 30 20 10 0 -10
f1 (ppm)

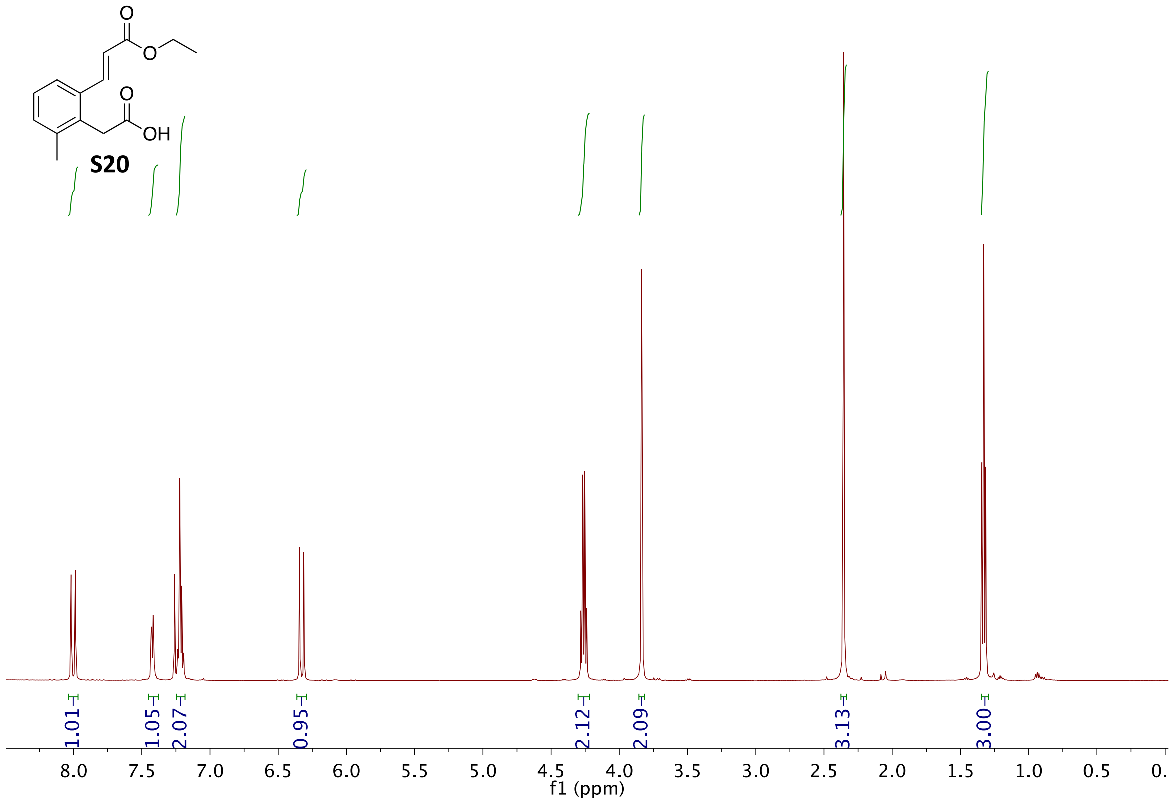
S20
1.01
1.05
2.07
0.95
2.12
2.09
3.13
3.00
8.0 7.5 7.0 6.5 6.0 5.5 5.0 4.5 4.0 3.5 3.0 2.5 2.0 1.5 1.0 0.5 0.
f1 (ppm)

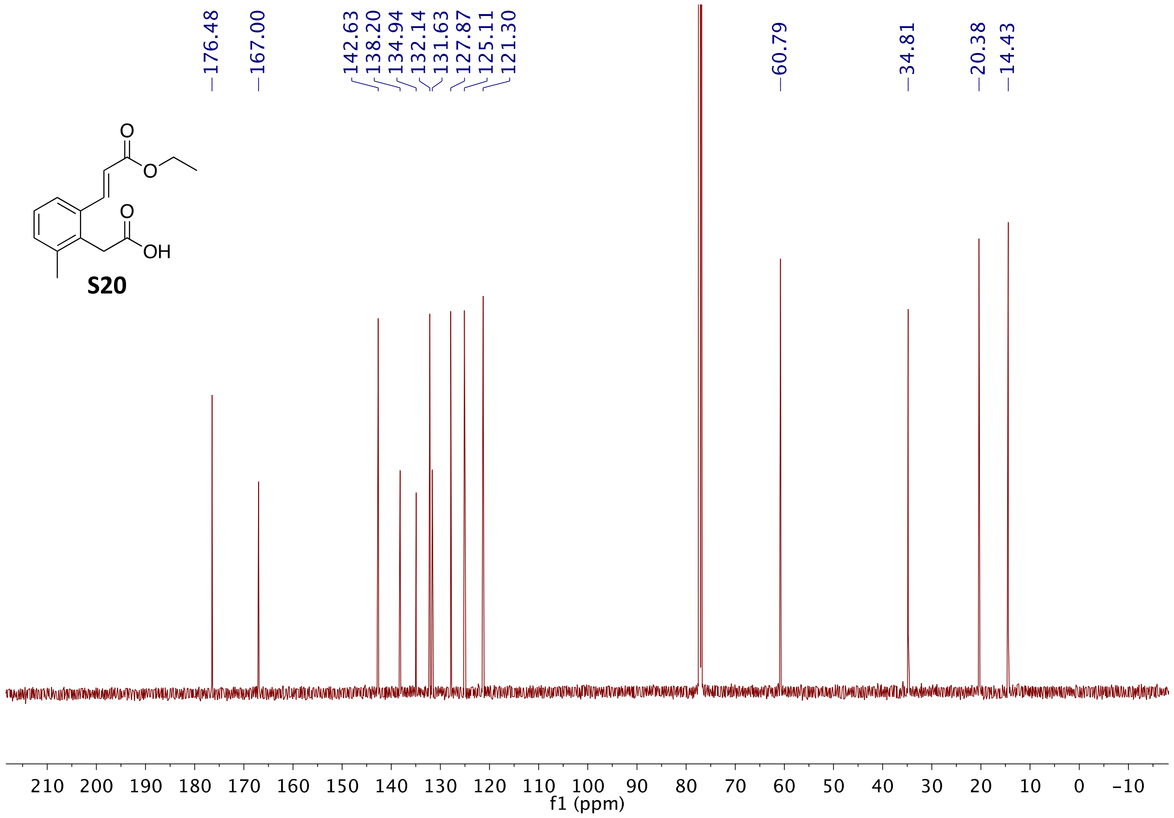
176.48
167.00
142.63
138.20
134.94
132.14
131.63
127.87
125.11
121.30
60.79
34.81
20.38
14.43
S20
210 200 190 180 170 160 150 140 130 120 110 100 90 80 70 60 50 40 30 20 10 0 -10
f1 (ppm)

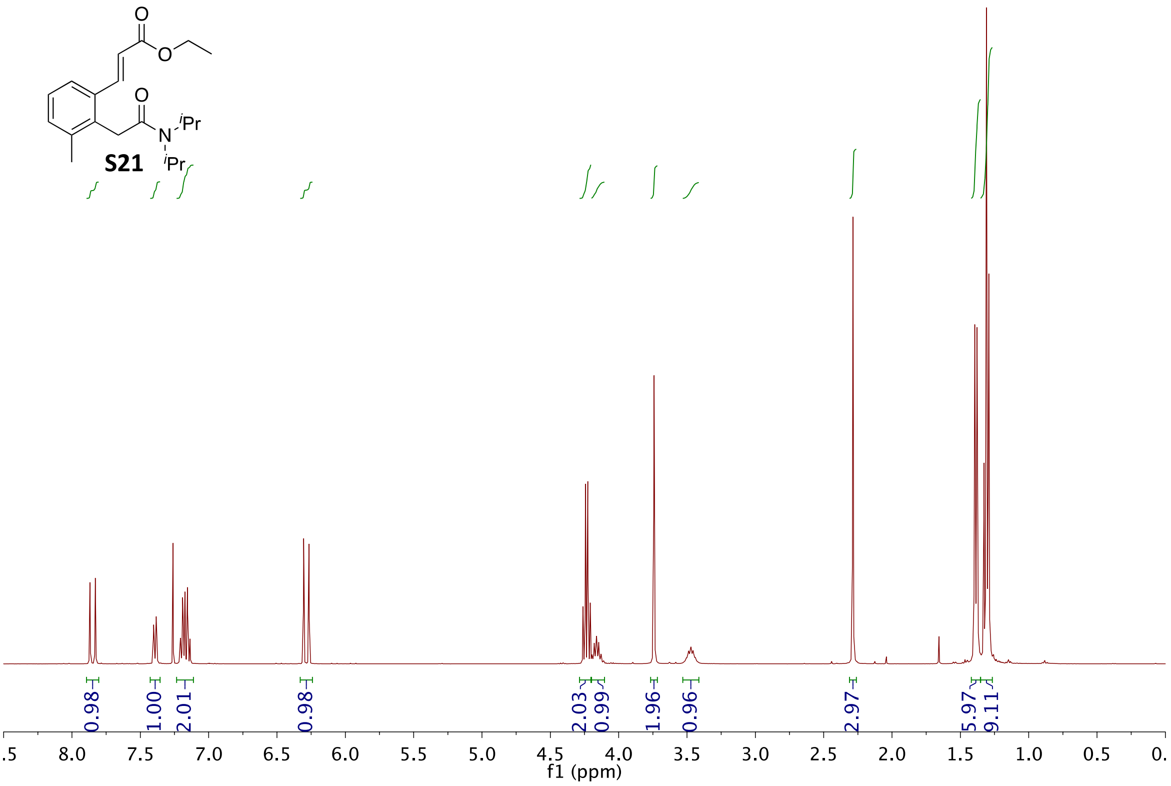
S21
iPr
iPr
0.98
1.00
2.01
0.98
2.03
0.99
1.96
0.96
2.97
5.97
9.11
.5 8.0 7.5 7.0 6.5 6.0 5.5 5.0 4.5 4.0 3.5 3.0 2.5 2.0 1.5 1.0 0.5 0.
f1 (ppm)

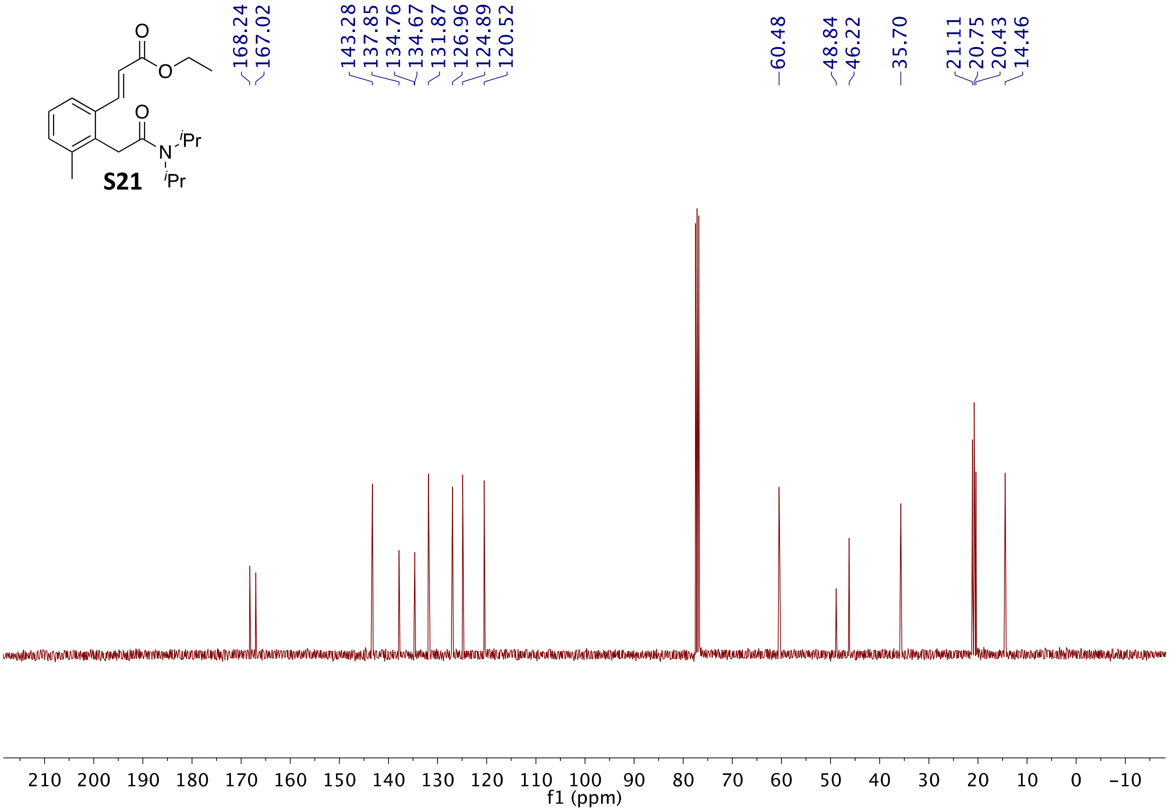
S21
iPr
iPr
168.24
167.02
143.28
137.85
134.76
134.67
131.87
126.96
124.89
120.52
60.48
48.84
46.22
35.70
21.11
20.75
20.43
14.46
210 200 190 180 170 160 150 140 130 120 110 100 90 80 70 60 50 40 30 20 10 0 -10
f1 (ppm)

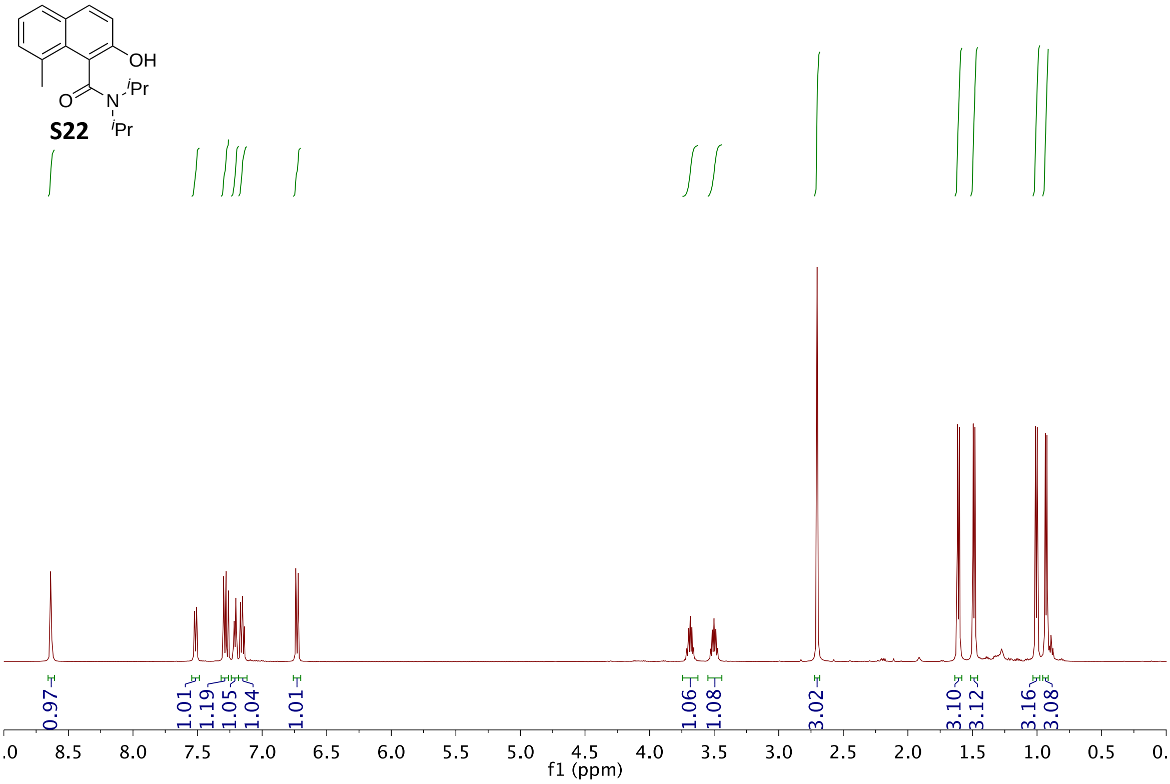
OH
iPr
O
N
iPr
S22
0.97
1.01
1.19
1.05
1.04
1.01
1.06
1.08
3.02
3.10
3.12
3.16
3.08
.0 8.5 8.0 7.5 7.0 6.5 6.0 5.5 5.0 4.5 4.0 3.5 3.0 2.5 2.0 1.5 1.0 0.5 0.
f1 (ppm)

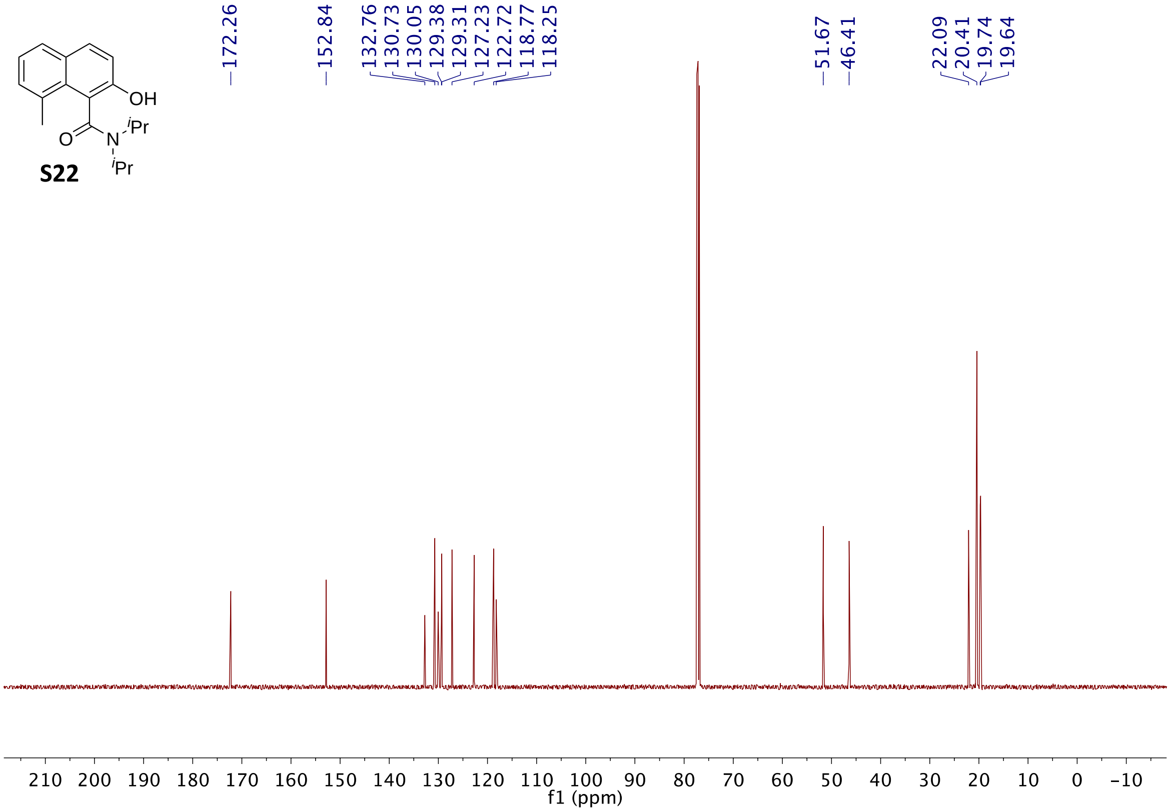
OH
iPr
O
N
iPr
S22
172.26
152.84
132.76
130.73
130.05
129.38
129.31
127.23
122.72
118.77
118.25
51.67
46.41
22.09
20.41
19.74
19.64
210 200 190 180 170 160 150 140 130 120 110 100 90 80 70 60 50 40 30 20 10 0 −10
f1 (ppm)

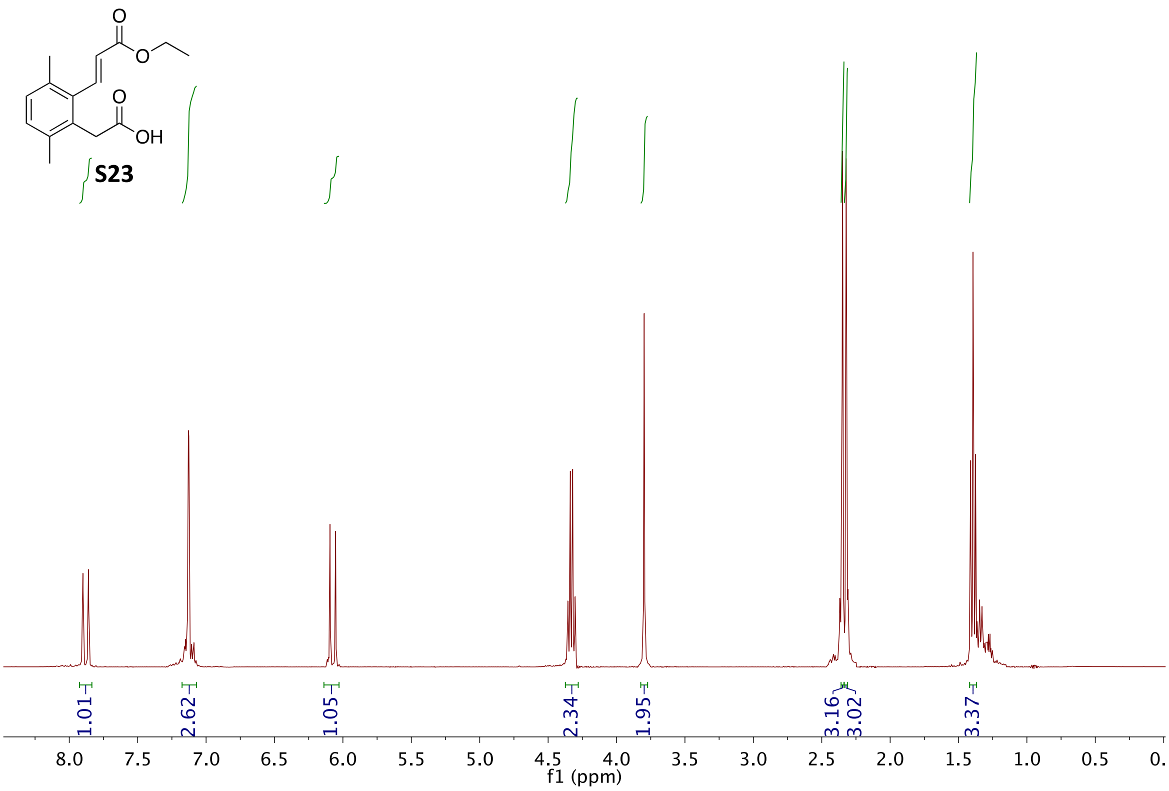
O
O
O
OH
1.01
2.62
1.05
2.34
1.95
3.16
3.02
3.37
8.0 7.5 7.0 6.5 6.0 5.5 5.0 4.5 4.0 3.5 3.0 2.5 2.0 1.5 1.0 0.5 0.
f1 (ppm)

S23

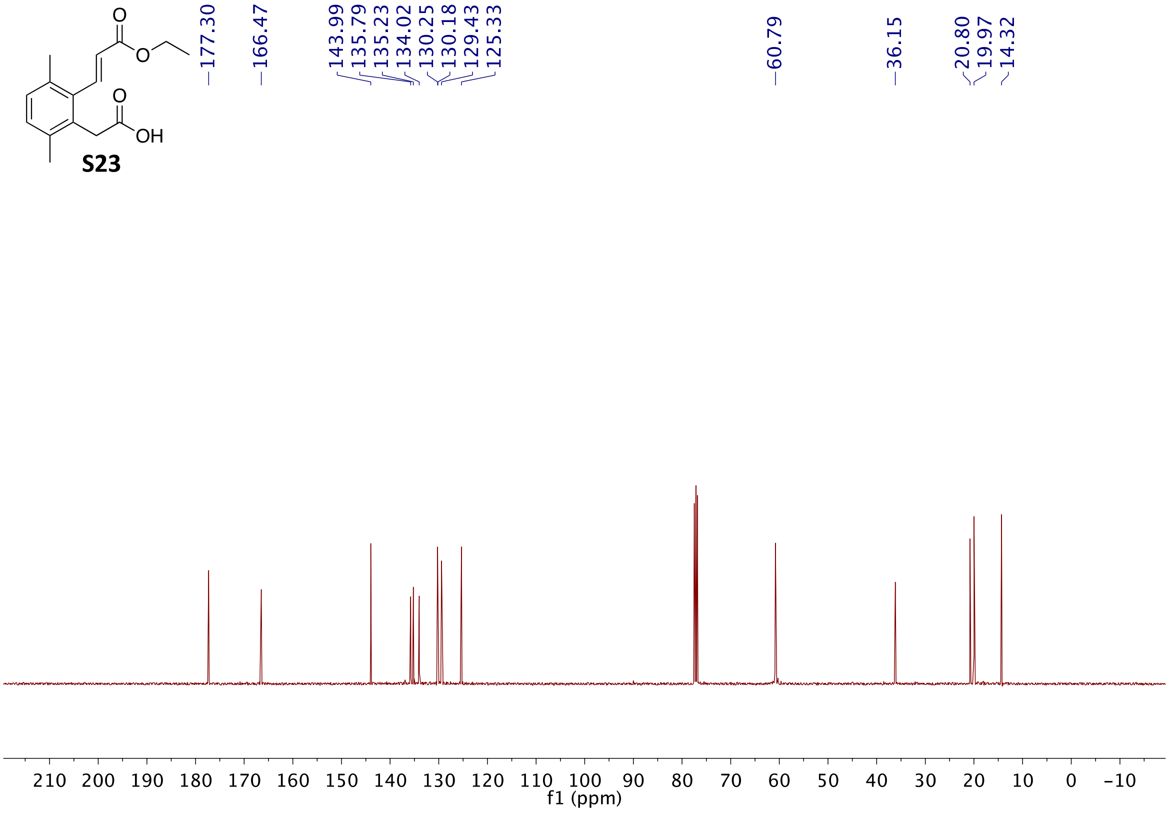
O
O
O
OH
177.30
166.47
143.99
135.79
135.23
134.02
130.25
130.18
129.43
125.33
60.79
36.15
20.80
19.97
14.32
210 200 190 180 170 160 150 140 130 120 110 100 90 80 70 60 50 40 30 20 10 0 −10
f1 (ppm)

S23

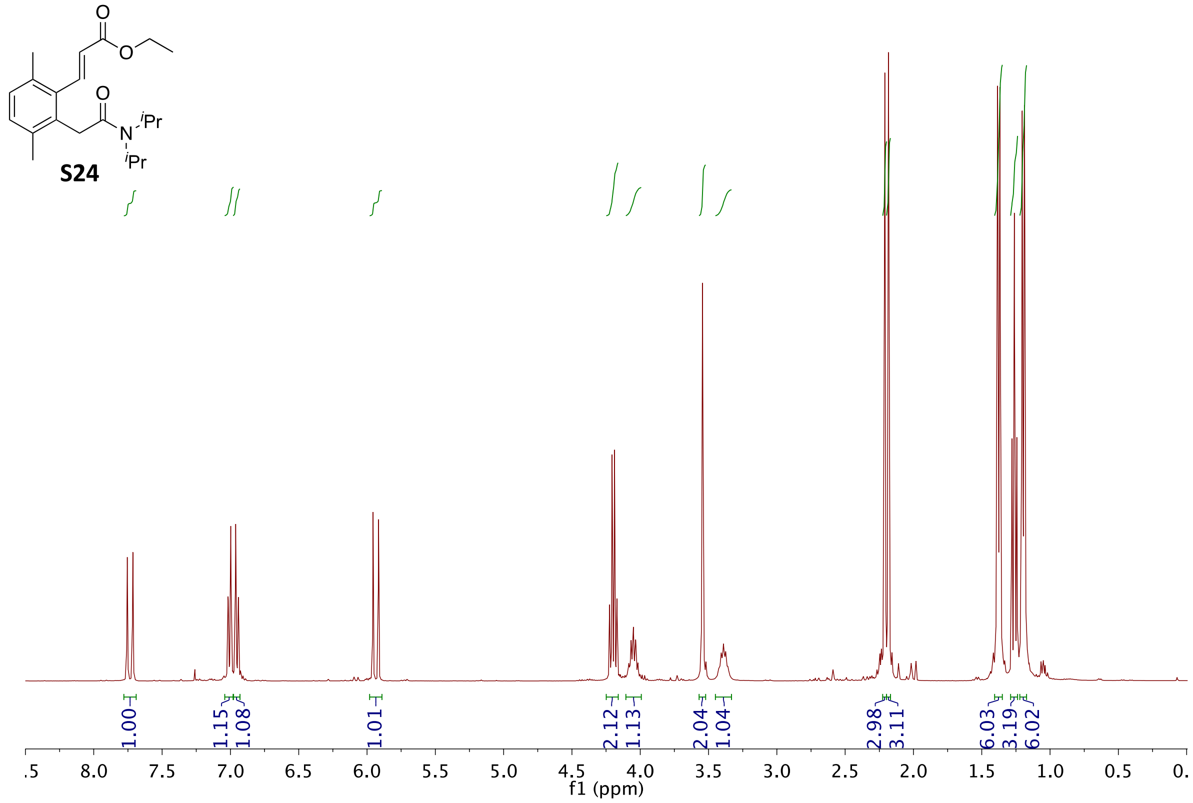
O
O
O
N
iPr
iPr
S24
1.00
1.15
1.08
1.01
2.12
1.13
2.04
1.04
2.98
3.11
6.03
3.19
6.02
f1 (ppm)

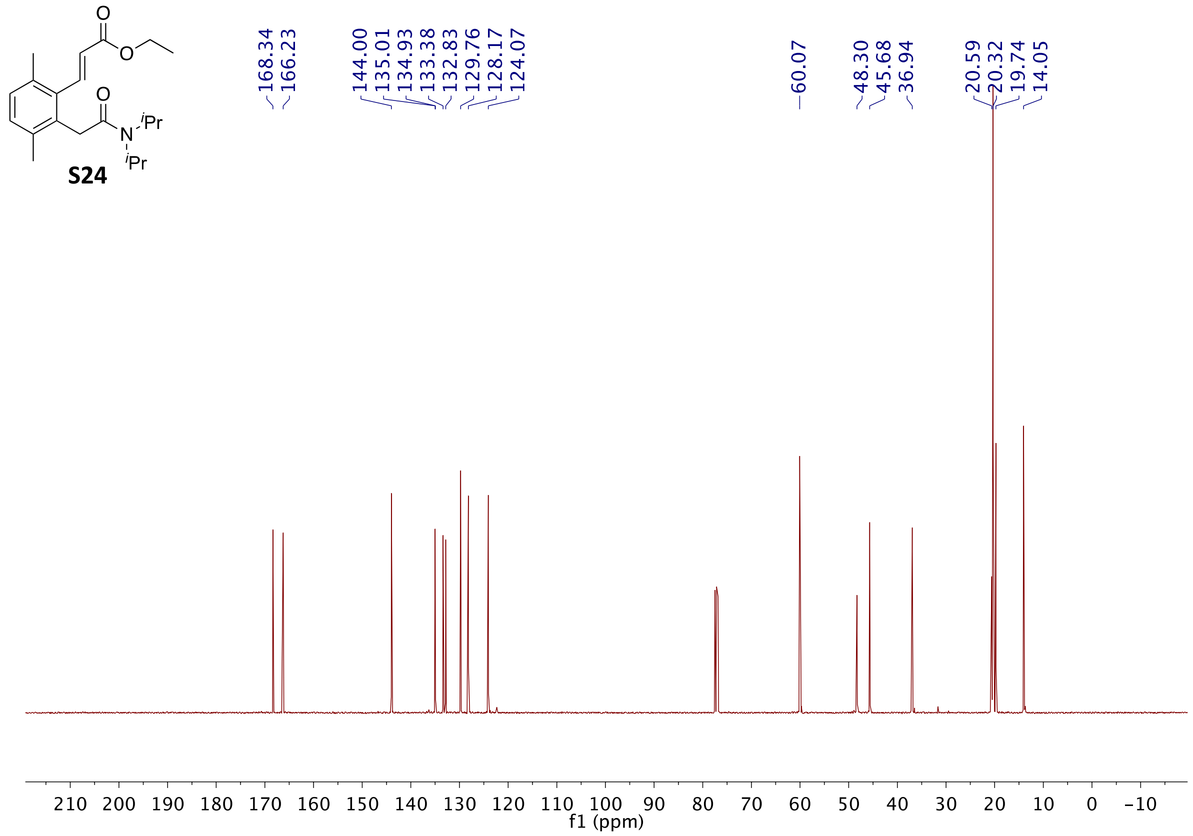
O
O
O
N
iPr
iPr
S24
168.34
166.23
144.00
135.01
134.93
133.38
132.83
129.76
128.17
124.07
60.07
48.30
45.68
36.94
20.59
20.32
19.74
14.05
f1 (ppm)

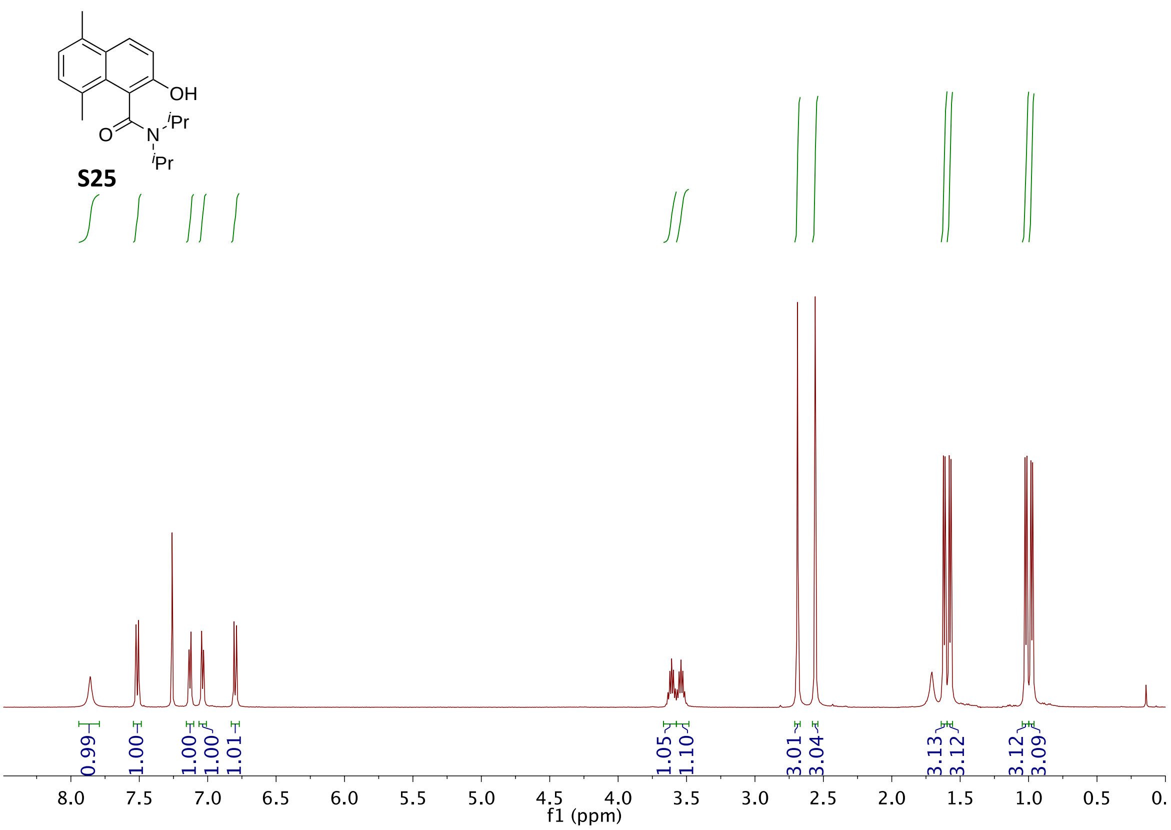
OH
iPr
N
O
iPr
0.99
1.00
1.00
1.00
1.01
1.05
1.10
3.01
3.04
3.13
3.12
3.12
3.09
8.0 7.5 7.0 6.5 6.0 5.5 5.0 4.5 4.0 3.5 3.0 2.5 2.0 1.5 1.0 0.5 0.
f1 (ppm)

S25

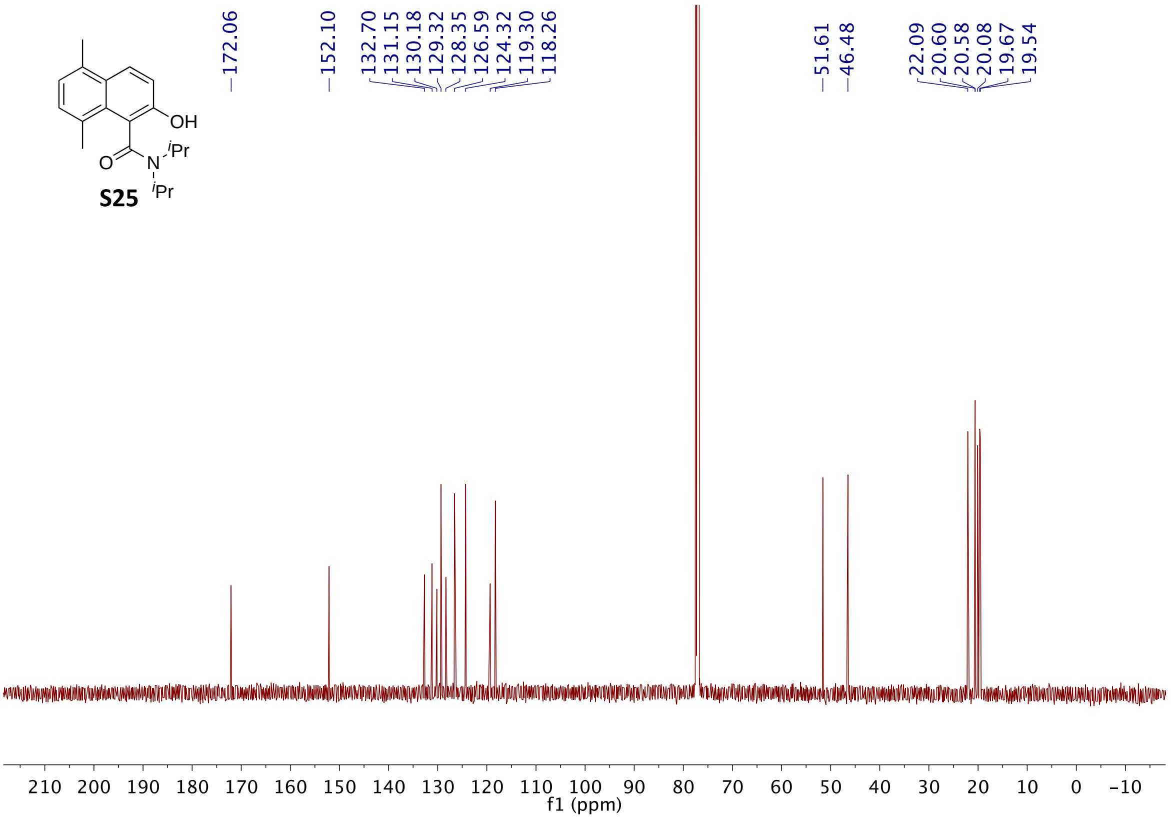
172.06
152.10
132.70
131.15
130.18
129.32
128.35
126.59
124.32
119.30
118.26
51.61
46.48
22.09
20.60
20.58
20.08
19.67
19.54
OH
iPr
N
O
iPr
210 200 190 180 170 160 150 140 130 120 110 100 90 80 70 60 50 40 30 20 10 0 −10
f1 (ppm)

S25

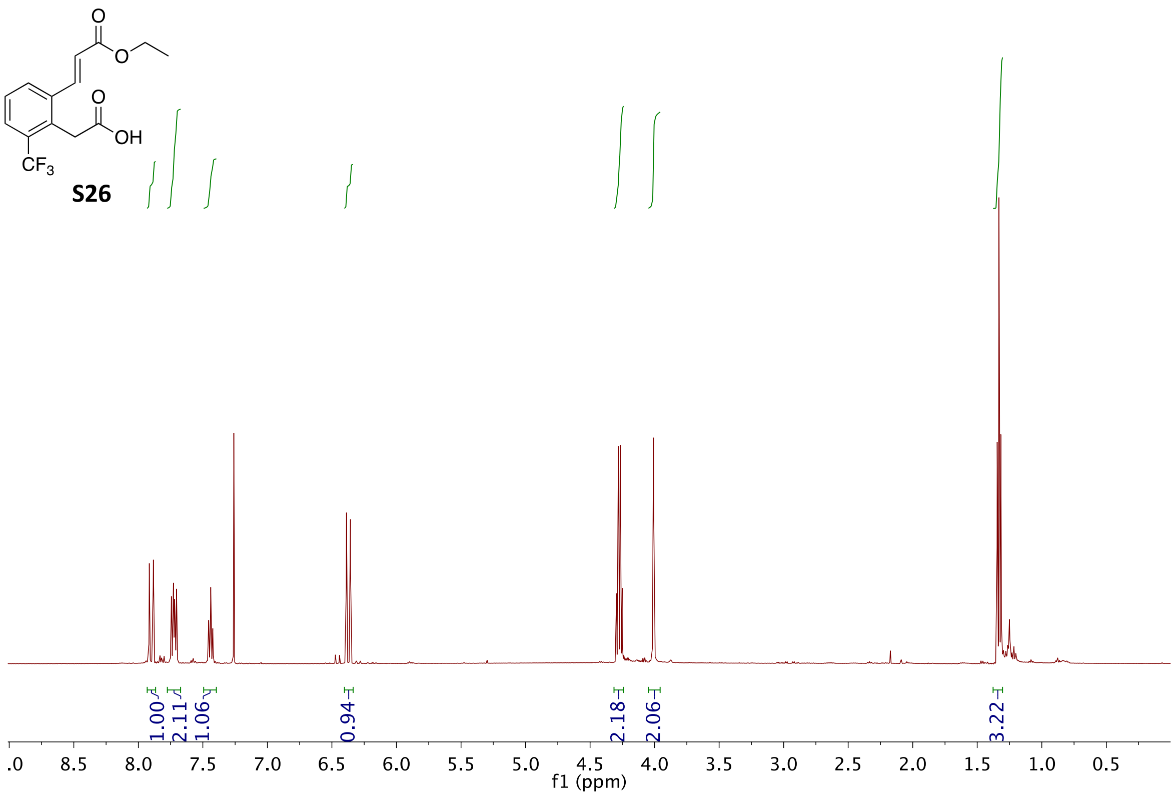
S26
1.00
2.11
1.06
0.94
2.18
2.06
3.22
f1 (ppm)

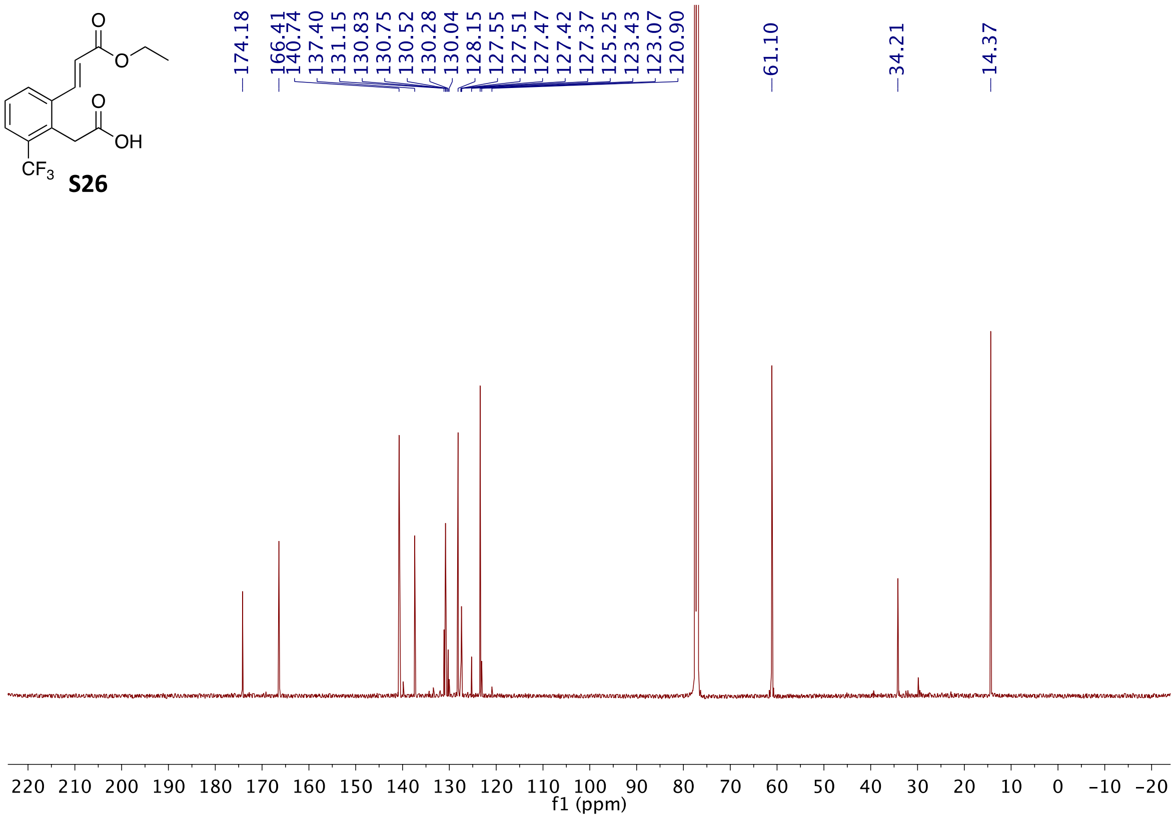
S26
174.18
166.41
140.74
137.40
131.15
130.83
130.75
130.52
130.28
130.04
128.15
127.55
127.51
127.47
127.42
127.37
125.25
123.43
123.07
120.90
61.10
34.21
14.37
f1 (ppm)

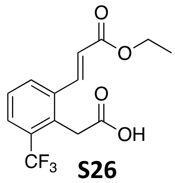

S26

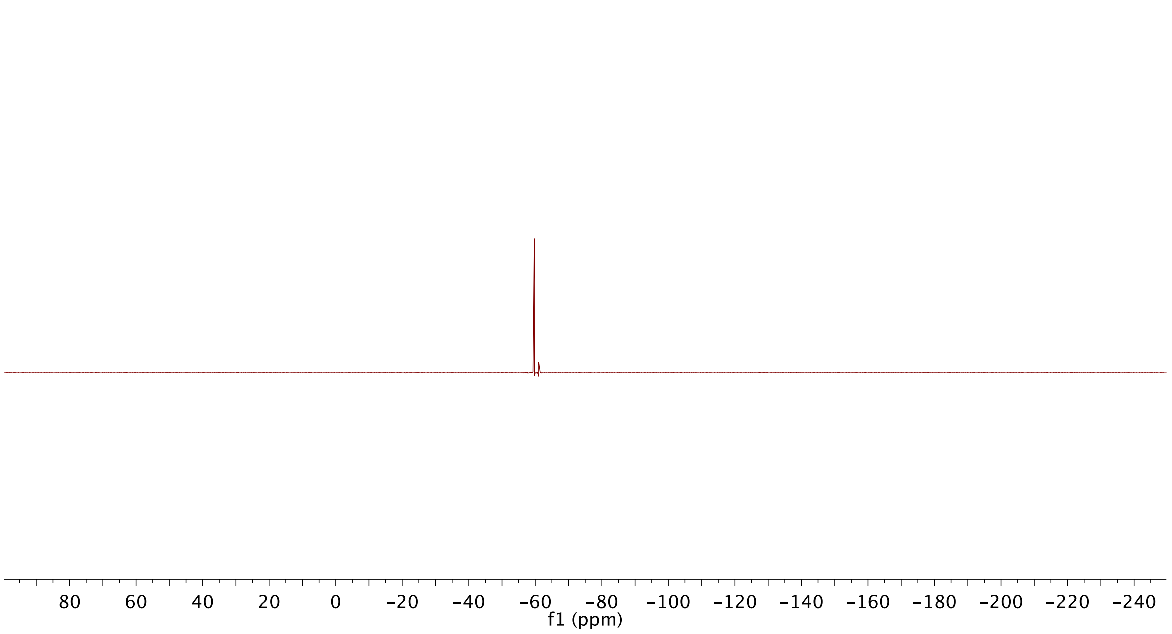

-59.70
80 60 40 20 0 -20 -40 -60 -80 -100 -120 -140 -160 -180 -200 -220 -240
f1 (ppm)

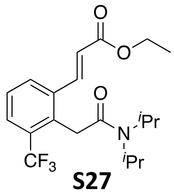

S27

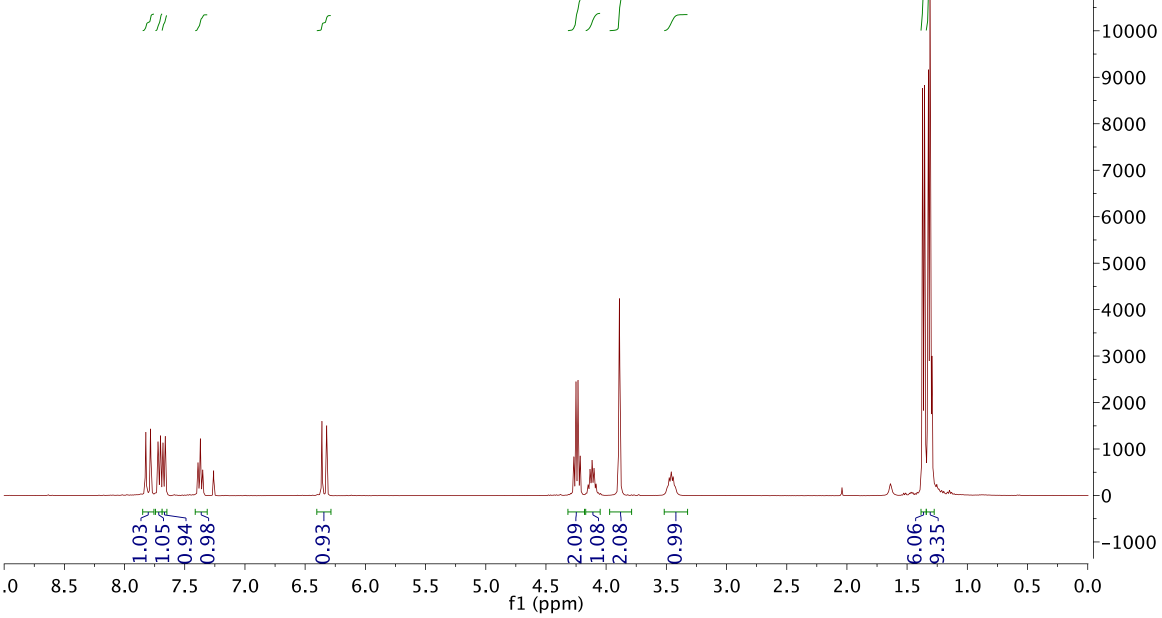

1.03 1.05 0.94 0.98 0.93 2.09 1.08 2.08 0.99 6.06 9.35
14000 13000 12000 11000 10000 9000 8000 7000 6000 5000 4000 3000 2000 1000 0 -1000
.0 8.5 8.0 7.5 7.0 6.5 6.0 5.5 5.0 4.5 4.0 3.5 3.0 2.5 2.0 1.5 1.0 0.5 0.0
f1 (ppm)

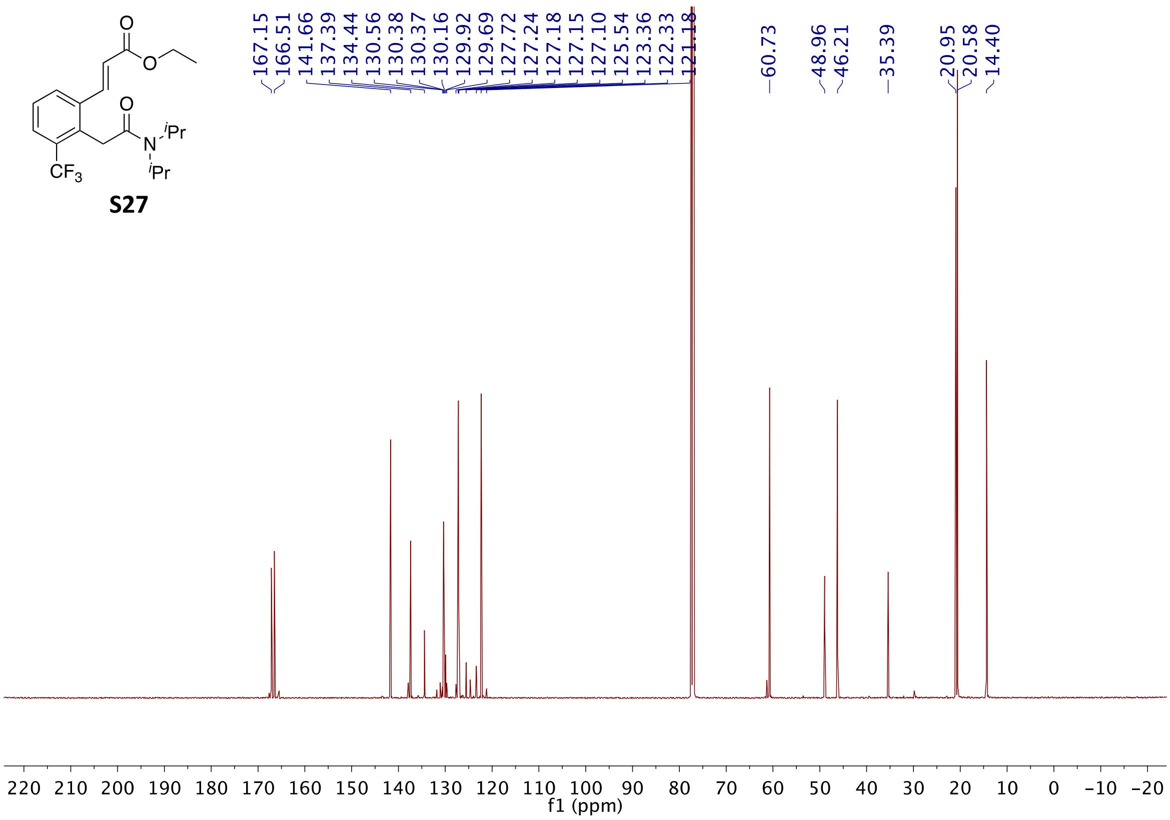
O
O
O
N
iPr
iPr
CF3
S27
167.15
166.51
141.66
137.39
134.44
130.56
130.38
130.37
130.16
129.92
129.69
127.72
127.24
127.18
127.15
127.10
125.54
123.36
122.33
121.18
60.73
48.96
46.21
35.39
20.95
20.58
14.40
220 210 200 190 180 170 160 150 140 130 120 110 100 90 80 70 60 50 40 30 20 10 0 −10 −20
f1 (ppm)

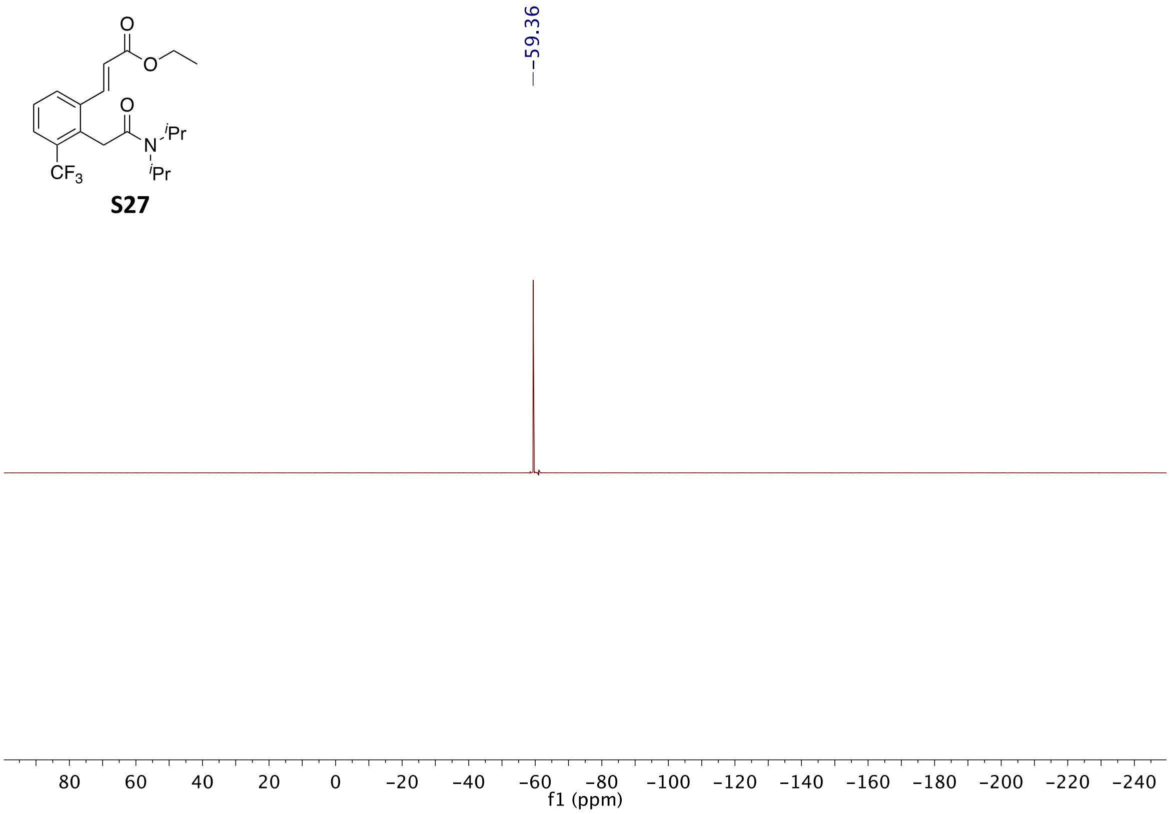
O
O
O
N
iPr
iPr
CF3
S27
−59.36
80 60 40 20 0 −20 −40 −60 −80 −100 −120 −140 −160 −180 −200 −220 −240
f1 (ppm)

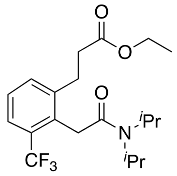
O
O
O
N
iPr
iPr
CF3

S28

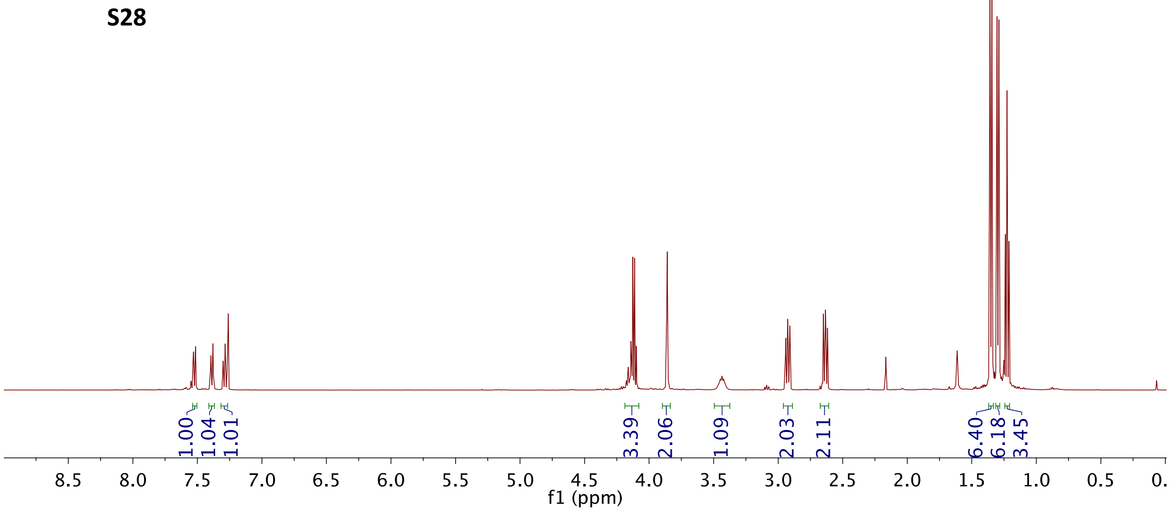
1.00
1.04
1.01
3.39
2.06
1.09
2.03
2.11
6.40
6.18
3.45
8.5 8.0 7.5 7.0 6.5 6.0 5.5 5.0 4.5 4.0 3.5 3.0 2.5 2.0 1.5 1.0 0.5 0.
f1 (ppm)

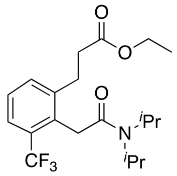
O
O
O
N
iPr
iPr
CF3

S28

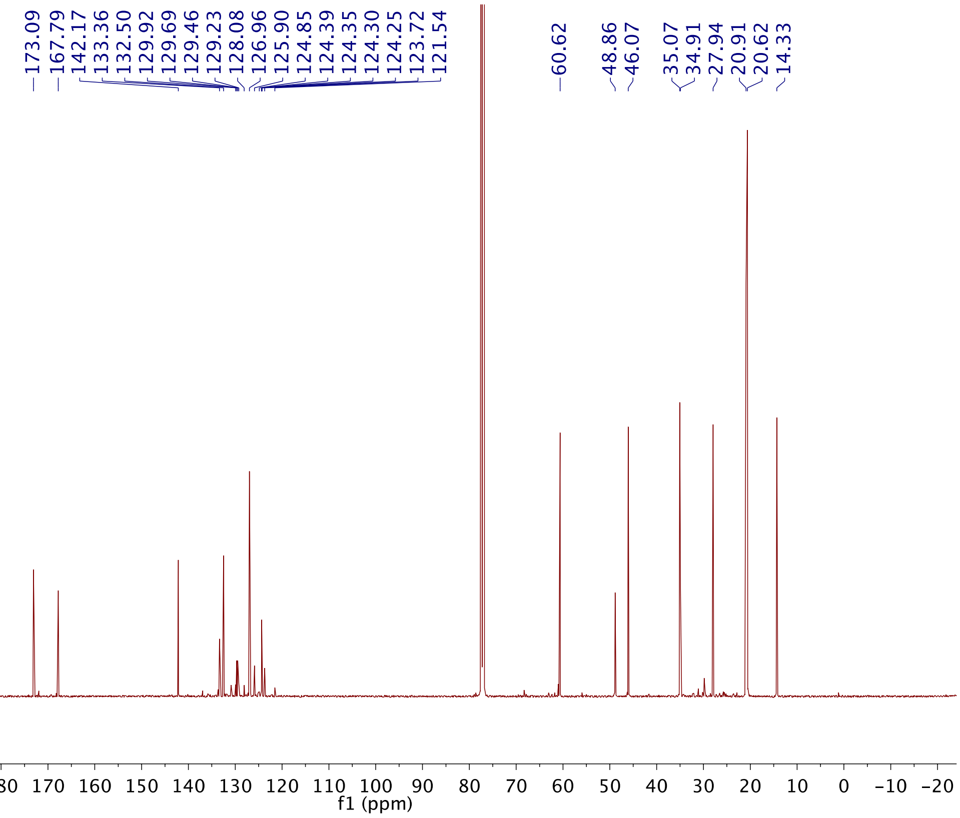
173.09
167.79
142.17
133.36
132.50
129.92
129.69
129.46
129.23
128.08
126.96
125.90
124.85
124.39
124.35
124.30
124.25
123.72
121.54
60.62
48.86
46.07
35.07
34.91
27.94
20.91
20.62
14.33
220 210 200 190 180 170 160 150 140 130 120 110 100 90 80 70 60 50 40 30 20 10 0 −10 −20
f1 (ppm)

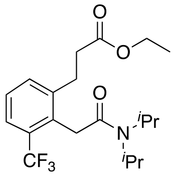
O
O
O
N
iPr
iPr
CF3

S28

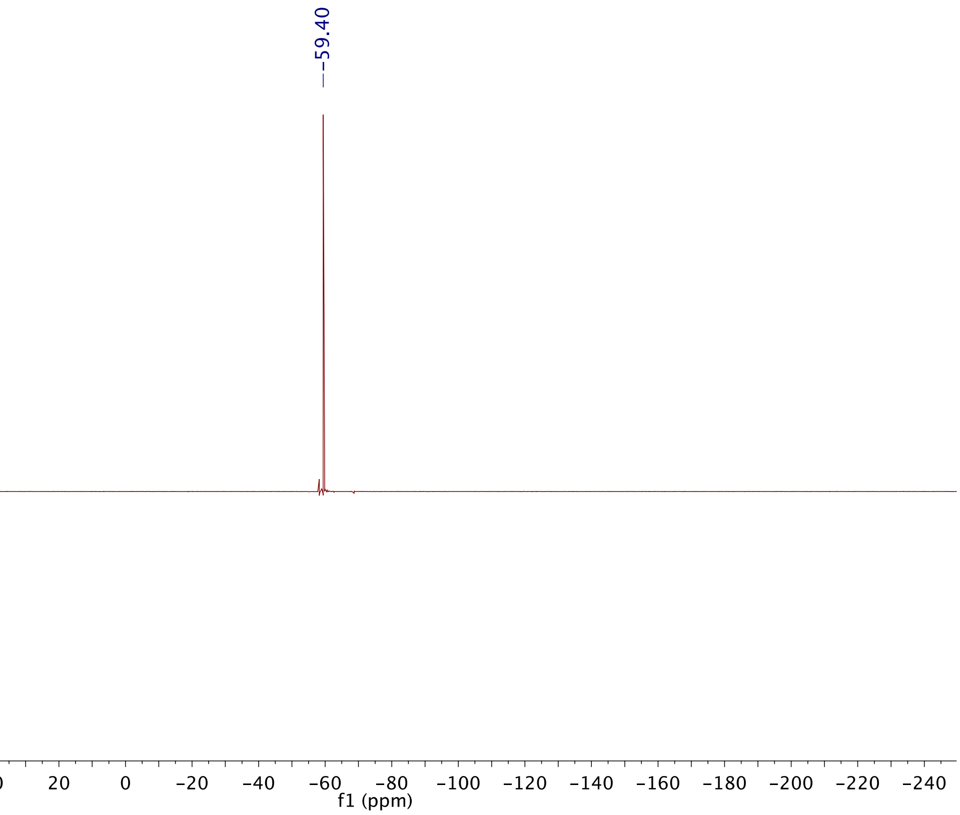
−59.40
80 60 40 20 0 −20 −40 −60 −80 −100 −120 −140 −160 −180 −200 −220 −240
f1 (ppm)

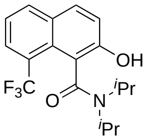
OH
F3C
O
N
iPr
iPr

S30

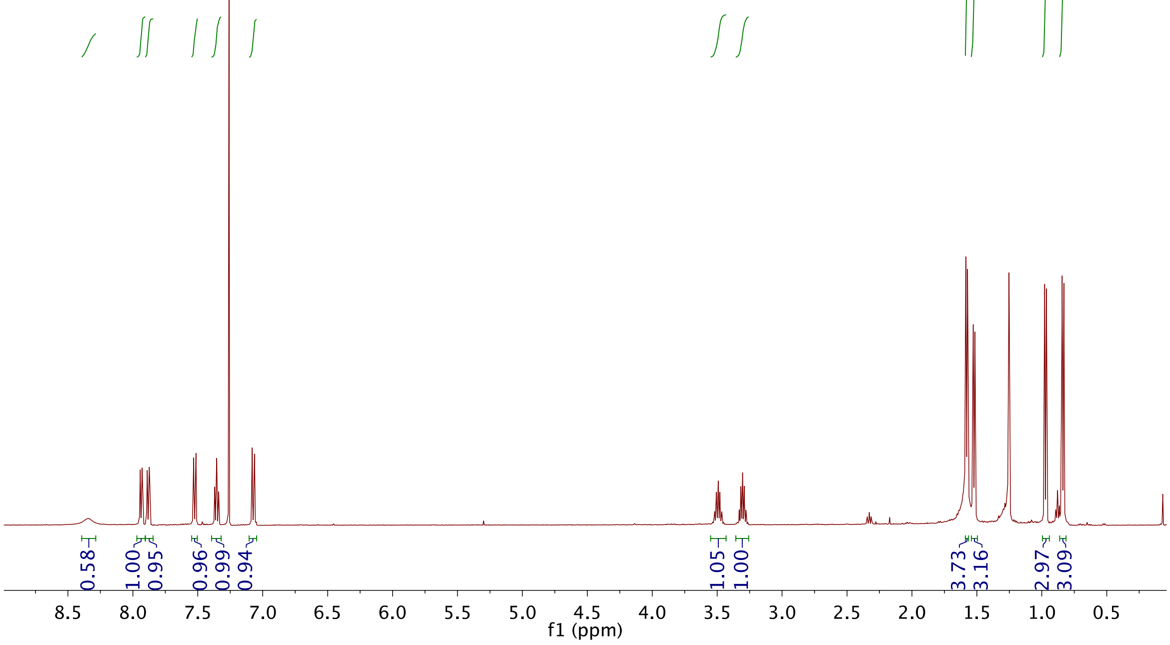
0.58
1.00
0.95
0.96
0.99
0.94
1.05
1.00
3.73
3.16
2.97
3.09
8.5 8.0 7.5 7.0 6.5 6.0 5.5 5.0 4.5 4.0 3.5 3.0 2.5 2.0 1.5 1.0 0.5
f1 (ppm)

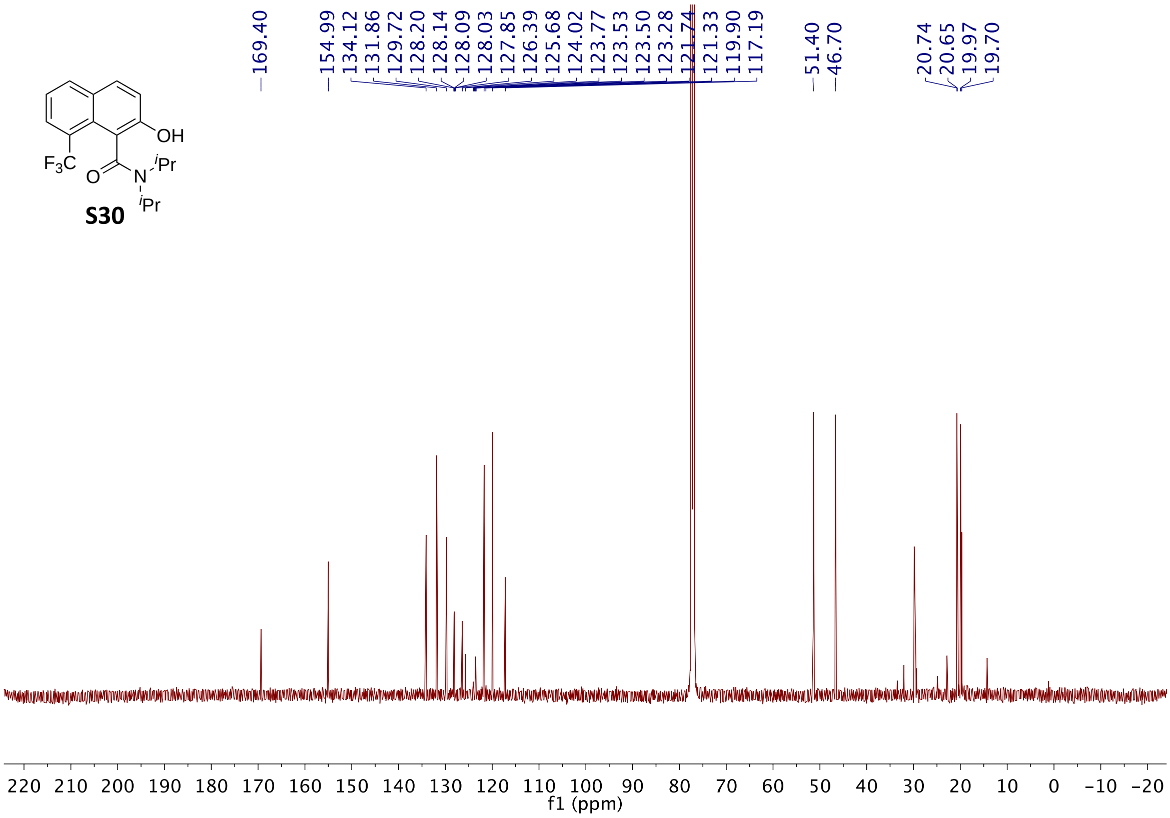
OH
F3C
O
N
iPr
iPr
S30
169.40
154.99
134.12
131.86
129.72
128.20
128.14
128.09
128.03
127.85
126.39
125.68
124.02
123.77
123.53
123.50
123.28
121.74
121.33
119.90
117.19
51.40
46.70
20.74
20.65
19.97
19.70
220 210 200 190 180 170 160 150 140 130 120 110 100 90 80 70 60 50 40 30 20 10 0 −10 −20
f1 (ppm)

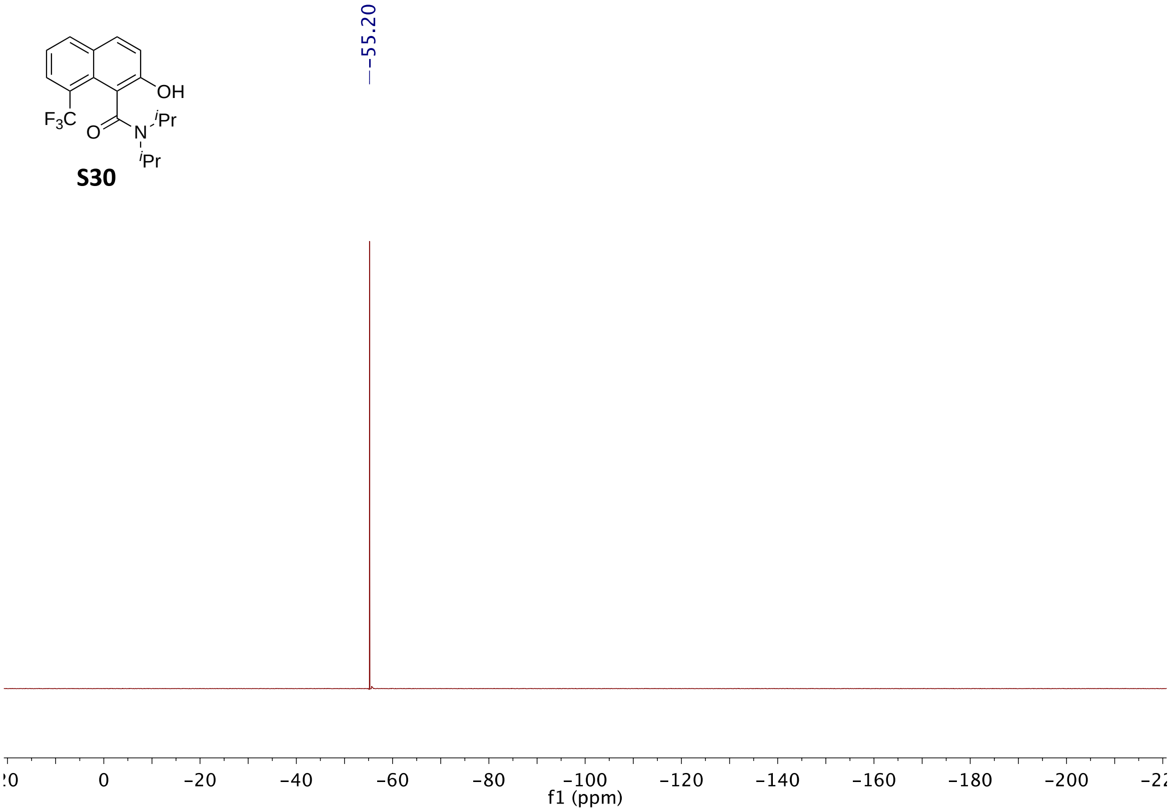
OH
F3C
O
N
iPr
iPr
S30
−55.20
0 −20 −40 −60 −80 −100 −120 −140 −160 −180 −200
f1 (ppm)

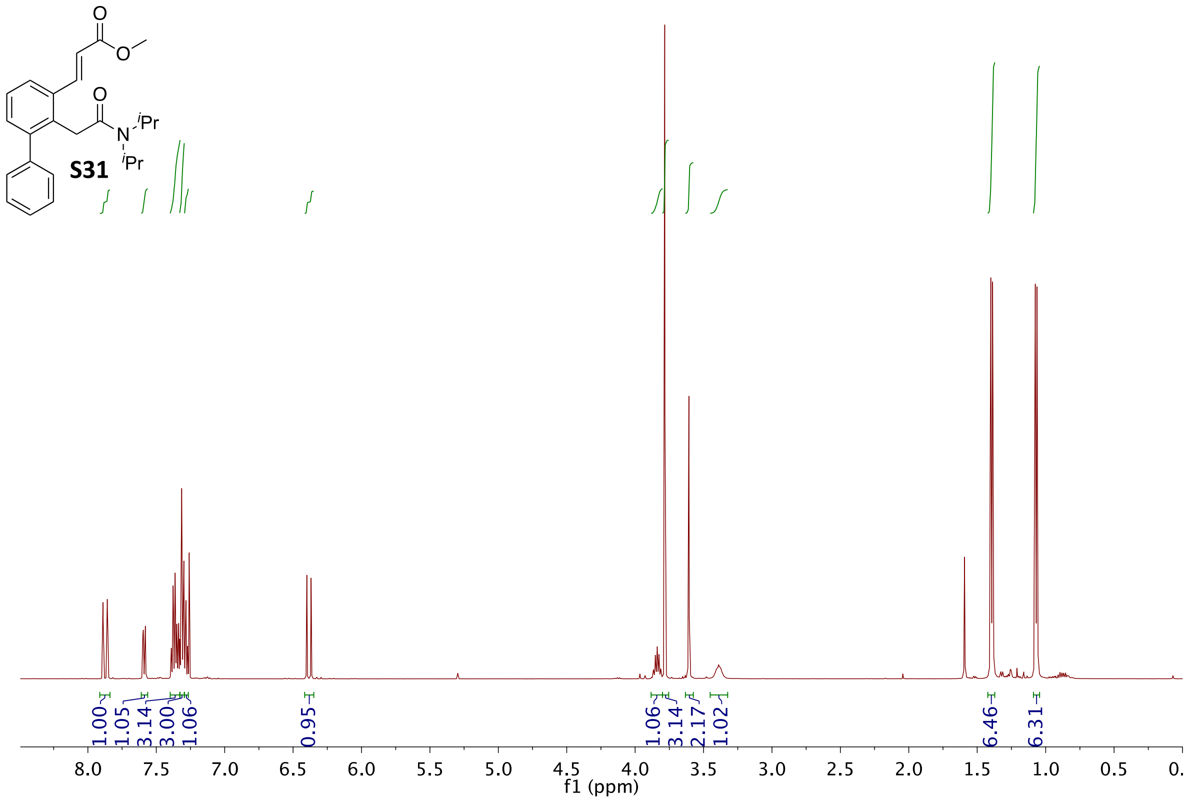
S31
1.00
1.05
3.14
3.00
1.06
0.95
1.06
3.14
2.17
1.02
6.46
6.31
f1 (ppm)

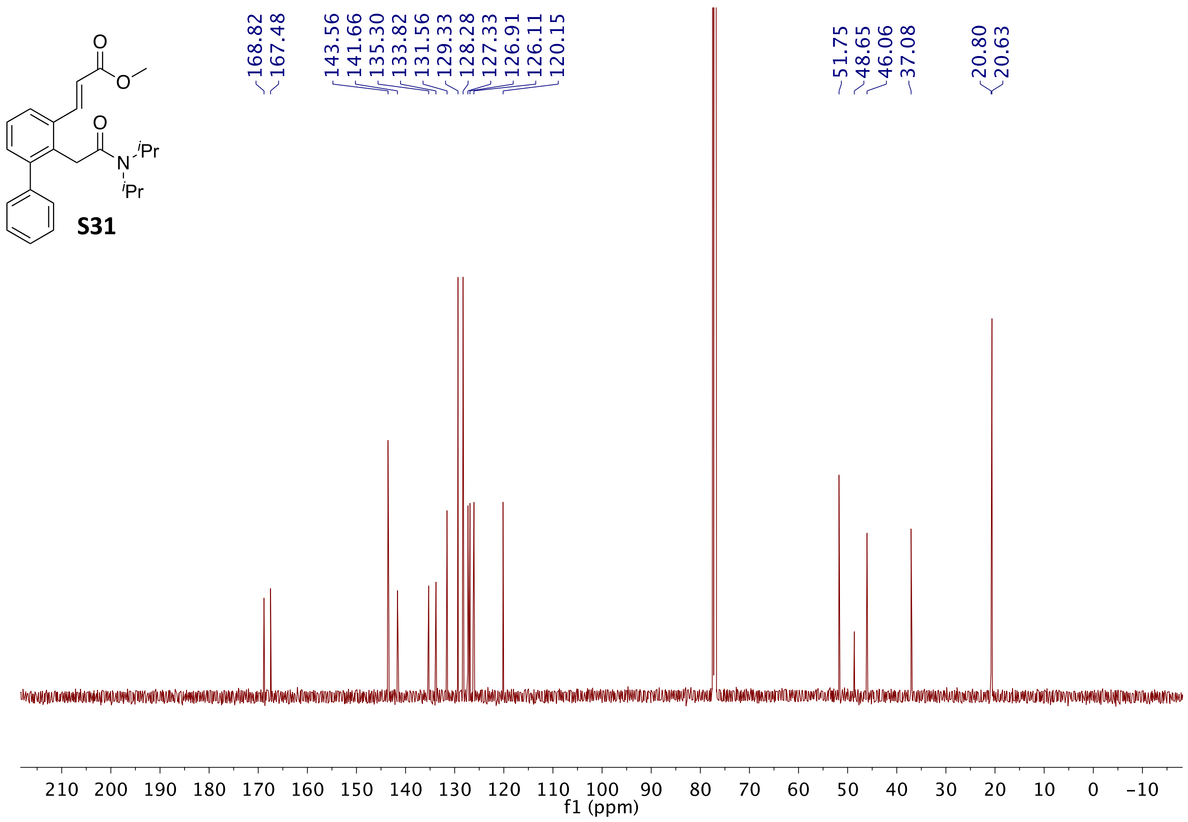
168.82
167.48
143.56
141.66
135.30
133.82
131.56
129.33
128.28
127.33
126.91
126.11
120.15
51.75
48.65
46.06
37.08
20.80
20.63
S31
f1 (ppm)

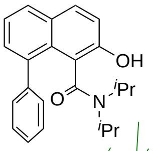

S32

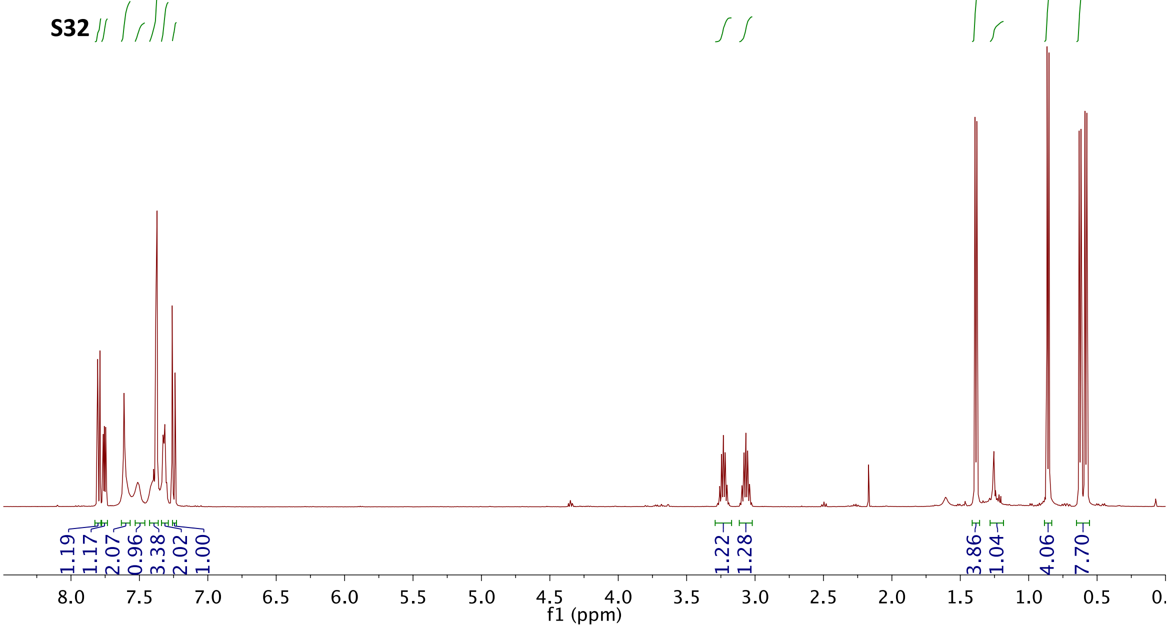

1.19
1.17
2.07
0.96
3.38
2.02
1.00
1.22
1.28
3.86
1.04
4.06
7.70
8.0 7.5 7.0 6.5 6.0 5.5 5.0 4.5 4.0 3.5 3.0 2.5 2.0 1.5 1.0 0.5
f1 (ppm)

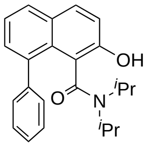

S32

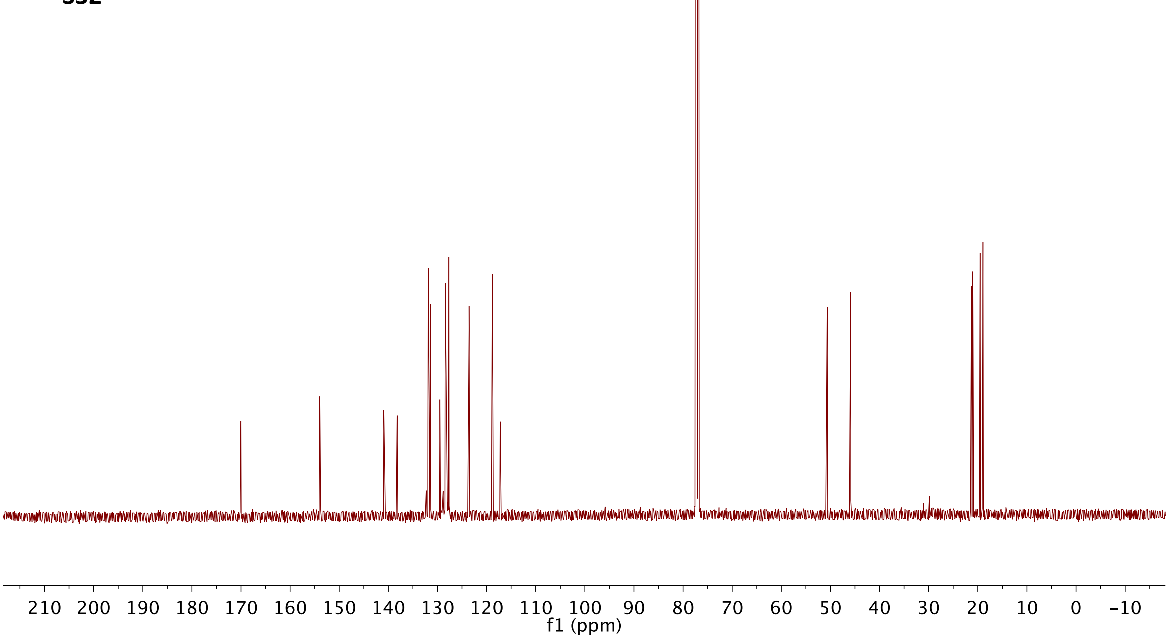

170.03
153.95
140.92
138.21
132.30
131.89
131.48
129.51
128.87
128.42
128.36
128.29
127.86
127.67
123.56
118.83
117.24
50.66
45.88
21.30
21.01
19.54
18.95
210 200 190 180 170 160 150 140 130 120 110 100 90 80 70 60 50 40 30 20 10 0 −10
f1 (ppm)

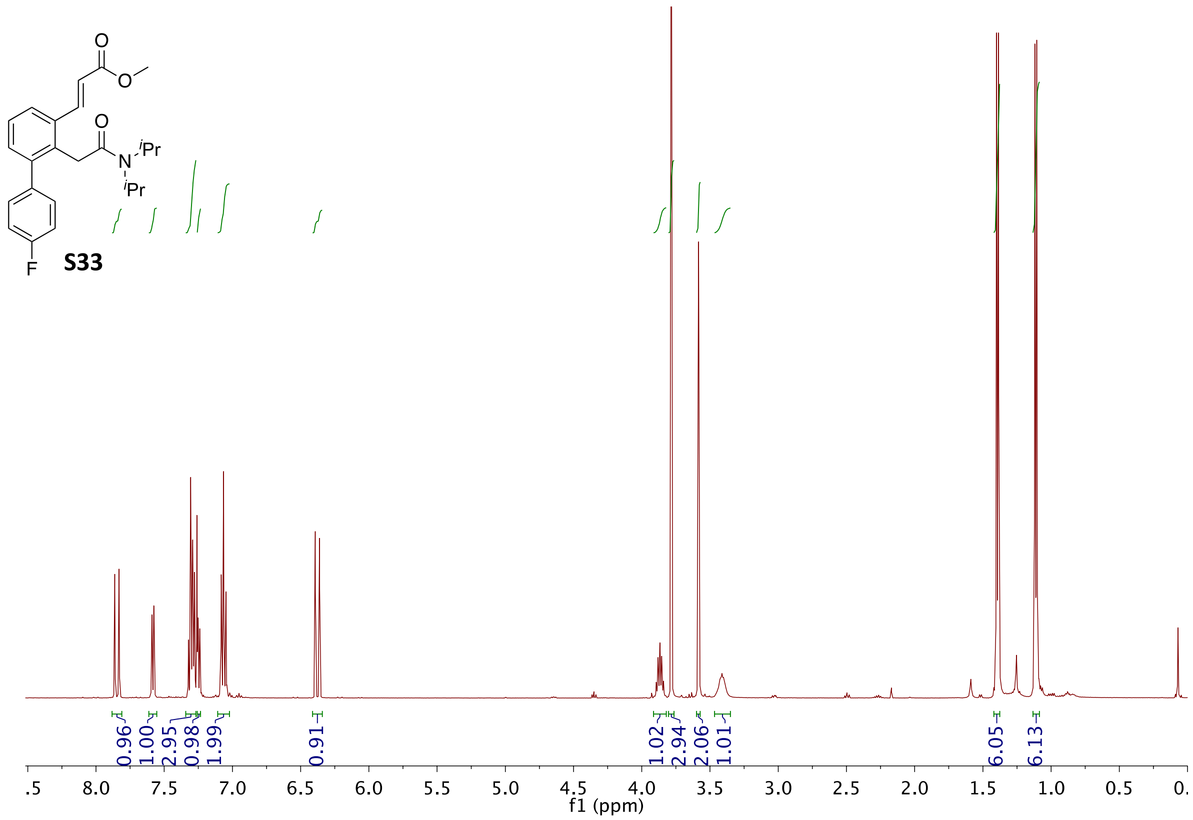
O
O
O
N
iPr
iPr
F
S33
0.96
1.00
2.95
0.98
1.99
0.91
1.02
2.94
2.06
1.01
6.05
6.13
f1 (ppm)

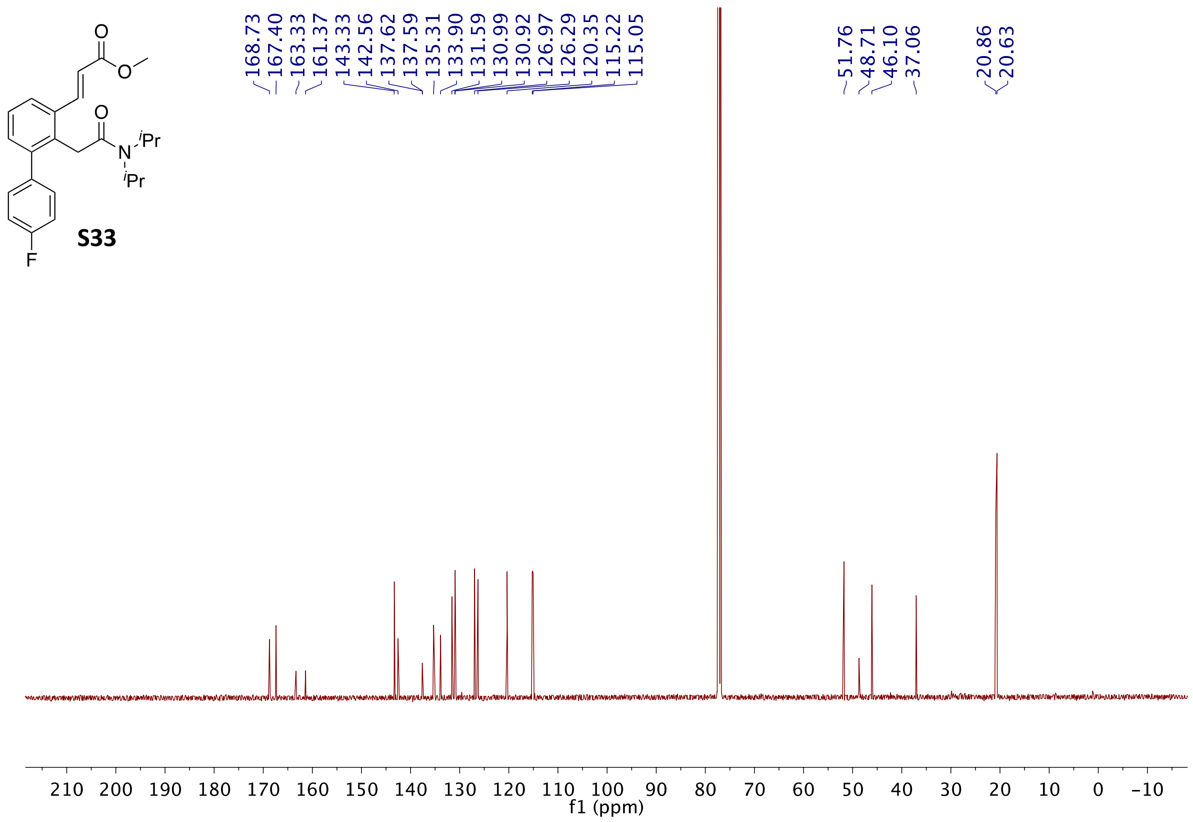
168.73
167.40
163.33
161.37
143.33
142.56
137.62
137.59
135.31
133.90
131.59
130.99
130.92
126.97
126.29
120.35
115.22
115.05
51.76
48.71
46.10
37.06
20.86
20.63
O
O
O
N
iPr
iPr
F
S33
f1 (ppm)

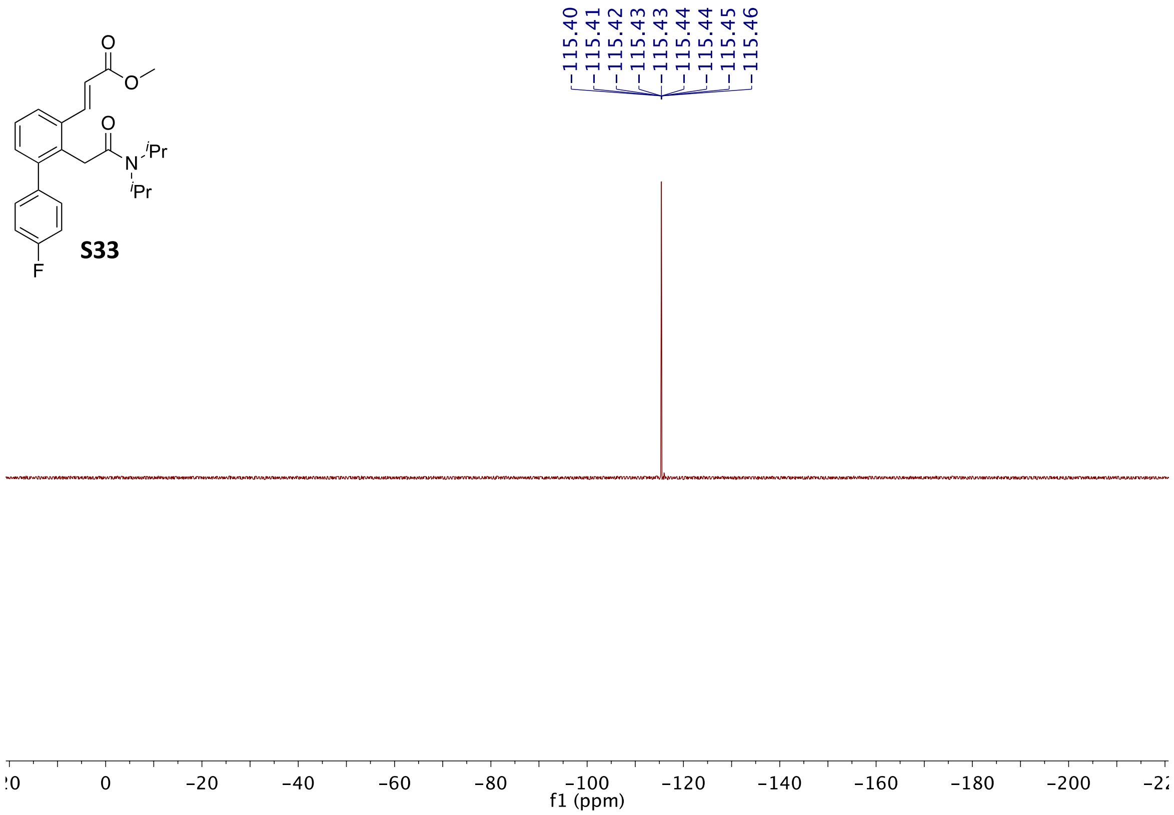

O
O
O
N
iPr
iPr
F
-115.40
-115.41
-115.42
-115.43
-115.43
-115.44
-115.44
-115.45
-115.46
f1 (ppm)

S33

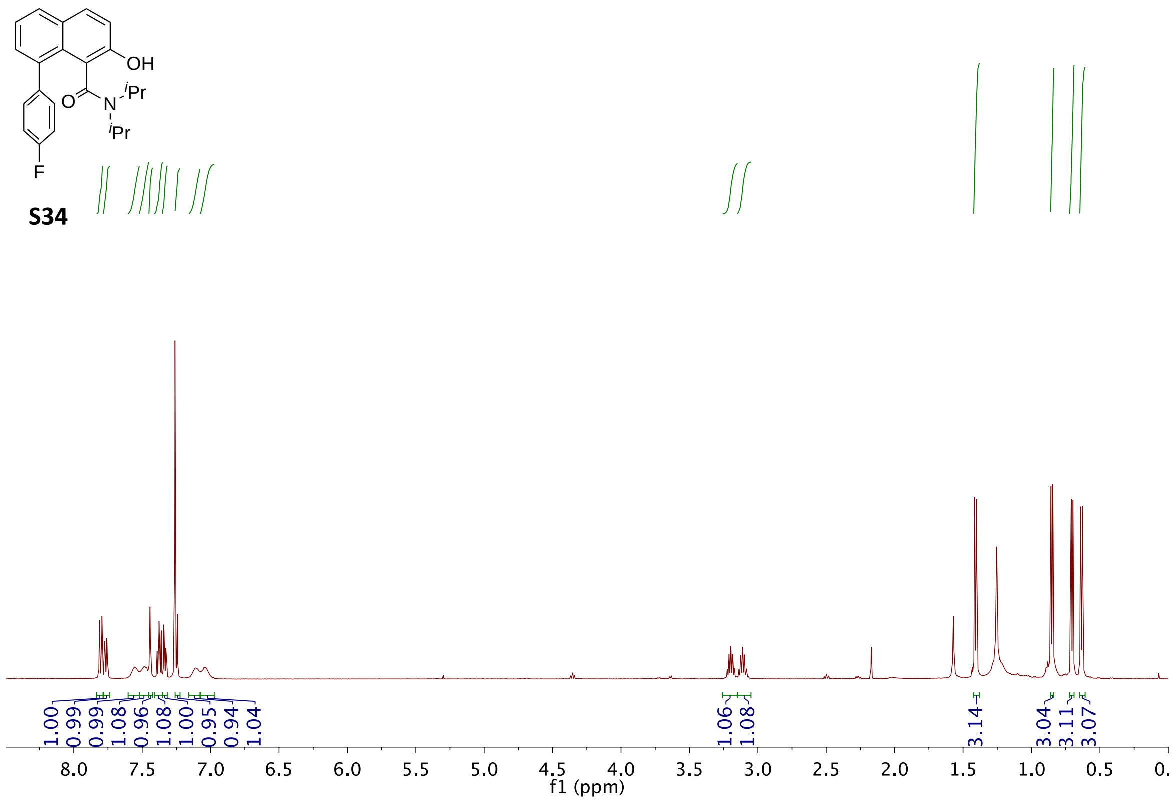

OH
O
N
iPr
iPr
F
1.00
0.99
1.08
0.96
1.08
1.00
0.95
0.94
1.04
1.06
1.08
3.14
3.04
3.11
3.07
f1 (ppm)

S34

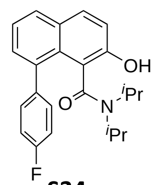
OH
iPr
O
N
iPr
F

S34

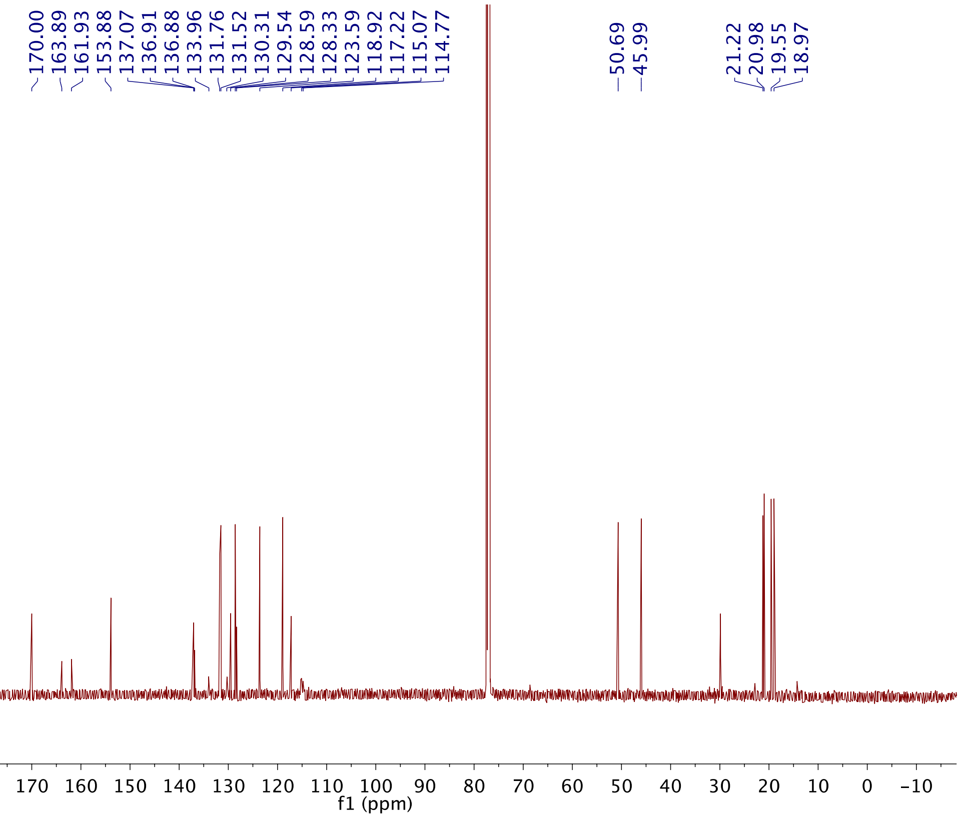
170.00
163.89
161.93
153.88
137.07
136.91
136.88
133.96
131.76
131.52
130.31
129.54
128.59
128.33
123.59
118.92
117.22
115.07
114.77
50.69
45.99
21.22
20.98
19.55
18.97
210 200 190 180 170 160 150 140 130 120 110 100 90 80 70 60 50 40 30 20 10 0 −10
f1 (ppm)

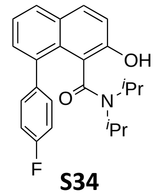
OH
iPr
O
N
iPr
F

S34

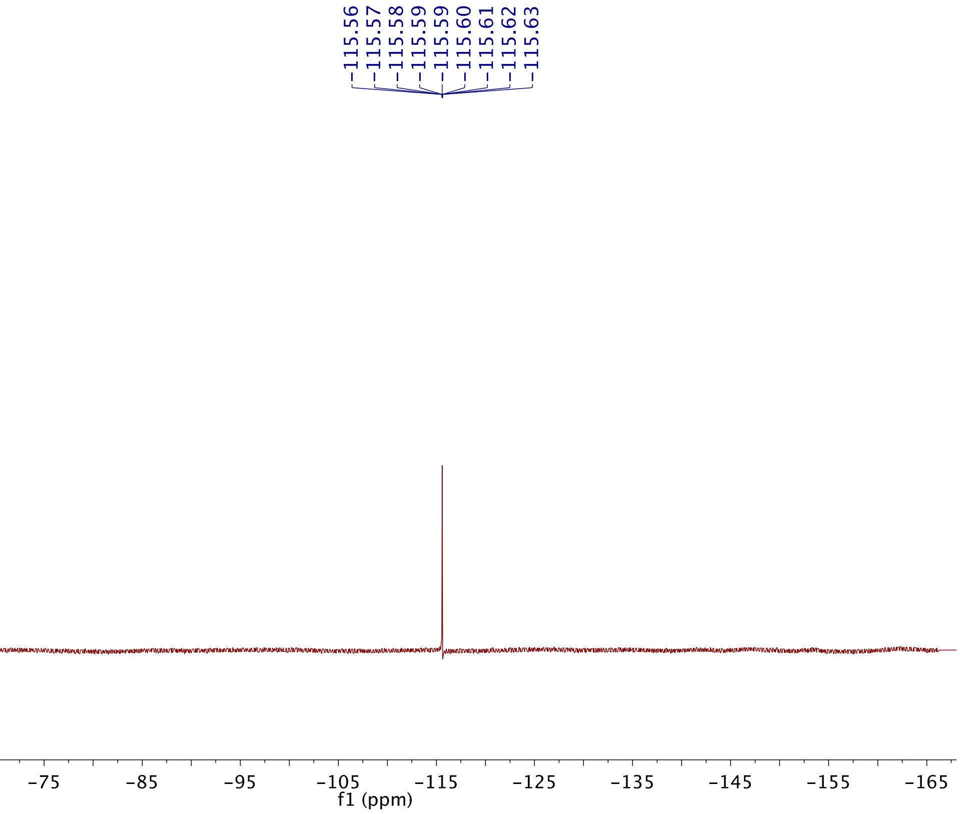
−115.56
−115.57
−115.58
−115.59
−115.59
−115.60
−115.61
−115.62
−115.63
−55 −65 −75 −85 −95 −105 −115 −125 −135 −145 −155 −165
f1 (ppm)

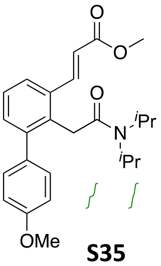
O
O
O
N
iPr
iPr
OMe

S35

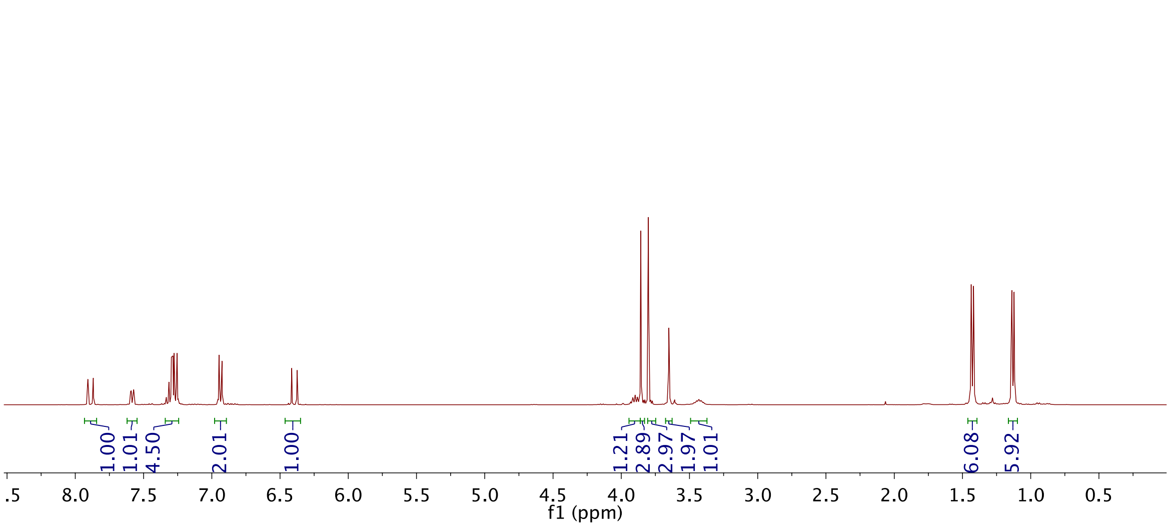
1.00
1.01
4.50
2.01
1.00
1.21
2.89
2.97
1.97
1.01
6.08
5.92
.5 8.0 7.5 7.0 6.5 6.0 5.5 5.0 4.5 4.0 3.5 3.0 2.5 2.0 1.5 1.0 0.5
f1 (ppm)

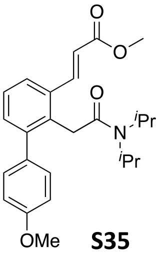
O
O
O
N
iPr
iPr
OMe

S35

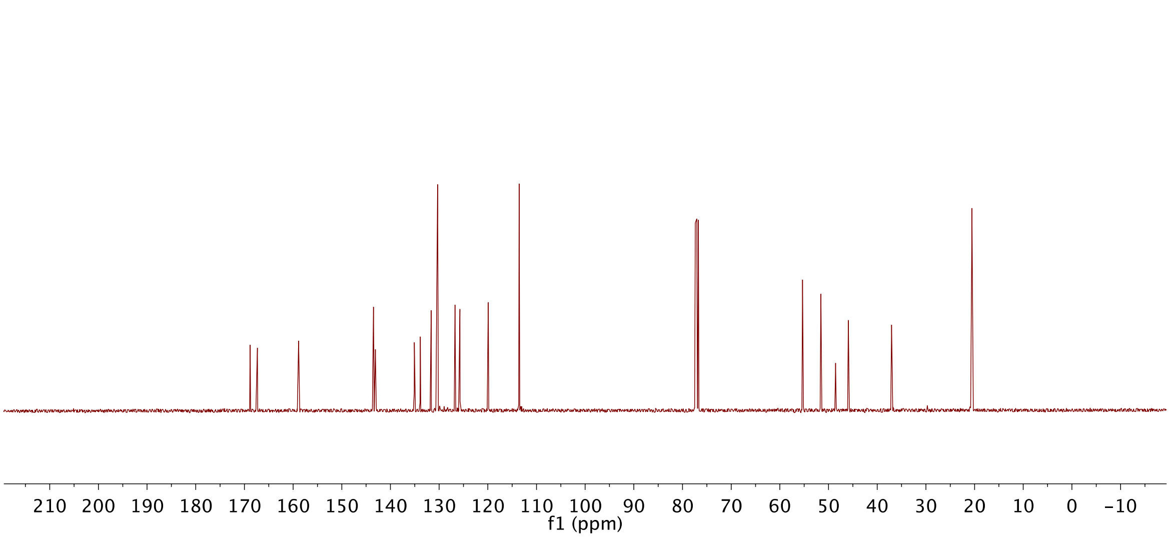
168.83
167.32
158.88
143.49
143.09
135.10
133.91
133.87
131.64
130.30
126.74
125.73
119.92
113.56
55.34
51.58
48.54
45.93
37.04
20.72
20.52
210 200 190 180 170 160 150 140 130 120 110 100 90 80 70 60 50 40 30 20 10 0 −10
f1 (ppm)

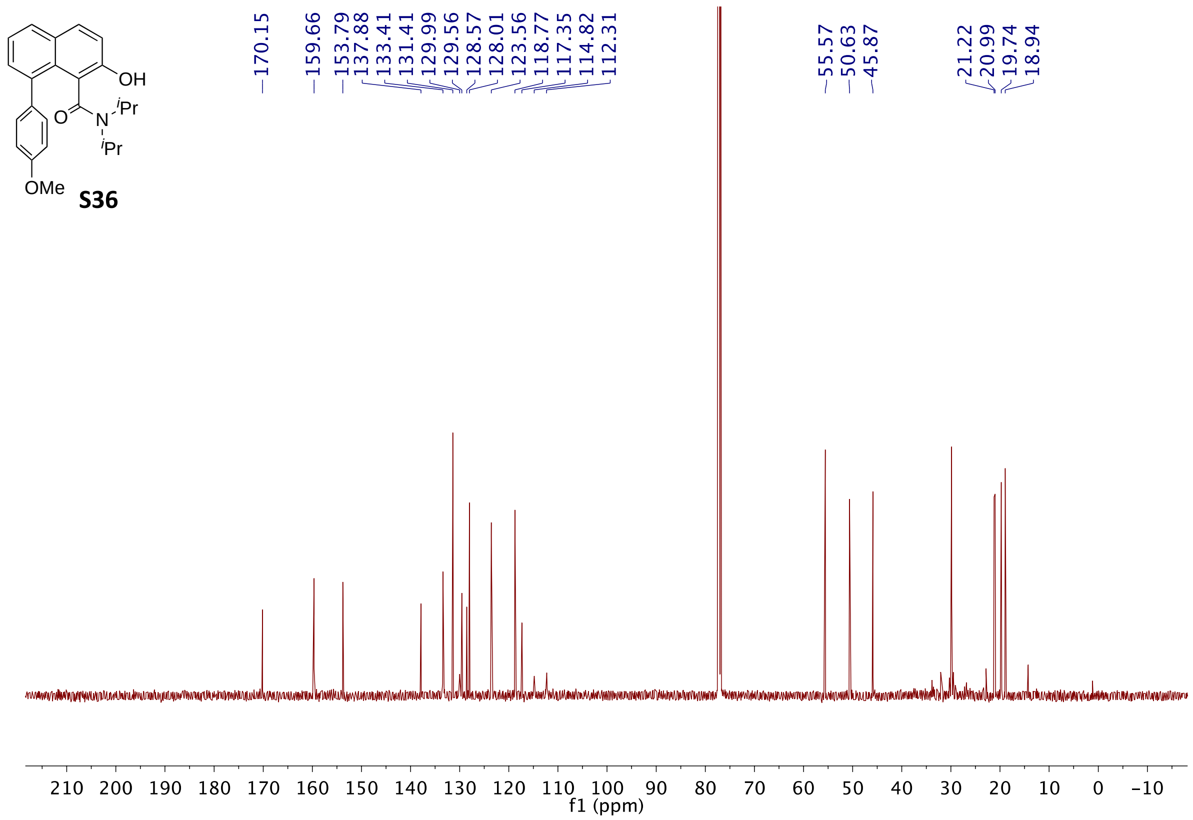
OH
O
N
iPr
iPr
OMe
S36
170.15
159.66
153.79
137.88
133.41
131.41
129.99
129.56
128.57
128.01
123.56
118.77
117.35
114.82
112.31
55.57
50.63
45.87
21.22
20.99
19.74
18.94
210 200 190 180 170 160 150 140 130 120 110 100 90 80 70 60 50 40 30 20 10 0 -10
f1 (ppm)

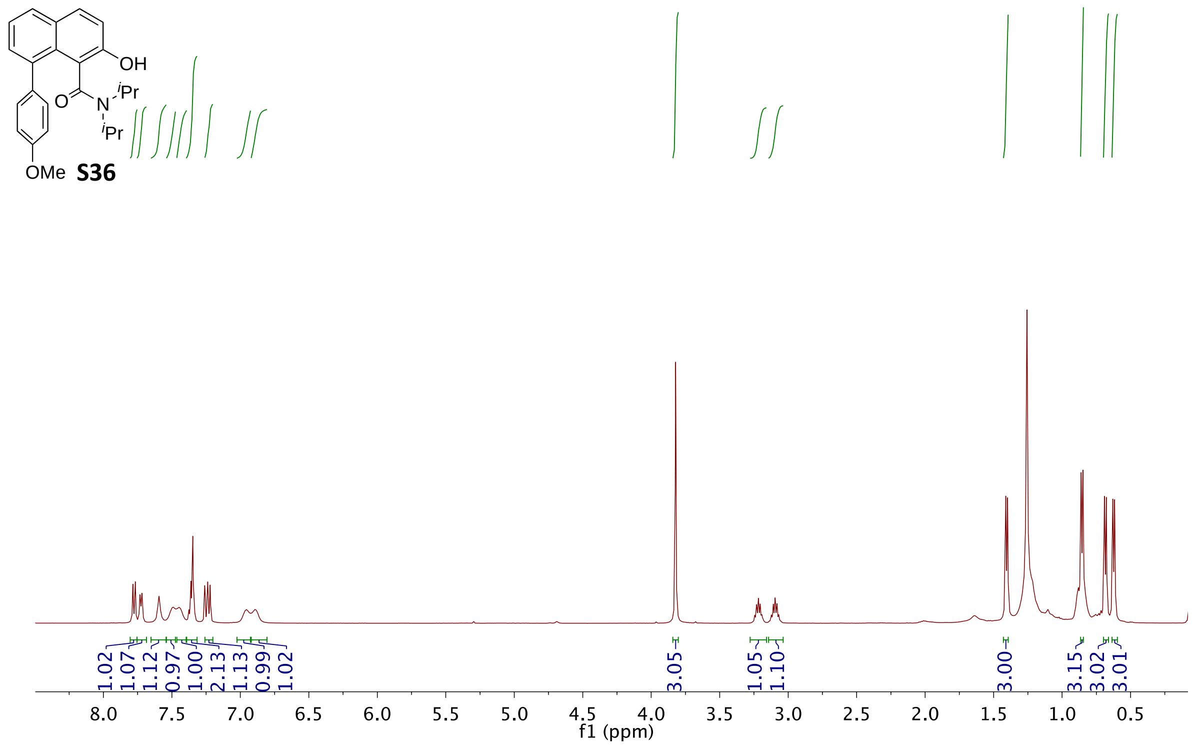
OH
O
N
iPr
iPr
OMe
S36
1.02
1.07
1.12
0.97
1.00
2.13
1.13
0.99
1.02
3.05
1.05
1.10
3.00
3.15
3.02
3.01
8.0 7.5 7.0 6.5 6.0 5.5 5.0 4.5 4.0 3.5 3.0 2.5 2.0 1.5 1.0 0.5
f1 (ppm)

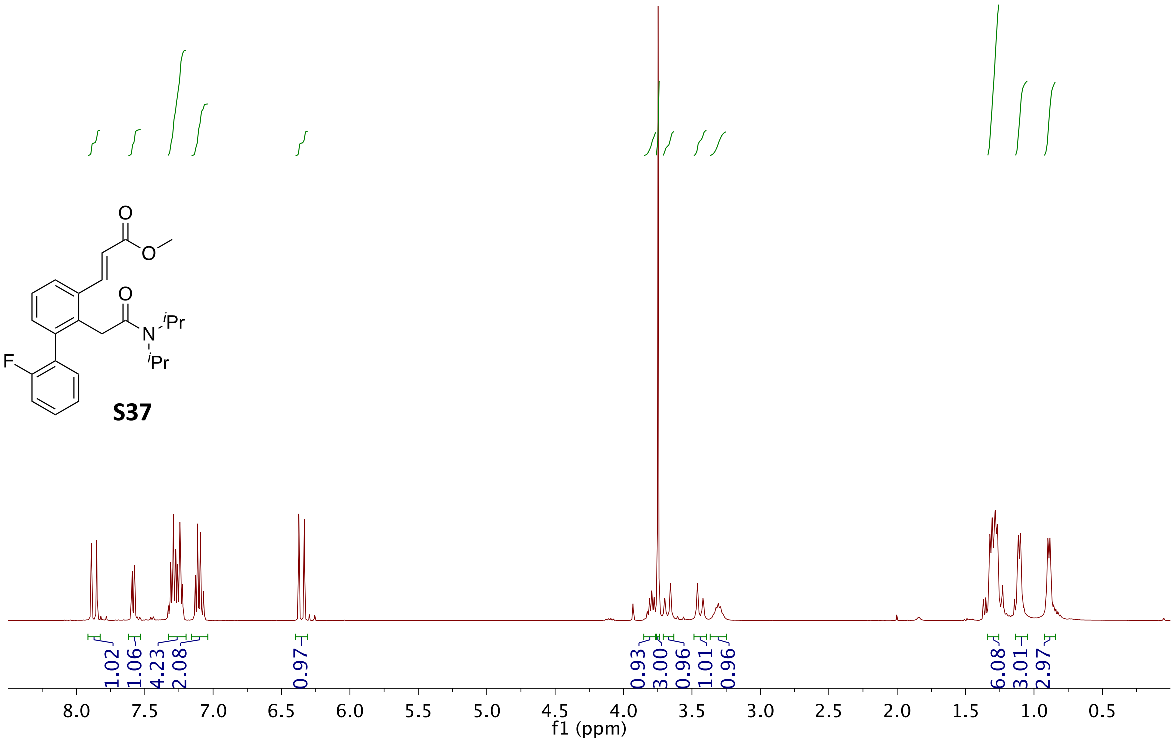
O
O
O
N
iPr
iPr
F
S37
1.02
1.06
4.23
2.08
0.97
0.93
3.00
0.96
1.01
0.96
6.08
3.01
2.97
8.0 7.5 7.0 6.5 6.0 5.5 5.0 4.5 4.0 3.5 3.0 2.5 2.0 1.5 1.0 0.5
f1 (ppm)

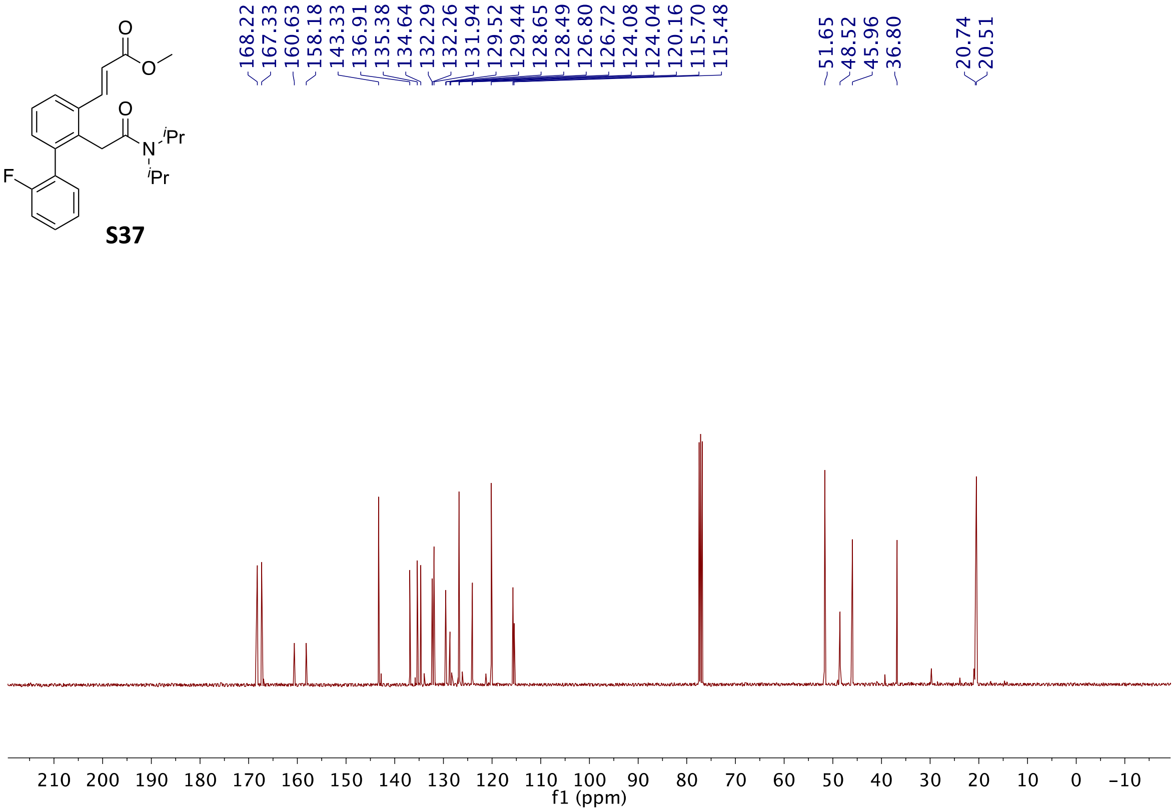
168.22
167.33
160.63
158.18
143.33
136.91
135.38
134.64
132.29
132.26
131.94
129.52
129.44
128.65
128.49
126.80
126.72
124.08
124.04
120.16
115.70
115.48
51.65
48.52
45.96
36.80
20.74
20.51
O
O
O
N
iPr
iPr
F
S37
210 200 190 180 170 160 150 140 130 120 110 100 90 80 70 60 50 40 30 20 10 0 −10
f1 (ppm)

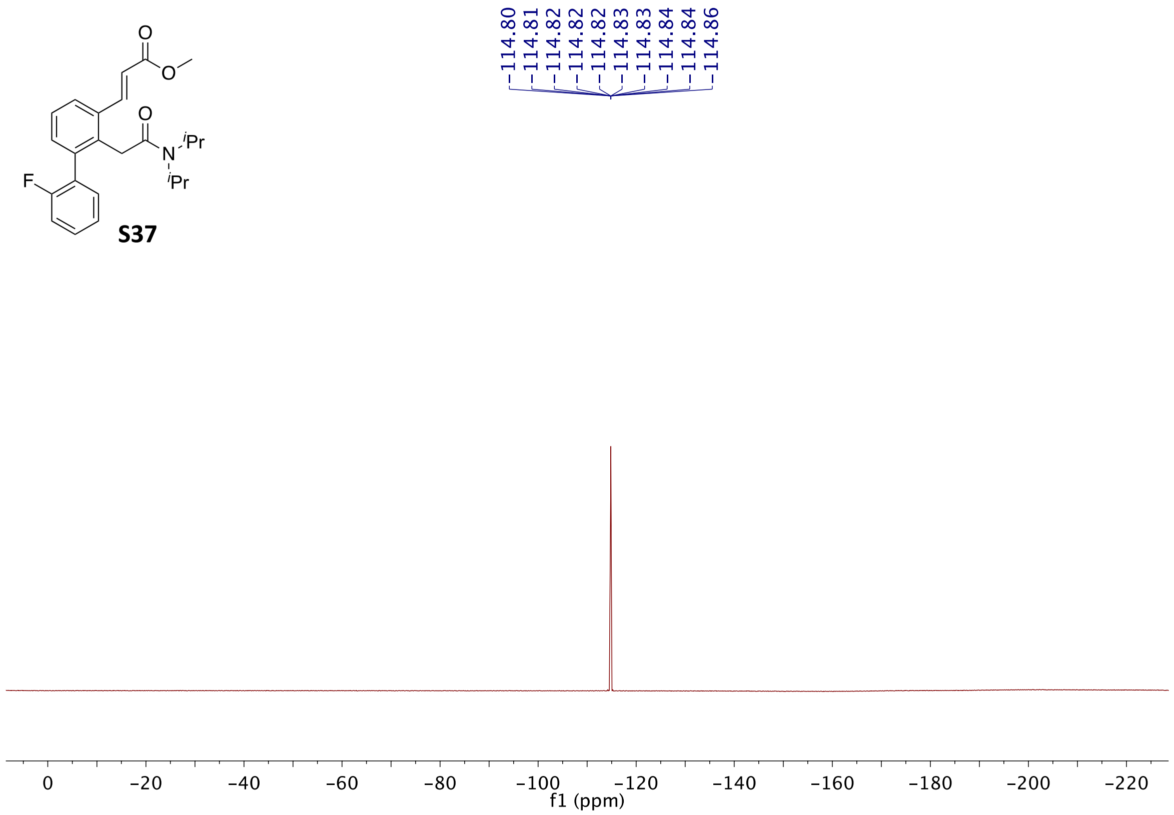
-114.80
-114.81
-114.82
-114.82
-114.82
-114.83
-114.83
-114.84
-114.84
-114.86
F
O
O
O
N
iPr
iPr
S37
f1 (ppm)

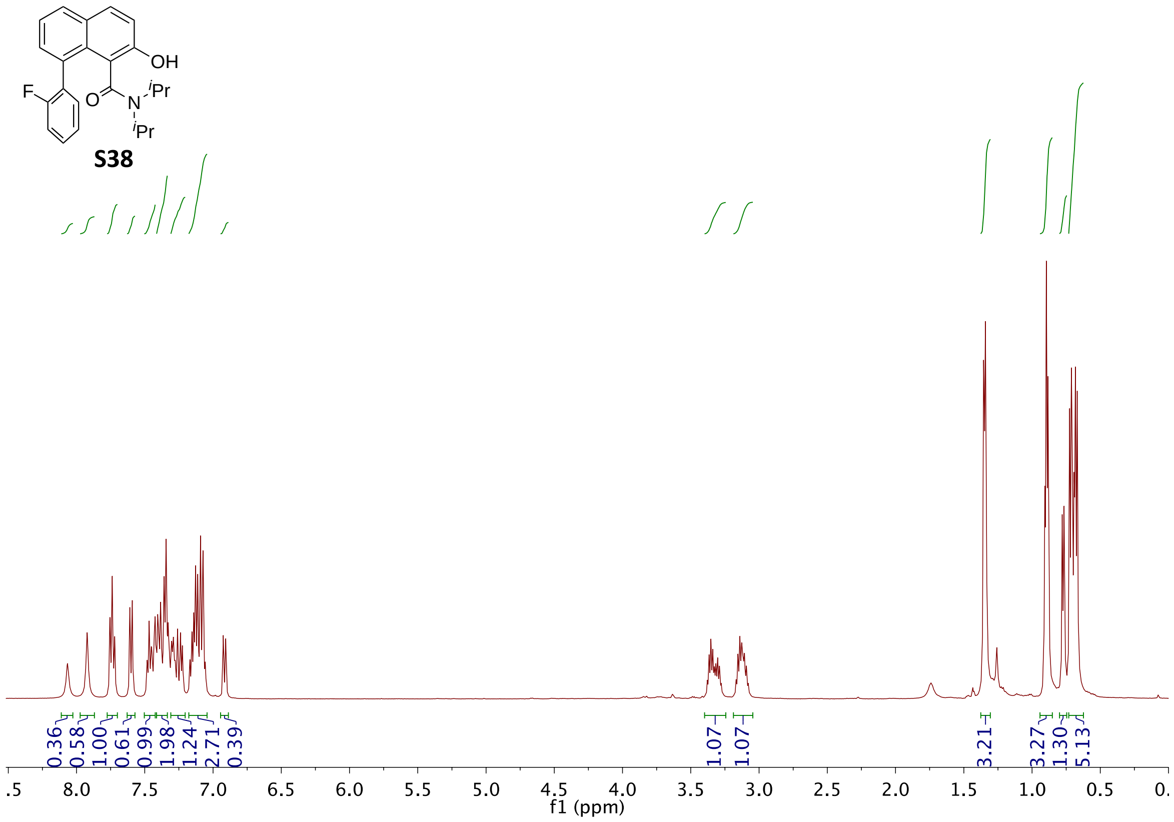
OH
F
O
N
iPr
iPr
S38
0.36
0.58
1.00
0.61
0.99
1.98
1.24
2.71
0.39
1.07
1.07
3.21
3.27
1.30
5.13
f1 (ppm)

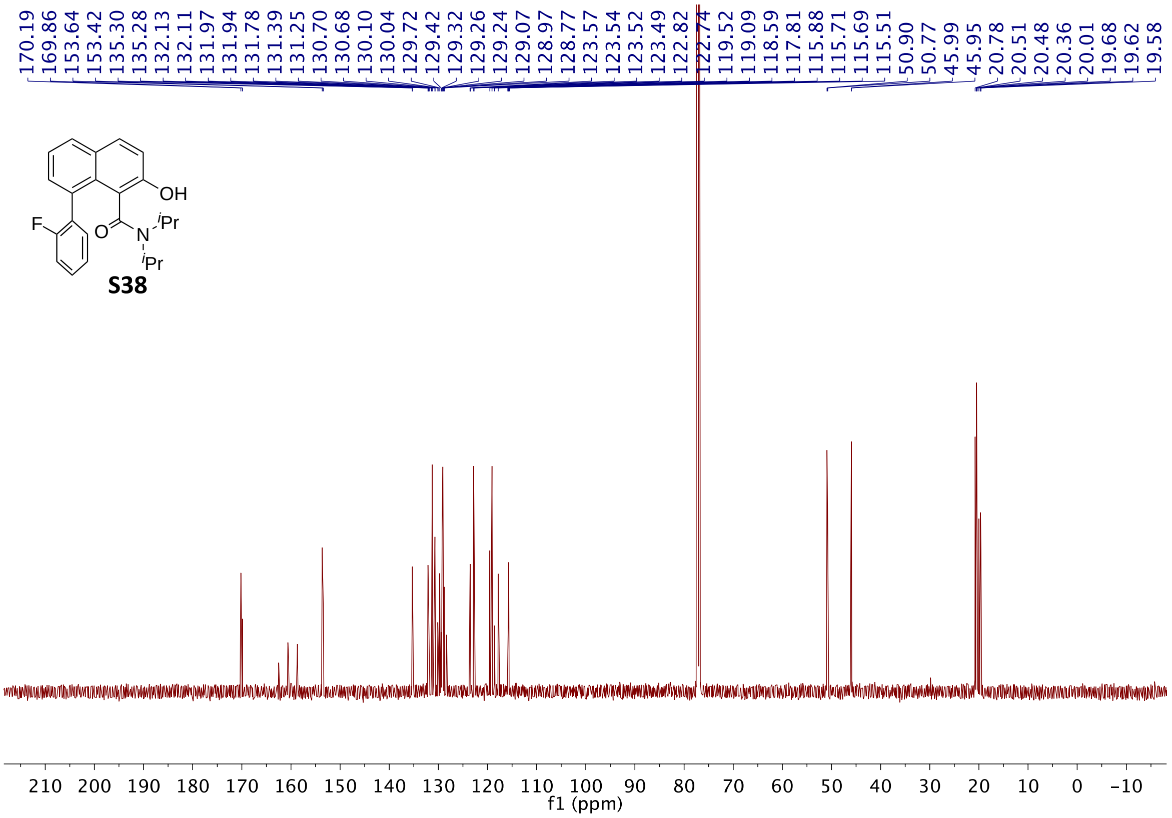
170.19
169.86
153.64
153.42
135.30
135.28
132.13
132.11
131.97
131.94
131.78
131.39
131.25
130.70
130.68
130.10
130.04
129.72
129.42
129.32
129.26
129.24
129.07
128.97
128.77
123.57
123.54
123.52
123.49
122.82
122.74
119.52
119.09
118.59
117.81
115.88
115.71
115.69
115.51
50.90
50.77
45.99
45.95
20.78
20.51
20.48
20.36
20.01
19.68
19.62
19.58
OH
F
O
N
iPr
iPr
S38
210 200 190 180 170 160 150 140 130 120 110 100 90 80 70 60 50 40 30 20 10 0 −10
f1 (ppm)

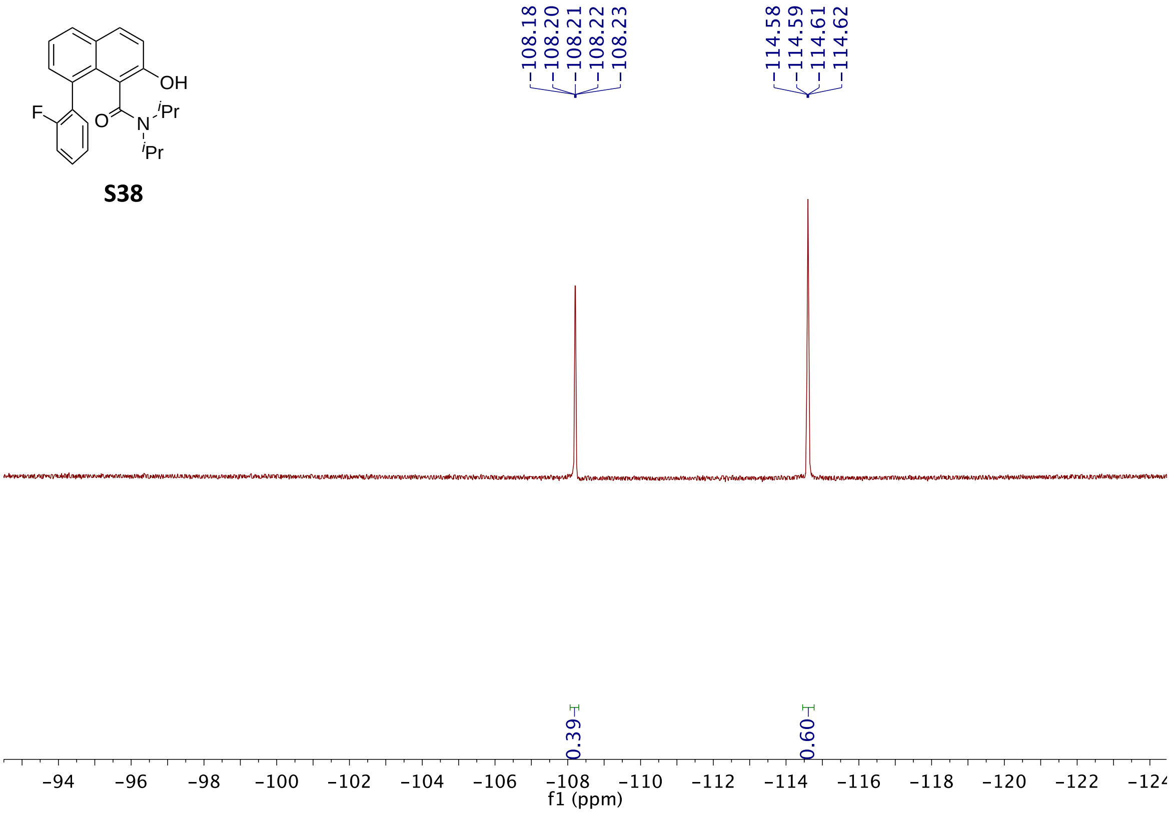
−108.18
−108.20
−108.21
−108.22
−108.23
−114.58
−114.59
−114.61
−114.62
OH
F
O
N
iPr
iPr
S38
0.39
0.60
−94 −96 −98 −100 −102 −104 −106 −108 −110 −112 −114 −116 −118 −120 −122 −124
f1 (ppm)

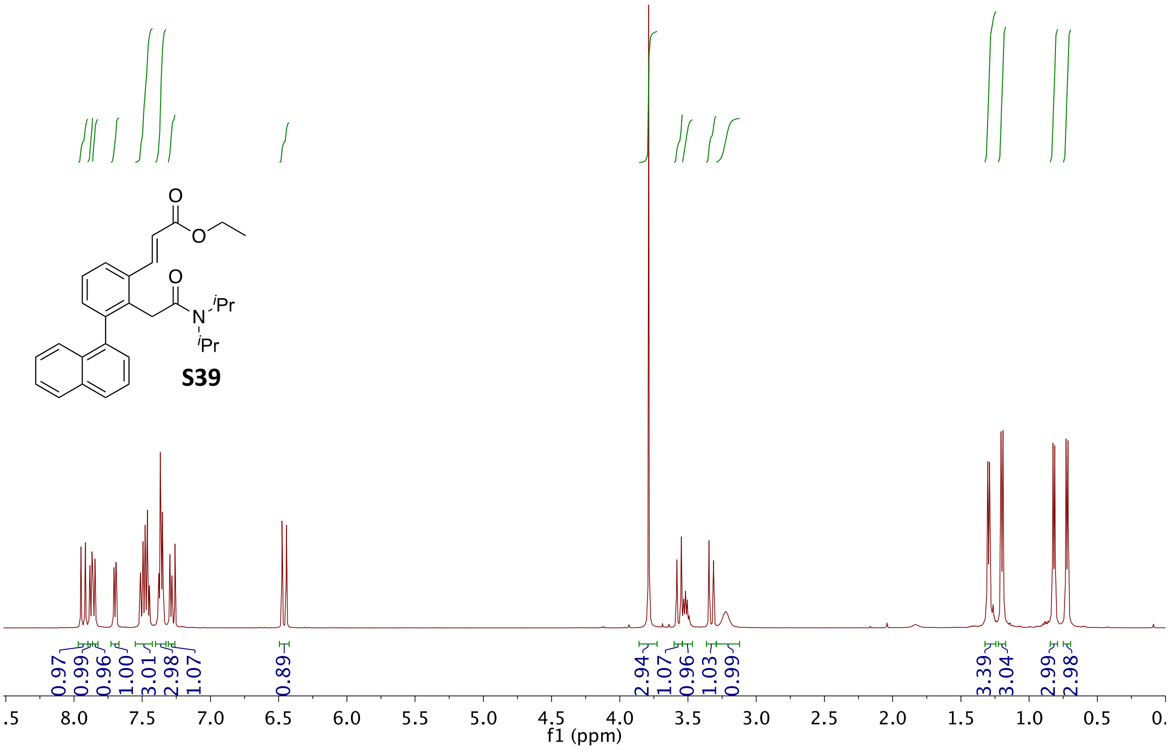
S39
0.97
0.99
0.96
1.00
3.01
2.98
1.07
0.89
2.94
1.07
0.96
1.03
0.99
3.39
3.04
2.99
2.98
f1 (ppm)

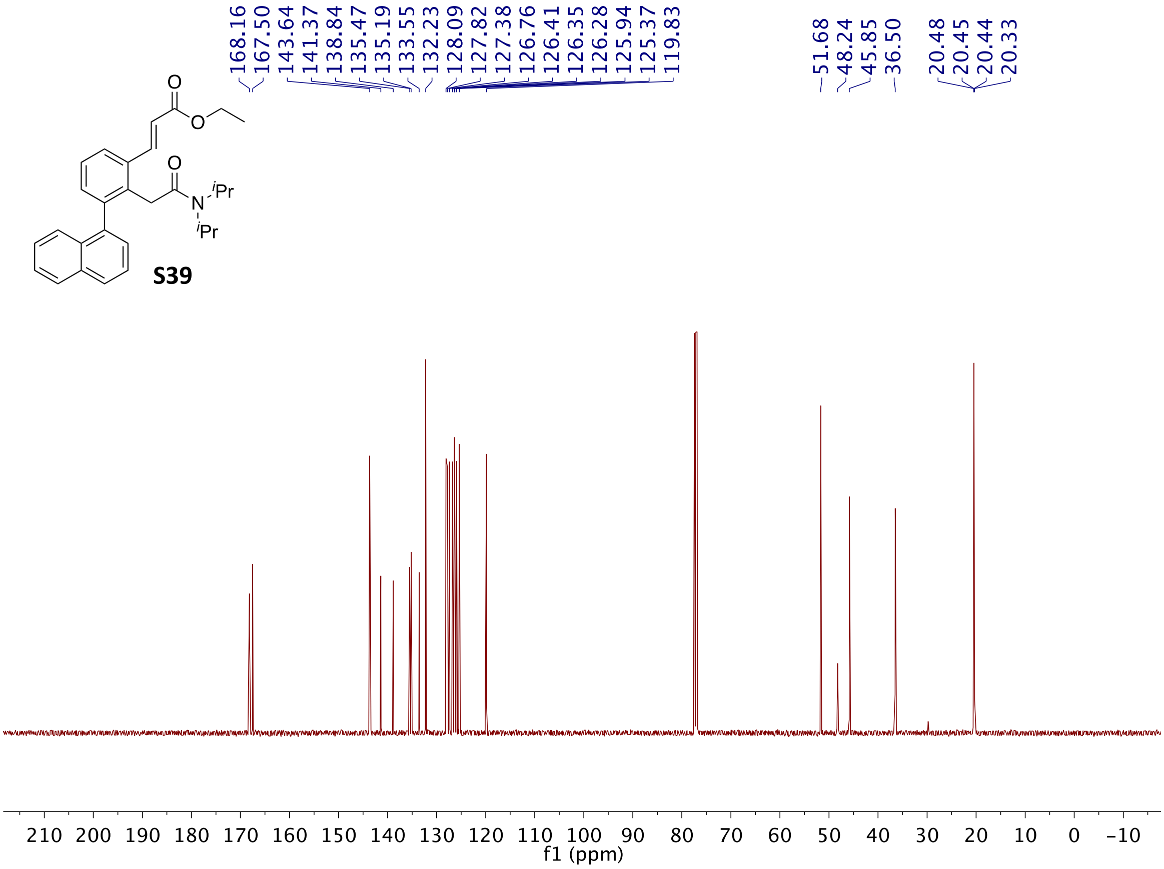
168.16
167.50
143.64
141.37
138.84
135.47
135.19
133.55
132.23
128.09
127.82
127.38
126.76
126.41
126.35
126.28
125.94
125.37
119.83
51.68
48.24
45.85
36.50
20.48
20.45
20.44
20.33
S39
f1 (ppm)

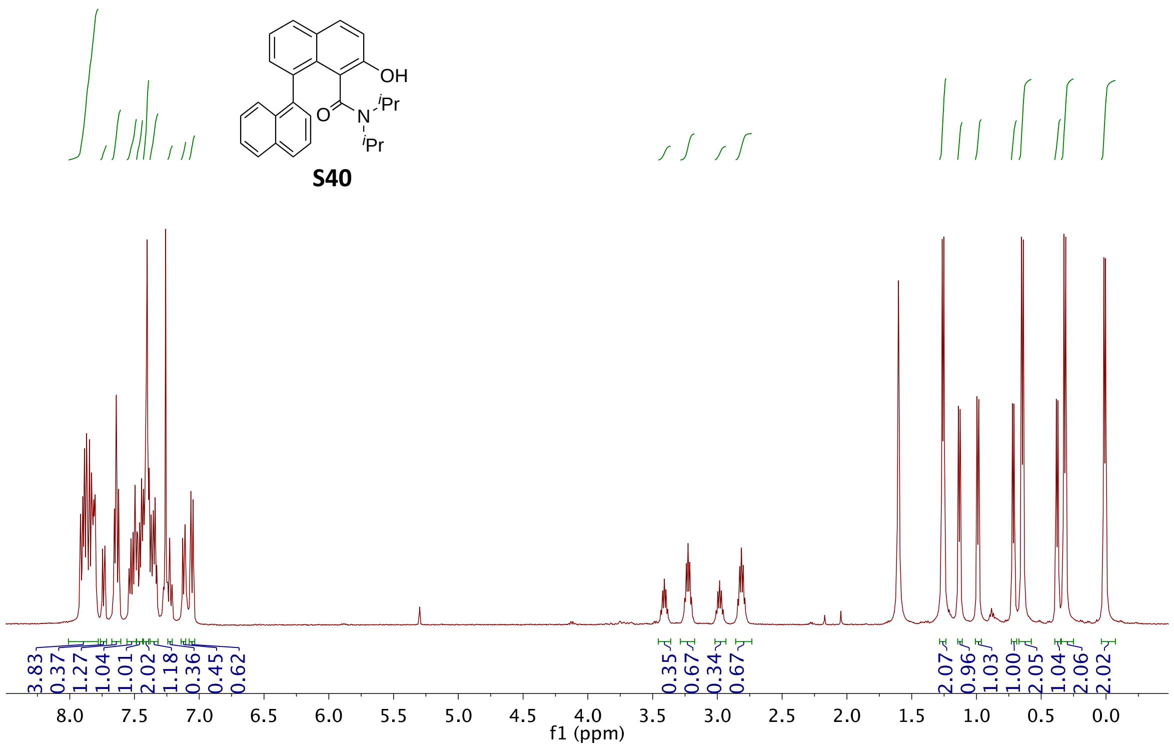
OH
iPr
N
O
iPr
S40
3.83 0.37 1.27 1.04 1.01 2.02 1.18 0.36 0.45 0.62
0.35 0.67 0.34 0.67
2.07 0.96 1.03 1.00 2.05 1.04 2.06 2.02
8.0 7.5 7.0 6.5 6.0 5.5 5.0 4.5 4.0 3.5 3.0 2.5 2.0 1.5 1.0 0.5 0.0
f1 (ppm)

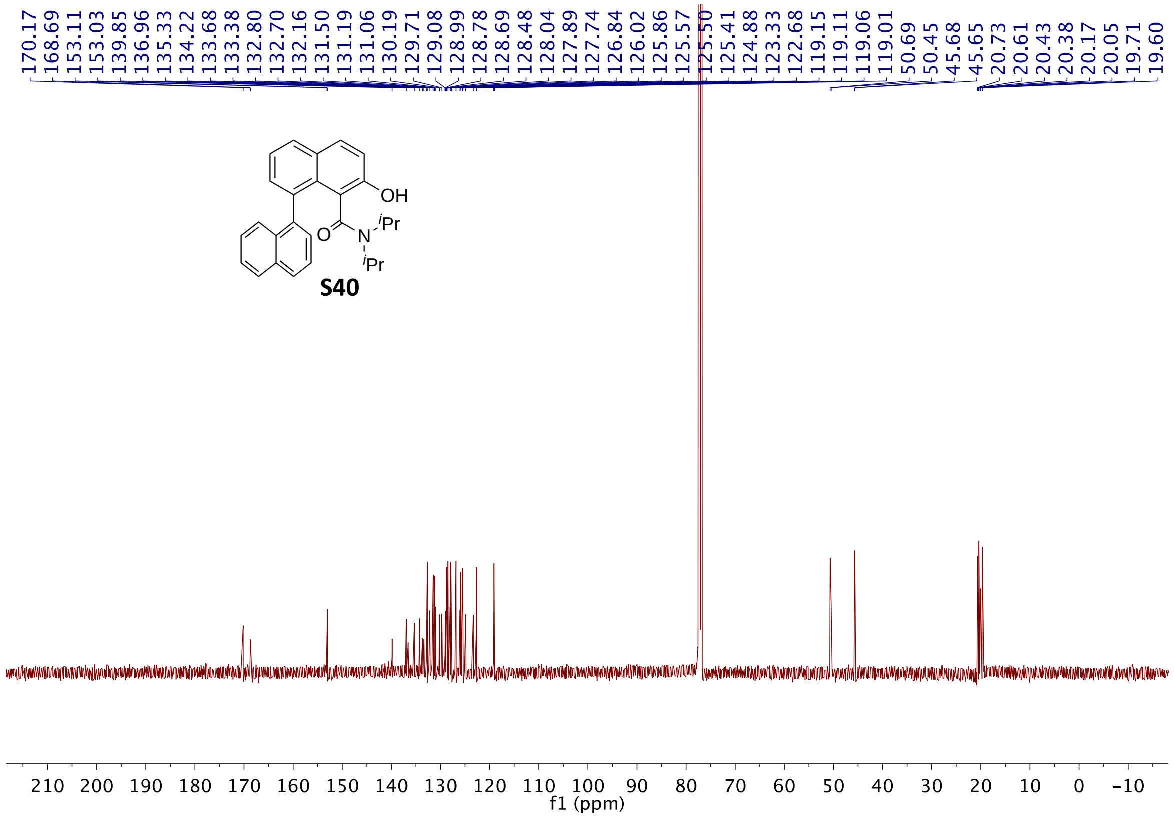
170.17 168.69 153.11 153.03 139.85 136.96 135.33 134.22 133.68 133.38 132.80 132.70 132.16 131.50 131.19 131.06 130.19 129.71 129.08 128.99 128.78 128.69 128.48 128.04 127.89 127.74 126.84 126.02 125.86 125.57 125.50 125.41 124.88 123.33 122.68 119.15 119.11 119.06 119.01
50.69 50.45 45.68 45.65 20.73 20.61 20.43 20.38 20.17 20.05 19.71 19.60
OH
iPr
N
O
iPr
S40
210 200 190 180 170 160 150 140 130 120 110 100 90 80 70 60 50 40 30 20 10 0 −10
f1 (ppm)

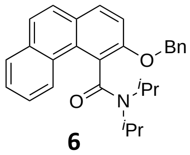

O
Bn
O
N
iPr
iPr

6

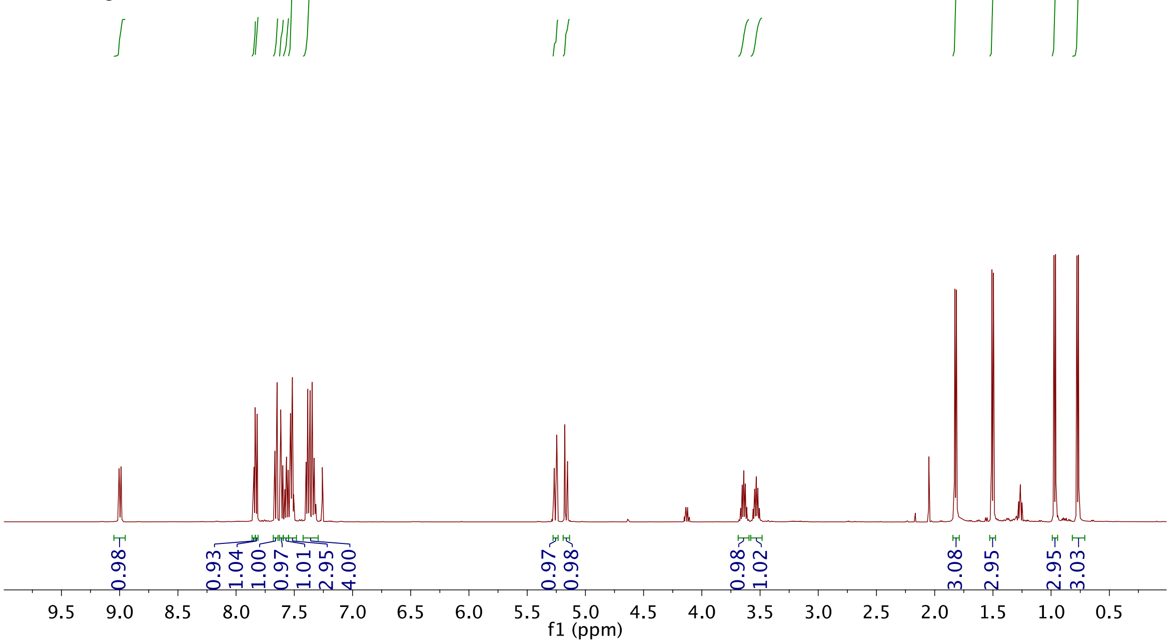

0.98
0.93
1.04
1.00
0.97
1.01
2.95
4.00
0.97
0.98
0.98
1.02
3.08
2.95
2.95
3.03
9.5 9.0 8.5 8.0 7.5 7.0 6.5 6.0 5.5 5.0 4.5 4.0 3.5 3.0 2.5 2.0 1.5 1.0 0.5
f1 (ppm)

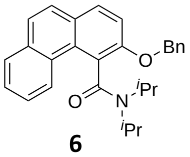

O
Bn
O
N
iPr
iPr

6

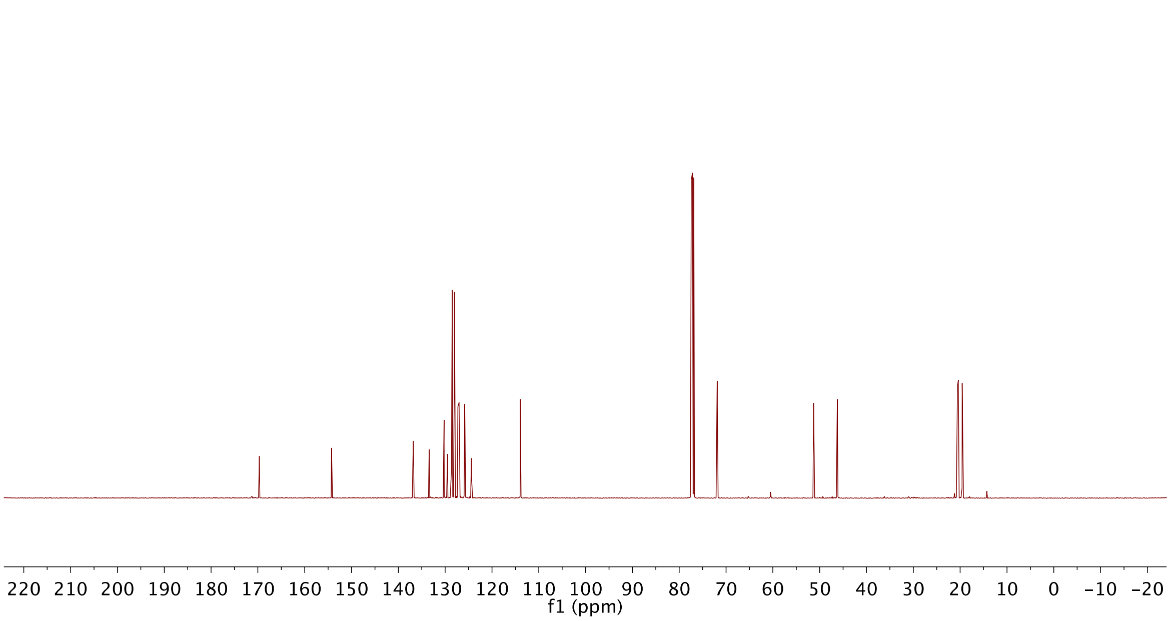

169.69
154.27
136.81
133.41
130.24
129.50
128.65
128.48
128.11
128.06
128.02
127.96
127.27
127.03
126.92
125.82
125.72
124.38
113.94
71.90
51.30
46.24
20.64
20.57
20.40
19.57
220 210 200 190 180 170 160 150 140 130 120 110 100 90 80 70 60 50 40 30 20 10 0 −10 −20
f1 (ppm)

Chiral HPLC: (Chiralpak ODH, 30% iso, 70% hexane, 1.0 mL min^{-1}, λ = 254nm) τ_R (major) = 10.3 min, τ_R (minor) = 15.6 min.

Asymmetric

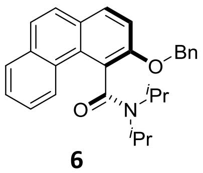

6

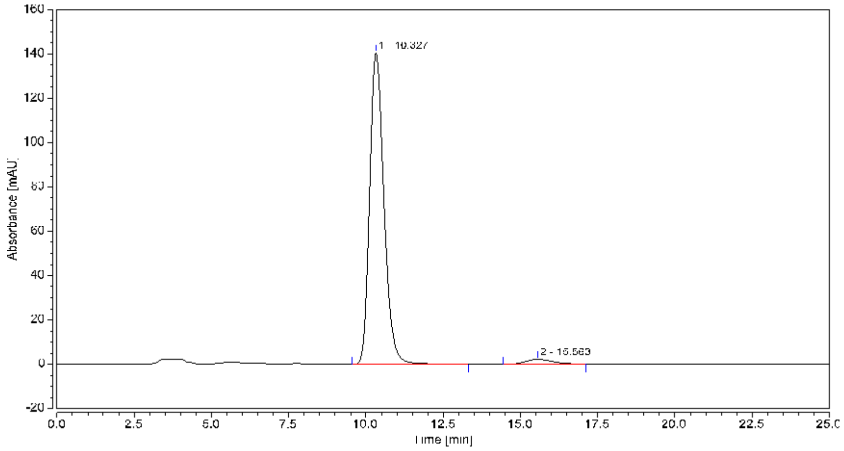

No.	Peak Name	Retention Time	Area	Height	Relative Area
		min	mAU*min	mAU	%
1		10.327	75.352	140.437	97.18
2		15.563	2.187	2.198	2.82
**Total:**			77.540	142.635	100.00

Racemic

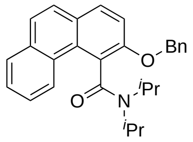

6

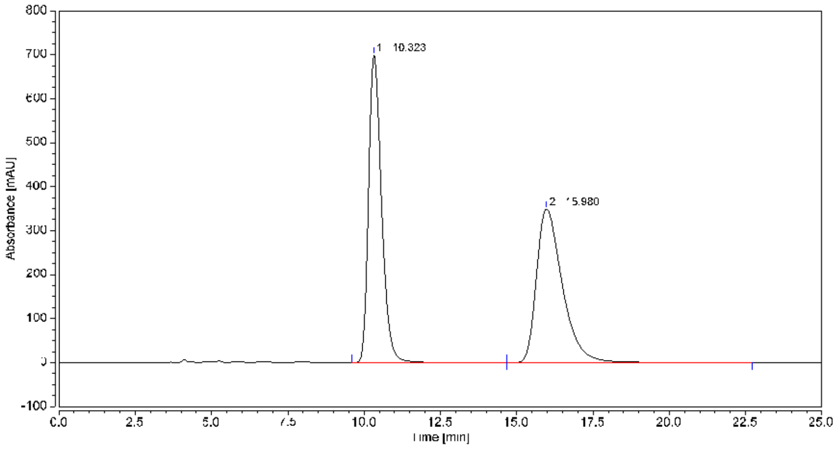

No.	Peak Name	Retention Time	Area	Height	Relative Area
		min	mAU*min	mAU	%
1		10.323	344.889	699.439	50.02
2		15.980	344.608	349.855	49.98
**Total:**			689.497	1049.293	100.00

12

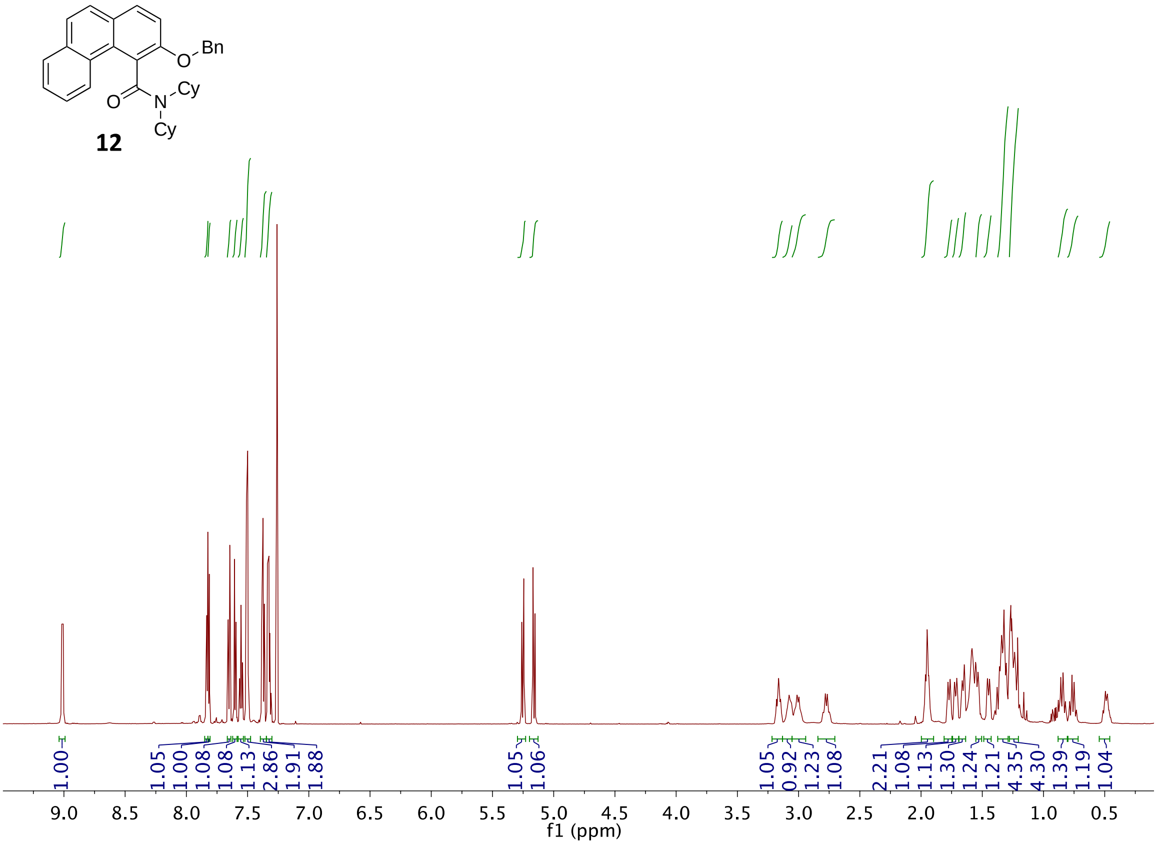
O
Bn
Cy
N
Cy
1.00
1.05
1.00
1.08
1.08
1.13
2.86
1.91
1.88
1.05
1.06
1.05
0.92
1.23
1.08
2.21
1.08
1.13
1.30
1.24
1.21
4.35
4.30
1.39
1.19
1.04
9.0 8.5 8.0 7.5 7.0 6.5 6.0 5.5 5.0 4.5 4.0 3.5 3.0 2.5 2.0 1.5 1.0 0.5
f1 (ppm)

12

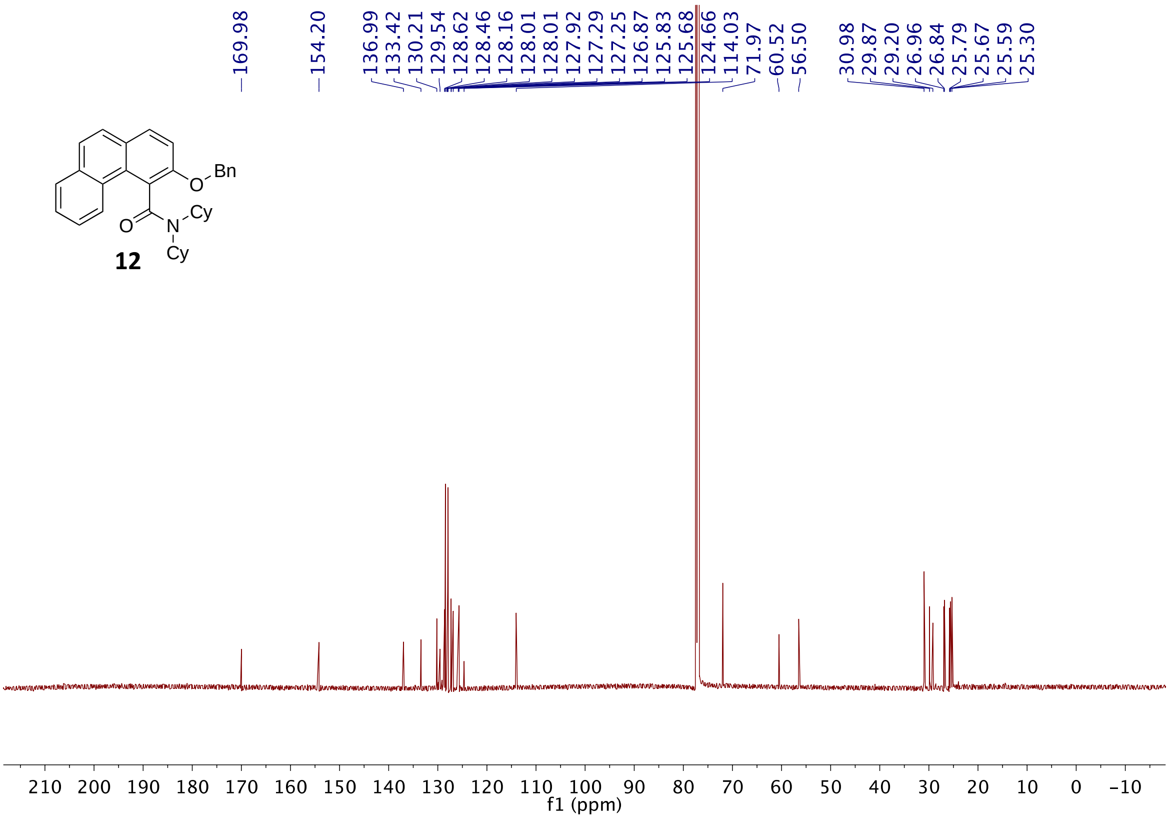
169.98
154.20
136.99
133.42
130.21
129.54
128.62
128.46
128.16
128.01
128.01
127.92
127.29
127.25
126.87
125.83
125.68
124.66
114.03
71.97
60.52
56.50
30.98
29.87
29.20
26.96
26.84
25.79
25.67
25.59
25.30
O
Bn
Cy
N
Cy
210 200 190 180 170 160 150 140 130 120 110 100 90 80 70 60 50 40 30 20 10 0 −10
f1 (ppm)

Chiral HPLC: (Chiralpak ADH, 30% isopropanol, 70% hexane, 1.0 mL min^{-1}, λ = 260 nm) τ_R (major) = 6.8 min, τ_R (minor) = 10.9 min.

Asymmetric

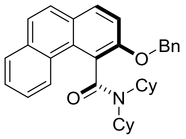

12

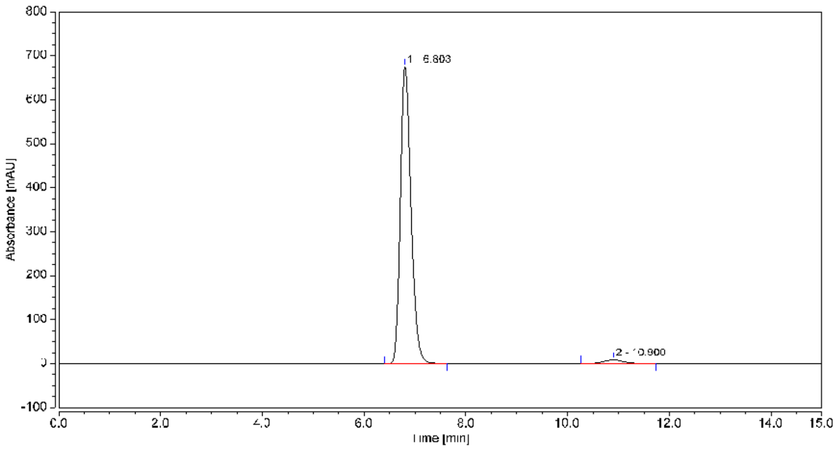

No.	Peak Name	Retention Time	Area	Height	Relative Area
		min	mAU*min	mAU	%
1		6.803	163.765	675.693	97.63
2		10.900	3.975	8.518	2.37
**Total:**			167.740	684.211	100.00

Racemic

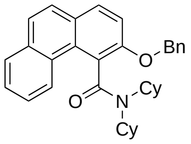

12

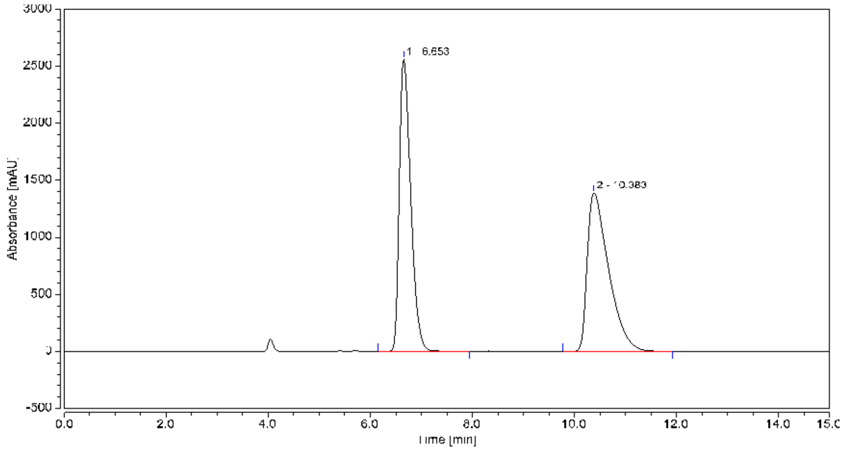

No.	Peak Name	Retention Time	Area	Height	Relative Area
		min	mAU*min	mAU	%
1		6.653	662.472	2563.567	49.53
2		10.383	675.131	1391.741	50.47
**Total:**			1337.603	3955.309	100.00

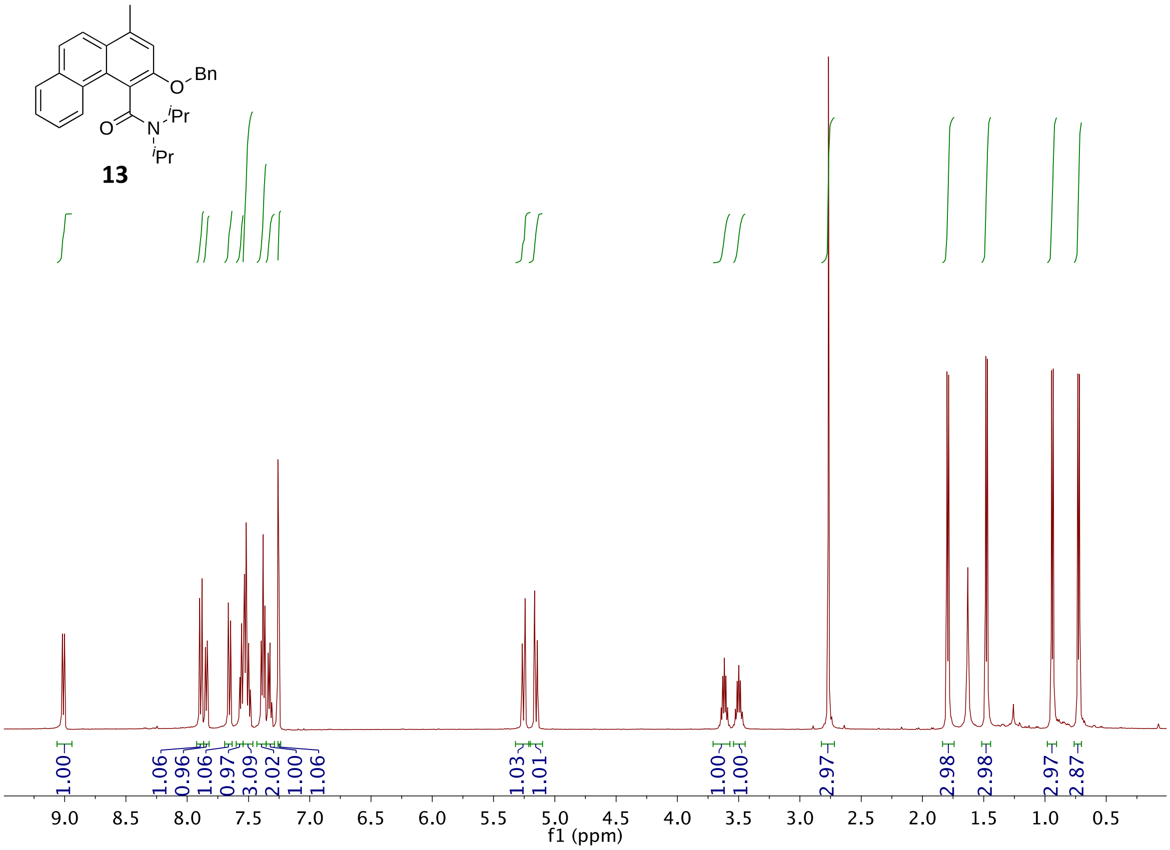
O
Bn
N
iPr
iPr
O
13
1.00
1.06
0.96
1.06
0.97
3.09
2.02
1.00
1.06
1.03
1.01
1.00
1.00
2.97
2.98
2.98
2.97
2.87
9.0 8.5 8.0 7.5 7.0 6.5 6.0 5.5 5.0 4.5 4.0 3.5 3.0 2.5 2.0 1.5 1.0 0.5
f1 (ppm)

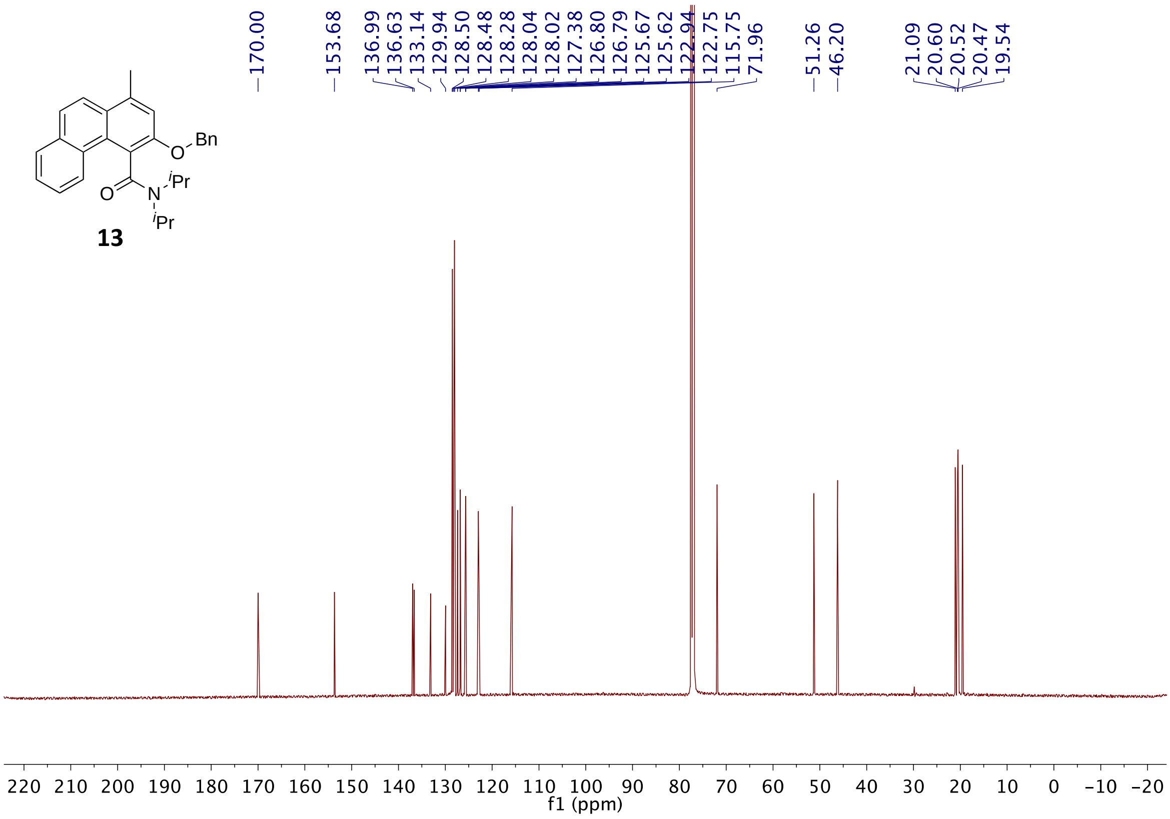
170.00
153.68
136.99
136.63
133.14
129.94
128.50
128.48
128.28
128.04
128.02
127.38
126.80
126.79
125.67
125.62
122.94
122.75
115.75
71.96
51.26
46.20
21.09
20.60
20.52
20.47
19.54
O
Bn
N
iPr
iPr
O
13
220 210 200 190 180 170 160 150 140 130 120 110 100 90 80 70 60 50 40 30 20 10 0 −10 −20
f1 (ppm)

Chiral HPLC: (Chiralpak ODH, 30% ^{i}PrOH, 70% hexane, 1.0 mL min^{-1}, λ = 310 nm) τ_R (major) = 17.7 min, τ_R (minor) = 23.5 min.

Asymmetric

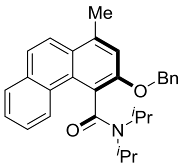

13

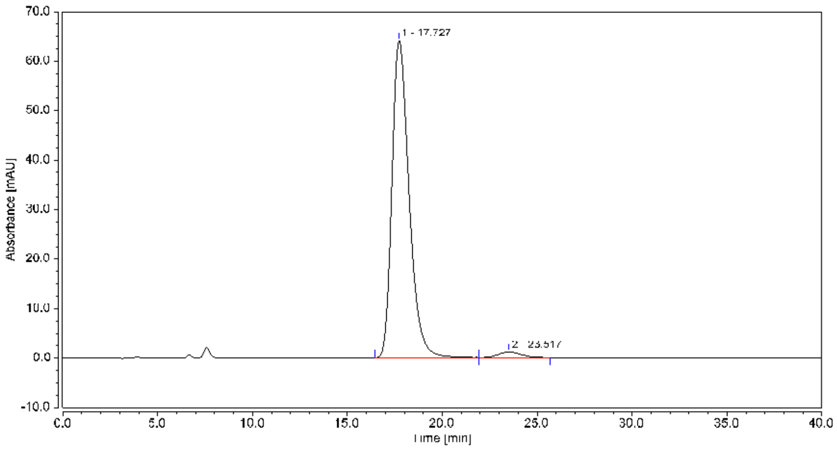

No.	Peak Name	Retention Time	Area	Height	Relative Area
		min	mAU*min	mAU	%
1		17.727	66.831	64.148	97.64
2		23.517	1.612	1.144	2.36
**Total:**			198.122	189.183	100.00

Racemic

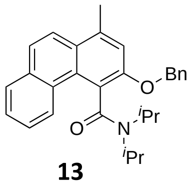

13

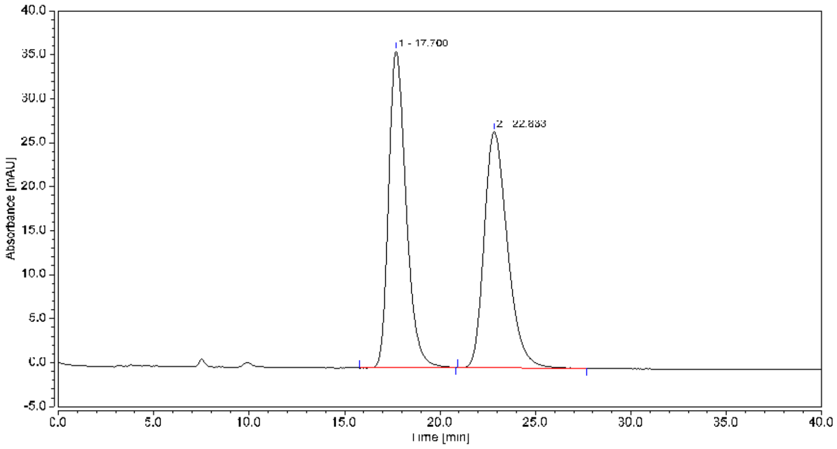

No.	Peak Name	Retention Time	Area	Height	Relative Area
		min	mAU*min	mAU	%
1		17.717	38.708	36.467	50.08
2		23.090	38.577	26.939	49.92
**Total:**			77.284	63.405	100.00

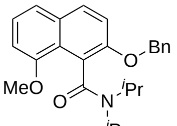

O
Bn
MeO
O
N
iPr
iPr

14

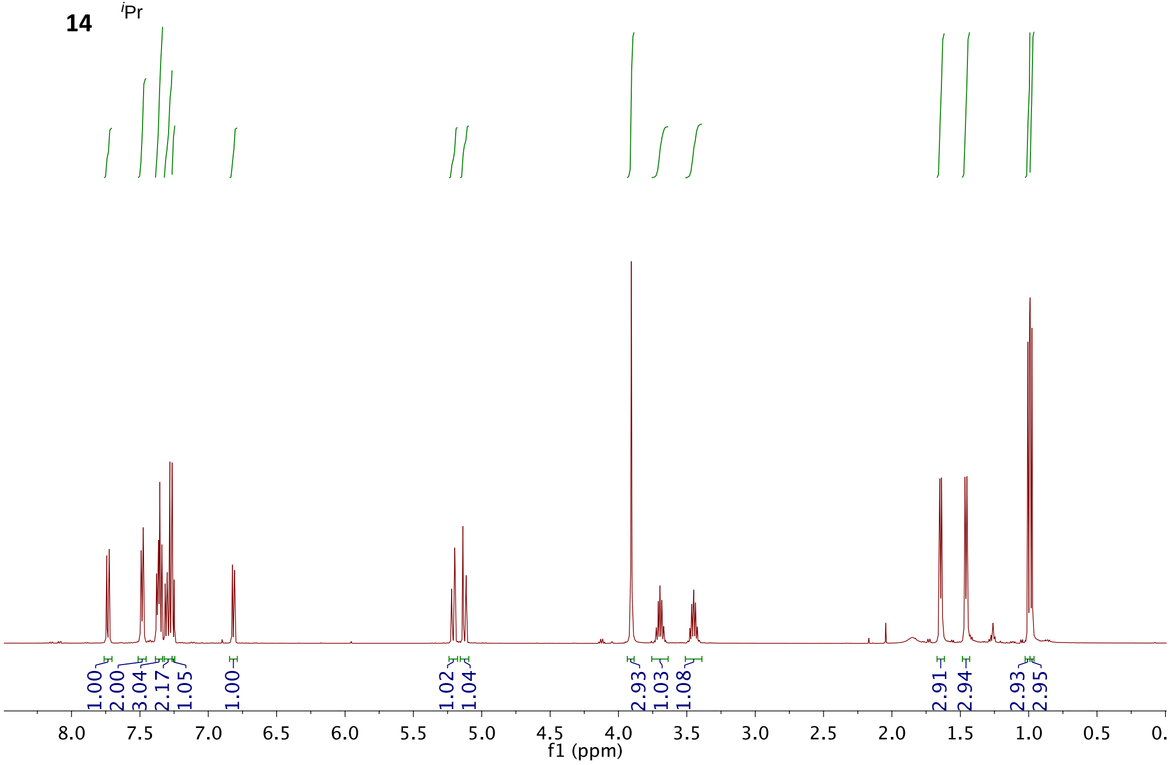

1.00
2.00
3.04
2.17
1.05
1.00
1.02
1.04
2.93
1.03
1.08
2.91
2.94
2.93
2.95
8.0 7.5 7.0 6.5 6.0 5.5 5.0 4.5 4.0 3.5 3.0 2.5 2.0 1.5 1.0 0.5 0.
f1 (ppm)

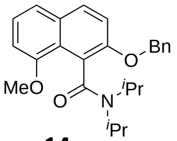

O
Bn
MeO
O
N
iPr
iPr

14

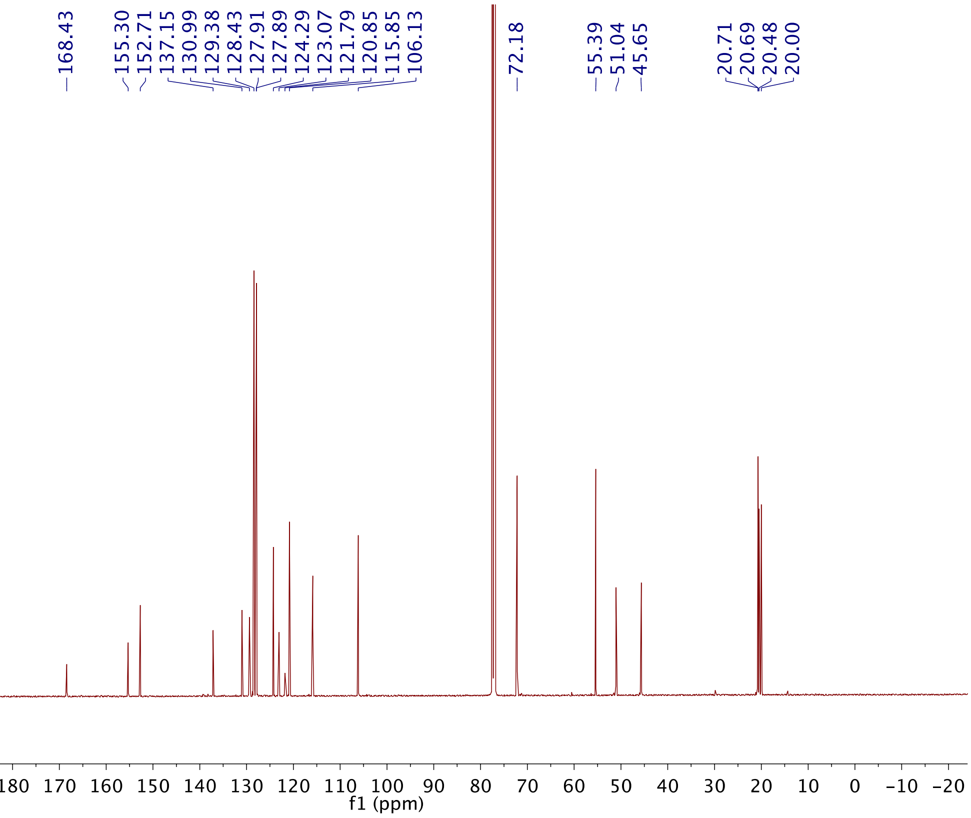

168.43
155.30
152.71
137.15
130.99
129.38
128.43
127.91
127.89
124.29
123.07
121.79
120.85
115.85
106.13
72.18
55.39
51.04
45.65
20.71
20.69
20.48
20.00
220 210 200 190 180 170 160 150 140 130 120 110 100 90 80 70 60 50 40 30 20 10 0 −10 −20
f1 (ppm)

Chiral HPLC: (Chiralpak IA,40% isopropanol, 60% hexane, 1.0 mL min^{-1}, λ =240 nm,) τ_R (major) = 6.5 min, τ_R (minor) = 11.5 min.

Asymmetric

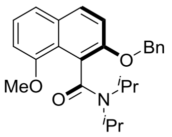

14

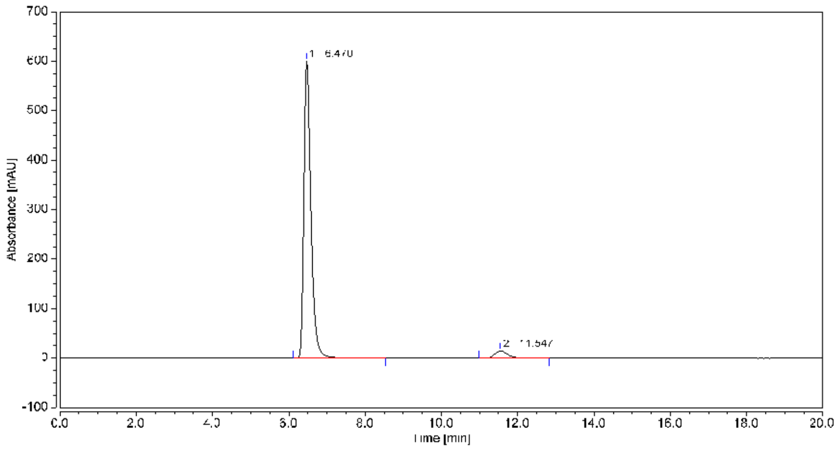

No.	Peak Name	Retention Time	Area	Height	Relative Area
		min	mAU*min	mAU	%
1		6.470	129.754	600.478	95.57
2		11.547	6.014	14.717	4.43
**Total:**			135.768	615.194	100.00

Racemic

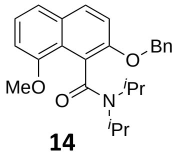

14

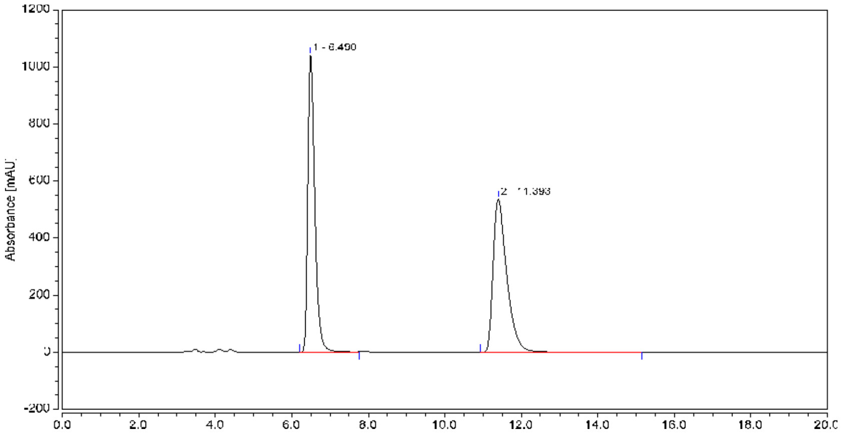

No.	Peak Name	Retention Time	Area	Height	Relative Area
		min	mAU*min	mAU	%
1		6.490	222.282	1042.870	49.95
2		11.393	222.743	537.144	50.05
**Total:**			445.025	1580.014	100.00

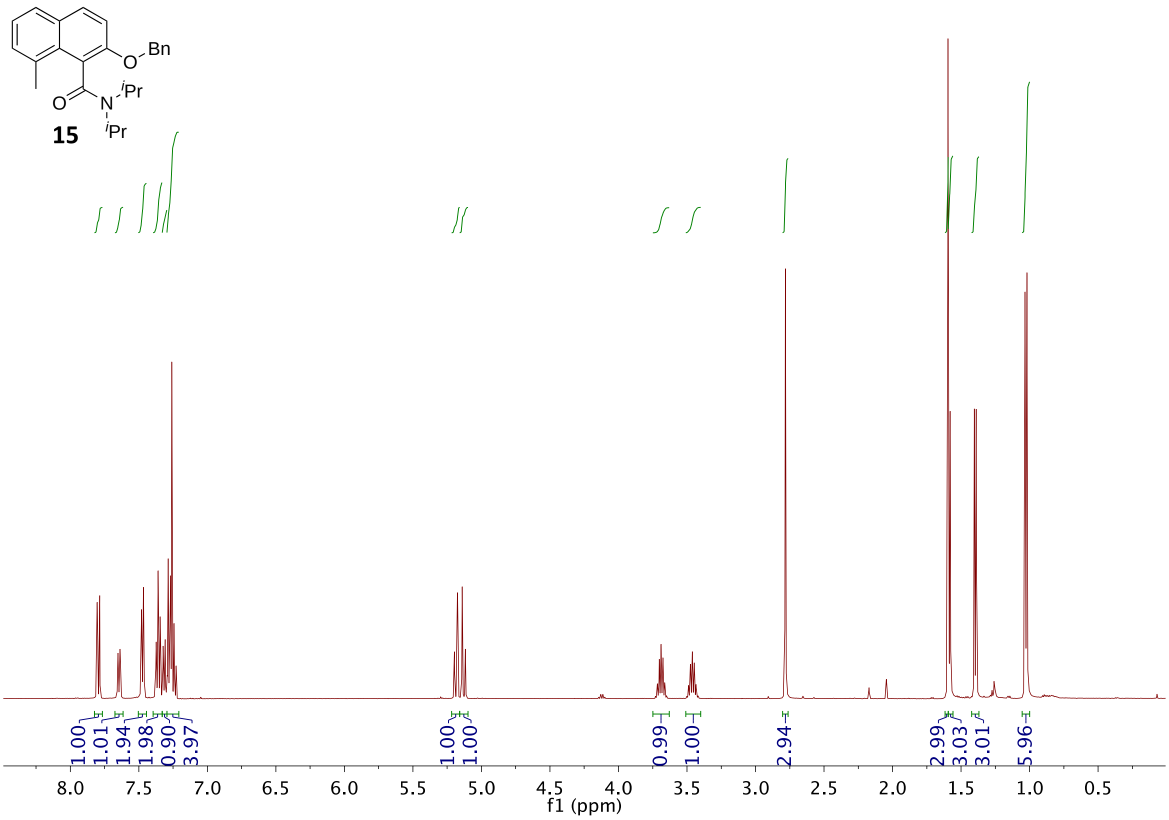

O
Bn
N
^{i}Pr
O
^{i}Pr
8.0 7.5 7.0 6.5 6.0 5.5 5.0 4.5 4.0 3.5 3.0 2.5 2.0 1.5 1.0 0.5
f1 (ppm)
1.00
1.01
1.94
1.98
0.90
3.97
1.00
1.00
0.99
1.00
2.94
2.99
3.03
3.01
5.96

15

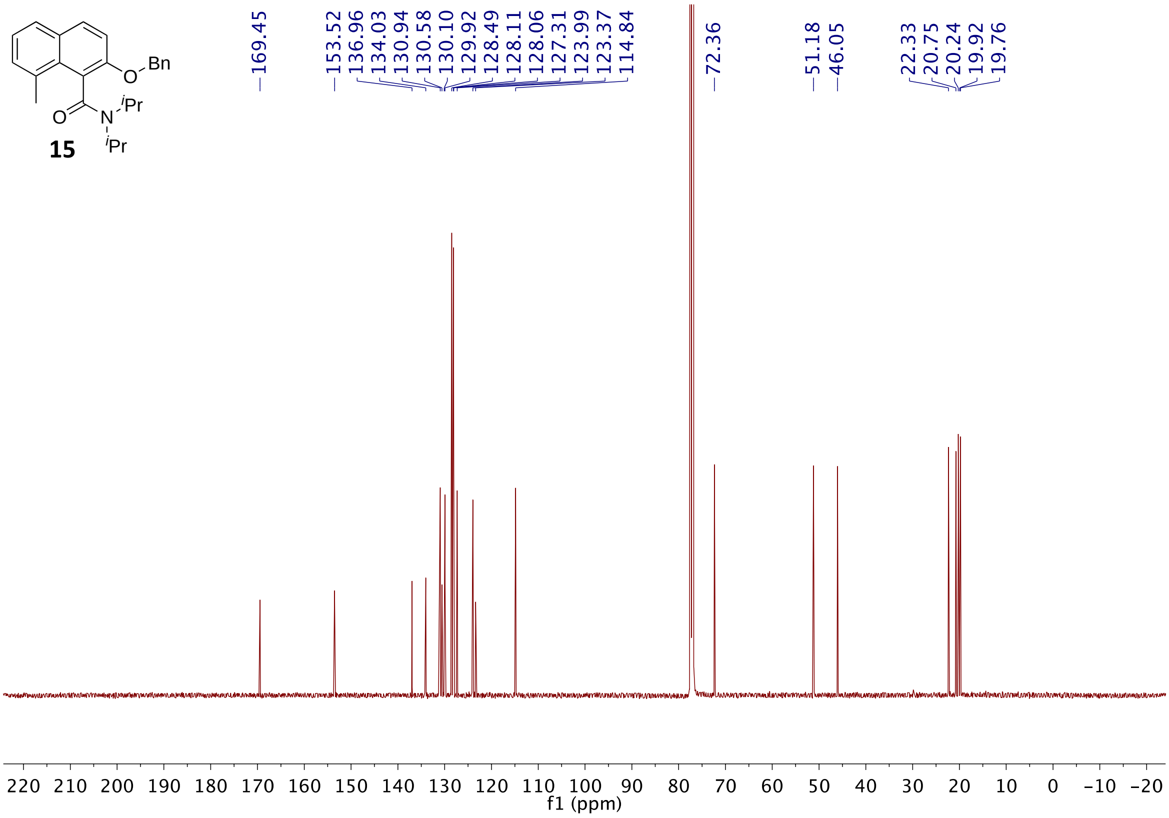

O
Bn
N
^{i}Pr
O
^{i}Pr
169.45
153.52
136.96
134.03
130.94
130.58
130.10
129.92
128.49
128.11
128.06
127.31
123.99
123.37
114.84
72.36
51.18
46.05
22.33
20.75
20.24
19.92
19.76
220 210 200 190 180 170 160 150 140 130 120 110 100 90 80 70 60 50 40 30 20 10 0 −10 −20
f1 (ppm)

15

Chiral HPLC: (Chiralpak ODH, 20% isopropanol, 80% hexane, 1.0 mL min^{-1}, λ = 240 nm) τ_R (major) = 6.8 min, τ_R (minor) = 8.7 min.

Asymmetric

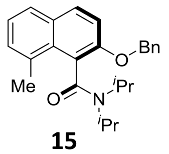

15

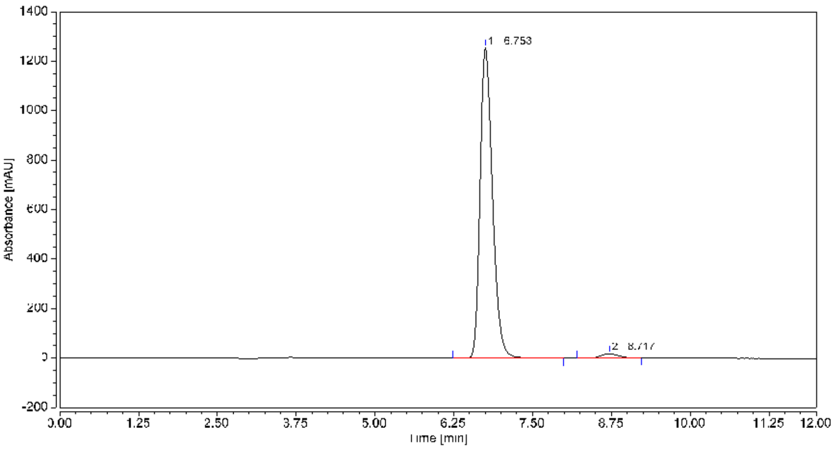

No.	Peak Name	Retention Time	Area	Height	Relative Area
		min	mAU*min	mAU	%
1		6.753	286.902	1253.815	97.92
2		8.717	6.103	18.090	2.08
**Total:**			293.004	1271.904	100.00

Racemic

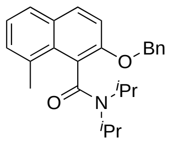

15

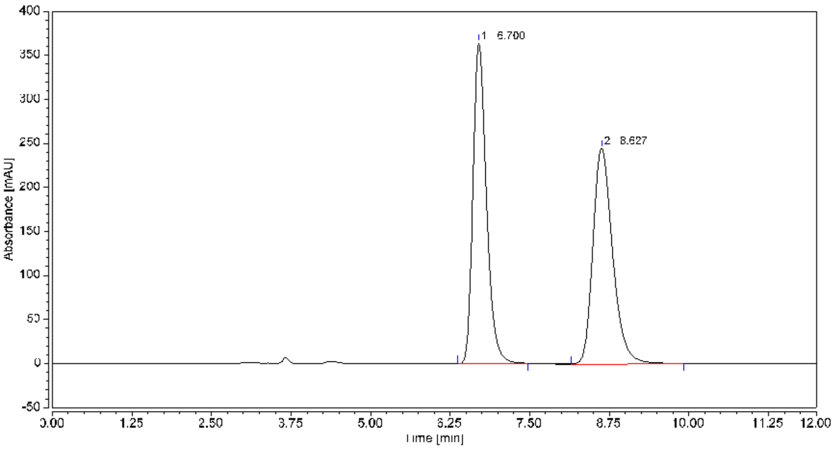

No.	Peak Name	Retention Time	Area	Height	Relative Area
		min	mAU*min	mAU	%
1		6.700	89.031	364.371	50.24
2		8.627	88.192	245.769	49.76
**Total:**			177.222	610.141	100.00

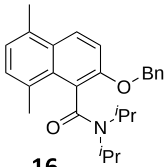

16

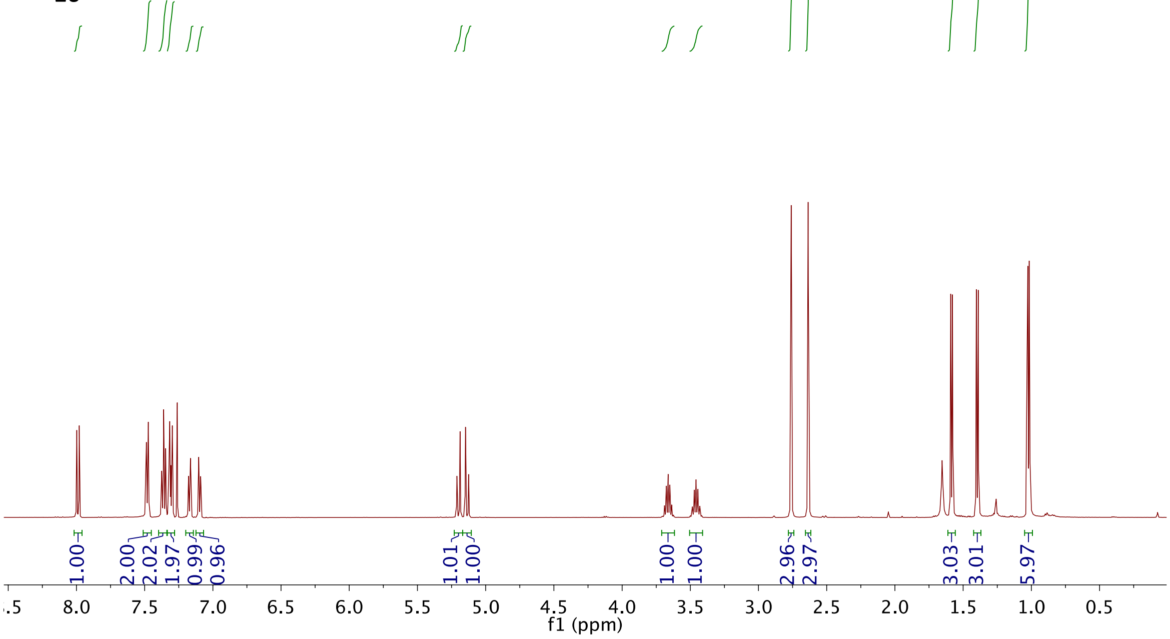
1.00
2.00
2.02
1.97
0.99
0.96
1.01
1.00
1.00
1.00
2.96
2.97
3.03
3.01
5.97
f1 (ppm)

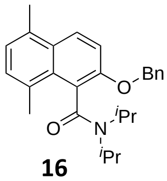

16

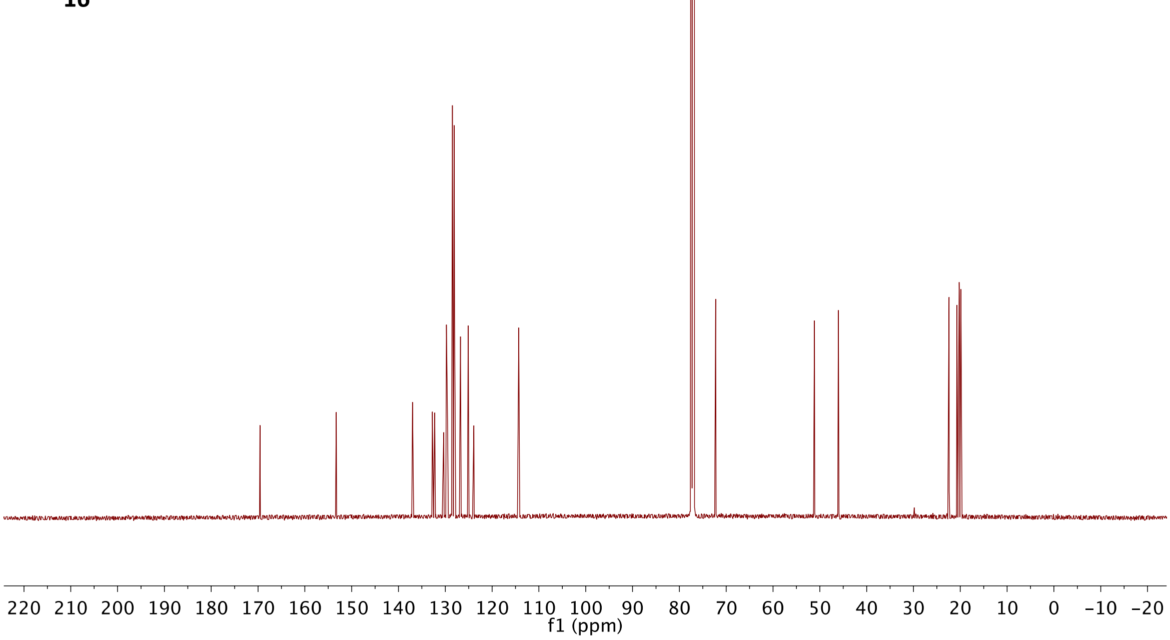
169.57
153.30
137.00
132.76
132.26
130.32
129.76
129.50
128.48
128.11
128.03
126.76
125.10
123.91
114.33
72.24
51.15
46.04
22.39
20.71
20.24
20.18
19.87
19.75
f1 (ppm)

Chiral HPLC: (Chiralpak ODH, 5% isopropanol, 95% hexane, 1.0 mL min^{-1}, λ = 300 nm) τ_R (major) = 24.8 min, τ_R (minor) = 31.6 min.

Asymmetric

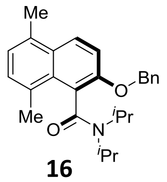

16

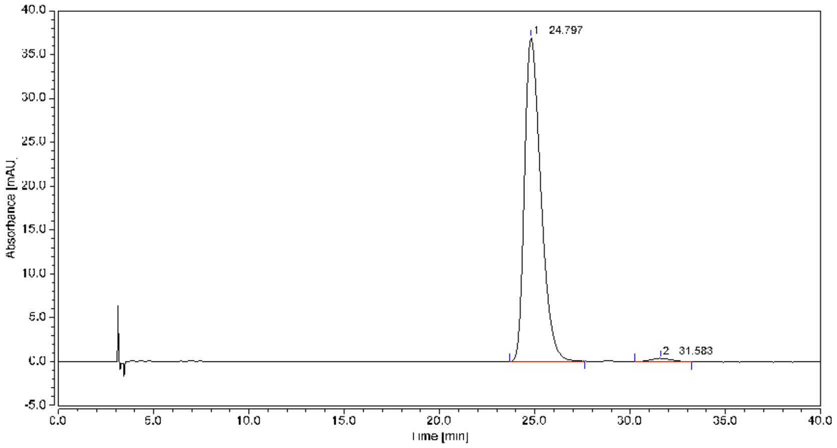

No.	Peak Name	Retention Time	Area	Height	Relative Area
		min	mAU*min	mAU	%
1		24.797	36.693	36.920	98.51
2		31.583	0.555	0.415	1.49
**Total:**			37.248	37.335	100.00

Racemic

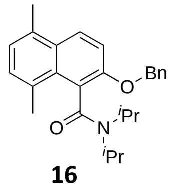

16

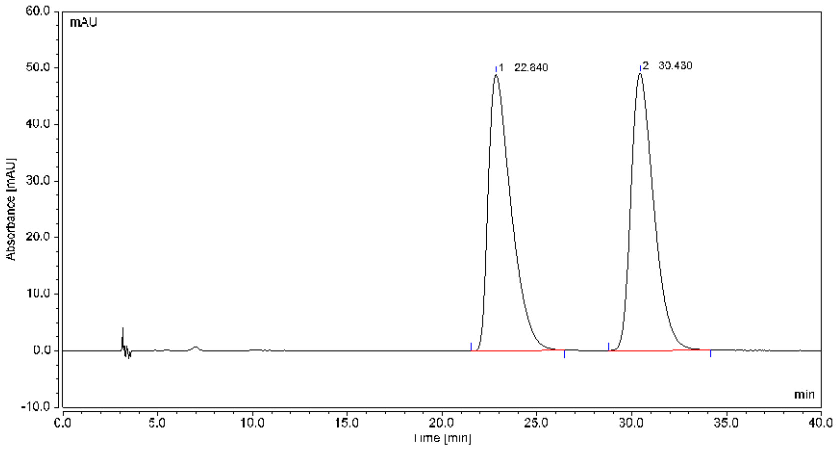

No.	Peak Name	Retention Time	Area	Height	Relative Area
		min	mAU*min	mAU	%
1		22.840	68.145	48.795	49.95
2		30.430	68.289	49.027	50.05
**Total:**			136.434	97.822	100.00

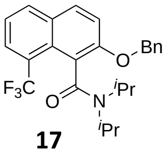
Bn
O
F3C
O
N
iPr
iPr

17

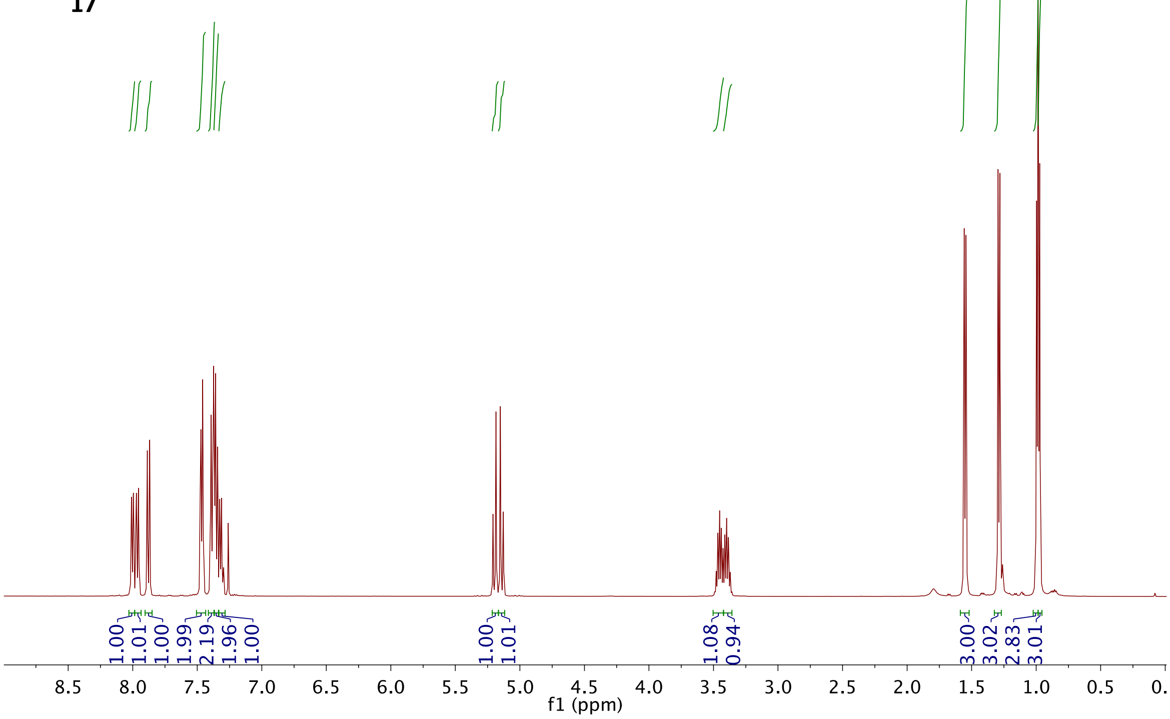
1.00
1.01
1.00
1.99
2.19
1.96
1.00
1.00
1.01
1.08
0.94
3.00
3.02
2.83
3.01
8.5 8.0 7.5 7.0 6.5 6.0 5.5 5.0 4.5 4.0 3.5 3.0 2.5 2.0 1.5 1.0 0.5 0.
f1 (ppm)

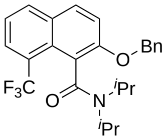
Bn
O
F3C
O
N
iPr
iPr

17

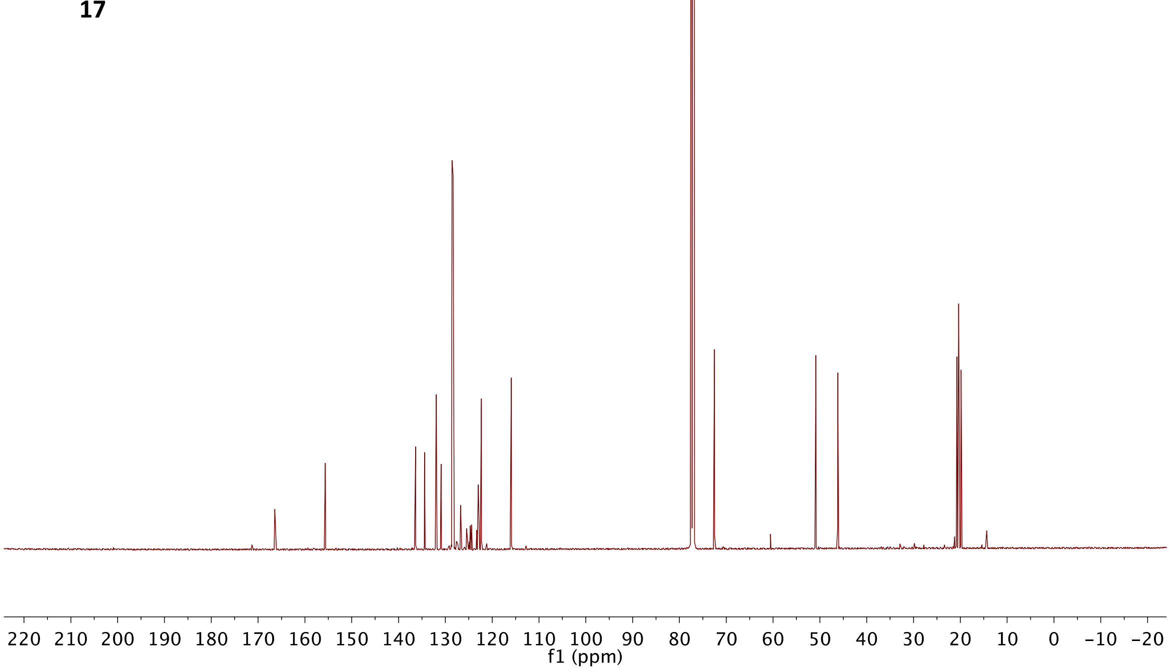
166.44
155.65
136.36
134.46
131.94
130.92
128.58
128.58
128.53
128.47
128.35
128.27
127.64
127.32
126.75
125.47
124.90
124.65
124.41
124.16
123.30
122.97
122.31
121.12
115.90
72.51
50.85
46.20
20.73
20.36
19.85
220 210 200 190 180 170 160 150 140 130 120 110 100 90 80 70 60 50 40 30 20 10 0 −10 −20
f1 (ppm)

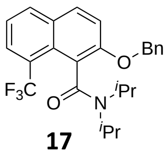

17

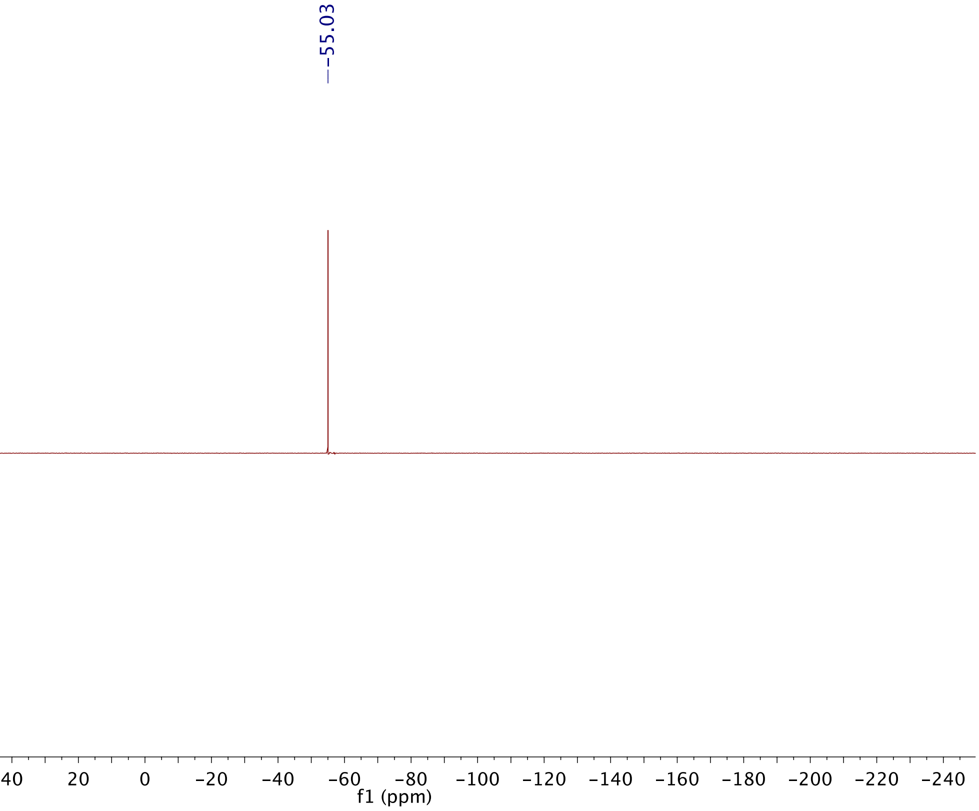

-55.03
80
60
40
20
0
−20
−40
−60
−80
−100
−120
−140
−160
−180
−200
−220
−240
f1 (ppm)

Chiral HPLC: (Chiralpak ODH, 10% ^{i}PrOH, 90% hexane, 1.0 mL min^{-1}, λ = 240 nm) τ_R (major) = 12.0 min, τ_R (minor) = 25.3 min.

Asymmetric

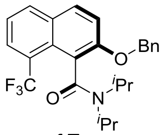

17

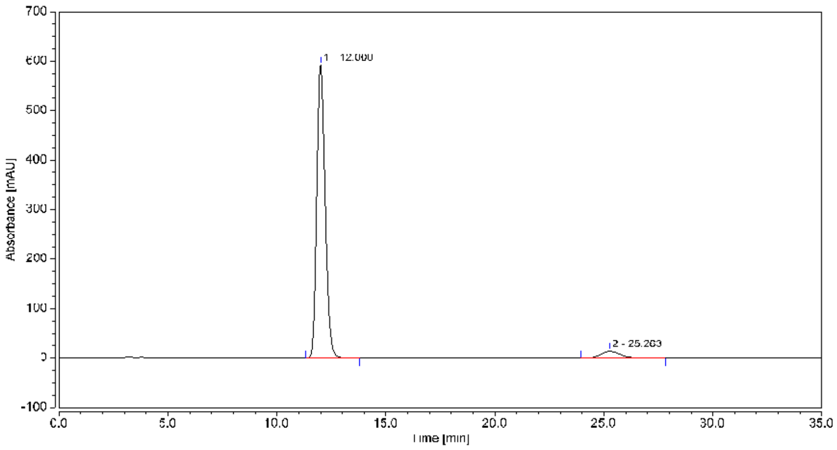

No.	Peak Name	Retention Time	Area	Height	Relative Area
		min	mAU*min	mAU	%
1		12.000	263.065	592.603	94.77
2		25.263	14.526	13.908	5.23
**Total:**			277.590	606.511	100.00

Racemic

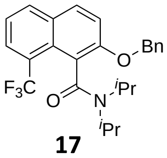

17

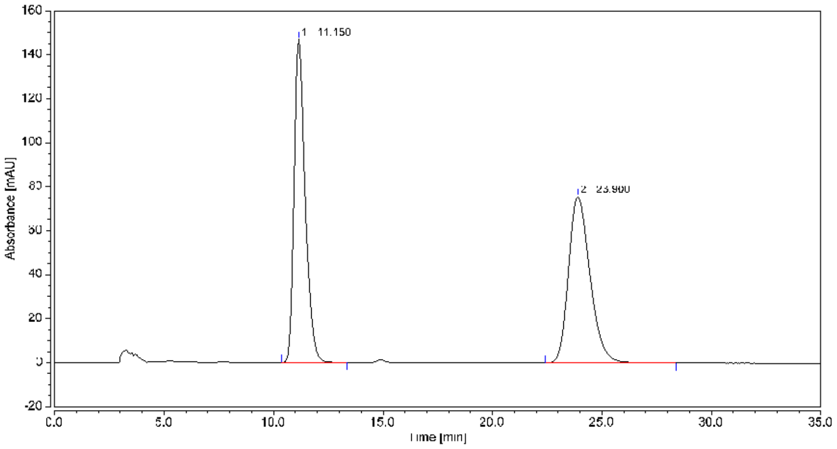

No.	Peak Name	Retention Time	Area	Height	Relative Area
		min	mAU*min	mAU	%
1		11.150	85.840	146.874	49.97
2		23.900	85.928	75.603	50.03
**Total:**			171.768	222.477	100.00

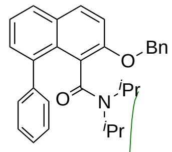

18

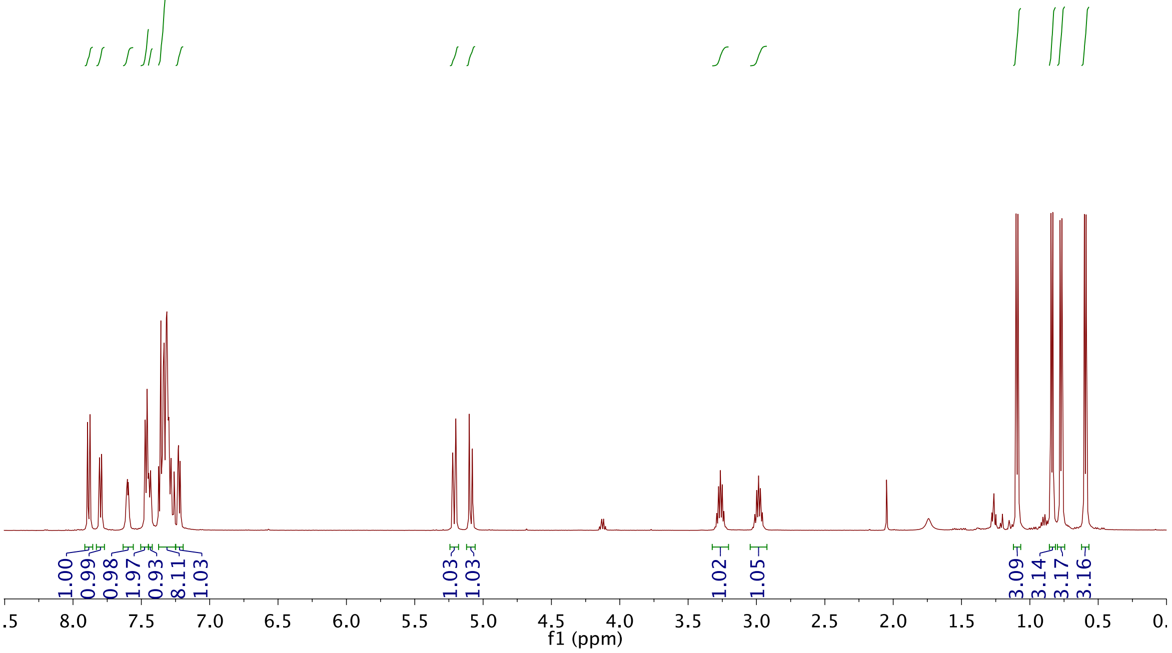

1.00
0.99
0.98
1.97
0.93
8.11
1.03
1.03
1.03
1.02
1.05
3.09
3.14
3.17
3.16
f1 (ppm)

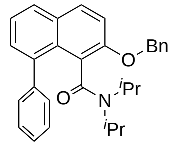

18

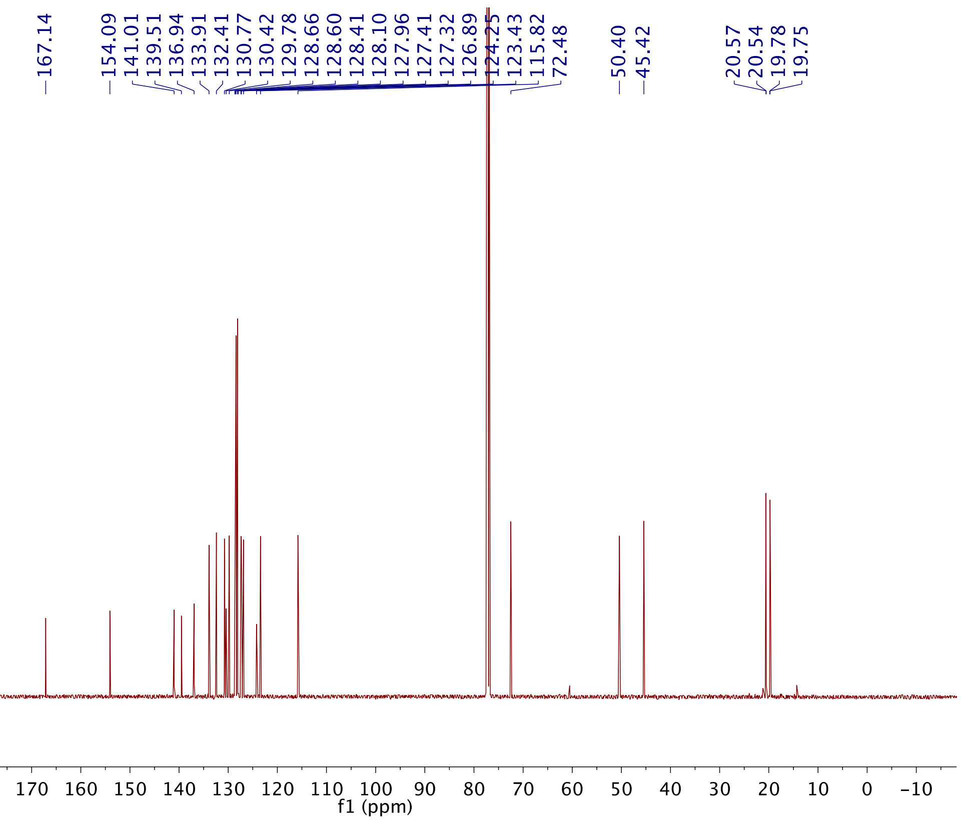

167.14
154.09
141.01
139.51
136.94
133.91
132.41
130.77
130.42
129.78
128.66
128.60
128.41
128.10
127.96
127.41
127.32
126.89
124.25
123.43
115.82
72.48
50.40
45.42
20.57
20.54
19.78
19.75
f1 (ppm)

Chiral HPLC: (Chiralpak IC, 10% isopropanol, 90% hexane, 1.0 mL min^{-1}, λ = 240 nm) τ_R (minor) = 11.8 min, τ_R (major) = 25.3 min.

Asymmetric

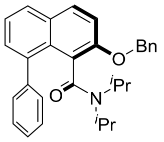

18

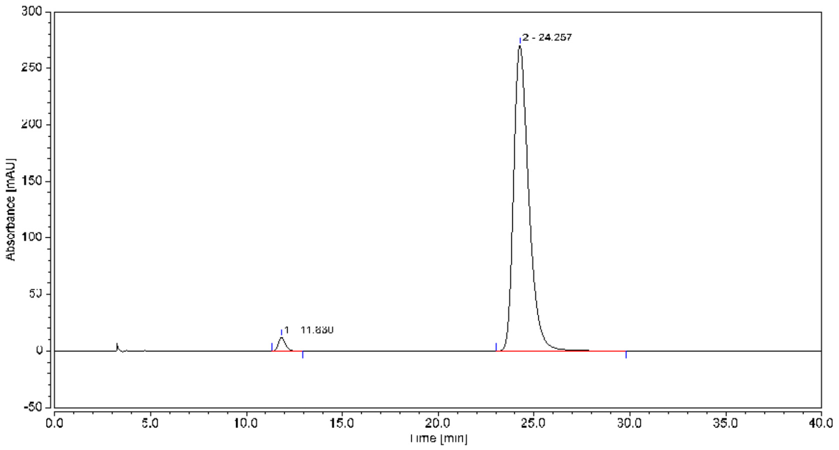

No.	Peak Name	Retention Time	Area	Height	Relative Area
		min	mAU*min	mAU	%
1		11.830	4.980	12.206	1.98
2		24.257	246.559	270.482	98.02
**Total:**			251.539	282.687	100.00

Racemic

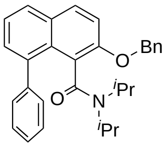

18

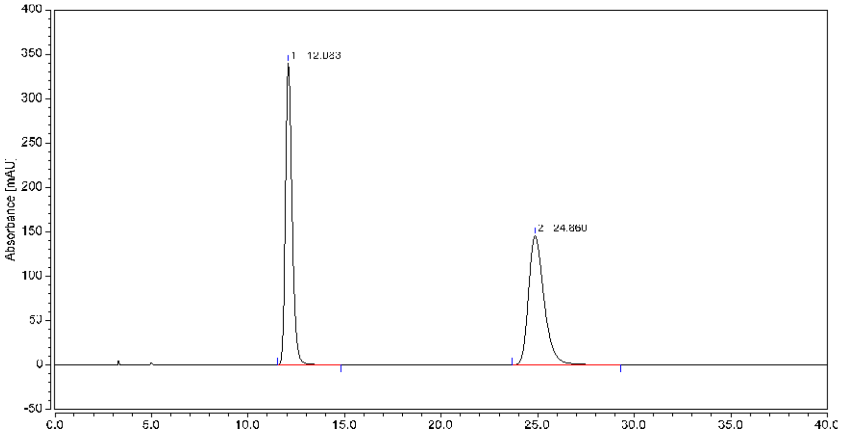

No.	Peak Name	Retention Time	Area	Height	Relative Area
		min	mAU*min	mAU	%
1		12.083	136.050	339.968	50.11
2		24.860	135.445	145.556	49.89
**Total:**			271.494	485.524	100.00

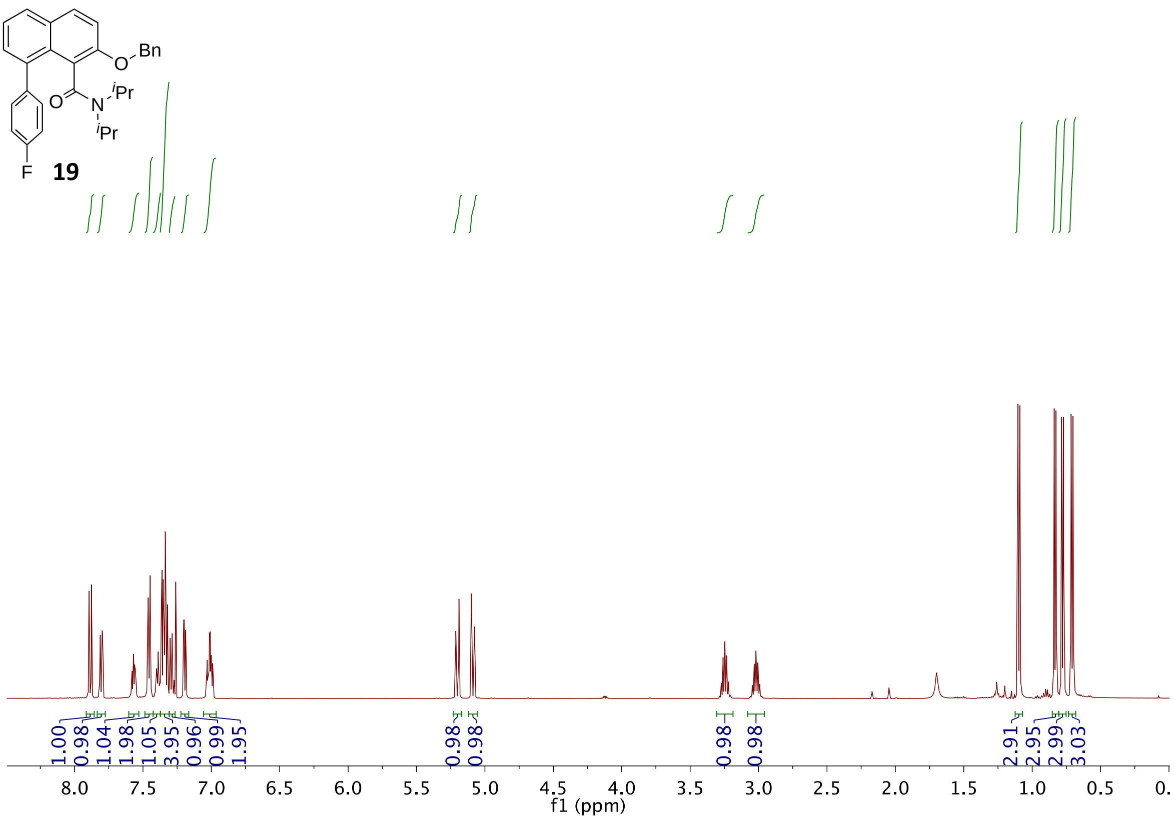
O
Bn
O
N
iPr
iPr
F
19
1.00
0.98
1.04
1.98
1.05
3.95
0.96
0.99
1.95
0.98
0.98
0.98
0.98
2.91
2.95
2.99
3.03
f1 (ppm)

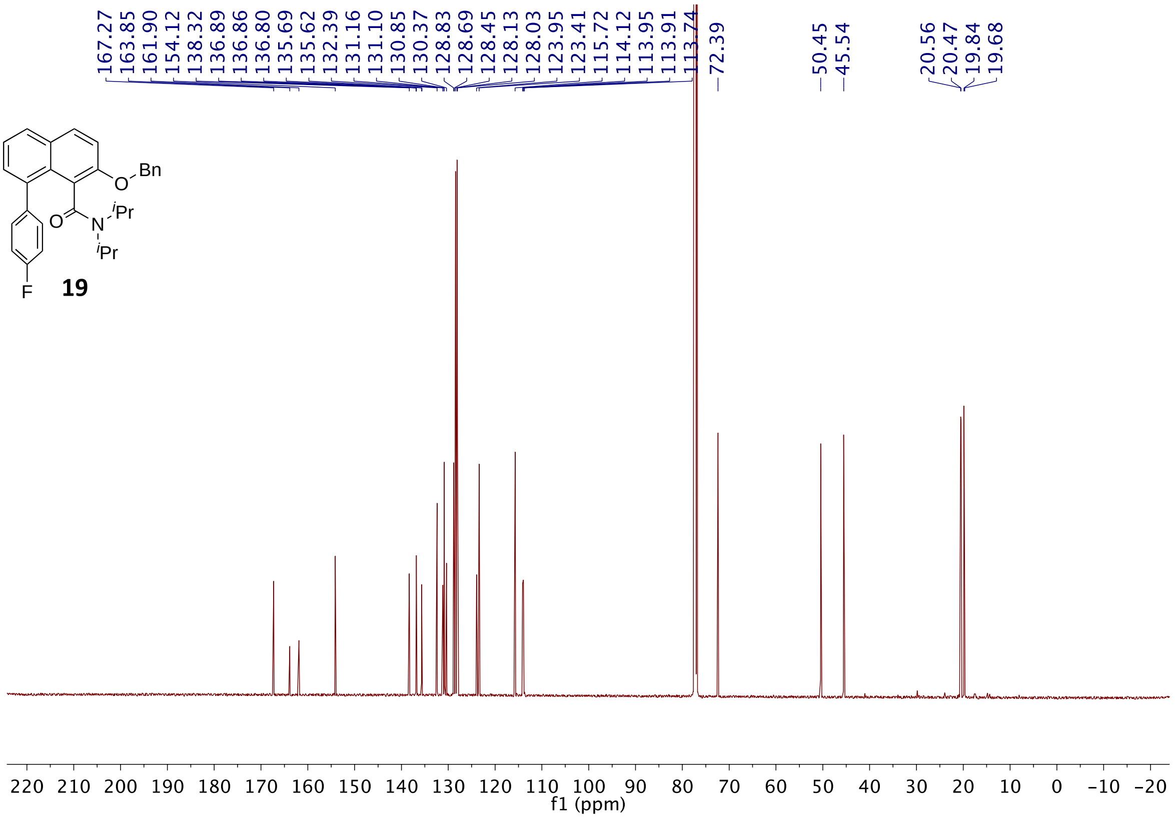
167.27
163.85
161.90
154.12
138.32
136.89
136.86
136.80
135.69
135.62
132.39
131.16
131.10
130.85
130.37
128.83
128.69
128.45
128.13
128.03
123.95
123.41
115.72
114.12
113.95
113.91
113.74
72.39
50.45
45.54
20.56
20.47
19.84
19.68
O
Bn
O
N
iPr
iPr
F
19
f1 (ppm)

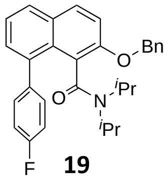
O
Bn
O
N
iPr
iPr
F

19

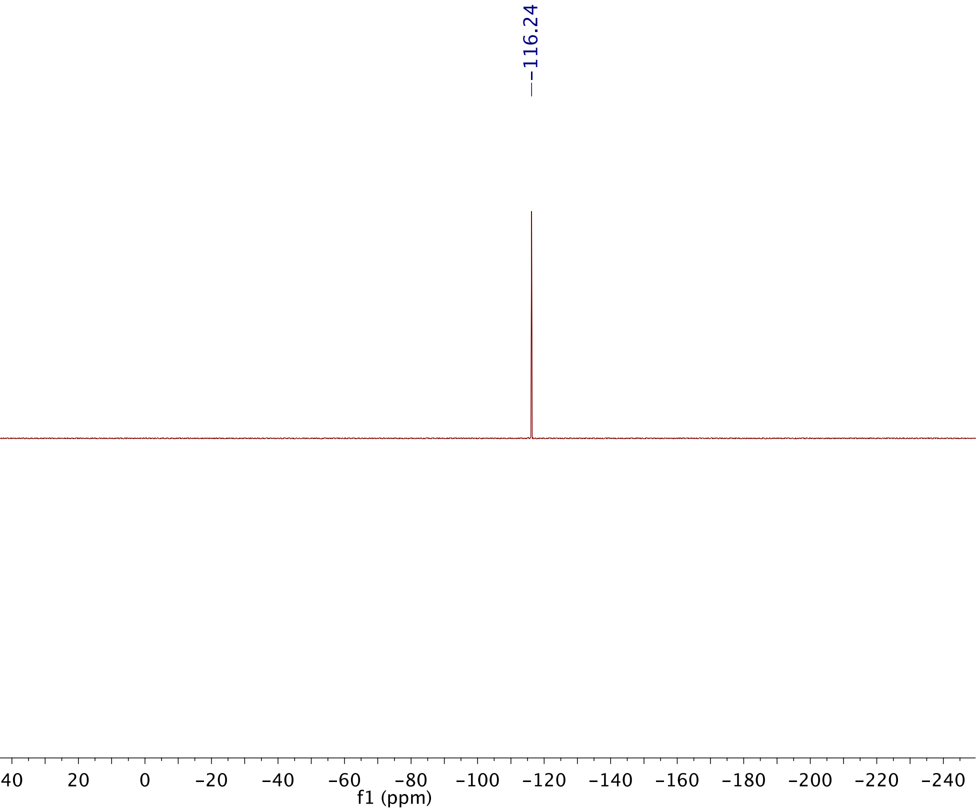
-116.24
80 60 40 20 0 -20 -40 -60 -80 -100 -120 -140 -160 -180 -200 -220 -240
f1 (ppm)

Chiral HPLC: (Chiralpak IC, 10% isopropanol, 90% hexane, 1.0 mL min^{-1}, λ = 240 nm) τ_R (major) = 9.8 min, τ_R (minor) = 13.7 min.

Asymmetric

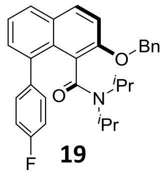

19

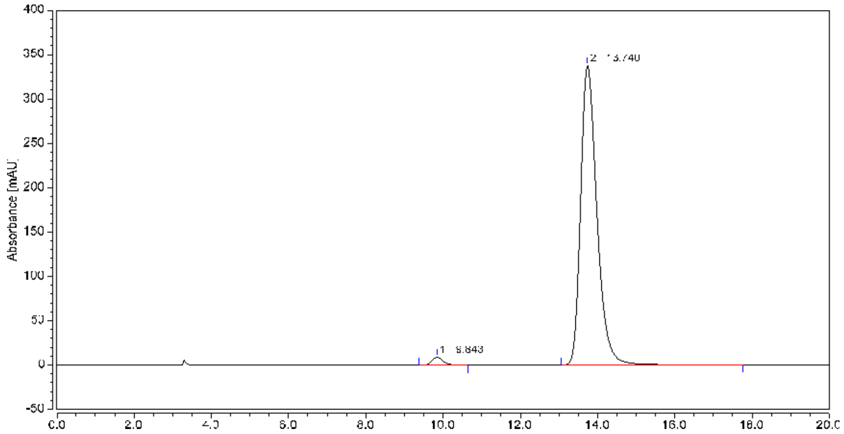

No.	Peak Name	Retention Time	Area	Height	Relative Area
		min	mAU*min	mAU	%
1		9.843	2.753	8.722	1.66
2		13.740	163.520	337.802	98.34
**Total:**			166.273	346.524	100.00

Racemic

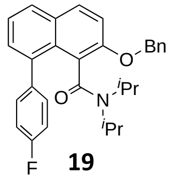

19

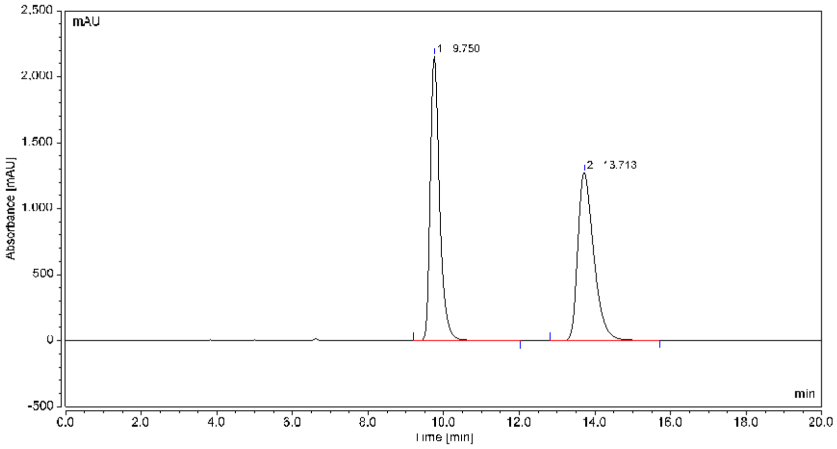

No.	Peak Name	Retention Time	Area	Height	Relative Area
		min	mAU*min	mAU	%
1		9.750	616.400	2158.331	49.86
2		13.713	619.924	1273.828	50.14
**Total:**			1236.324	3432.159	100.00

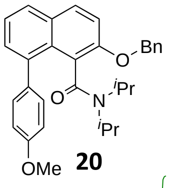
O
Bn
O
N
iPr
iPr
OMe

20

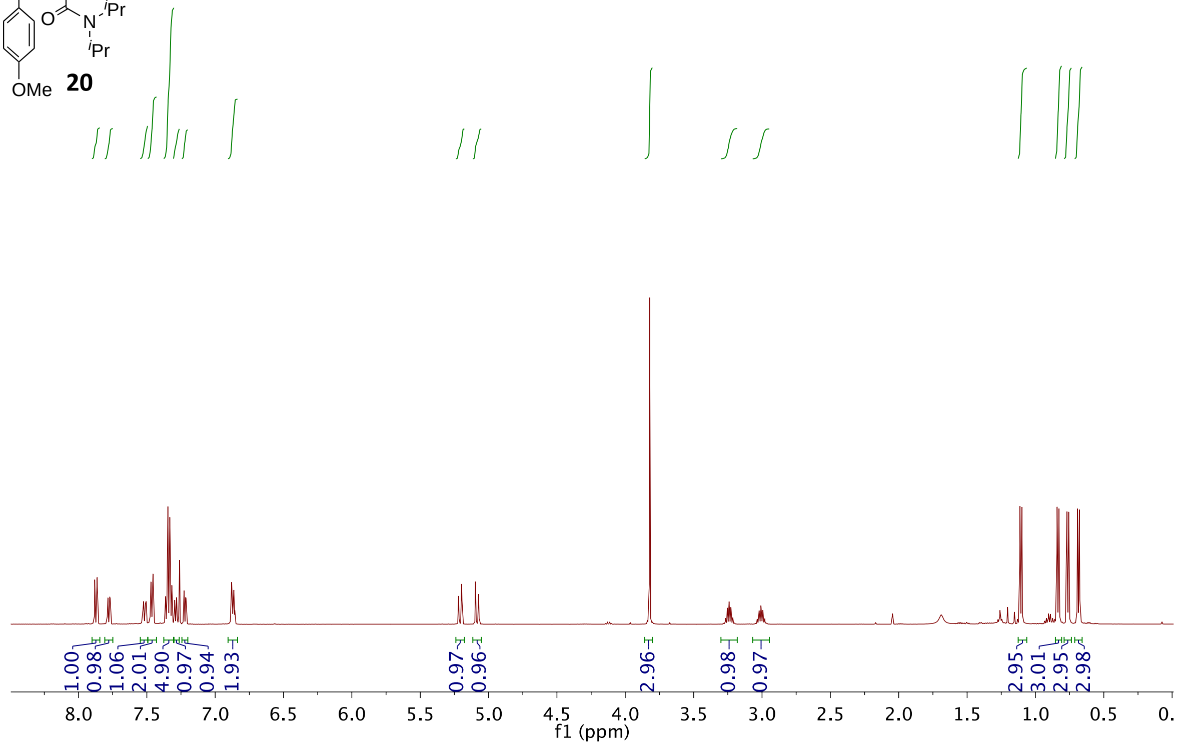
1.00
0.98
1.06
2.01
4.90
0.97
0.94
1.93
0.97
0.96
2.96
0.98
0.97
2.95
3.01
2.95
2.98
8.0 7.5 7.0 6.5 6.0 5.5 5.0 4.5 4.0 3.5 3.0 2.5 2.0 1.5 1.0 0.5 0.
f1 (ppm)

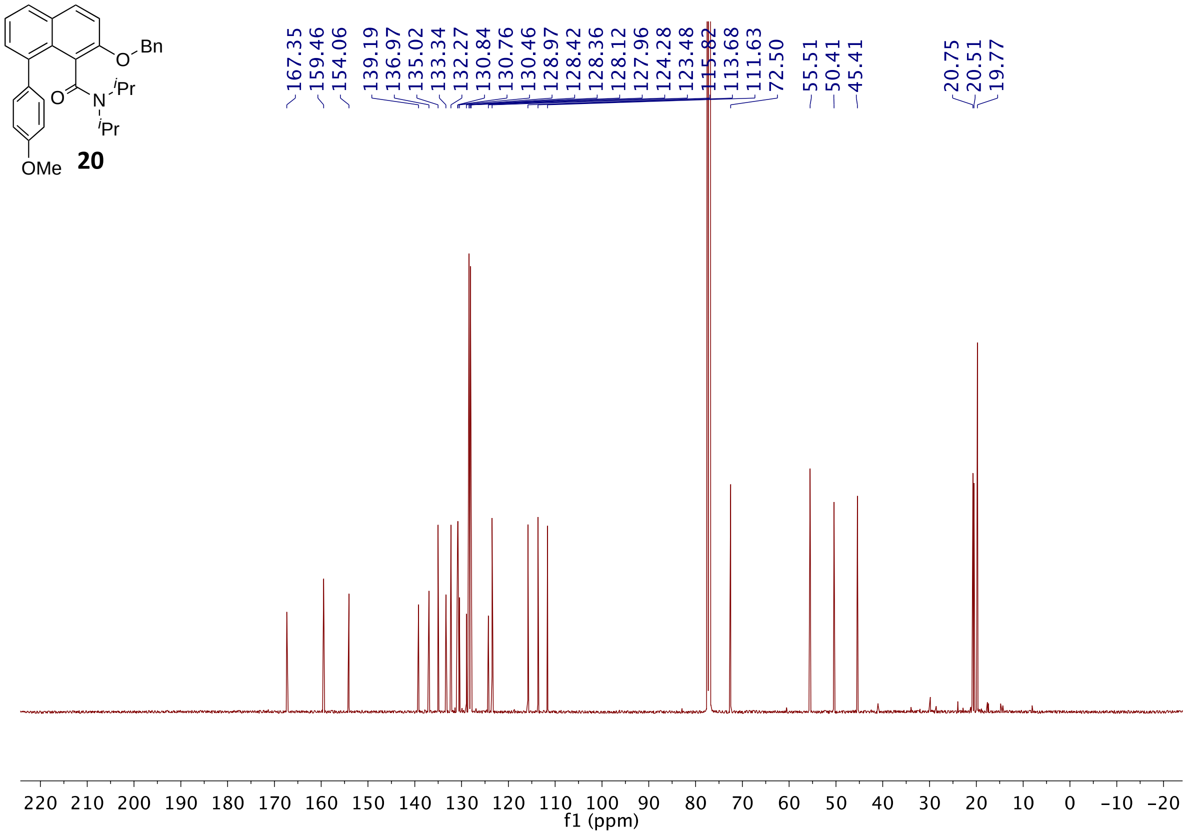
O
Bn
O
N
iPr
iPr
OMe
20
167.35
159.46
154.06
139.19
136.97
135.02
133.34
132.27
130.84
130.76
130.46
128.97
128.42
128.36
128.12
127.96
124.28
123.48
115.82
113.68
111.63
72.50
55.51
50.41
45.41
20.75
20.51
19.77
220 210 200 190 180 170 160 150 140 130 120 110 100 90 80 70 60 50 40 30 20 10 0 −10 −20
f1 (ppm)

Chiral HPLC: (Chiralpak ODH, 30% isopropanol, 70% hexane, 1.0 mL min^{-1}, λ = 260 nm) τ_R (major) = 7.8 min, τ_R (minor) = 12.6 min.

Asymmetric

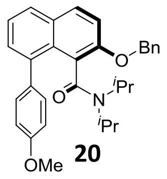

20

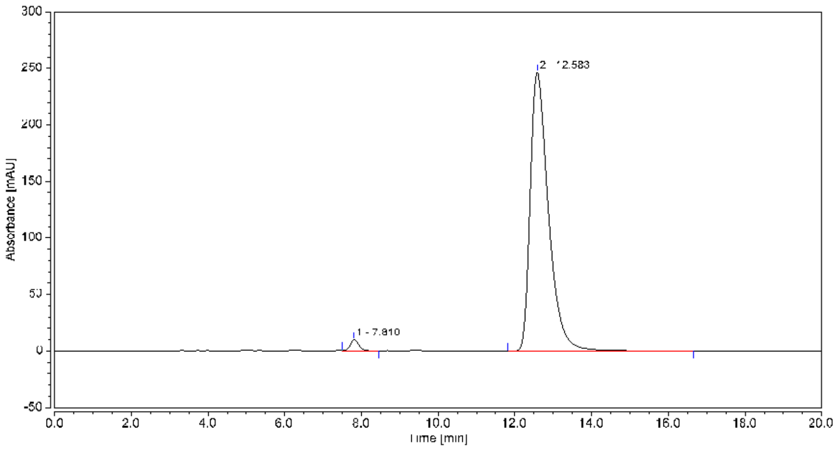

No.	Peak Name	Retention Time	Area	Height	Relative Area
		min	mAU*min	mAU	%
1		7.810	2.756	10.019	1.99
2		12.583	135.385	246.370	98.01
Total:			138.141	256.389	100.00

Racemic

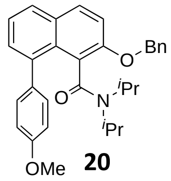

20

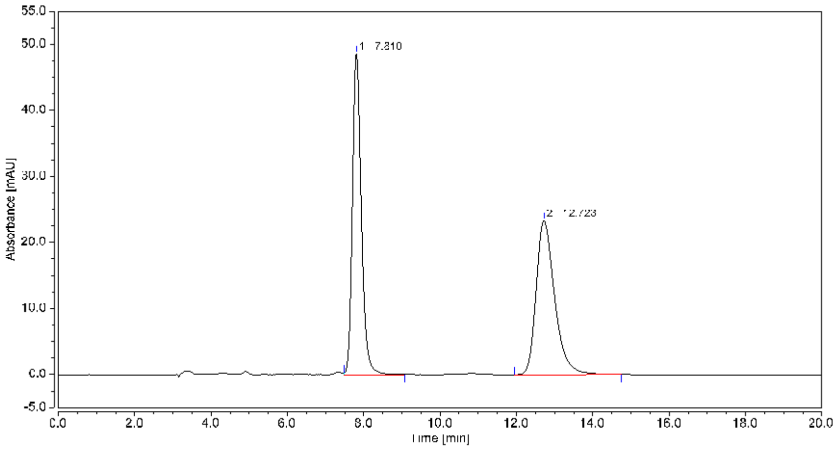

No.	Peak Name	Retention Time	Area	Height	Relative Area
		min	mAU*min	mAU	%
1		7.810	13.213	48.613	50.19
2		12.723	13.114	23.341	49.81
Total:			26.327	71.953	100.00

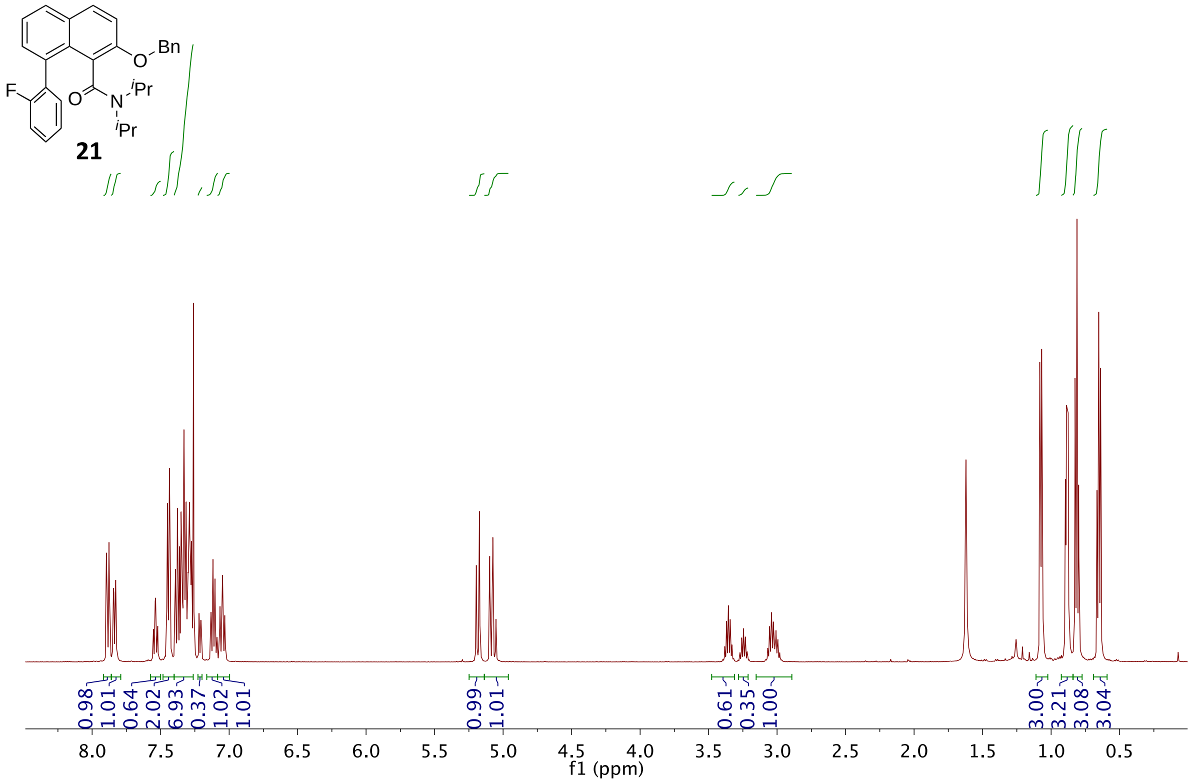
O
Bn
F
O
N
iPr
iPr
21
0.98
1.01
0.64
2.02
6.93
0.37
1.02
1.01
0.99
1.01
0.61
0.35
1.00
3.00
3.21
3.08
3.04
8.0 7.5 7.0 6.5 6.0 5.5 5.0 4.5 4.0 3.5 3.0 2.5 2.0 1.5 1.0 0.5
f1 (ppm)

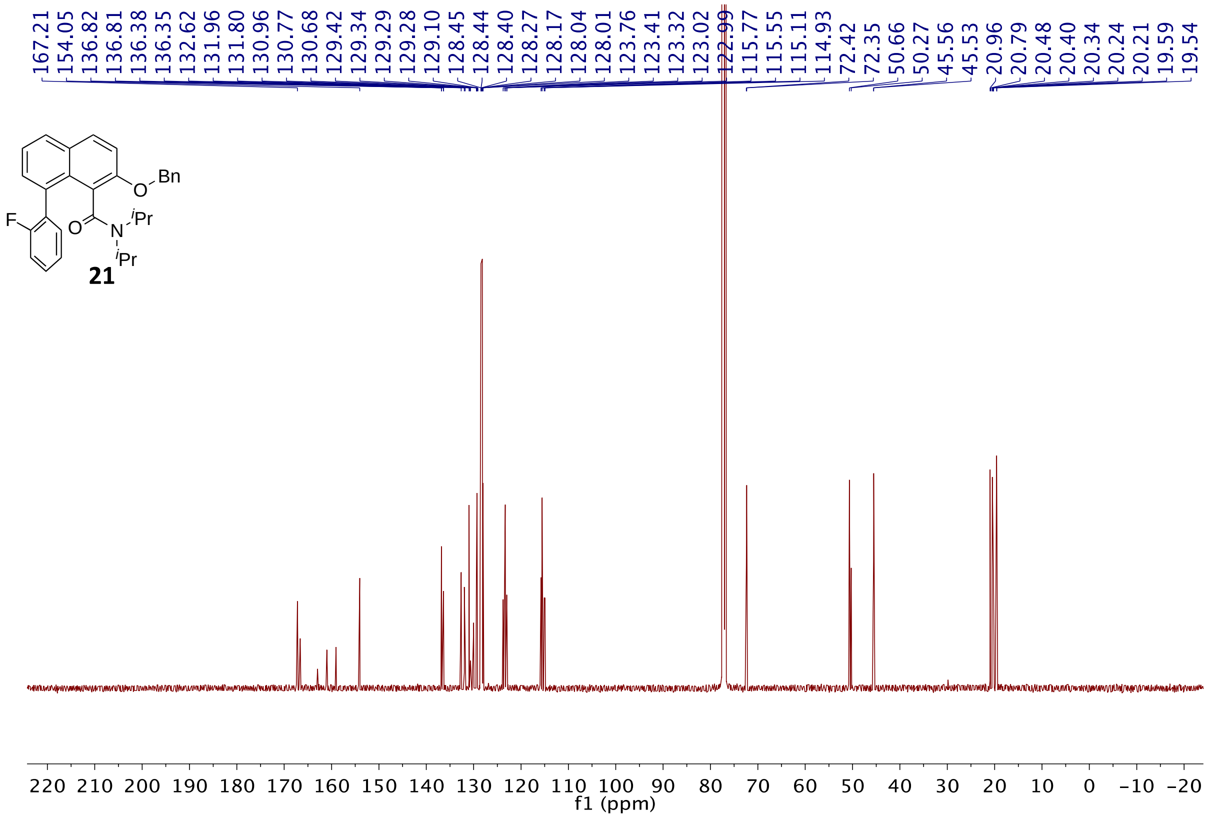
167.21
154.05
136.82
136.81
136.38
136.35
132.62
131.96
131.80
130.96
130.77
130.68
129.42
129.34
129.29
129.28
129.10
128.45
128.44
128.40
128.27
128.17
128.04
128.01
123.76
123.41
123.32
123.02
122.99
115.77
115.55
115.11
114.93
72.42
72.35
50.66
50.27
45.56
45.53
20.96
20.79
20.48
20.40
20.34
20.24
20.21
19.59
19.54
O
Bn
F
O
N
iPr
iPr
21
220 210 200 190 180 170 160 150 140 130 120 110 100 90 80 70 60 50 40 30 20 10 0 −10 −20
f1 (ppm)

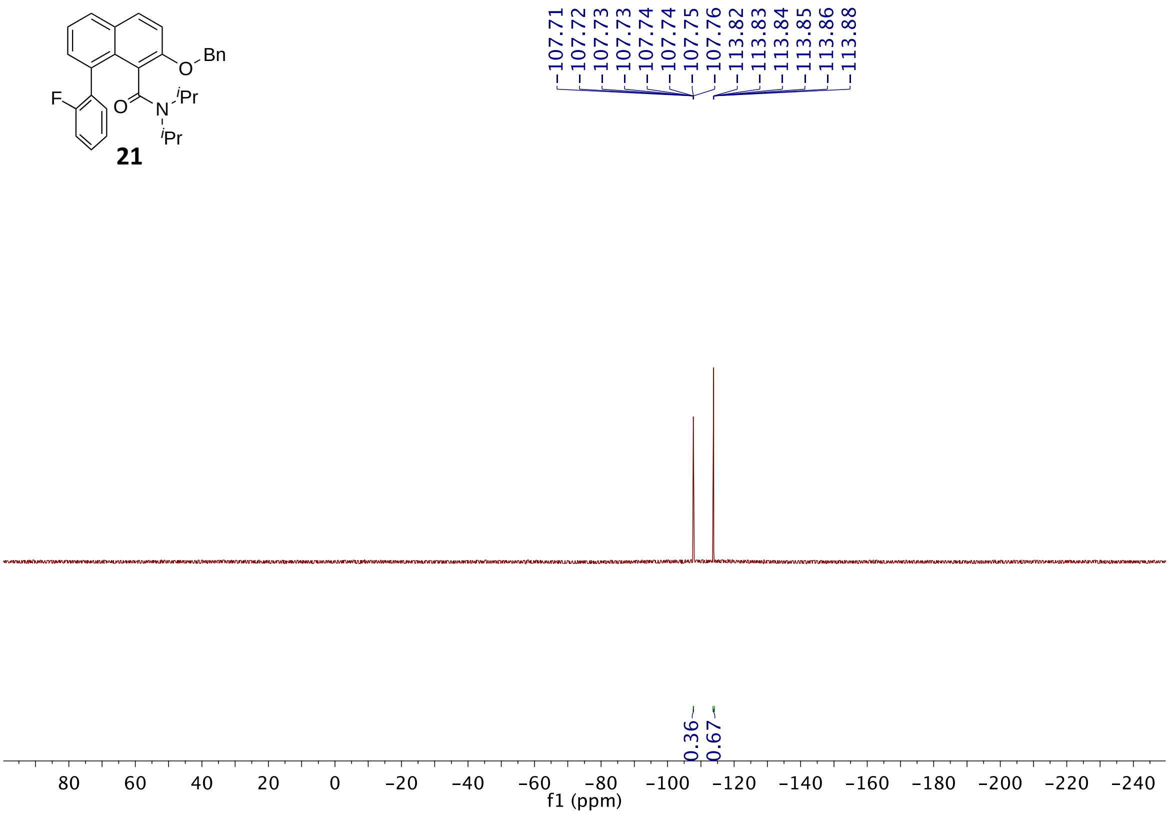
F
O
Bn
O
N
iPr
iPr
21
107.71
107.72
107.73
107.73
107.74
107.74
107.75
107.76
113.82
113.83
113.84
113.85
113.86
113.88
0.36
0.67
80 60 40 20 0 −20 −40 −60 −80 −100 −120 −140 −160 −180 −200 −220 −240
f1 (ppm)

Chiral HPLC: (Chiralpak IC, 10% isopropanol, 90% hexane, 1.0 mL min^{-1}, λ = 260 nm) major diastereomer: τ_R (minor) = 9.9 min, τ_R (major) = 11.7 min, minor diastereomer τ_R (minor) = 21.7 min, τ_R (major) = 37.9 min.

Asymmetric

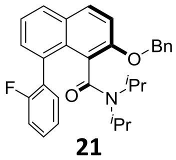

21

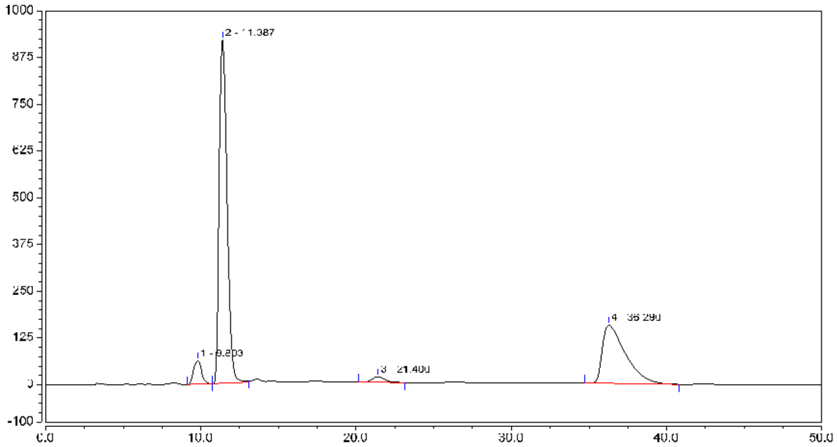

No.	Peak Name	Retention Time	Area	Height	Relative Area
		min	mAU*min	mAU	%
1		9.857	1.061	3.012	3.02
2		11.657	24.824	62.132	70.70
3		21.680	0.358	0.453	1.02
4		37.900	8.867	5.742	25.25
**Total:**			35.109	71.339	100.00

Racemic

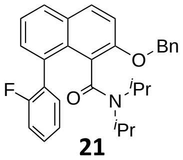

21

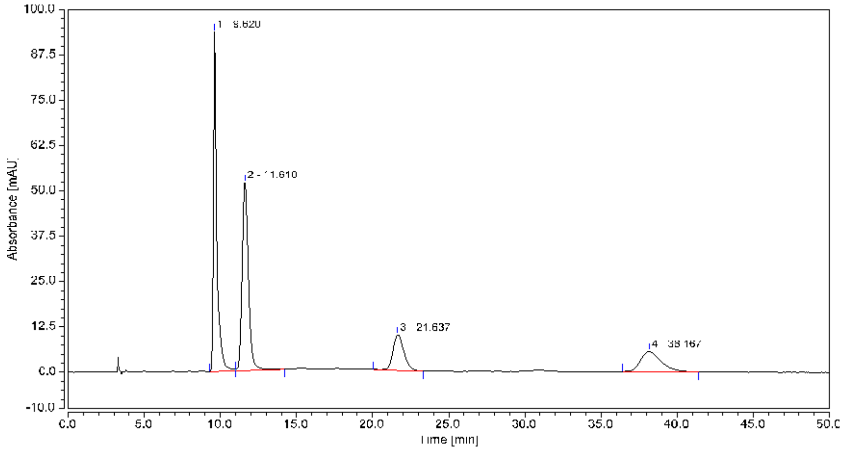

No.	Peak Name	Retention Time	Area	Height	Relative Area
		min	mAU*min	mAU	%
1		9.620	23.767	93.597	36.23
2		11.610	24.788	51.914	37.78
3		21.637	8.214	9.880	12.52
4		38.167	8.838	5.585	13.47
**Total:**			65.607	160.976	100.00

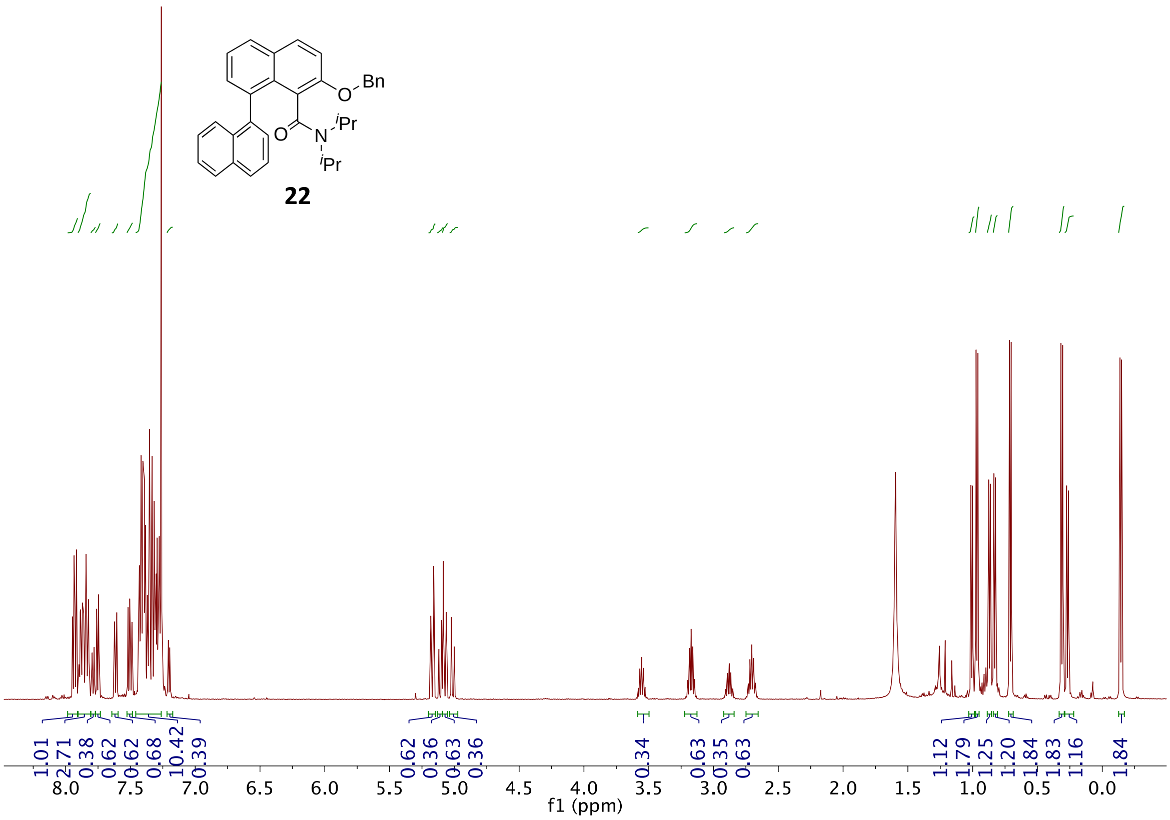
O
Bn
N
iPr
iPr
O
22
1.01
2.71
0.38
0.62
0.62
0.68
10.42
0.39
0.62
0.36
0.63
0.36
0.34
0.63
0.35
0.63
1.12
1.79
1.25
1.20
1.84
1.83
1.16
1.84
8.0 7.5 7.0 6.5 6.0 5.5 5.0 4.5 4.0 3.5 3.0 2.5 2.0 1.5 1.0 0.5 0.0
f1 (ppm)

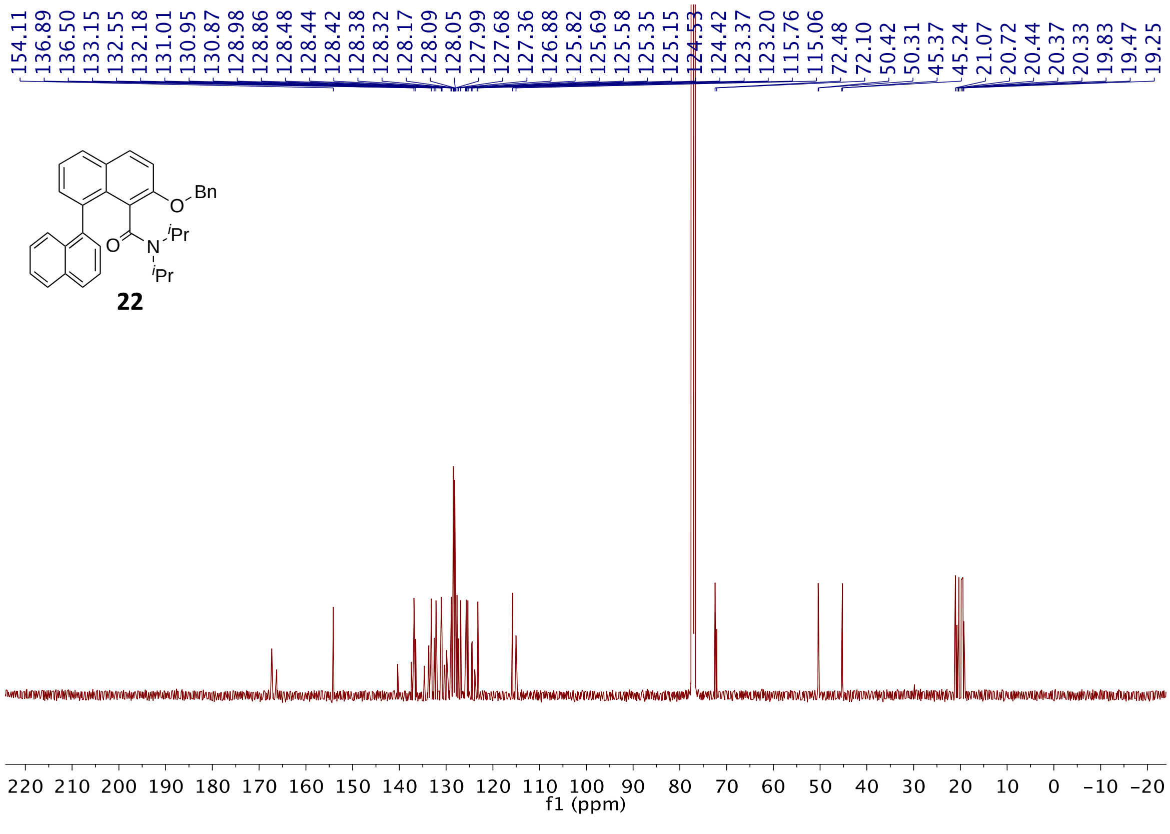
154.11
136.89
136.50
133.15
132.55
132.18
131.01
130.95
130.87
128.98
128.86
128.48
128.44
128.42
128.38
128.32
128.17
128.09
128.05
127.99
127.68
127.36
126.88
125.82
125.69
125.58
125.35
125.15
124.53
124.42
123.37
123.20
115.76
115.06
72.48
72.10
50.42
50.31
45.37
45.24
21.07
20.72
20.44
20.37
20.33
19.83
19.47
19.25
O
Bn
N
iPr
iPr
O
22
220 210 200 190 180 170 160 150 140 130 120 110 100 90 80 70 60 50 40 30 20 10 0 −10 −20
f1 (ppm)

Chiral HPLC: (Chiralpak ADH, 5% isopropanol, 95% hexane, 1.0 mL min^{-1}, λ = 240 nm) minor diastereomer: τ_R (minor) = 11.0 min, τ_R (major) = 12.3 min, minor diastereomer: τ_R (major) = 13.3 min, τ_R (minor) = 19.8 min.

Asymmetric

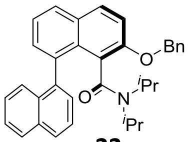

22

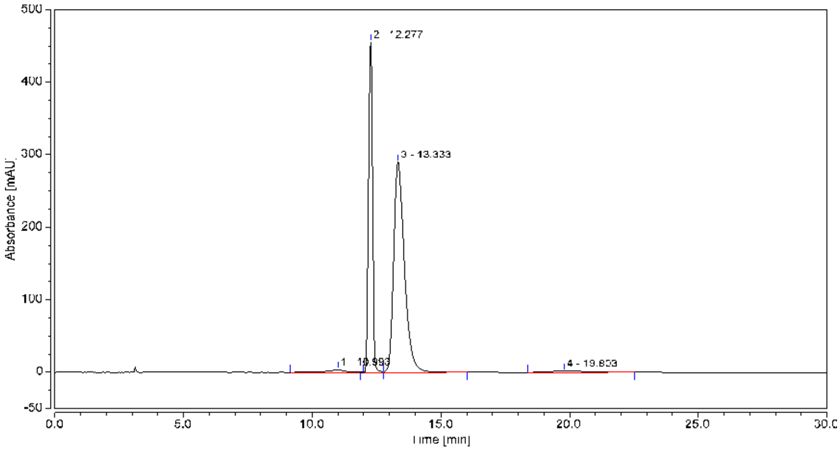

No.	Peak Name	Retention Time	Area	Height	Relative Area
		min	mAU*min	mAU	%
1		10.993	3.259	3.974	1.43
2		12.277	82.392	455.248	36.08
3		13.333	139.427	289.758	61.05
4		19.803	3.297	1.838	1.44
**Total:**			228.375	750.819	100.00

Racemic

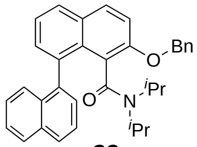

22

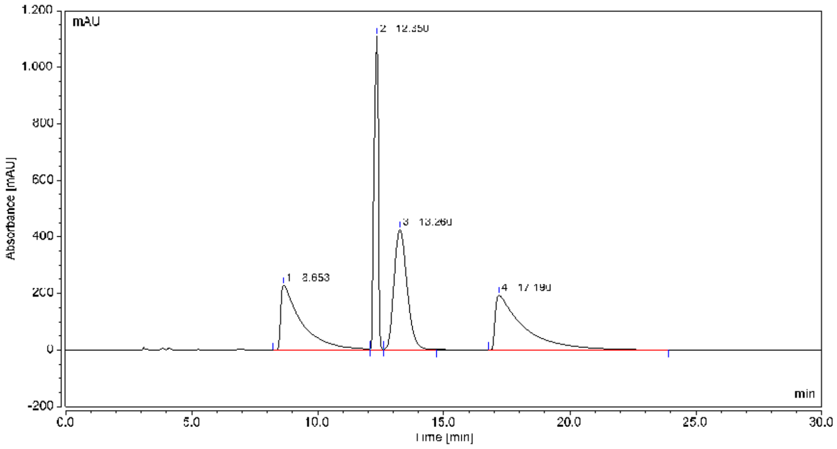

No.	Peak Name	Retention Time	Area	Height	Relative Area
		min	mAU*min	mAU	%
1		8.653	218.758	230.026	23.08
2		12.350	218.019	1109.720	23.00
3		13.260	256.179	426.578	27.02
4		17.190	255.067	194.085	26.91
**Total:**			948.024	1960.409	100.00

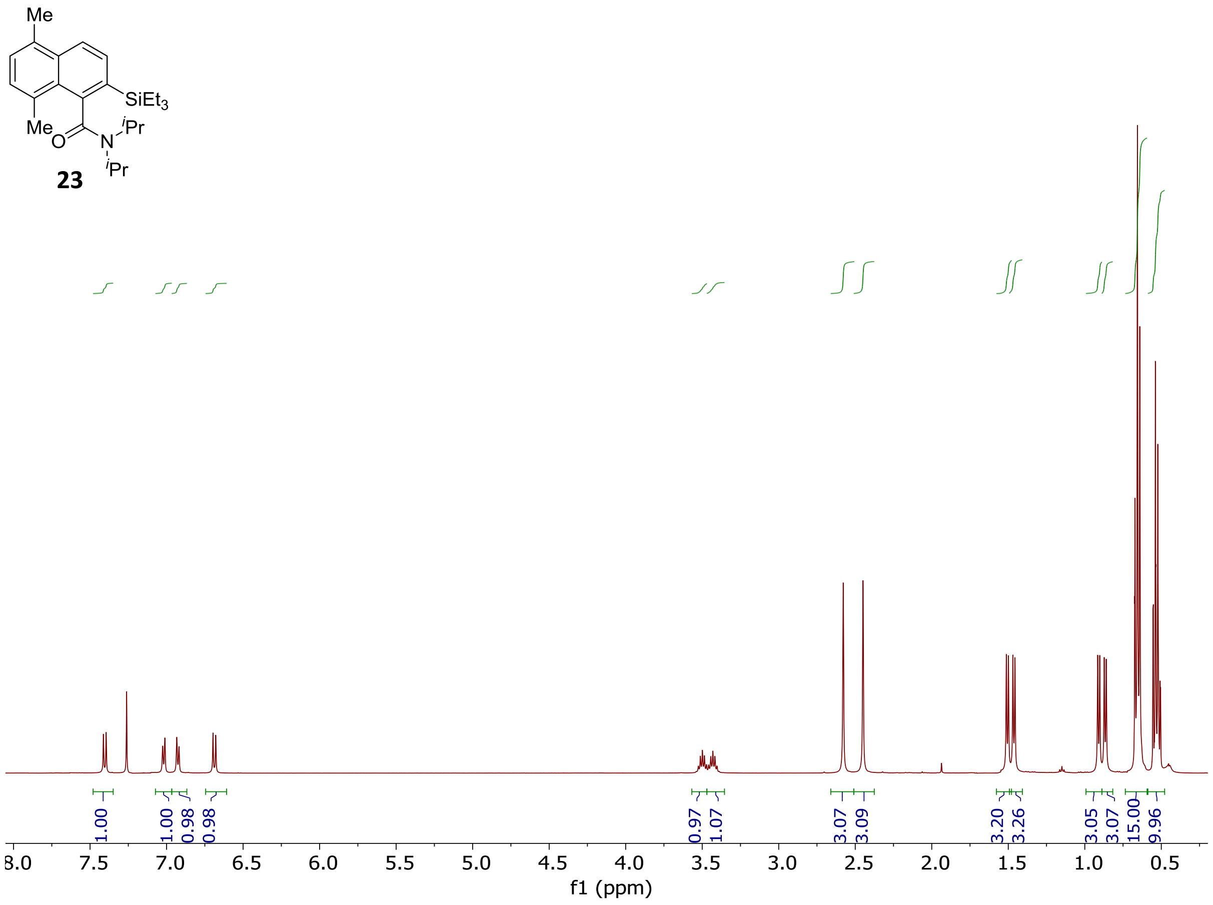
Me
SiEt3
Me
O
N
iPr
iPr
23
1.00
1.00
0.98
0.98
0.97
1.07
3.07
3.09
3.20
3.26
3.05
3.07
15.00
9.96
8.0 7.5 7.0 6.5 6.0 5.5 5.0 4.5 4.0 3.5 3.0 2.5 2.0 1.5 1.0 0.5
f1 (ppm)

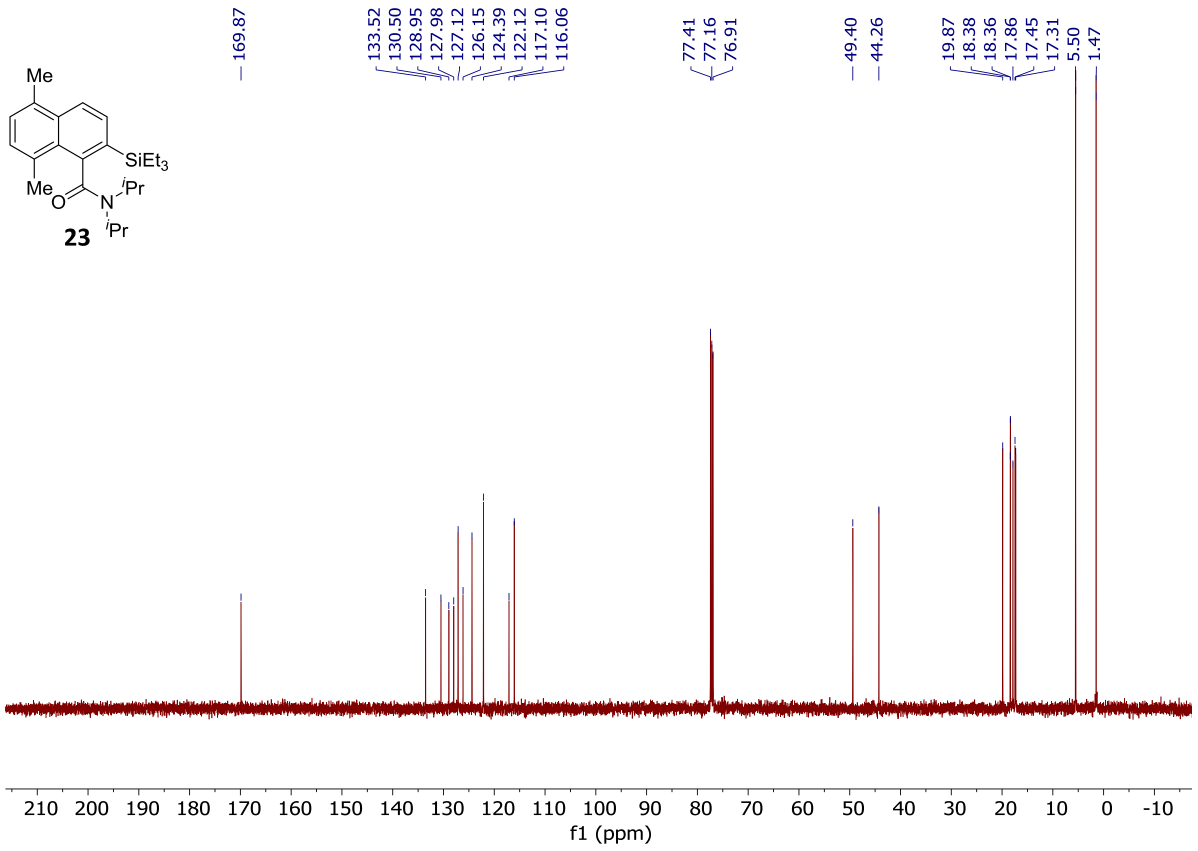
169.87
133.52
130.50
128.95
127.98
127.12
126.15
124.39
122.12
117.10
116.06
77.41
77.16
76.91
49.40
44.26
19.87
18.38
18.36
17.86
17.45
17.31
5.50
1.47
Me
SiEt3
Me
O
N
iPr
iPr
23
210 200 190 180 170 160 150 140 130 120 110 100 90 80 70 60 50 40 30 20 10 0 -10
f1 (ppm)

Chiral HPLC: (Chiralpak ODH, 5% isopropanol, 95% hexane, 1.0 mL min^{-1}, λ = 254 nm) τ_R (major) = 20.2 min, τ_R (minor) = 27.1 min.

Asymmetric

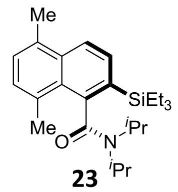

23

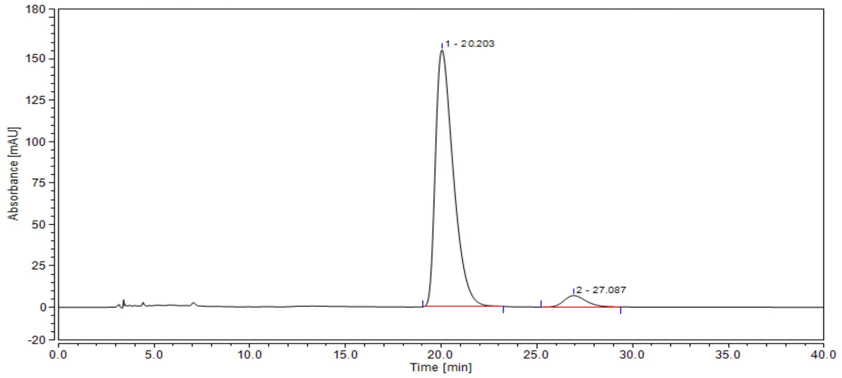

No.	Peak Name	Retention Time	Area	Height	Relative Area
		min	mAU*min	mAU	%
1		20.203	167.622	155.13	94.76
2		27.087	9.2607	6.90	5.24
**Total:**			176.883	162.02	100.00

Racemic

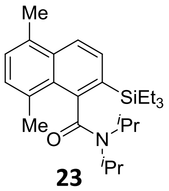

23

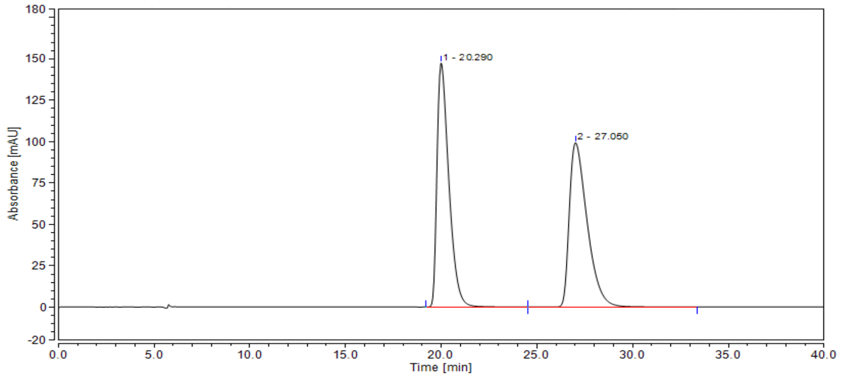

No.	Peak Name	Retention Time	Area	Height	Relative Area
		min	mAU*min	mAU	%
1		20.290	54.254	148.675	49.945
2		27.050	54.373	100.126	50.055
**Total:**			108.627	248.801	100.00

1.9 Computational Methods

Density Functional Theory (DFT) calculations were performed with *Gaussian09*[1] using Truhlar's M06-2X hybrid meta GGA functional[2] and Pople's 6-31G(d) split valence basis set.[3] M06-2X methods were found to give small errors for activation enthalpies when compared against G3B3[4], a composite *ab initio* method derived giving high accuracy.[5,6] Stationary points were optimized with default optimization criteria, tight SCF convergence and ultrafine grid for numerical integration of the exchange-correlation energy and potential. Solvation by dichloromethane (CH_2Cl_2) and toluene were described by a Solvent Model Based on Density Model (SMD).[7] Additionally, high-precision harmonic frequencies for vibrational modes were computed from the Hessian. All transition structures (TSes) were verified by intrinsic reaction coordinate (IRC) calculations connecting them to relevant ground state structures. Reported energies are zero-point corrected electronic energies at 298.15K, using a quasi-RRHO treatment of vibrational entropies introduced by Grimme,[8] unless otherwise stated. All molecular images are produced using *Cylview*.[9]

Single point calculations were carried out using basis set extrapolation of coupled cluster (CC) methods. CC methods like CCSD(T) have been shown to be very successful in calculating ground-state energies when using a large basis set, and has been widely acclaimed as the "gold standard" method for its outstanding accuracy.[10–12] This was performed using the ORCA program 4.0.1[13] with the basis set def2-TZVPD[14] and SMD solvation. Extrapolation to the basis set limit was performed using def2-SVP and def2-TZVP energies, treating the convergence of SCF and correlation energies separately:

(a) The convergence of the HF energy to the basis set limit is calculated as:

$$E_{SCF}^{(X)} = E_{SCF}^{(\infty)} + Ae^{(-\alpha\sqrt{X})} \quad (1)$$

where E_{SCF}^{X} is the SCF energy calculated with the basis set having highest angular momentum X, E_{SCF}^{∞} is the basis set limit SCF energy, α = 10.39 (empirically optimized for def2), and A is a parameter to be determined.

(b) The correlation energy is assumed to converge as:

$$E_{corr}^{(\infty)} = \frac{X^{\beta}E_{corr}^{(X)} - Y^{\beta}E_{corr}^{(Y)}}{X^{\beta} - Y^{\beta}} \quad (2)$$

where $E_{corr}^{(\infty)}$ is the correlation energy calculated with the basis sets having successive highest angular momentums X and Y, and β = 2.40 (empirically optimized for def2).[15] All preparation and analysis was automated through custom Python and Bash scripts.

1.9.1 Rotational Transition Structures

DLPNO-CCSD(T)/def2-TZVPD//M062X/6-31G* with SMD solvation using toluene

Entry (R = H/Me)		Ground state	TS of rotation	ΔG‡ (kJ mol^{-1})	ΔΔG‡ (kJ mol^{-1})	k_{rel}
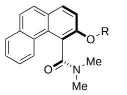	R = H	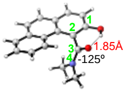	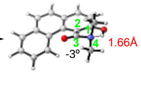	81.2 (CH_2Cl_2)<br>80.7 (Toluene)	45.4 (CH_2Cl_2)	0.0358 (CH_2Cl_2)<br>0.0439 (Toluene)
	R = Me	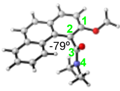	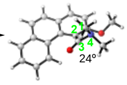	126.7 (CH_2Cl_2)<br>120.9 (Toluene)	40.2 (Toluene)	3.89×10^{-10} (CH_2Cl_2)<br>3.97×10^{-9} (Toluene)
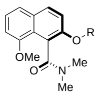	R = H	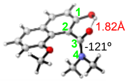	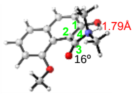	89.7	51.5	1.17×10^{-3}
	R = Me	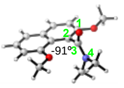	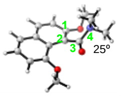	141.2		1.11×10^{-12}
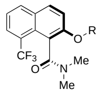	R = H	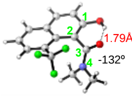	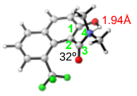	68.8	44.1	5.40
	R = Me	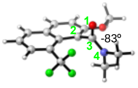	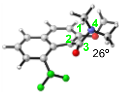	112.9		1.02×10^{-7}

1.9.2 Absolute energies and Cartesian coordinates

Table 2	M06-2X/6-31g* in toluene (SMD)						DLPNO-CCSD(T)/def2-TZVPD (SMD)
	E/au	ZPE/au	H/au	T.qh-S/au	qh-G(T)/au	imag. v	E/au
OH_TS_toluene	-861.709983	0.285263	-861.408626	0.056592	-861.465218	-33.1	-860.8975804
OH_toluene	-861.737922	0.286271	-861.434423	0.058954	-861.493377		-860.9281088
OMe_TS_toluene	-900.968999	0.313652	-900.637333	0.060528	-900.697861	-63.3	-900.1106865
OMe_toluene	-901.010918	0.314648	-900.677193	0.062837	-900.740030		-900.1564912
OH_TS_DCM	-861.714514	0.284846	-861.413567	0.056601	-861.470168	-29.3	-860.9040428
OH_DCM	-861.741787	0.285757	-861.438694	0.059200	-861.497894		-860.9345308
OMe_TS_DCM	-900.974102	0.313648	-900.642558	0.060210	-900.702769	-66.8	-900.1173677
OMe_DCM	-901.017605	0.314547	-900.683996	0.062789	-900.746785		-900.1651021
OH_OMe_TS_toluene	-822.596530	0.271340	-822.308996	0.056721	-822.365717	-66.1	-821.8799457
OH_OMe_toluene	-822.628680	0.272200	-822.339381	0.058640	-822.398022		-821.9139598
OMe_OMe_TS_toluene	-861.853437	0.299315	-861.536207	0.060175	-861.596382	-90.7	-861.0914783
OMe_OMe_toluene	-861.901875	0.299909	-861.582713	0.063298	-861.646011		-861.144060
OH_CF3_TS_toluene	-1045.056302	0.243493	-1044.795686	0.058888	-1044.854574	-45.6	-1044.306627
OH_CF3_toluene	-1045.084009	0.244249	-1044.821994	0.060072	-1044.882066		-1044.333050
OMe_CF3_TS_toluene	-1084.312829	0.271048	-1084.022888	0.062493	-1084.085381	-77.7	-1083.517278
OMe_CF3_toluene	-1084.355593	0.272405	-1084.063315	0.064618	-1084.127933		-1083.560477

OH_TS_toluene

C	-3.72247	-2.12444	-0.24171
C	-3.91205	-0.86893	0.28241
C	-2.85734	0.07336	0.31353
C	-1.55689	-0.30281	-0.12145
C	-1.41976	-1.57577	-0.72757
C	-2.46904	-2.46329	-0.78588
C	-3.13151	1.43040	0.67927
C	-0.47712	0.67609	-0.06708
C	-0.85547	2.02756	0.09237

C	-2.18328	2.37990	0.49831
C	0.08712	3.05784	-0.15950
H	-0.23570	4.09117	-0.05931
C	1.35613	2.77214	-0.56466
C	1.80379	1.42766	-0.57659
C	0.93024	0.37313	-0.23063
H	-4.13139	1.68964	1.01631
H	-4.54146	-2.83686	-0.27335
H	-4.88780	-0.56278	0.65075
H	-0.47763	-1.84863	-1.18273
H	-2.33013	-3.42746	-1.26524
H	-2.40100	3.43035	0.67323
H	2.07594	3.54483	-0.81197
O	3.09308	1.27292	-0.88395
H	3.29417	0.29222	-0.77393
C	1.49179	-0.93285	0.20055
O	0.86344	-1.85269	0.67278
N	2.94581	-1.05485	0.10982
C	3.54499	-0.61008	1.38338
H	3.24283	-1.26813	2.20845
H	4.63340	-0.62875	1.28588
H	3.23268	0.41251	1.60956
C	3.34961	-2.43049	-0.19347
H	2.87899	-2.75112	-1.12551
H	4.43445	-2.44531	-0.32360
H	3.06721	-3.12608	0.60455

OMe_TS_toluene

C	-4.22160	-1.66490	-0.39409
C	-4.21600	-0.45588	0.26381
C	-3.04346	0.32771	0.32953
C	-1.82660	-0.17347	-0.20803
C	-1.88810	-1.37603	-0.94946
C	-3.05370	-2.10623	-1.03873
C	-3.10940	1.66731	0.83988
C	-0.61867	0.64041	-0.11839
C	-0.79071	2.01271	0.20490
C	-2.04748	2.49388	0.70902
C	0.28193	2.91563	0.05837
H	0.11101	3.96551	0.28254
C	1.50541	2.49798	-0.40271
C	1.72623	1.12246	-0.58373
C	0.72640	0.17464	-0.33186
H	-4.05037	2.02158	1.25181
H	-5.13275	-2.25294	-0.45117
H	-5.12686	-0.06427	0.70941
H	-1.00892	-1.71370	-1.47850
H	-3.06865	-3.02165	-1.62190
H	-2.10933	3.53807	1.00425
H	2.30263	3.20776	-0.59327
O	2.91464	0.67090	-1.04838
C	1.15858	-1.20728	0.08515
O	0.43231	-2.17356	0.13734
N	2.49199	-1.28770	0.65795
C	2.40994	-1.90595	1.98216
H	2.05569	-2.94577	1.95044
H	3.40801	-1.88351	2.43016
H	1.73597	-1.32413	2.61756
C	3.36196	-2.08389	-0.20988
H	3.39203	-1.64428	-1.20867
H	4.37471	-2.07640	0.20625
H	3.02284	-3.12908	-0.28704
C	4.08921	1.19308	-0.44207
H	4.29053	2.21946	-0.76697
H	4.00890	1.14939	0.64828
H	4.91142	0.55615	-0.77363

OH_TS_DCM

C	-3.73975	-2.10532	-0.24279
C	-3.91567	-0.85447	0.29936
C	-2.85401	0.08121	0.32927
C	-1.55974	-0.29984	-0.12098
C	-1.43742	-1.56536	-0.74726
C	-2.49430	-2.44519	-0.80676
C	-3.11715	1.43820	0.70660
C	-0.47209	0.67146	-0.06735
C	-0.84289	2.02513	0.09658
C	-2.16523	2.38355	0.51770
C	0.10110	3.05201	-0.16575
H	-0.21612	4.08672	-0.06245
C	1.36461	2.75787	-0.58451
C	1.80543	1.41106	-0.59455
C	0.93230	0.36071	-0.23773
H	-4.11215	1.70051	1.05574
H	-4.56389	-2.81193	-0.27462
H	-4.88547	-0.54540	0.68089
H	-0.50170	-1.83644	-1.21691
H	-2.36817	-3.40294	-1.30261
H	-2.37416	3.43508	0.69688
H	2.08460	3.52771	-0.84148
O	3.09211	1.24843	-0.91486
H	3.29462	0.26924	-0.79540
C	1.49304	-0.95149	0.18421
O	0.85181	-1.88470	0.61586
N	2.94134	-1.06303	0.12715
C	3.52800	-0.55670	1.38623
H	3.23445	-1.18952	2.23376
H	4.61645	-0.56304	1.29138
H	3.19734	0.46719	1.57497
C	3.37126	-2.44347	-0.11536
H	2.91078	-2.81327	-1.03392
H	4.45668	-2.44109	-0.23911
H	3.10116	-3.10762	0.71324

OMe_TS_DCM

C	-4.23892	-1.61053	-0.44820
C	-4.22147	-0.41237	0.23059
C	-3.03794	0.35386	0.31853
C	-1.82384	-0.15585	-0.21848
C	-1.89699	-1.34397	-0.98350
C	-3.07218	-2.05706	-1.09365
C	-3.08956	1.68604	0.85177
C	-0.60446	0.63878	-0.10597
C	-0.76009	2.00804	0.23875

C	-2.01403	2.49933	0.74131
C	0.33006	2.89524	0.12513
H	0.17543	3.94178	0.37542
C	1.55308	2.46992	-0.33249
C	1.75109	1.09472	-0.54942
C	0.73388	0.15581	-0.31661
H	-4.02887	2.04603	1.26262
H	-5.15784	-2.18466	-0.52214
H	-5.12982	-0.01515	0.67644
H	-1.01837	-1.68271	-1.51337
H	-3.09667	-2.96087	-1.69475
H	-2.06154	3.53963	1.05275
H	2.36395	3.17271	-0.48588
O	2.92058	0.61637	-1.01243
C	1.14305	-1.23307	0.09202
O	0.40759	-2.19611	0.10567
N	2.45404	-1.33229	0.71010
C	2.31571	-1.96552	2.02401
H	1.95754	-3.00324	1.96666
H	3.29570	-1.95627	2.51038
H	1.62061	-1.38657	2.63934
C	3.34825	-2.13175	-0.13184
H	3.43167	-1.67978	-1.12173
H	4.34049	-2.14983	0.32994
H	2.99359	-3.16931	-0.23899
C	4.11835	1.29341	-0.65111
H	4.24850	2.20989	-1.23557
H	4.12429	1.52628	0.41833
H	4.93373	0.60572	-0.88114

OH_OMe_TS_toluene

C	3.66955	-0.28533	0.76563
C	0.96119	-0.29363	-0.10128
C	1.67186	-1.50008	0.15949
C	3.01119	-1.47872	0.61772
C	1.03107	-2.74682	-0.09676
H	1.59400	-3.66267	0.06389
C	-0.23865	-2.79474	-0.57688
C	-1.01699	-1.60549	-0.65338
C	-0.47504	-0.35698	-0.32918
H	4.68877	-0.25448	1.13786
H	3.50003	-2.42291	0.84064
H	-0.73047	-3.72910	-0.82530
O	-2.29635	-1.82553	-1.00190
H	-2.78696	-0.97660	-0.87598
C	-1.39781	0.79243	-0.02446
O	-1.11692	1.96928	0.00461
N	-2.75200	0.40788	0.25590
C	-2.94330	-0.42352	1.45659
H	-2.89914	0.19658	2.36184
H	-3.92307	-0.90593	1.40770
H	-2.17755	-1.19536	1.52869
C	-3.67525	1.53964	0.25941
H	-3.57557	2.10010	-0.67074
H	-4.69257	1.14755	0.33655
H	-3.48563	2.22005	1.09850
C	3.05188	0.90296	0.33552
H	3.62285	1.82491	0.31508
C	1.76095	0.89370	-0.16112
O	1.24993	1.94405	-0.83640
C	1.63589	3.24380	-0.44699
H	1.61665	3.35205	0.64347
H	2.63452	3.50108	-0.82216
H	0.90561	3.92387	-0.88767

OMe_OMe_TS_toluene

C	-3.76021	0.91591	0.71262
C	-1.09043	0.39517	-0.09430
C	-1.52416	1.70703	0.25483
C	-2.85726	1.94348	0.68220
C	-0.61282	2.79104	0.14480
H	-0.96575	3.79005	0.38727
C	0.65784	2.60075	-0.31734
C	1.12379	1.28379	-0.54497
C	0.32176	0.17093	-0.31434
H	-4.77738	1.08236	1.05378
H	-3.13899	2.95017	0.97716
H	1.32180	3.44174	-0.48457
O	2.36795	1.08013	-1.04161
C	1.01443	-1.12545	0.03120
O	0.52653	-2.22852	0.01486
N	2.33743	-0.94153	0.62377
C	2.33594	-1.52364	1.96536
H	2.15916	-2.60929	1.96222
H	3.30712	-1.32438	2.42890
H	1.56208	-1.04403	2.57270
C	3.35175	-1.58895	-0.20621
H	3.33079	-1.15932	-1.21044
H	4.33732	-1.40281	0.23359
H	3.20062	-2.67758	-0.27901
C	-3.39852	-0.35547	0.22075
H	-4.15697	-1.12495	0.13272
C	-2.11686	-0.59819	-0.23270
O	-1.78044	-1.70546	-0.92006
C	-2.59640	-2.84816	-0.80038
H	-2.78767	-3.09178	0.25146
H	-3.55218	-2.71954	-1.32447
H	-2.04176	-3.66403	-1.26535
C	3.44000	1.76510	-0.40950
H	4.35687	1.32757	-0.80890
H	3.43346	2.83505	-0.64435
H	3.40415	1.61222	0.67334

OH_CF3_TS_toluene

C	-3.07846	1.54571	1.18991
C	-0.61765	0.69923	0.00305
C	-0.96215	2.08045	0.14702
C	-2.17344	2.47817	0.75920
C	-0.08122	3.08587	-0.34230
H	-0.39592	4.12366	-0.27261
C	1.11319	2.76342	-0.90438
C	1.56275	1.41900	-0.84132
C	0.75693	0.39838	-0.33709
H	-3.99612	1.83609	1.68959

H	-2.36388	3.54139	0.88067
H	1.78790	3.50811	-1.31201
O	2.83478	1.25068	-1.25026
H	3.12291	0.34331	-1.02064
C	1.40646	-0.90767	0.02927
O	0.89224	-1.99924	-0.08929
N	2.73056	-0.81274	0.48995
C	3.11173	0.18903	1.49030
H	3.01854	-0.23618	2.49759
H	4.15062	0.49256	1.33335
H	2.47500	1.07068	1.43094
C	3.38451	-2.09796	0.71184
H	3.22897	-2.74579	-0.15048
H	4.45416	-1.91980	0.84744
H	2.98822	-2.60318	1.60141
C	-2.85359	0.20140	0.85167
H	-3.65084	-0.51555	1.01389
C	-1.69529	-0.22706	0.23105
C	-1.82521	-1.60494	-0.39756
F	-1.51678	-2.62318	0.41258
F	-1.14446	-1.71033	-1.54013
F	-3.12108	-1.81714	-0.73870

OMe_CF3_TS_toluene

C	-3.17682	1.81468	1.07778
C	-0.76050	0.72252	0.04203
C	-0.91197	2.12348	0.27943
C	-2.11419	2.64012	0.81965
C	0.14757	3.01327	-0.05105
H	-0.00892	4.07752	0.10418
C	1.31152	2.56055	-0.60248
C	1.52420	1.16304	-0.69343
C	0.56956	0.25276	-0.25419
H	-4.09560	2.20092	1.50550
H	-2.17509	3.70713	1.01751
H	2.07919	3.25099	-0.93361
O	2.65371	0.66421	-1.23972
C	1.04938	-1.07387	0.23965
O	0.35315	-2.05026	0.38141
N	2.40482	-1.05057	0.75897
C	2.34272	-1.14810	2.21716
H	1.92006	-2.10364	2.56338
H	3.35561	-1.04301	2.61783
H	1.73166	-0.33070	2.61389
C	3.19326	-2.13893	0.18999
H	3.22395	-2.03128	-0.89799
H	4.21576	-2.06514	0.57382
H	2.79184	-3.13261	0.43977
C	-3.10289	0.47211	0.66559
H	-3.99931	-0.13904	0.70229
C	-1.95099	-0.06894	0.13057
C	-2.16210	-1.39238	-0.58042
F	-2.21054	-2.45582	0.22845
F	-1.27146	-1.61284	-1.55051
F	-3.36894	-1.36857	-1.19755
C	3.88851	1.22011	-0.80655
H	3.93834	1.22619	0.28673
H	4.66809	0.56787	-1.20316
H	4.04061	2.22972	-1.20235

1.9.3 References

1. Schmid, M. *et al.* References. *Cytogenet. Genome Res.* **138,** 341–367 (2012).

2. Lin, H. & Truhlar, D. G. QM/MM: What have we learned, where are we, and where do we go from here? *Theor. Chem. Acc.* **117,** 185–199 (2007).

3. Hehre, W. J., Ditchfield, R. & Pople, J. A. Self-Consistent Molecular Orbital Methods. 12. Further extensions of Gaussian-type basis sets for use in molecular-orbital studies of organic-molecules. *J. Chem. Phys.* **56,** 2257–2261 (1972).

4. Baboul, A. G., Curtiss, L. A., Redfern, P. C. & Raghavachari, K. Gaussian-3 theory using density functional geometries and zero-point energies. *J. Chem. Phys.* **110,** 7650–7657 (1999).

5. Bachrach, S. M. DFT study of [2.2]-, [3.3]-, and [4.4]paracyclophanes: Strain energy, conformations, and rotational barriers. *J. Phys. Chem. A* **115,** 2396–2401 (2011).

6. Wentrup, C., Koch, R. & Kleinpeter, E. Twisted C=C Double Bonds with Very Low Rotational Barriers in Dioxanediones and Isoxazolones Determined by Low-Temperature Dynamic NMR Spectroscopy and Computational Chemistry. *European J. Org. Chem.* **2016,** 4985–4990 (2016).

7. Mennucci, B., Cancès, E. & Tomasi, J. Evaluation of Solvent Effects in Isotropic and Anisotropic Dielectrics and in Ionic Solutions with a Unified Integral Equation Method: Theoretical Bases, Computational Implementation, and Numerical Applications. *J. Phys. Chem. B* **101,** 10506–10517 (1997).

8. Grimme, S. Supramolecular binding thermodynamics by dispersion-corrected density functional theory. *Chem. - A Eur. J.* (2012). doi:10.1002/chem.201200497

9. Legault, C. Y. CYLview User Manual. *Comput. Programs Biomed.* **18,** 99–108 (2010).

10. Řezáč, J., Šimová, L. & Hobza, P. CCSD[T] Describes Noncovalent Interactions Better than the CCSD(T), CCSD(TQ), and CCSDT Methods. *J. Chem. Theory Comput.* **9,** 364–369 (2013).

11. Čížek, J. On the Correlation Problem in Atomic and Molecular Systems. Calculation of Wavefunction Components in Ursell-Type Expansion Using Quantum-Field Theoretical Methods. *J. Chem. Phys.* **45,** 4256 (1966).

12. Čížek, J. and J. P. Coupled Cluster Approach. *Phys. Scr.* **21,** 251–254 (1980).

13. Neese, F. The ORCA program system. *Wiley Interdiscip. Rev. Comput. Mol. Sci.* **2,** 73–78 (2012).

14. Weigend, F. & Ahlrichs, R. Balanced basis sets of split valence, triple zeta valence and quadruple zeta valence quality for H to Rn: Design and assessment of accuracy. *Phys. Chem. Chem. Phys.* **7,** 3297 (2005).

15. Neese, F., Hansen, A. & Liakos, D. G. Efficient and accurate approximations to the local coupled cluster singles doubles method using a truncated pair natural orbital basis. *J. Chem. Phys.* **131,** (2009).
